# Association Between Exposure to Per- and Polyfluoroalkyl Substances and Birth Outcomes: A Systematic Review and Meta-Analysis

**DOI:** 10.3389/fpubh.2022.855348

**Published:** 2022-03-24

**Authors:** Si-Yu Gui, Yue-Nan Chen, Ke-Jia Wu, Wen Liu, Wen-Jing Wang, Huan-Ru Liang, Zheng-Xuan Jiang, Ze-Lian Li, Cheng-Yang Hu

**Affiliations:** ^1^Department of Ophthalmology, The Second Affiliated Hospital of Anhui Medical University, Hefei, China; ^2^Department of Clinical Medicine, The Second School of Clinical Medicine, Anhui Medical University, Hefei, China; ^3^Department of Pharmacy, School of Clinical Pharmacy, Anhui Medical University, Hefei, China; ^4^Department of Clinical Medicine, The First School of Clinical Medicine, Anhui Medical University, Hefei, China; ^5^Department of General Surgery, The Second Affiliated Hospital of Anhui Medical University, Hefei, China; ^6^Department of Gynecology and Obstetrics, The First Affiliated Hospital of Anhui Medical University, Hefei, China; ^7^Department of Humanistic Medicine, School of Humanistic Medicine, Anhui Medical University, Hefei, China; ^8^Department of Epidemiology and Biostatistics, School of Public Health, Anhui Medical University, Hefei, China

**Keywords:** per- and polyfluoroalkyl substances, birth outcome, birth weight, systematic review, meta-analysis

## Abstract

**Background:**

A large body of emerging evidence suggests that per- and polyfluoroalkyl substances (PFAS) affect birth outcomes in various pathways, but the evidence is inconsistent. Therefore, this study aimed to systematically review the epidemiological evidence on PFAS exposure and birth outcomes.

**Methods:**

Three electronic databases were searched for epidemiological studies through February 13, 2021. We used random-effects meta-analysis for eight birth outcome indicators to calculate summary effect estimates for various exposure types. The risk of bias and the overall quality and level of evidence for each exposure-outcome pair were assessed.

**Results:**

The initial search identified 58 potentially eligible studies, of which 46 were ultimately included. Many PFAS were found to have previously unrecognized statistically significant associations with birth outcomes. Specifically, birth weight (BW) was associated with PFAS, with effect sizes ranging from −181.209 g (95% confidence interval (CI) = −360.620 to −1.798) per 1 ng/ml increase in perfluoroheptanesulfonate (PFHpS) to −24.252 g (95% CI = −38.574 to −9.930) per 1 ln (ng/ml) increase in perfluorodecaoic acid (PFDA). Similar patterns were observed between other PFAS and birth outcomes: perfluorooctanoic acid (PFOA) and perfluorooctane sulfonate (PFOS) with birth length (BL) and ponderal index (PI), PFOS and perfluorododecanoic acid (PFDoDA) with head circumference (HC), PFHpS with gestational age (GA), and perfluorononanoic acid (PFNA) and PFHpS with preterm birth (PTB). Additionally, PFDA showed a statistically significant association with small for gestational age (SGA). The level of the combined evidence for each exposure-outcome pair was considered to be “moderate”.

**Conclusion:**

This study showed that PFAS exposure was significantly associated with increased risks of various adverse birth outcomes and that different birth outcome indicators had different degrees of sensitivity to PFAS. Further studies are needed to confirm our results by expanding the sample size, clarifying the effects of different types or doses of PFAS and the time of blood collection on birth outcomes, and fully considering the possible confounders.

## Introduction

Per- and polyfluoroalkyl substances (PFAS) have become one of the most widely investigated classes of persistent organohalogen compounds of environmental concern. They have been used for a multitude of industrial and consumer-use products, including fire-extinguishing foams, photographic emulsifiers, furniture, carpets, leather, and paper (1–3). PFAS include a cluster of substances that are classified as persistent organic pollutants due to their high resistance to biodegradation, hydrolysis, photolysis, and atmospheric photo-oxidation (4); the reason for their remarkable stability is because their F-C (fluorine–carbon) covalent bond is stronger than H-C (hydrogen–carbon) bonds (5). These substances are widespread in global ecosystems and even in wildlife in the Arctic, where direct exposure to synthetic chemicals is rare. Therefore, it is unsurprising that, according to biomonitoring data, the general population has had extensive exposure to certain PFAS that are persistent in the environment and capable of bioaccumulation in the food chain (6). Worse, some of these PFAS are transferred through the placenta (7). Thus, PFAS are becoming increasingly significant for the international community in terms of ecological and environmental impacts and human health hazards (4, 8, 9), not only because they are widely used in households but also because the shorter-chain PFAS accumulated in wildlife and humans still have a continuous widespread effect on the environment (10, 11).

The World Health Organization (WHO) estimated that children under 5 years of age bear more than 40% of the burden of environment-related diseases despite accounting for only 10% of the global population (12–14). Air pollution has been extensively studied as a direct cause of child health problems, such as adverse birth outcomes, impaired cognitive and behavioral development (15–17), and respiratory diseases (18, 19). Additionally, environmental exposures, including prenatal exposure to metals, organochlorine pesticides, and other persistent organic pollutants (POPs), have been reported to be associated with adverse birth outcomes (4, 20, 21), including PTB, low birth weight (LBW), SGA, and other birth anthropometric features (BW, HC, and BL). Therefore, clarifying the relationship of environmental exposures, especially exposures to persistent compounds, with birth outcomes is a task of considerable significance to public health (3, 13, 20).

The detrimental effects of PFAS have attracted great attention over the past few years. Many reports have shown that PFAS exposure is associated with cancer risk (22), immune function (23), reproductive function (24), renal function (25), endocrine function (26, 27), and the risk of metabolic diseases (28). The U.S. EPA (Environmental Protection Agency) has reported that PFOA may cause human cancer (15), and some other PFAS are listed under the Stockholm Convention for global regulatory action (29–31). In addition, both animal and human studies have shown that PFOS and PFOA may negatively impact birth outcomes (13, 30, 32). Recent studies have indicated that PFAS may influence human sex hormone biosynthesis, serum levels, and receptors (33–35). However, the specific mechanism involved remains largely unknown. Since 2007, the number of relevant studies per year has steadily increased; most of these studies have focused on the associations of PFOS or PFOA with BW (20, 36–38). Additionally, the number of studies on long-chain PFAS exposure and fetal growth indicators is much less than that of well known short-chain PFAS (e.g., PFOA/PFOS). In addition, some studies (39–41) revealed that differences in fetal sex and gestational order may influence the exposure and birth outcome associations, while others (12, 29, 42–45) discussed the time-course variation in the timing of PFAS collection vs. exposure measurement. Although some epidemiological studies have been conducted to explore the associations, the conclusions of this evidence are quite inconsistent or even opposite (12, 13, 20, 38, 46, 47), even if studies (48, 49) focus on only one outcome indicator (e.g., BW). Such inconsistency may be related to the degree of adjustment for confounding factors, the difference in study design and population characteristics, and the difference in ways of data units and transformations. These inconsistencies make it difficult to interpret the results and then provide specific advice to policy-makers. Therefore, it is necessary to synthesize and evaluate this evidence from multiple sources.

In the massive body of available environmental epidemiological research regarding the effects of PFAS on human health, systematic reviews and meta-analyses (SRMAs) are increasingly used to quantitatively combine data across studies and help translate evidence into policies. However, of massive data available on the effects of PFAS on human health, few studies have investigated the relationships of specific exposure and outcome combinations. To our knowledge, only one SRMA has assessed the effects of PFAS exposure on pregnancy outcomes including miscarriage, PTB, and stillbirth, and four meta-analyses focused on PFOA/PFOS with BW. Specifically, the SRMA (50) on pregnancy outcomes encountered a crucial methodological issue in which estimates from single studies are not comparable and thus make the final estimates difficult to interpret. In the three studies on BW (47, 48, 51), they all concluded that PFOA was significantly associated with BW. Regrettably, Johnson et al. (51) did not distinguish samples originating from plasma vs. serum and did not fully incorporate the available data (only 1 ng/ml increments of PFOA were included). Similarly, Negri et al. (47) and Steenland et al. (48) highlighted differences in the shape of the dose-response curves and presented their data in both forms of analyses (per ng/ml and ln ng/ml). Most recently, one review (52) examined the association of PFAS exposure with the risk of PTB in the United States and suggested that PFAS exposure was a “significantly associated” risk of PTB.

In addition to the aforementioned deficiencies, these four studies also failed to analyze other types of PFAS (particularly, few long-chain PFAS in place of short-chain PFAS have been reported) and more types of birth outcome indicators. Furthermore, only a small fraction of studies noted differences in exposure distribution characteristics (or forms of data transformation) (log-transformed vs. non-log-transformed) and sample collection effects of timing and type, not to mention further meta-analyses for gender and other subgroups. Therefore, there is an increasingly urgent need for systematic reviews and meta-analyses on the relationships between different types of PFAS and different birth outcome indicators in a more rigorous and standardized format as well as for detailed studies of heterogeneity and assessment of the quality of evidence.

In this sense, our study aimed to further clarify the existing inconsistent findings and summarize the latest evidence in a more standardized and detailed format. It is supposed to provide policy-makers and health care providers with a more comprehensive assessment of the association between PFAS and birth outcomes, thereby improving the preventive health of the public, especially for pregnant women.

## Methods

### Search Strategy

Our reporting process is based on the PRISMA Statement Guidelines (53) (Supplementary Table 1). Primarily, following the PECOS (Population, Exposure, Comparator, Outcome, Study) statement, we configured the following as the specific research question: “Among pregnant women exposed to perfluorinated compounds (population), when exposed to or present with one or more perfluorinated compounds in the body (exposure), are they linked with worse birth outcomes (outcome) than those who are assessed to have a low risk of exposure to perfluorinated compounds or who have the lowest level of concentration in body (comparator) evaluated in all cross-sectional, case-control, or cohort studies (study design)?” Studies were identified through a bibliographic search in Web of Science, PubMed, and EMBASE databases. Search terms (perfluoride OR perfluorinated OR perfluoroalkyl OR pfc OR pfcs OR PFASs OR fluoride OR fluorides OR fluorinated OR fluorine OR polyfluorinated OR pfhxa OR pfhpa OR pfoa OR pfna OR pfda OR pfunda OR pfdoda OR pftrda OR pfbs OR PFHxS OR pfos OR mefosaa OR etfosaa OR pfca) and (birth outcomes OR adverse birth outcomes OR birth weight OR low birth weight OR preterm birth OR gestational age OR small for gestational age) were used to identify eligible studies. The search is not limited by languages with the last update on February 13, 2021. Supplementary Table 2 shows the detailed search methods and results. Reference lists of the eligible studies and excluded reviews were searched out and deleted.

### Study Selection

We established the following eligibility criteria according to the predefined PECOS statement: (P): Pregnant women exposed to perfluorinated compounds (living, residing, working or schooling in factories, near polluted water or in polluted environment, etc.); (E): Exposure to or presence of one or more perfluorinated compounds in the body (body fluids such as serum, plasma, etc.) included but not limited to the following categories: “PFC” OR “PFCs” OR “PFASs” OR “PFAS” OR “PFPeA” OR “PFHxA” OR “PFHpA” OR “PFOA” OR “PFNA” OR “PFDA” OR “PFUnDA” OR “PFDoDA” OR “PFTrDA” OR “PFBS” OR “PFHxS” OR “PFOS” OR “MeFOSAA” OR “EtFOSAA”; (C): People who are assessed to be at low exposure risk to perfluorinated compounds or who are in the lowest level of concentration based on body fluid measurements; (O): Birth outcomes determined by clinical assessments from the International Classification of Diseases (ICD), registry data, or medical records, and here our focus is on BW, BW-Z score, BW/GA-Z score, BL, HC and PI, GA, PTB, LBW and SGA. (S): All cross-sectional, case-control, or cohort studies were considered eligible for the review. First, two coauthors (S.Y.G. and Y.N.C.) independently filtered complete titles and abstracts after deleting duplicates. After the initial screening, we downloaded the full text and screened the text again to check whether it could be fully evaluated. All disagreements will be resolved through group discussion.

### Data Extraction and Quality Assessment

Two authors (S.Y.G. and Y.N.C) independently extracted data using a predefined data template. We defined two sets of studies based on our definition of outcomes. Our main analysis focused on the combined association between different types of PFAS and a variety of indicators for the abovementioned birth outcomes. Details of the extracted eligible studies are shown in Supplementary Table 10. Furthermore, if the necessary information could not be extracted from an article or its Supplementary Materials or related publications, the corresponding author or first author of the selected study was contacted. Meanwhile, for documents that miss the necessary information or that fail to provide necessary data in their Supplementary Materials or relevant publications, we performed a separate image data extraction (ORIGIN software) or contacted their corresponding/first author. Then, to assess the quality of each study, we used the correction list used in the study of Andersen, Andreassen, and Krogh (54). The list differentiates high quality from medium and low quality (quality scores from 1.00 to 0.80, 0.79 to 0.60, and <0.60, respectively). Each element has a maximum score of 1.00 and a minimum score of 0.12, and it is then divided into a total score for all elements to calculate a unit-weighted mass score (Supplementary Table 3).

### Risk-of-Bias Assessment

To confirm the conclusions, we assessed the risk of bias (RoB) for each study included in the meta-analysis. RoB assessment describes the quality of research and may indicate systematic errors in results associated with methodological quality assessments (55). For each study, classification was performed across human and animal studies using the NTP/OHAT risk of bias tool (56). In the observational studies, three important factors (detection bias, confounding bias in exposure and outcome assessment) and four other factors (selection bias, consumption/exclusion bias, selective reporting bias, and conflict of interest) were taken into account. A series of questions were used to evaluate the risk for each item as “absolutely low,” “possibly low,” “possibly high,” or “absolutely high.” (see Supplementary Table 4 for detailed questions and Supplementary Table 5 for the rationale for evaluations of each included study). The directed acyclic graph (DAG) (A and B) described by Bach et al. (57) portrays the most reliable causal network connecting exposure (PFAS), outcomes (A illustrating the BW, the BL and HC at birth; B illustrating the PTB and the continuous GA outcome), and potential confounders and mediators for the same research topic. As for the confounder bias domain, this domain was assigned a high RoB if the study considered no significant confounders or only one (parity or maternal age or maternal education) was considered; the domain was classified as probably high RoB if at least two of the following confounders (parity or maternal age or maternal education or infant sex or multiple pregnancy or fetal growth or maternal smoking or GA at birth) were considered; the domain was categorized as possibly low RoB if all of the above confounders and maternal pre-pregnancy BMI were considered; the domain was categorized as definitely low RoB if all of the above confounders and at least one of the following confounders was considered: ethnicity, marital status/single motherhood, obstetric history, air pollution/noise, rural vs. urban residence, and green space in the community of residence. The domain was designated as probably high RoB if no sufficient information was available to determine the RoB. Finally, each study was categorized into an overall tier 1, 2, or 3 RoB. RoB assessment was then re-evaluated by two authors and any discrepancies were discussed to reach a consensus.

### Meta-Analysis

This study was designed to appropriately integrate and analyze the results of individual trials to quantitatively assess the association of exposure to PFAS and birth outcome. We defined 2 exposure-outcome pairs as the threshold for running a meta-analysis, as previous similar studies indicated (58, 59). We considered the study as a cohort (or nested case-control) study if pregnant women were enrolled before the measurement of outcomes (i.e., before delivery) or as a cross-sectional design if women were enrolled at the time of delivery and measured the exposure and outcome concomitantly.

Meta-analyses calculated two types of summary effect estimates: (a) estimate per 1 ng/ml or ln (ng/ml) unit change and (b) estimates at the highest vs. the lowest exposure levels. We performed continuous and categorical exposure meta-analyses because there is no consensus that the association between PFAS exposure and birth outcome is linear or non-linear.

For continuous exposures, we considered their linear regression coefficients (LRC) as effect estimates. Some studies introduced untransformed PFAS levels in the regression models, while others used log-transformed values. We performed two separate meta-analyses of actual and log-transformed values. We chose 1 ng/ml and the natural logarithm of 1 ng/ml (ln) as the units of measurement for LRC for the untransformed and log-transformed analyses, respectively. We recalibrated LRC and standard error in models using different measurement units and used the base change formula for log-transformed PFAS values to change the base to the natural constant e, as described before in the study of Negri et al. 2017, which had a similar research focus as ours. In addition, we used BW/BW-Z/BW/GA-Z (g), HC/BL (cm), GA (weeks), and PI (g/cm^3^) as the uniform outcome indicator units. Detailed transformation methods and estimates of the effects extracted before and after standardization are shown in Supplementary Material B (1–10).

In the highest vs. the lowest exposure meta-analysis, if the included studies provided effect estimates for categorical PFAS exposure, we selected high percentile effect estimates vs. low percentile effect estimates (i.e., the fourth percentile vs. the first percentile exposure or the third percentile vs. the first percentile exposure).

The effect estimates used for evidence synthesis were extracted from the “fully adjusted model” or “main model” among the included studies. If studies used the same population cohort and did not differ in composition and follow-up time, we analyzed only the latest date. Fei et al. (60) reported two different sets of birth outcome indicators separately for PTB, LWB, and SGA in the same Danish national birth cohort, while Fei et al. (43) reported indicators for WB, HC, and placental weight; thus, both studies were included. Similarly, Chen et al. (61), following the same Taiwan Birth Panel Study (TBPS), further collected growth indicators in 2017 from participants aged 9 years to the onset of puberty to explore long-term growth effects; thus, both studies were also retained. Four PFAS (PFOA, PFOS, PFNA, PFUA) were included in the study published in 2012 (62), while only two (PFOA and PFOS) were considered in the later study. Maximally adjusted effect estimates (regression coefficients, ORs, and RRs) were additionally extracted wherever possible. When the effect estimates for the total population are not available, for example, in studies (39–41) that showed the data for males and females separately, we treated them as separately studies as described by Negri et al. (47) and Dzierlenga et al. (49). We also did the same with Lauritzen et al. (63), which included two cohorts (Norway and Sweden). Moreover, Bjerregaard-Olesen et al. (64) and Hjermitslev et al. (29) reported the sum of perfluorocarbons acid (total PFCA), perfluorosulfonic acid (total PFSA), and total PFAS, and Li et al. (65) measured total PFOA, n-PFOA, total PFOS, n-PFOS, Br-PFOS, 1m-PFOS, iso-PFOS an 3+4+5m-PFOS; we disregarded the division of PFAS subgroups and only analyzed data on total PFAS. All analyses were performed and reported separately for each type of PFAS.

Available data for the main analysis or subgroup analysis were extracted from texts or digitized figures (66–68) from the forest plots reported in studies whose raw data are not available. Specifically, we used Origin 2019b software to digitally transform the image data into extractable data, an approach similar to that of Premaratne et al. (69).

To pool effect estimates across the included studies, we performed a meta-analysis of each exposure-outcome pair with a random-effects or fixed-effects model based on heterogeneity (70). Most studies reported regression coefficients (β) or ORs, with only one reporting RRs (71); therefore, we removed that study from our meta-analysis. In addition, to compare effect estimates across studies, we normalized all effect estimates in units to 1 ng/ml or 1 ln (ng/ml) difference in PFAS by the following equation formula: OR_standardized_ = e∧(ln (OR_origin_)/Increment_origin_ × Increment_standardized_). Meanwhile, we assessed heterogeneity using the I^2^ statistic (greater than 50% is considered to indicate substantial heterogeneity) (Higgins et al. 2003) and the *p*-value of the chi-squared test for heterogeneity (less than 0.1 indicates statistical significance) (72). Additionally, univariate meta-regression for gender, timing category of blood sample collection, study design, geographic region, blood collection matrix, outcome assessment and adjustment of GA, and parity for birth outcome were conducted to identify sources of heterogeneity that included more than 10 studies (73). Moreover, we assessed publication bias using Egger's test (72). We further assessed publication bias using Doi plots and the Luis Furuya-Kanamori (LFK) index (< |1|, “no asymmetry”; between |1| and |2|, “slight asymmetry”; > |2|, “severe asymmetry”), taking into account the inaccuracy of statistical and graphical methods in assessing publication bias in a few studies (74). Additionally, a sensitivity analysis was conducted to assess stability of the results, specifically by recalculating the pooled effect estimates by excluding individual studies in turn.

All analyses were conducted with Stata version 14 (StataCorp LP, College Station, TX, USA) and MetaXL v.5.3 software (EpiGear International Pty Ltd, Sunrise Beach, Queensland, Australia, www.epigear.com). Studies that failed to qualify for the meta-analysis but investigated relevant pairs of associations were analyzed by descriptive summary. Additional subgroup analyses were conducted to further assess whether the pooled effect estimates remained robust across gender, timing category of blood sample collection, study design, geographic region, blood collection matrix, outcome assessment, and adjustment of GA and parity.

### Confidence Ratings of the Body of Evidence and Translation of Evidence Levels

According to the NTP/OHAT framework, we assessed the body of evidence using the Grading of Recommendations Assessment, Development, and Evaluation (GRADE) guideline (75). The NTP/OHAT framework was used to assess the quality and strength of the evidence because it was consistent with the causality criteria of Hill. In brief, observational studies exhibit low to moderate initial confidence ratings for the lack of control in assigning exposures. The initial confidence of the evidence body was determined by the key study design features. Then, we evaluated each exposure-outcome pair, with either five factors lowering initial confidence or four factors increasing initial confidence. The final confidence of each exposure-outcome pair was divided into four descriptors: “high,” “moderate,” “low,” or “very low.” Reliability is translated into “high,” “moderate,” “low,” “inadequate,” or “evidence of no effect on health” levels of epidemiological evidence, depending on the confidence of different exposure-outcome pairs.

## Results

### Study Selection and Characteristics

**Figure 1:**
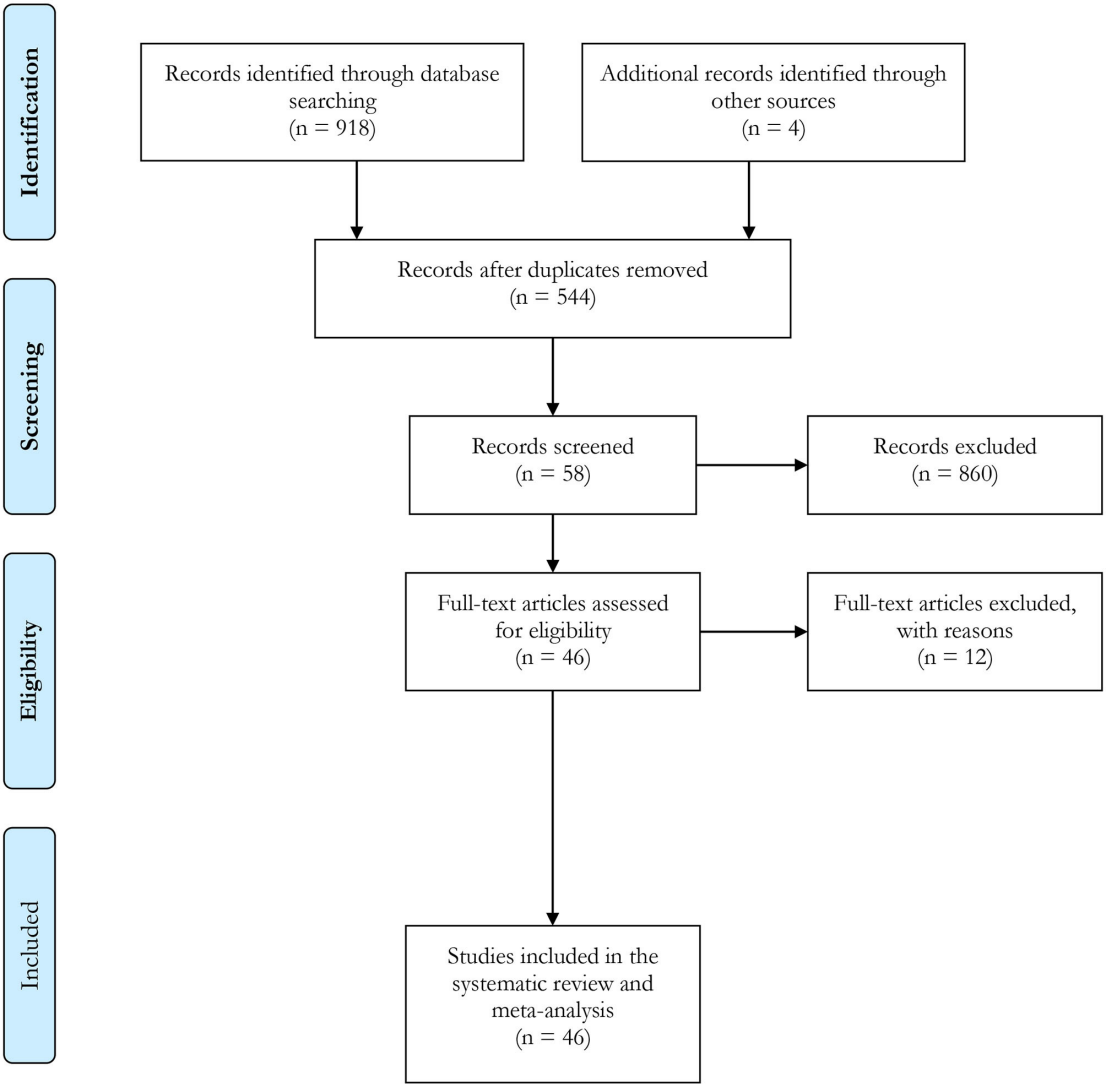
Flow diagram of study selection process.

After excluding duplicates, we retained a total of 918 papers for title/abstract screening. As shown in Figure 1, we conducted a full-text search of 58 relevant articles, of which 46 provided available data on PFAS and birth outcomes that could be pooled for meta-analysis as well as evaluation (12–14, 20, 29, 36–39, 41, 43–46, 57, 60–68, 71, 76–95). Among them, 2 publications (96, 97) examined the association between PFAS and reproductive distance, while 3 articles (98–100) focused on the association between PFAS exposure in the environment and birth outcomes. These five studies were separately synthesized again in a descriptive manner.

The characteristics of the included studies are summarized in Supplementary Table 3. Years of publication ranged from 2007 to 2021, with 13 new articles after Steenland et al. (48) published the previous meta-analysis (PFOA and BW). Two different studies from Norway and Sweden were described in one article (63), and studies (39–41) only presented separate data for gender subgroups and did not aggregate and summarize data for the population at large, resulting in the smaller volume of included documents than the studies. Of the included studies, 16 were from Asia, 15 were from Europe, 10 were from North America, and 3 were from Oceania. In particular, the population of Lenters et al. (45) consisted of people from Greenland, Poland, and Ukraine. In addition, 37 studies were longitudinal, including 33 cohort studies and 4 case-control studies, while the remaining 9 were cross-sectional studies. The included study subjects ranged from 85 to 11,737. The vast majority of study subjects had outcome measures at birth (i.e., early fetal life), but Chen et al. (61) and Cao et al. (36) extended those measures to 24 weeks postpartum to better explore the long-term effects of PFAS exposure. Notably, 16 studies focused on sex differences, while Marks et al. (37) included only male infants and Maisonet et al. (44) included only female infants. Eight studies investigated differences in the timing of blood sample collection. Twenty-nine studies had blood samples from serum, 14 studies had blood samples from plasma, and the remaining 4 studies had blood samples from whole blood collected at 0 (preconception) to 40 weeks (cord blood at term). For exposure assessment, 43 studies of PFOA and 40 studies of PFOS were included. Furthermore, for continuous data, 41 studies examined BW indicator, 21 studies examined BL, 16 studies examined HC, and 13 studies examined each of GA and PI; for categorical data, 11 studies examined PTB, 6 studies examined LBW, and 7 studies examined SGA. Forty studies assessed the corresponding birth indicators by clinical examination, and 3 studies were self-reported by questionnaires. Of interest, 28 studies adjusted for GA as an independent confounder, and 30 studies adjusted for parity.

### Study Quality and Risk-of-Bias Assessment

The studies included in our meta-analysis could be considered moderate to high quality, with scores between 0.77 and 0.94 based on quality assessment (Supplementary Table 3). Supplementary Table 11 shows the results of the RoB assessment of the meta-analysis studies using heat maps. We had predetermined criteria for the assessment of quality exposure from body fluids, with 7 studies classified as “definitely low” and all the remaining included studies (*n* = 39) rated as “definitely low” for RoB. Regarding the RoB of outcome assessment, 11 studies were classified as “definitely low,” 1 study as “probably high” (101) and 1 as “definitely high” (82) because of their deficiencies in medical record validation. For confounding bias in the included studies, 6 were rated as “definitely low” RoB, 14 as “probably low” RoB, 20 as “probably high” RoB, and 6 “definitely high” RoB because they ignored the main confounding factor GA and parity and adjusted for too few confounders. Moreover, given the inherent characteristics of cross-sectional vs. longitudinal studies and the way control groups were matched, 17 studies were rated as “definitely low” or “probably low” for selectivity bias, while 4 studies were rated as “probably high” and 8 studies were rated as “definitely high,” respectively. Nevertheless, for attrition/exclusion bias, four studies were rated as “definitely low” for the RoB. However, the study of Maisonet et al. (44) was rated as “probably high” because the authors stated to observe changes in the magnitude and direction of the bivariate associations arising after excluding some of the data, and other studies without explicit exclusion bias were rated as “probably low.” Additionally, for selective reporting bias, one study (81) was rated as “definitely low” for RoB because the results included in the meta-analysis provided sufficient detail for reporting, 3 studies were rated as “probably low” because of indirect reporting of results, and the rest studies were rated as “probably low” RoB. The authors of most studies declared no conflicts of interest, and four studies that did not report conflicts of interest or financial support were rated as “probably high.”

In summary, all eligible studies were categorized as either tier 2 (*n* = 17) or tier 1 (*n* = 29) of the RoB, indicating that there may be some reasonable bias that casts some doubt on the results.

### Data Synthesis

#### Meta-Estimates for Associations of PFAS With BW (g)

The summary regression coefficient for the association of per 1 ln (ng/ml) increase in exposure to any type of PFAS was −0.110 (95% CI: −0.246, 0.026, *p*-value < 0.001), and for per 1 ng/ml was −0.016 (95% CI: −0.029, −0.002, *p*-value = 0.159), while the highest vs. the lowest level was −0.106 (95% CI: −0.221, 0.008, *p*-value < 0.001) using random-effects models (Supplementary Table 12). The overall I^2^ was 18.3% to 56.7%, indicating low to moderate between-study heterogeneity.

**Figure 2:**
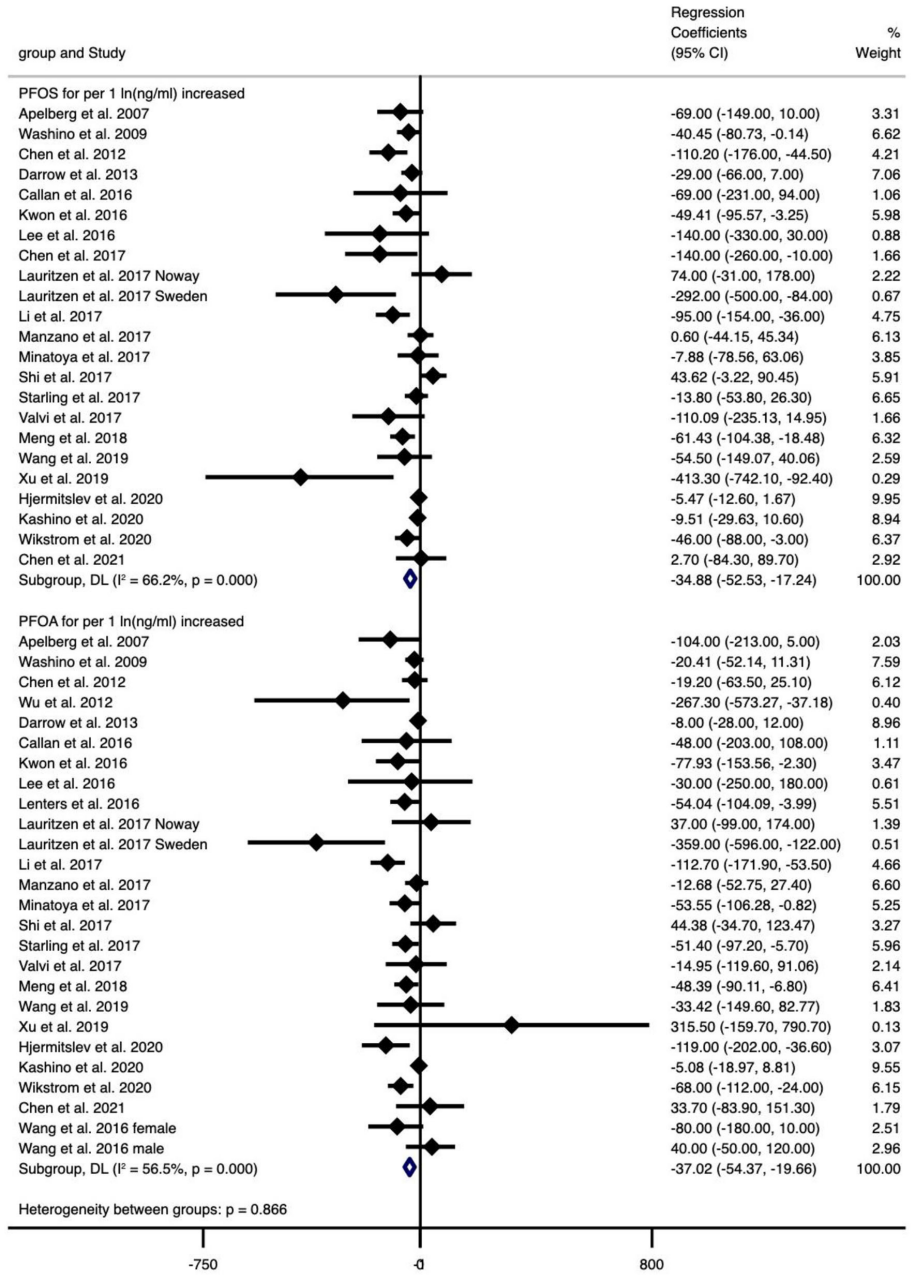
Meta-analysis of the associations of PFOS and PFOA with birth weight (g) for per 1 ln(ng/ml) increment of exposure.

**Figure 3:**
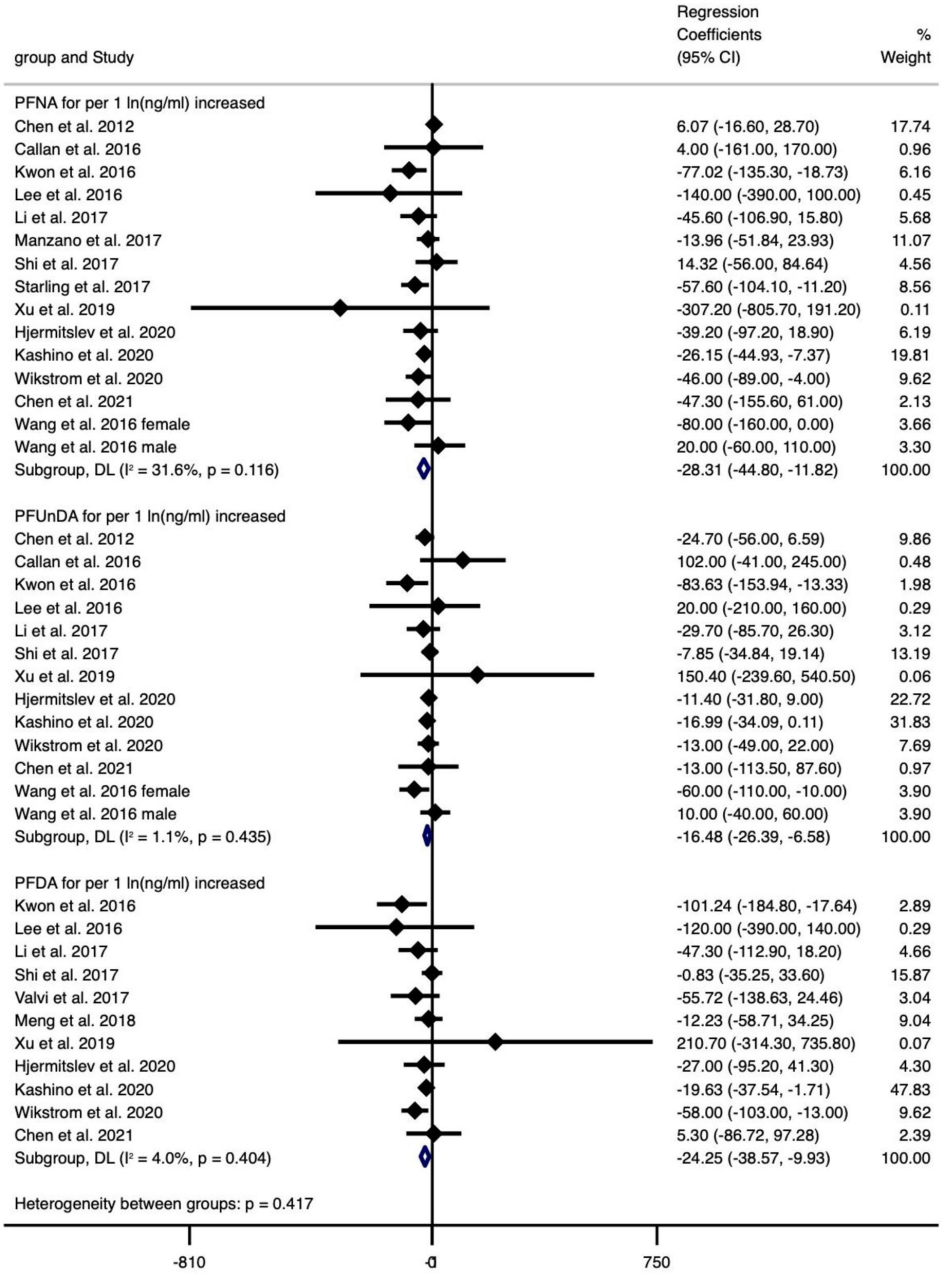
Meta-analysis of the associations of PFNS, PFUnDA and PFDA with birth weight (g) for per 1 ln(ng/ml) increment of exposure.

**Figure 4:**
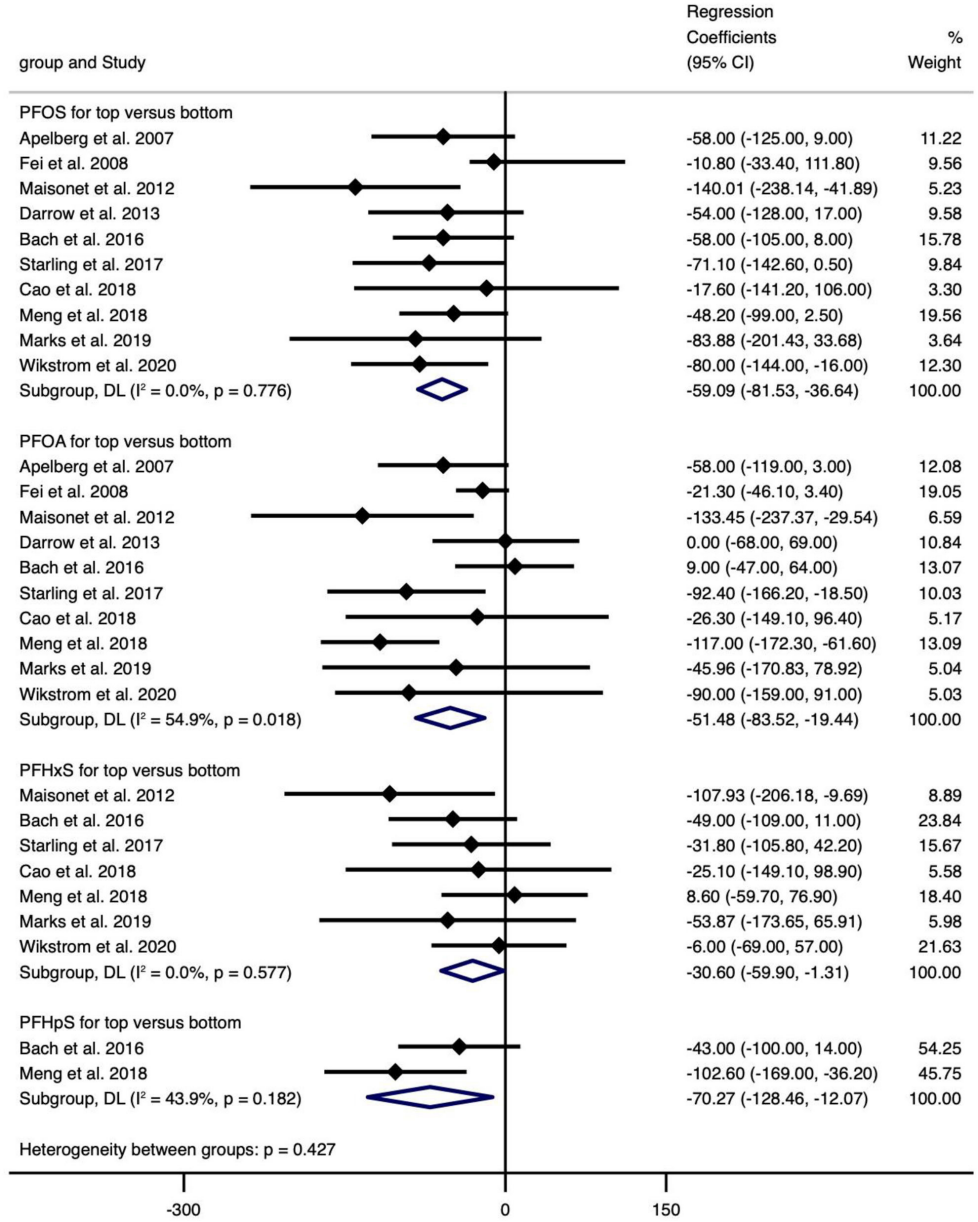
Meta-analysis of the association of PFOS, PFOA, PFHxS and PFHpS with birth weight (g) for top versus bottom categories of exposure.

The type of PFAS demonstrated different effects on BW and vice versa (Figures 2–4). We found that PFOS (−0.002 per 1 ng/ml increase), PFHpS (−181.209 per 1 ng/ml increase and −70.267 for the highest vs. the lowest level exposure), and PFDA (−24.252 per 1 ln (ng/ml) increase) reduced BW (all types), like most PFAS, although others still had some regression coefficients with 95% CIs covering 0. In turn, the type of BW also showed a differential sensitivity to PFAS, for unstandardized BW, there exhibited statistically significant reductions in the case of the following PFAS: PFOS [−34.882 per 1 ln (ng/ml) increase and −59.085 for the highest vs. the lowest level exposure], PFOA [−37.017 per 1 ln (ng/ml) increase and −51.482 for the highest vs. the lowest level exposure], PFHxS (−30.604 for the highest vs. the lowest level exposure), PFNA [−28.312 per 1 ln (ng/ml) increase], PFUnDA [−16.485 per 1 ln (ng/ml) increase], PFHpS (−181.209 per 1 ng/ml increase and −70.267 for the highest vs. the lowest level exposure) and PFDA [−24.252 per 1 ln (ng/ml) increase]. In contrast, for standardized (GA adjusted) BW, only PFOS [−0.002 per 1 ln (ng/ml) increase and −0.133 per 1 ln (ng/ml) increase] and PFNA (−0.1 per 1 ng/ml increase, *n* = 1) showed statistically significant reductions. Overall, the heterogeneity of studies related to BW and PFAS was low, even after we classified BW by “standardized” vs. “non-standardized” and by continuous or categorical data (Supplementary Table 12). Specifically, PFOS per 1 ln (ng/ml) increase in crude BW had the highest heterogeneity (66.2%), while most of the meta-analyses showed non-significant heterogeneity. Pooled regression coefficient values ranged from −60.550 (95% CI: −158.964, 37.865) for PFUnDA (*n* = 2) to −93.076 (95% CI: −257.910, 71.760) for PFHpS exposure (*n* = 2) when considering per 1 ln (ng/ml) increase; and from −0.002 (95% CI: −0.004, 0) for PFOS (*n* = 10) to 22.831 (95% CI: −52.297, 97.960) for PFDA exposure (*n* = 2) when considering per 1 ng/ml increase; and from −70.267 (95% CI: −128.462, −12.071) for PFHpS (*n* = 2) to 31.000 for PFHpA exposure (*n* = 1, 95% CI: −31.000, 93.000) when considering the highest vs. the lowest dose exposure (Supplementary Table 12).

Overall, exposure to PFOS, PFHpS, PFDA decreased BW, exposure to PFOS, PFOA, PFHxS, PFNA, PFUnDA, PFHpS, PFDA decreased BW (without GA adjustment), and exposure to PFOS, PFNA decreased BW-Z score, while the other PFAS did not show statistically significant effects (Supplementary Table 12).

#### Meta-Estimates for Associations of PFAS With BL (cm), HC (cm), and PI (g/cm^3^)

**Figure 5:**
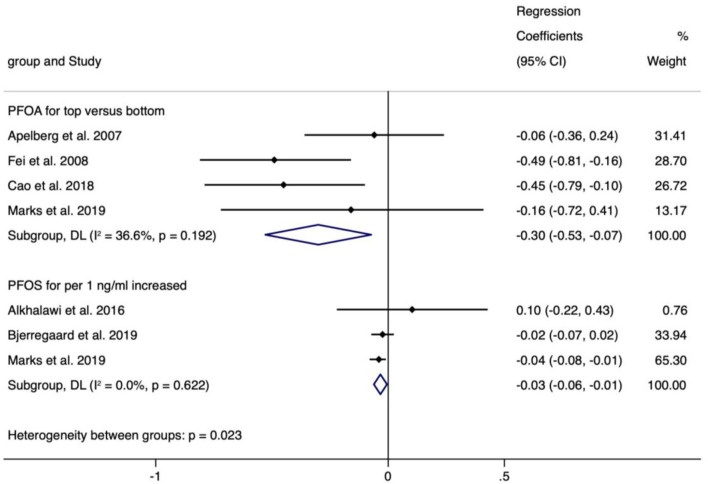
Meta-analysis of the association of PFOS and PFOA with birth length (cm).

The meta-analyses indicated that exposure to PFOS (*n* = 3, −0.034, 95% CI = −0.062 to −0.005, per 1 ng/ml increase) and PFOA (*n* = 4, −0.301, 95% CI = −0.529 to −0.073, the highest vs. the lowest level exposure) showed statistically significant decrease in BL (Figure 5), and the corresponding meta-analyses were of low heterogeneity (I^2^ = 0.0% and 36.6%), with regression coefficients varied from −0.301 (PFOA, *n* = 3) to 0.420 (PFHpA, *n* = 1), which was similar to the case of continuous or categorical exposure (Supplementary Table 12).

**Figure 6:**
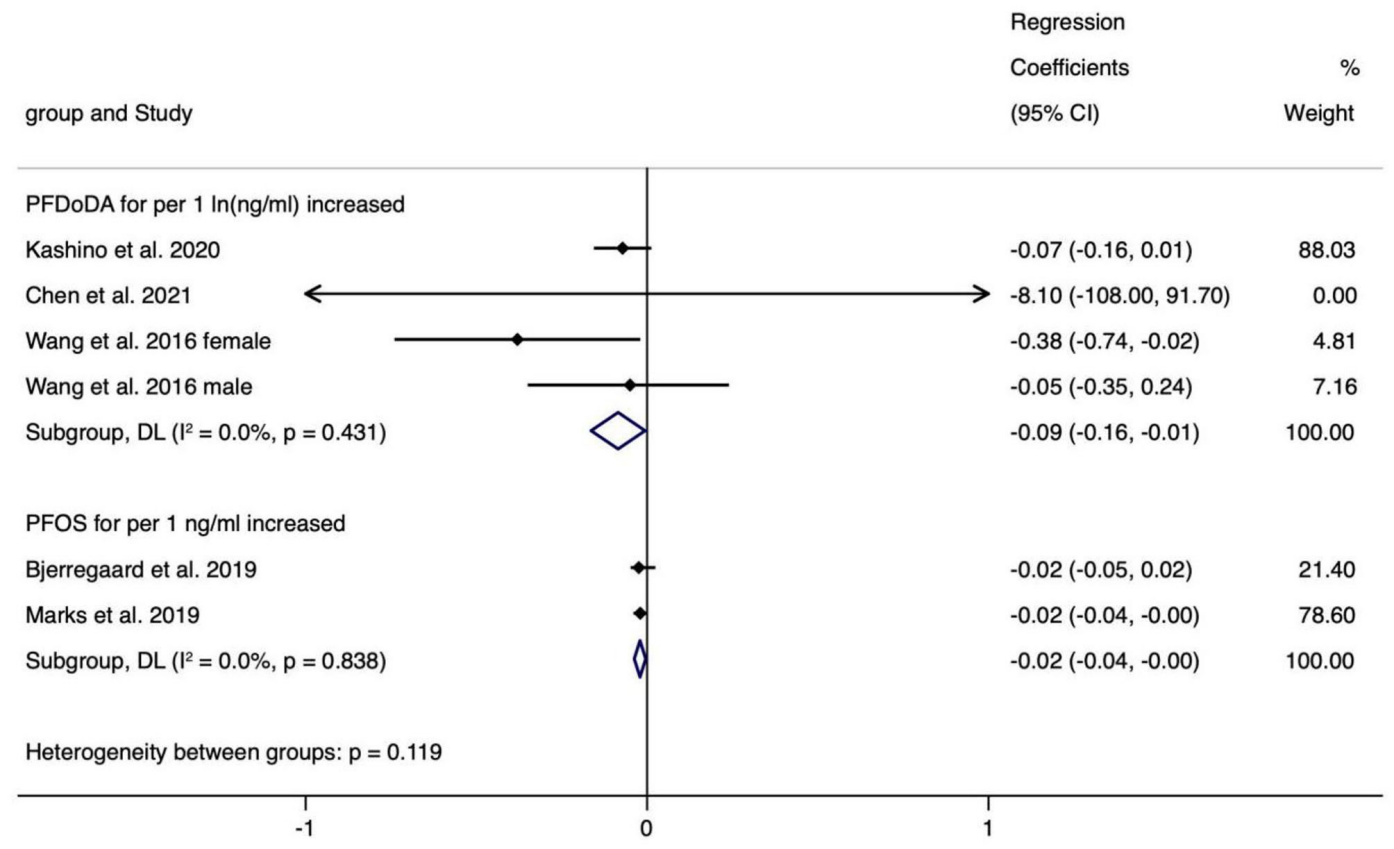
Meta-analysis of the association of PFDoDA and PFOS with head circumference (cm).

The meta-analyses indicated that exposure to PFOS (*n* = 2, −0.021, 95% CI = −0.038 to −0.004 per 1 ng/ml increase) and PFDoDA (*n* = 4, −0.085, 95% CI = −0.164 to −0.006 per 1 ln (ng/ml) increase) showed statistically significant decrease in HC and with non-significant heterogeneity (I^2^ = 0.0% and 0.0%), which were similar to the results of BL (Figure 6). The regression coefficients varied from −36.900 (PFHpA, *n* = 1) to 0.066 (PFOA, *n* = 2) (Supplementary Table 12).

The meta-analyses indicated that only exposure to PFOS (*n* = 2, −0.355, 95% CI = −0.702 to −0.008 per 1 ng/ml increase) and PFOA (*n* = 3, −0.412, 95% CI = −0.787 to −0.037 per 1 ng/ml increase) showed statistically significant decrease in PI and no heterogeneity (I^2^ = 0.0% and 0.0%), with regression coefficients varied from −0.412 (PFOA, *n* = 2) to 0.100 (PFNA, *n* = 1 and PFDA, *n* = 1) (Supplementary Table 12).

Overall, exposure to PFOS, PFOA, PFDoDA decreased BL, exposure to PFOS, PFDoDA decreased HC, exposure to PFOS, PFOA decreased PI, while the other PFAS did not show statistically significant effects (Supplementary Table 12).

#### Meta-Estimates for Associations of PFAS With GA (Weeks)

**Figure 7:**
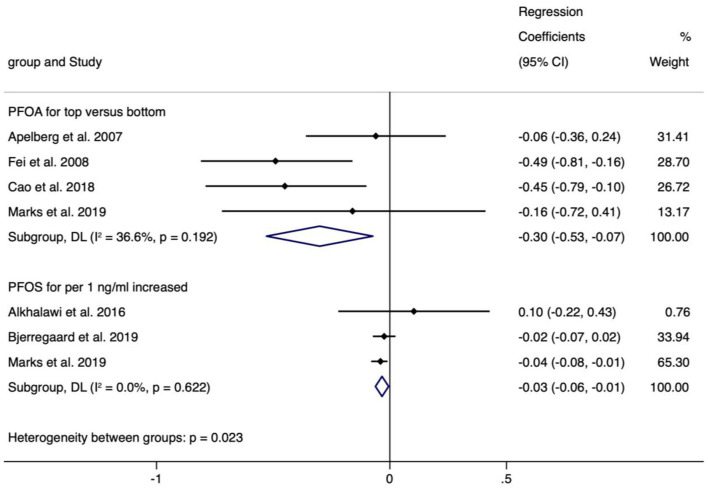
Meta-analysis of the association of PFOS and PFOA with ponderal index (g/cm^3^).

The meta-analyses indicated that only PFHpS (−0.232, 95% CI = −0.367 to −0.097, per 1 ln (ng/ml) increase and −2.000, 95% CI = −3.300 to −0.700, for the highest vs. the lowest level exposure) showed statistically significant decrease in GA, and there was no heterogeneity (I^2^ = 0.0%) (Figure 7), with regression coefficients ranging from −2.000 (PFHpS, n=1, for the highest vs. the lowest level exposure) to 0.180 (PFOSA, n=1, for the highest vs. the lowest level exposure). Overall, exposure to PFHpS reduced GA, while the other PFAS did not show statistically significant effects (Supplementary Table 12).

#### Meta-Estimates for Associations of PFAS With PTB, LBW, and SGA

For PTB, exposure to PFOA (13.3% for categorical exposure), PFOS [23.7% per 1 ln (ng/ml) increase, 0.6% per 1 ng/ml increase and 39.4% for categorical exposure], PFHxS [1.7% per 1 ln (ng/ml) increase and 17.8% for categorical exposure], PFNA (57.7% per 1 ng/ml increase and 75.3% for categorical exposure), PFHpS [73.5% per 1 ln (ng/ml) increase and 80% for categorical analysis] and PFOSA (60% for categorical exposure) were associated with non-significant increased risk (Supplementary Table 12).

For LBW, exposure to PFOS [21.3% per 1 ln (ng/ml) increase and 35.2% for categorical exposure], PFOA (2.3% for categorical exposure), PFHxS [3.4% per 1 ln (ng/ml) increase] or PFNA (50.0% for categorical exposure), PFUnDA [1.0% per 1 ln (ng/ml) increase] and PFDA [28.1% per 1 ln (ng/ml) increase] were also were associated with non-significant increased risk (Supplementary Table 12).

For SGA, exposure to PFOS [21.4% per 1 ln (ng/ml) increase], PFOA [21.6% per 1 ln (ng/ml) increase and 16.8% for categorical exposure], PFHxS (4% for categorical exposure) or PFNA [7.4% per 1 ln (ng/ml) increase and 23% for categorical exposure], PFUnDA [10.4% per 1 ln (ng/ml) increase and 52% for categorical exposure] and PFDA [46% per 1 ln (ng/ml) increase and 50% for categorical exposure], PFDoDA [13.8% for per 1 ln (ng/ml) increase], PFHpA [6% per 1 ln (ng/ml) increase and 25% for categorical exposure] and PFDeA [42% for per 1 ln (ng/ml) increase] were associated with increased risk; however, most of them are non-significant (Supplementary Table 12).

Overall, exposure to PFNA, PFHpS increased the risk of PTB, exposure to PFDA increased the risk of SGA, while the other PFAS did not show statistically significant effects (Supplementary Table 12).

#### Subgroup Analysis

Supplementary Table 13 shows the results of subgroup analyses by fetal gender, time of blood sample collection, blood sample type and whether adjusted for GA/parity, study design, and geographic region.

Subgroup analyses for gender suggested that the associations of PFAS with birth outcome may modify by gender; for example, girls in PFOS/PFOA–BW showed a stronger variation in the effect estimates than boys for the highest vs. the lowest level exposure. However, continuous exposure did not show significant differences by gender (Supplementary Tables 10, 13). Meanwhile, regarding time of blood sample collection, similar details were shown in Supplementary Tables 10, 13. Furthermore, when subgroup analyses were performed by study design (Supplementary Tables 10, 13), effect estimates were influenced by it for some of the exposure-outcome pairs. Additionally, in the combination of PFOS with BW, effect estimates were different for studies performed in Asia (*n* = 12, −44.792, −76.694 to −12.889), Europe (*n* = 7, −49.645, −88.805 to −10.486) and North America (*n* = 5, −0.002, −0.004 to 0). Results for the subgroup analyses by blood sample type and whether adjusted for GA/parity also indicated that these factors may modify the associations of some exposure and outcome combinations.

#### Small-Scale Studies on Environmental Exposure to PFAS

In addition to our meta-analysis, 3 similar papers also examined the effects of PFAS on birth outcomes among populations with known exposure to high level PFAS in contaminated environments. Nolan et al. (98) investigated the associations in Little Hocking Water Association (LHWA) cohort in Washington County, Ohio, where serum PFOA levels were ~80 times higher than those in the general US population. This cross-sectional study included 1,555 singleton newborns and suggested that exposure to high level of PFOA was associated with neonatal birth weight, PTB and gestational age. Similarly, Manea et al. (100) published a study in 2019 that was performed from 2003 to 2018 including 105,114 singleton live births, which focused on the risk of severe SGA in PFAS-contaminated areas. The results showed that exposure to PFAS-contaminated areas is significantly associated with an increased risk of SGA.

#### The Effects of PFAS on the Anogenital Distance (AGD) of New-Born

Our systematic review found two additional cohort studies from Canada and Denmark examining the relationship between prenatal exposure to PFAS and AGD. Lind et al. (96) examined the association between levels of PFOS, PFOA, PFHxS, PFNA, and PFDA and AGD in the Odense Children's Cohort, which showed a sexually dimorphic effect of PFOS on AGD. Arbuckle et al. (97) assessed the association between AGD in 401 newborns and PFOA, PFOS and PFHxS in maternal plasma in early pregnancy in the MIREC cohort, which showed that for a 1 ln (ng/ml) increase in PFOA exposure, AGD increased by 1.36 mm (95% CI: 0.30, 2.41), but the statistical significance disappeared in either categorical exposure analysis or gender subgroups.

#### Other Types of Birth Outcome Indicators

We identified additional 11 studies assessing the associations of miscarriage and 1 study assessing stillbirth with PFAS exposure. Savitz et al. (101) did not find a significant association between estimated PFOA exposure and stillbirth after they examined multiple birth outcome indicators in pregnant women in areas with high exposure to PFOA-contaminated water (located in West Virginia and Ohio). In terms of miscarriage, Two studies (81, 102) performed by Darrow et al. were considered as duplicates, and only Darrow et al. (102) was included because both studies were based on the same cohort and no updates were available. Similar studies (36, 80, 94) involved only descriptive analysis and failed to provide extractable data. Louis et al. (103) examining the relationship between PFAS and pregnancy loss, they did not give an explicit indicator of miscarriage (their definition of “pregnancy loss” was significantly different from miscarriage). Stein et al. (104) found that exposure to neither PFOA nor PFOS showed an association with miscarriage for the populations live in the Central Ohio Valley region. We standardized the data of Darrow et al. (102) and Jensen et al. (105), which produced the ln-transformed data and for Liew et al. (106), doubling data were provided, and the specific transformation method was described above. We performed a meta-analysis on the remaining 3 studies and found that with exception of PFDA (OR = 2.293, 95% CI: 1.252, 4.201, for the highest vs. the lowest level exposure), others (PFOA, PFOS, PFHxS, PFNA, PFHpS, PFOSA) were statistically significantly associated with miscarriage. However, considering that half of the tested exposure-outcome pairs included fewer than 3 studies, further expansion of the sample size is needed to determine the association between PFAS and miscarriage.

#### Sensitivity Analysis and Publication Bias

The results of sensitivity analyses are presented in Supplementary Tables 6–8. Most of the results did not substantially change, which indicated the stability of our results. Meta-regression analyses for the exposure-outcome pairs containing at least 10 observations per covariate (i.e., gender for PFOS/PFOA/PFHxS/PFNA-BW, time of sample collection for PFOS/PFOA/PFHxS/PFNA/PFDA-BW, and other covariates for PFOS/PFOA-BW/HC/BL and PFOA-GA) indicated that heterogeneity may derive from adjustment of parity for PFOA-GA (*P* = 0.039), study design for PFOA-HC/BW (*P* = 0.020 and *p* = 0.040), adjustment of GA for PFOA/PFNA-BW (*P* = 0.047 and *p* = 0.031). It was also noteworthy that when meta-analyses were conducted under the quality effect models (Supplementary Figures 1–65), significant differences were found only in the combinations of PFOA/PFOS/PFDA-BW, PFDoDA-BL and PFHpS-GA for per 1 ln (ng/ml) increment in exposure, PFOA-PI and PFOS-BL for per 1 ln (ng/ml) increment in exposure, PFOA-BL/HC, PFOS-BW and PFNA-PTB for the highest vs. the lowest categories of exposure. Moreover, we detected publication bias in some of the exposure-outcome pairs, as shown in Supplementary Table 12. However, among the 75 exposure-outcome pairs (*n* ≥ 3), Doi plots and the LFK index suggested publication bias (major asymmetry) in 61 of the 75 pairs (Supplementary Figures 34–65).

### Confidence Rating and Level of Evidence Translation

Supplementary Table 9 shows the results of our summary on confidence ratings for each exposure-outcome pair in the body of evidence. The NTP/OHAT framework stipulates that only controlled and experimental studies should initially be rated as “high confidence,” so all included observational studies were rated as “moderate confidence” throughout our rating process. Most of the studied pairs with significant associations were assessed as moderate confidence, and thus were translated to “moderate level” of epidemiological evidence.

## Discussion

To the best of our knowledge, the present study is the largest and most comprehensive systematic review and meta-analysis combining all PFAS exposure types available with all birth outcomes available. It has been well documented that newborn can be prenatally exposed to a wide variety of PFAS through placental transfer. Therefore, over the past decades, an emerging number of studies on association of birth outcomes with PFAS have been performed as newborns are more susceptible to the toxicity from environmental pollutants than adults.

Our study suggests that the currently studies are varied in PFAS type, birth outcome indicators, timing of blood sampling, data analysis methods, and covariate adjustment. Our findings may support the conclusion that certain types of PFAS are increasingly proven to be could partly reduce physical measures such as BW, BL and HC, reduce GA, and increase the incidence of adverse birth outcomes such as PTB, LBW and SGA. With exception of LBW, at least one of the remaining pairs mentioned above showed statistical significance. However, further analysis and interpretation of the results were hindered by the limited number of studies. Meta-analysis and subgroup analysis indicated that the associations between different types of PFAS and different birth outcome indicators were not identical, and there was no consistent strength of evidence to support them. Additionally, the sensitivity of the indicators and differences in gender, time of blood sampling, and the shape of the dose-response curve led to subtle differences in the conclusions (Supplementary Table 13). We did not observe substantial and statistically significant heterogeneity in the results of most meta-analyses (Supplementary Table 12), as suggested by Negri et al. (47) and Steenland et al. (48). In the meta-analysis, heterogeneity may arise from the types of PFAS studied, sample size and statistical analysis methods, study designs, or geographic regions.

Regional socioeconomic status (SES) or potential sociodemographic predictors have emerged as potentially key confounders in environmental health studies. Therefore, the heterogeneity of the present study may be partly explained by SES as 11 studies we included were all from Asia, representing a lower economic level with a higher probability of exposure to particular types of PFAS for the majority of the study population. The between-study heterogeneity may also arise from gender, time of blood collection and source of blood samples. These results have been validated in our subgroup analyses, but these estimates need to be interpreted with caution, given the inherent limitations of epidemiological data. In particular, the results of cross-sectional studies did not show temporal associations; thus, the possibility of reverse causality cannot be ruled out. Wang et al. (40) found a reduction in birth size and GA for girls and boys when exposed to PFNA and PFDoDA, while several studies (66, 67, 85) found that a reduction in BW with PFOA exposure was presented only in male rather than female fetuses. Interestingly, Manzano-Salgado et al. (66) and Fei et al. (43) found a reverse association on the combination of PFOS with BW, which suggesting that there is indeed a modify effect of gender in associations between different types of PFAS and birth outcomes. With regards to the timing of blood sampling, its effect on birth outcomes has been widely noted for a long time. Meanwhile, the differences in PFAS concentrations between different stages of labor and in cord blood (at term) have been greatly studied. For example, Dzierlenga et al. (49) illustrated that PFOA levels vary by gestational length and decrease during pregnancy, possibly due to pregnancy-related blood volume expansion or parallel increase in glomerular filtration rate. Other studies (48, 79) have further indicated that there is an undetermined reverse causality in the blood during pregnancy, which can affect the feasibility of the conclusions.

Different birth outcome indicators may represent different pathogenic mechanisms. BW and BL have traditionally been proposed as the two main indicators that could reflect short-term and long-term fetal growth and development (especially nutritional status), respectively, representing the fetal development in utero. The BW-Z score, BW/GA-Z score or WAZ (age-specific Z-scores for weight) is a measure of BW that standardized to total body mass using US reference data for GA, adjusted for the effect of GA on fetal growth and development to some extent. Similar indicators are weight-for-age, length-for-age, weight-for-length, and body mass index (BMI) Z-scores. The PI is measured by birth weight (g)/(birth length (cm)^3^) ^*^ 100 and is used as a proxy for body weight that takes into account the effect of body length on body mass. It was first introduced in the early 1920s and is widely used to assess the growth and development of newborns and young children, similar to the BMI of adults (35, 93). In particular, BW values are easier to obtain than PI or BW-Z score values because only one measurement is required. This may partly explain the inconsistencies in our subgroup analysis of BW (crude weight, weight-Z index, PI) and the contradictory results of previous study (91). HC is closely related to brain and cranial growth as well as biparental growth and can reflect brain development or other brain disorders (e.g., hydrocephalus) and neurological completeness (37, 64). The timing effects of different PFAS on fetal development was significant because several indicators were closely related to time periods: the increasing of number and volume of fetal brain cells and the formation of cortical gyri are developed during the first trimester of pregnancy while the proliferation of brain cells occurs at 20 weeks of gestation and the active growth of brain cells lasts from 30 weeks of gestation to delivery. It is also worth noted that GA, as an intermediate variable for other birth outcome indicators (e.g., BW), is associated with BW and cumulative PFAS exposure. Some studies indicated that adjusting for GA would result in a positive association of PFOA with BW. However, adjustment of GA does not affect the relationship between PFOA and BW, which is also supported by the present study, although more than half of the included studies suggest that GA is unlikely to be a confounding or intermediate variable (62, 77). In any case, GA should be given adequate attention and treated appropriately as an important variable in future studies. In addition, age and gender differences in the measurement of birth and developmental indicators are also of great concern (40, 41).

Our results revealed that different types of PFAS result in differences in the sensitivity of different outcome indicators for PFAS with different molecular weights and backbone lengths. Specifically, BW was observed to appear to be significantly reduced when exposed to PFOS (per 1 ng/ml: −34.882), PFNA (per 1 ng/ml: −28.312), PFUnDA (per 1 ng/ml: −16.485), PFDA (per 1 ng/ml: −24.252), PFOA (highest vs. lowest: −51.482), PFHxS (highest vs. lowest: −30.604), PFHpS (highest vs. lowest: −70.267). By comparison, BL had the opposite significant effects when exposed to PFOS (per 1 ln (ng/ml): −0.034), PFDoDA (per 1 ln (ng/ml): −0.148), and PFUnDA (highest vs. lowest: 0.410). Meanwhile, PTB showed statistically significant differences in exposure to PFNA (57.7%) and PFHpS (73.5%). Similar results for other birth outcome indicators corresponding to one or more specific types of PFAS are described in Supplementary Table 13. These findings have been widely reported in previous studies (38, 100). Callan et al. (83) even found that PFHxS and PFUnDA seem to have completely opposite effects on the increase of optimal BW ratio.

Our subgroup analyses demonstrated the modify effect of gender in the associations of birth outcome indicators with prenatal PFAS exposure, such as, in PFOS and BW combination [male: −45.073 (−104.248, 14.102) and female: −111.402 (−180.314, −42.490)] (Supplementary Table 13), which was also found by previous studies (13, 40, 41, 68). One of these studies (40) found that prenatal exposure to PFDoDA was associated with reduced HC in girls but not in boys. Wikstrom et al. (13) and Chen et al. (12) shared a similar finding that PFOS had a greater negative effect on weight in girls than in boys, and similar findings were also observed in other studies (40, 77, 107). Furthermore, the modify effect of gender seems to be more pronounced in an inverse relationship between long-chain PFAS exposure and height (61) as well as weight later in life (40). The potential mechanism involved may be related to the effect of PFAS on human sex hormone biosynthesis (34, 39), insulin-like growth factor-1 serum levels (35) and estrogen receptor function (33), even though some *in vitro* tests remain questionable (108).

It should be noted that maternal blood samples were collected at different times during gestation, which was presumed to have significant differential effects in our subgroup analysis (Supplementary Tables 10, 13). Chen et al. (12) found that indicators of birth somatic measurements mainly appeared in the first trimester by analyzing single and multiple time points, further suggesting that primary ossification centers, affected hormones and high PFAS levels resulted in a “sensitive window” in the first trimester that affects fetal skeletal growth. In contrast, some studies (13, 47, 48) suggest that differences in the timing of blood sampling on birth outcome indicators may arise from two sources: first, PFAS level during pregnancy are affected by the diluting effect of the expanded blood volume during pregnancy, as well as by the accompanying decrease in serum albumin levels; second, PFAS level during pregnancy decreases due to the diluting effect of the expanded blood volume during pregnancy, and result in decreasing serum albumin levels and placental and fetal uptake. Salas et al. (109) found that in the third trimester to delivery, the median level of maternal serum PFOA decreased from 2.4 to 1.9 ng/ml. Fromme et al. (110) and Chen et al. (12) found that most PFAS levels, with exception of elevated PFBS level, decreased as pregnancy progressed, while PFHxS level remained unchanged (109, 111), which seems to be inherently related to the properties of PFAS (e.g., transrenal excretion of short-chain PFAS). The association between maternal blood volume and fetal growth has been demonstrated by Fromme et al. (110). Additionally, the differences in transplacental transfer of different PFAS lead to differences in PFAS level in maternal vs. cord blood. Kato et al. (112) found maternal and cord serum concentrations of PFOA to be ~1:1 and PFOS to be ~2:1. Therefore, the combined effect of changes in renal function and plasma volume may affect fetal growth and development in multiple ways by modulating PFAS levels through actions including reverse causality (12, 51). In fact, the glomerular filtration rate (GFR) can affect both PFAS level and birth outcomes, with Steenland et al. (48) found that late gestational blood corresponds to a greater GFR (40–50% increase), mainly in the third trimester. As Verner et al. (113) reported, babies with smaller BW tend to have mothers with lower GFRs, and indeed, higher levels of PFAS in blood do correspond to lower GFRs. The pharmacokinetics of PFAS during pregnancy may modulate fetal growth through reverse causality, as discussed in detail by Johnson et al. (51). Additionally, GFR may play a confounding or reverse causality role by affecting the renal clearance of different types of PFAS; for example, as for identical short-chain PFAS, renal function changes during pregnancy have a greater impact on PFBS (half-life 25.8 days) than on PFHxS (half-life 8.5 years). However, more evidence is needed to further clarify the role of changes in PFAS levels during pregnancy, PFAS structure, GFR and serum albumin in distorting the results.

In addition, the choice of data log-transformed or untransformed in the analysis should be carefully considered, given that dose-response relationships such as the “plateau phenomenon” (114) associated with exceeding background doses are not well studied for different PFAS. Darrow et al. (81) and Savitz et al. (78) found that log-transformed for PFOA is better in fitting data than untransformed. However, Steenland et al. (48) calculated the QIC [the goodness of fit statistic, analogous to the AIC (Akaike information criterion)] and concluded that the linear model was similar to the log-transformed model in terms of QIC, indicating that the two models in fitting the data are approximately well for PFOA at serum levels below 10 ng/ml. Furthermore, given the ambiguity of the dose-response relationship in the multiple PFAS-birth outcome pairs, we referred to the study of Negri et al. (47) for direct separate analyses without model reprocessing, and we did detect differences in the linear vs. log-transformed fit models (Supplementary Tables 6–10, 13, 14). However, this still requires a larger number of studies for in-depth interpretation.

Several mechanisms by which PFAS interferes with fetal development and growth have been proposed to explain some of the putative effects. Vanden Heuvel et al. (115) suggest that PFAS may be involved in the processes of insulin signaling, and regulation of glucose and lipid metabolism by activating peroxisome proliferator-activated receptor-alpha (PPARα) or PPARγ. Callan et al. (83) also observed alterations in lipid metabolism and thyroid hormone levels. The correlation of serum leptin and adiponectin levels, triglyceride concentrations, and insulin and HOMA-IR (homeostasis model assessment of insulin resistance) indices with PFAS has also been confirmed by *in vivo in vitro* trials (116, 117). In addition, Goudarzi et al. (118) found a negative association between PFOS and cord blood glucocorticoids, and an inverse relationship between PFAS and unsaturated fatty acids. PFAS is structurally similar to fatty acids; thus, Callan et al. (83) suggested that PFAS are likely to affect fatty acid metabolism. It is worth noted that some researchers proposed that different types of PFAS may involve different mechanisms. Fletcher et al. (119) reported an inverse and positive association between serum PFOA and PFOS levels and transcripts involved in cholesterol metabolism, respectively, in the C8 group. Unfortunately, the available evidence for this proposal is scarce. The existing experimental exposure doses in rodents are often 100–1,000 times higher than the human exposure level (47); thus, there is an urgent need to further clarify the different properties of the various PFAS and derivatives.

As previously described, the five available meta-analyses were flawed in either research methods or research design. Negri et al. (47) (PFOS and PFOA) examined BW with the number of included studies ranging from 12 to 29 and did not include additional literatures beyond 2020 (*n* = 8). Johnson et al. (51) aimed to examine associations of PFOA with multiple birth outcomes (BW, BL, PI, WC) but was quite inadequate in the number of included studies. Some studies (48, 49, 51) transformed data in log-transformed form, while Negri et al. (47) performed separate meta-analyses of log-transformed and untransformed data as two groups. The highlight is that Deji et al. (50) recently published a meta-analysis that attempted to analyze the association between PFOA, PFOS, PFNA, PFHxS and PTB, miscarriage, and stillbirth, but they incorrectly included two inappropriate studies. The first is Louis et al. (103), who examined the association between PFAS and “pregnancy loss,” which is fundamentally different from “miscarriages” or “stillbirth,” as we have described above. The other is Bjerregaard-Olesen et al. (120), who provided back-exponentiation of the linear parameter estimates [relative levels (RL)] of the association between PFAS and the number of miscarriages in 1,438 Danish pregnant women from 2008 to 2013. However, Deji et al. (50) directly combined RL values with other effect estimates (e.g., OR), without any interpretation, which is inappropriate. Additionally, they incorrectly combined log-transformed and untransformed data directly, and these effect estimates extracted from the continuous exposure analysis and the estimates used to generate the meta-analysis were inconsistently scaled and standardized so that the conclusions drawn had to be reconsidered. In addition, all of these studies have inappropriately combined effect estimates from categorical and continuous exposure analyses for the same exposure and outcome pair, confounding the hypothesis of a dose-response relationship as previously described. Only Johnson et al. (51) used the Navigation Guide systematic review methodology to determine the quality of the included studies in terms of risk of bias and level of evidence. Finally, a review from the United States examined the relationships of exposure to toxic metals and PFAS with preeclampsia and preterm delivery, but they did not directly attempt to synthesize the association between PFAS and preterm delivery. Dzierlenga et al. (49) and Steenland et al. (48) considered the effect of the time of blood sampling on outcomes, but they did not perform subgroup analyses of gender or other population characteristics.

The present study outweighs the existing meta-analyses in the following aspects. The greatest advantage is that our updates included the most recent studies, and only our study combined the various types of PFAS and birth outcome indicators available. In addition, we analyzed the effects of gender and time of blood collection by subgroup analyses. Furthermore, only our study adopted the NTP/OHAT framework, which is a recommended standard for conducting systematic reviews in the field of environmental health. Moreover, this study also performed meta-regression analysis to explore the source(s) of heterogeneity.

Equally strikingly, the results of the meta-analyses under the random-effects model and the quality-effects model were inconsistent. That is, only PFOA/PFOS/PFDA–BW, PFDoDA–BL and PFHpS–GA per 1 ln (ng/ml) increase in exposure; PFOA–PI and PFOS–BL per 1 ln (ng/ml) increase in exposure; and PFOA–BL/HC, PFOS–BW and PFNA–PTB for the highest vs. the lowest categories of exposure showed significant differences. The conflicting results suggest the need for further research to determine the relationship between each exposure and outcome.

## Strengths and Limitations

This study provides the most recent and comprehensive synthesis of evidence on the association of PFAS with birth outcomes. Birth outcome indicators including WC, BL, and GA were examined for the first time, and significant associations were discovered between several PFAS-birth outcome pairs, with almost all of the significant findings showing little heterogeneity. The key strengths of this study are the inclusion and broad coverage of the most recent research evidence, the rigorous, transparent, and reproducible assessment of the evidence, the use of standardized data processing methods to preserve the originality of the data as much as possible, and the comprehensive and systematic elucidation of the potential role of different types of PFAS exposure in increasing the risk of adverse birth outcomes. This study makes a prompt contribution to the rapidly evolving field of research, illuminates the changes confronting meta-analysis, and provides recommendations for improving the potential utility of future studies.

Despite these advantages, several limitations should be acknowledged. First, the associations of many other exposure-outcome pairs were not explored by our meta-analysis. This is because some other types of PFAS were inadequately explored by the current studies, including most long-chain PFAS (e.g., PFTrDA, PFTeDA) as well as short-chain congeners and functionalized perfluoropolyether as substitutes (e.g., chlorinated poly-fluoroalkyl ether sulfonic acids (Cl-PFESAs) or F-53B with China as the sole source of emissions (121), with some data coming from numerical processing of images as well as from formula conversion. Additionally, different data types (continuous vs. categorical exposure, log-transformed vs. untransformed) are not sufficiently grouped for a meta-analysis. Second, the generalizability of the study results was relatively inadequate. We must take into account that the type and prevalence of different birth outcomes are closely related to the level of public health prevention and control as well as the economy and socio-cultural environment of the area where pregnant women live. However, the majority of the included studies (34/46) were from developed countries/regions, which limits the generalizability of the findings. Third, the possibility of unknown or unmeasured confounders cannot be excluded from our summary effect estimates. Although potential confounders were controlled by the included studies, some important confounders, such as maternal age and GFR, were controlled in only a few of the included studies. Finally, the limited data that could be extracted from the included studies prohibited us from further analyzing the differences in maternal age and GFR, although we performed subgroup analyses to the extent possible. Given the limited amount of evidence observed, future studies should aim to elucidate the association between birth outcome indicators and PFAS exposure in terms of dose and causality.

## Conclusions

Our study showed that PFAS exposure was significantly associated with an increased risk of multiple adverse birth outcomes. Specifically, PFOS, PFOA, PFHxS, PFNA, PFUnDA, PFHpS, and PFDA exposures were negatively associated with BW; PFOA and PFOS exposures were negatively associated with BL and PI; PFOS and PFDoDA exposures were negatively associated with HC; PFHpS exposure was negatively associated with GA, and PFNA and PFHpS exposures were positively associated with PTB risk. Additionally, PFDA exposure was positively associated with risk of SGA. Further epidemiological studies, especially with cohort study designs, are needed to expand the sample size to further elaborate the effects of different types and doses of PFAS exposure on birth outcomes, which is essential to verify the causality and dose-response curves. In addition, future studies should use mediation analysis and fully consider residual confounders.

## Data Availability Statement

The original contributions presented in the study are included in the article/Supplementary Material, further inquiries can be directed to the corresponding author/s.

## Author Contributions

S-YG and Y-NC conceived the idea, performed the statistical analysis, and drafted this meta-analysis. K-JW, WL, and W-JW selected and retrieved relevant papers. H-RL, S-YG, and Y-NC assessed each study. C-YH and Z-XJ was the guarantor of the overall content. Z-LL and C-YH supervised the whole study process and contributed to the critical revision of the manuscript. All authors revised and approved the final manuscript.

## Funding

This study was supported by Key Projects of Natural Science Research of Anhui Provincial Department of Education (KJ2020A0163) and National Natural Science Foundation of China (82070986).

## Conflict of Interest

The authors declare that the research was conducted in the absence of any commercial or financial relationships that could be construed as a potential conflict of interest.

## Publisher's Note

All claims expressed in this article are solely those of the authors and do not necessarily represent those of their affiliated organizations, or those of the publisher, the editors and the reviewers. Any product that may be evaluated in this article, or claim that may be made by its manufacturer, is not guaranteed or endorsed by the publisher.
