# Supplementary material for: Association Between Exposure to Per- and Polyfluoroalkyl Substances and Birth Outcomes: A Systematic Review and Meta-Analysis
Source: Front Public Health. 2022 Mar 24;10:855348. doi: 10.3389/fpubh.2022.855348 (PMC8988915; doi:10.3389/fpubh.2022.855348)

Supplemental Materials A

Association between perfluoroalkyl substances exposure and birth outcomes: A systematic review and meta-analysis

Si-Yu Gui, Yue-Nan Chen, Ke-Jia Wu, Wen Liu, Wen-Jing Wang, Huan-Ru Liang, Zheng-Xuan Jiang, Ze-Lian Li, Cheng-Yang Hu

Table and Figure of contents

Table S1. Literature search strategy (dated to February 23th, 2021)

Table S2. Quality assessment of the included studies

Table S3. Criteria for the risk of bias assessment of each included study, adapted from the OHAT and Navigation Guide tool

Table S4. Details of risk of bias assessment for each study included in the systematic review and meta-analysis

Table S5. Sensitivity analyses of the associations between PFAS with diverse indicators of birth outcome for per 1 ng/ml increment of exposure.

Table S6. Sensitivity analyses of the associations between PFAS with diverse indicators of birth outcome for per 1 ln(ng/ml) increment of exposure.

Table S7. Sensitivity analyses of the associations between PFAS with diverse indicators of birth outcome for highest versus lowest of exposure.

Table S8. Confidence in the body of evidence and level of evidence for each exposure-outcome combination.

Table S9. Summary regression coefficients (β) or odds ratio (OR) and 95% CI by random effects meta-analysis stratified by different study characteristics (PFOA and PFOS were shown in table 4).

Fig S1. Forest plot on the effect of PFOA on BW (birth weight)/g (quality effects model) for (A) per 1 ln(ng/ml) increment , (B) per 1 ng/ml increment and (C) for high versus low categories of exposure.

Fig S2. Forest plot on the effect of PFOA on BL (birth length)/cm (quality effects model) for (A) per 1 ln(ng/ml) increment , (B) per 1 ng/ml increment and (C) for high versus low categories of exposure.

Fig S3. Forest plot on the effect of PFOA on HC (head circumference)/cm (quality effects model) for (A) per 1 ln(ng/ml) increment , (B) per 1 ng/ml increment and (C) for high versus low categories of exposure.

Fig S4. Forest plot on the effect of PFOA on GA (gestational age)/weeks (quality effects model) for (A) per 1 ln(ng/ml) increment , (B) for high versus low categories of exposure.

Fig S5. Forest plot on the effect of PFOA on PI (Ponderal index)/g/cm2 *100 (quality effects model) for (A) per 1 ln(ng/ml) increment , (B) per 1 ng/ml increment and (C) for high versus low categories of exposure.

Fig S6. Forest plot on the effect of PFOA on PTB (preterm birth) (quality effects model) for (A) per 1 ln(ng/ml) increment , (B) for high versus low categories of exposure.

Fig S7. Forest plot on the effect of PFOA on LBW (low birth weight) (quality effects model) for (A) per 1 ln(ng/ml) increment , (B) for high versus low categories of exposure.

Fig S8. Forest plot on the effect of PFOA on SGA (small for gestational age) (quality effects model) for (A) per 1 ln(ng/ml) increment , (B) for high versus low categories of exposure.

Fig S9. Forest plot on the effect of PFOS on BW (birth weight)/g (quality effects model) for (A) per 1 ln(ng/ml) increment , (B) per 1 ng/ml increment and (C) for high versus low categories of exposure.

Fig S10. Forest plot on the effect of PFOS on BL (birth length)/cm (quality effects model) for (A) per 1 ln(ng/ml) increment , (B) per 1 ng/ml increment and (C) for high versus low categories of exposure.

Fig S11. Forest plot on the effect of PFOS on HC (head circumference)/cm (quality effects model) for (A) per 1 ln(ng/ml) increment , (B) per 1 ng/ml increment and (C) for high versus low categories of exposure.

Fig S12. Forest plot on the effect of PFOS on GA (gestational age)/weeks (quality effects model) for (A) per 1 ln(ng/ml) increment , (B) for high versus low categories of exposure.

Fig S13. Forest plot on the effect of PFOS on PI (Ponderal index)/g/cm2 *100 (quality effects model) for (A) per 1 ln(ng/ml) increment , (B) per 1 ng/ml increment and (C) for high versus low categories of exposure.

Fig S14. Forest plot on the effect of PFOS on PTB (preterm birth) (quality effects model) for (A) per 1 ln(ng/ml) increment , (B) per 1 ng/ml increment and (C) for high versus low categories of exposure.

Fig S15. Forest plot on the effect of PFOS on LBW (low birth weight) (quality effects model) for (A) per 1 ln(ng/ml) increment , (B) for high versus low categories of exposure.

Fig S16. Forest plot on the effect of PFOS on SGA (small for gestational age) (quality effects model) for (A) per 1 ln(ng/ml) increment , (B) for high versus low categories of exposure.

Fig S17. Forest plot on the effect of PFDA on BW (birth weight)/g (quality effects model) for (A) per 1 ln(ng/ml) increment , (B) per 1 ng/ml increment and (C) for high versus low categories of exposure.

Fig S18. Forest plot on the effect of PFHxS on BW (birth weight)/g (quality effects model) for (A) per 1 ln(ng/ml) increment , (B) per 1 ng/ml increment and (C) for high versus low categories of exposure.

Fig S19. Forest plot on the effect of PFNA on BW (birth weight)/g (quality effects model) for (A) per 1 ln(ng/ml) increment , (B) per 1 ng/ml increment and (C) for high versus low categories of exposure.

Fig S20. Forest plot on the effect of PFDA on BW (birth weight)/g (quality effects model) for (A) per 1 ln(ng/ml) increment , (B) per 1 ng/ml increment and (C) for high versus low categories of exposure.

Fig S21. Forest plot on the effect of (A)PFDoDA (B)PFTrDA (C)PFHpA (D)PFDeA (E)PFOSA on BW (birth weight)/g (quality effects model) for per 1 ln(ng/ml) increment of exposure.

Fig S22. Forest plot on the effect of (a)PFNA (b)PFHxS on BL (birth length)/cm (quality effects model) for (A) per 1 ln(ng/ml) increment , (B) per 1 ng/ml increment and (C) for high versus low categories of exposure.

Fig S23. Forest plot on the effect of (A)PFDeDA (B)PFDA (C)PFDoDA (D)PFUnDA on BL (birth length)/cm (quality effects model) for per 1 ln(ng/ml) increment of exposure.

Fig S24. Forest plot on the effect of (a)PFNA (b)PFHxS on HC (head circumference)/cm (quality effects model) for (A) per 1 ln(ng/ml) increment and (B) per 1 ng/ml increment of exposure.

Fig S25. Forest plot on the effect of (A) PFDA (B) PFDeDA (C)PFDoDA (D)PFUnDA on HC (head circumference)/cm (quality effects model) for per 1 ln(ng/ml) increment of exposure.

Fig S26. Forest plot on the effect of (a)PFNA (b)PFHxS on GA (gestational age)/weeks (quality effects model) for (A) per 1 ln(ng/ml) increment and (B) per 1 ng/ml increment of exposure.

Fig S27. Forest plot on the effect of (A) PFDA (B) PFHpS (C)PFDoDA (D)PFUnDA (E)PFHpA on GA (gestational age)/weeks (quality effects model) for per 1 ln(ng/ml) increment of exposure.

Fig S28. Forest plot on the effect of PFHxS on PI (Ponderal index)/g/cm2 *100 (quality effects model) for (A) per 1 ln(ng/ml) increment , (B) per 1 ng/ml increment and (C) for high versus low categories of exposure.

Fig S29. Forest plot on the effect of (a)PFNA (b)PFUnDA (c)PFDA on PI (Ponderal index)/g/cm2 *100 (quality effects model) for per 1 ln(ng/ml) increment and (B) for high versus low categories of exposure.

Fig S30. Forest plot on the effect of (a)PFNA (b) PFHxS on PTB (preterm birth) (quality effects model) for per 1 ln(ng/ml) increment and (B) for high versus low categories of exposure.

Fig S31. Forest plot on the effect of (a)PFDA (b) PFUnDA on PTB (preterm birth) (quality effects model) for per 1 ln(ng/ml) increment of exposure.

Fig S32. Forest plot on the effect of (a)PFNA (b) PFHxS on LBW (low birth weight) (quality effects model) for per 1 ln(ng/ml) increment of exposure.

Fig S33. Forest plot on the effect of (a)PFDoDA (b) PFHxS (c)PFDeA (d)PFNA (e)PFUnDA on SGA (small for gestational age) (quality effects model) for per 1 ln(ng/ml) increment of exposure.

Fig S34. Doi plot and LFK index analysis of publication bias in reported associations between PFOA and BW (birth weight)/g for (A) per 1 ln(ng/ml) increment, (B) per 1 ng/ml increment and (C) for high versus low categories of exposure.

Fig S35. Doi plot and LFK index analysis of publication bias in reported associations between PFOA and BL (birth length)/cm for (A) per 1 ln(ng/ml) increment, (B) per 1 ng/ml increment and (C) for high versus low categories of exposure.

Fig S36. Doi plot and LFK index analysis of publication bias in reported associations between PFOA and HC (head circumference)/cm for (A) per 1 ln(ng/ml) increment, (B) for high versus low categories of exposure.

Fig S37. Doi plot and LFK index analysis of publication bias in reported associations between PFOA and GA (gestational age)/weeks for (A) per 1 ln(ng/ml) increment, (B) for high versus low categories of exposure.

Fig S38. Doi plot and LFK index analysis of publication bias in reported associations between PFOA and PI (Ponderal index)/g/cm2 *100 for (A) per 1 ln(ng/ml) increment, (B) for high versus low categories of exposure.

Fig S39. Doi plot and LFK index analysis of publication bias in reported associations between PFOA and PTB (preterm birth) for (A) per 1 ln(ng/ml) increment, (B) for high versus low categories of exposure.

Fig S40. Doi plot and LFK index analysis of publication bias in reported associations between PFOA and LBW (low birth weight) for (A) per 1 ln(ng/ml) increment, (B) for high versus low categories of exposure.

Fig S41. Doi plot and LFK index analysis of publication bias in reported associations between PFOA and SGA (small for gestational age) for (A) per 1 ln(ng/ml) increment, (B) for high versus low categories of exposure.

Fig S42. Doi plot and LFK index analysis of publication bias in reported associations between PFOS and BW (birth weight)/g for (A) per 1 ln(ng/ml) increment, (B) for high versus low categories of exposure.

Fig S43. Doi plot and LFK index analysis of publication bias in reported associations between PFOS and BL (birth length)/cm for (A) per 1 ln(ng/ml) increment, (B) per 1 ng/ml increment and (C) for high versus low categories of exposure.

Fig S44. Doi plot and LFK index analysis of publication bias in reported associations between PFOS and HC (head circumference)/cm for (A) per 1 ln(ng/ml) increment, (B) for high versus low categories of exposure.

Fig S45. Doi plot and LFK index analysis of publication bias in reported associations between PFOS and GA (gestational age)/weeks for (A) per 1 ln(ng/ml) increment, (B) for high versus low categories of exposure.

Fig S46. Doi plot and LFK index analysis of publication bias in reported associations between PFOS and PI (Ponderal index)/g/cm2 *100 for (A) per 1 ln(ng/ml) increment, (B) for high versus low categories of exposure.

Fig S47. Doi plot and LFK index analysis of publication bias in reported associations between PFOS and PTB (preterm birth) for (A) per 1 ln(ng/ml) increment, (B) for high versus low categories of exposure.

Fig S48. Doi plot and LFK index analysis of publication bias in reported associations between PFOS and LBW (low birth weight) for (A) per 1 ln(ng/ml) increment, (B) for high versus low categories of exposure.

Fig S49. Doi plot and LFK index analysis of publication bias in reported associations between PFOS and SGA (small for gestational age) for (A) per 1 ln(ng/ml) increment, (B) for high versus low categories of exposure.

Fig S50. Doi plot and LFK index analysis of publication bias in reported associations between PFDA on BW (birth weight)/g for (A) per 1 ln(ng/ml) increment, (B) for high versus low categories of exposure.

Fig S51. Doi plot and LFK index analysis of publication bias in reported associations between PFHxS on BW (birth weight)/g for (A) per 1 ln(ng/ml) increment, (B) for high versus low categories of exposure.

Fig S52. Doi plot and LFK index analysis of publication bias in reported associations between PFNA on BW (birth weight)/g for (A) per 1 ln(ng/ml) increment, (B) per 1 ng/ml increment and (C) for high versus low categories of exposure.

Fig S53. Doi plot and LFK index analysis of publication bias in reported associations between PFDA on BW (birth weight)/g for high versus low categories of exposure.

Fig S54. Doi plot and LFK index analysis of publication bias in reported associations between (A)PFDoDA (B)PFHpA (C)PFDeA on BW (birth weight)/g (quality effects model) for per 1 ln(ng/ml) increment of exposure.
Fig S55. Doi plot and LFK index analysis of publication bias in reported associations between (a)PFNA (b)PFHxS on BL (birth length)/cm for (A) per 1 ln(ng/ml) increment , (B) per 1 ng/ml increment and (C) for high versus low categories of exposure.
Fig S56. Doi plot and LFK index analysis of publication bias in reported associations between(A)PFDA (B)PFDoDA (C)PFUnDA on BL (birth length)/cm (quality effects model) for per 1 ln(ng/ml) increment of exposure.
Fig S57. Doi plot and LFK index analysis of publication bias in reported associations between (a)PFNA (b)PFHxS on HC (head circumference)/cm for per 1 ln(ng/ml) increment of exposure.
Fig S58. Doi plot and LFK index analysis of publication bias in reported associations between(A)PFDA (B)PFDoDA (C)PFUnDA on HC (head circumference)/cm for per 1 ln(ng/ml) increment of exposure.
Fig S59. Doi plot and LFK index analysis of publication bias in reported associations between (a)PFNA (b)PFHxS on GA (gestational age)/weeks for (A) per 1 ln(ng/ml) increment and (B) per 1 ng/ml increment of exposure.
Fig S60. Doi plot and LFK index analysis of publication bias in reported associations between(A)PFDA (B)PFUnDA on GA (gestational age)/weeks for per 1 ln(ng/ml) increment of exposure.
Fig S61. Doi plot and LFK index analysis of publication bias in reported associations between PFHxS on PI (Ponderal index)/g/cm2 *100 for (A) per 1 ln(ng/ml) increment and (B) per 1 ng/ml increment of exposure.
Fig S62. Doi plot and LFK index analysis of publication bias in reported associations between (a)PFNA (b)PFUnDA (c)PFDA on PI (Ponderal index)/g/cm2 *100 for per 1 ln(ng/ml) increment of exposure.

Fig S63. Doi plot and LFK index analysis of publication bias in reported associations between (a)PFNA (b) PFHxS on PTB (preterm birth) for per 1 ln(ng/ml) increment and (B) for high versus low categories of exposure.
Fig S64. Doi plot and LFK index analysis of publication bias in reported associations between PFNA on LBW (low birth weight) for per 1 ln(ng/ml) increment of exposure.
Fig S65. Doi plot and LFK index analysis of publication bias in reported associations between (a)PFNA (b)PFUnDA on SGA (small for gestational age) for per 1 ln(ng/ml) increment of exposure.

Table S1. Literature search strategy (dated to February 23th, 2021)

Database	Search strategies	**Results**
PubMed	(perfluoride[Title/Abstract] OR perfluorinated[Title/Abstract] OR perfluoroalkyl[Title/Abstract] OR pfc[Title/Abstract] OR pfcs[Title/Abstract] OR pfass[Title/Abstract] OR fluoride[Title/Abstract] OR fluorides[Title/Abstract] OR fluorinated[Title/Abstract] OR fluorine[Title/Abstract] OR polyfluorinated[Title/Abstract] OR pfhxa [Title/Abstract] OR pfhpa[Title/Abstract] OR pfoa[Title/Abstract] OR pfna[Title/Abstract] OR pfda[Title/Abstract] OR pfunda[Title/Abstract] OR pfdoda[Title/Abstract] OR pftrda[Title/Abstract] OR pfbs[Title/Abstract] OR pfhxs[Title/Abstract] OR pfos[Title/Abstract] OR mefosaa[Title/Abstract] OR etfosaa[Title/Abstract] OR pfca[Title/Abstract]) AND (birth outcomes[Title/Abstract] OR adverse birth outcomes[Title/Abstract]	261

	OR birth weight[Title/Abstract] OR low birth weight[Title/Abstract] OR preterm birth[Title/Abstract] OR gestational age[Title/Abstract] OR small for gestational age[Title/Abstract])	
Web of Science	TOPIC: (perfluoride OR perfluorinated OR perfluoroalkyl OR pfc OR pfcs OR pfass OR fluoride OR fluorides OR fluorinated OR fluorine OR polyfluorinated OR pfhxa OR pfhpa OR pfoa OR pf na OR pfda OR pfunda OR pfdoda OR pftrda OR pfbs OR pfhxs OR pfos OR mefosaa OR etfos aa OR pfca) AND TOPIC: (birth outcomes OR adverse birth outcomes OR birth weight OR low birth weight OR preterm birth OR gestational age OR small for gestational age)<br>Timespan: All years. Indexes: SCI-EXPANDED, CCR-EXPANDED, IC.	452
EMBASE	(perfluoride:ti,ab,kw OR perfluorinated:ti,ab,kw OR perfluoroalkyl:ti,ab,kw OR pfc:ti,ab,kw OR pfcs:ti,ab,kw OR pfass:ti,ab,kw OR fluoride:ti,ab,kw OR fluorides:ti,ab,kw OR fluorinated:ti,ab,kw OR fluorine:ti,ab,kw OR polyfluorinated:ti,ab,kw OR pfhxa:ti,ab,kw OR pfhpa:ti,ab,kw OR pfoa:ti,ab,kw OR pfna:ti,ab,kw OR pfda:ti,ab,kw OR pfunda:ti,ab,kw OR pfdoda:ti,ab,kw OR pftrda:ti,ab,kw OR pfbs:ti,ab,kw OR pfhxs:ti,ab,kw OR pfos:ti,ab,kw OR mefosaa:ti,ab,kw OR etfosaa:ti,ab,kw OR pfca:ti,ab,kw) AND ('birth outcomes':ti,ab,kw OR 'adverse birth outcomes':ti,ab,kw OR 'birth	205

	weight':ti,ab,kw OR 'low birth weight':ti,ab,kw OR 'preterm birth':ti,ab,kw OR 'gestational age':ti,ab,kw OR 'small for gestational age':ti,ab,kw)	

Table S2. Quality assessment of the included studies.

Reference	Sample size	Study design	Study site	Time frame	Response rate	Outcome definition	Outcome assessment	Assessment of PFAS	Bio-phsyco-social plausibility	Adjustments for personal covariates	Adjustments for environmental covariates	Effect size calculation	Quality
Benjamin J. Apelberg (2007)	1	0.25	1	1	1	1	1	1	0.75	1	0.75	0.75	0.88
Chunyuan Fei (2007)	1	1	1	1	0.75	1	1	1	0.75	1	0.75	0.75	0.92
Chunyuan Fei (2008)	1	1	1	1	0.75	0.5	1	1	0.75	1	1	0.75	0.90
Noriaki Washino (2009)	1	1	1	1	0.5	1	1	1	0.75	1	0.5	0.75	0.88
Michele P. Hamm (2010)	1	1	1	1	1	1	1	1	0.75	0.75	0.5	1	0.92
Mei-Huei Chen (2012)	1	1	1	1	1	1	1	1	0.75	1	0.5	1	0.94
Mildred Maisonet (2012)	1	1	0.75	1	1	1	1	1	0.75	0.75	0	1	0.85

David A. Savitz (2012)	1	1	1	1	1	0.5	0.25	1	0.75	0.5	1	0.75	0.81
Kristina W. Whitworth (2012)	1	1	1	1	0.75	0.5	1	1	0.75	0.75	1	0.75	0.88
Kusheng Wu (2012)	1	0.25	1	1	1	1	1	1	0.75	0.75	0.5	0.75	0.83
Lyndsey A. Darrow (2013	1	1	1	1	1	0.5	1	1	0.75	0.75	0.5	1	0.88
Candace A. Robledo (2015)	1	1	1	1	0.75	1	1	1	0.75	0.75	0.5	0.75	0.88
Eman Alkhalawi (2016)	1	1	1	1	1	1	0.25	1	0.75	0.5	0.75	0.75	0.83
Cathrine Carlsen Bach (2016)	1	1	1	1	1	0.5	1	1	0.75	0.5	0.5	0.75	0.83
A.C. Callan (2016)	0.5	0.25	1	1	1	1	1	1	0.75	0.5	1	0.75	0.81

Eun Jin Kwon (2016)	1	0.25	1	1	1	0.5	1	1	0.75	0.75	0.5	0.75	0.79
Eung-Sun Lee (2016)	0.5	0.25	1	1	1	0.5	1	1	0.75	0.75	0.5	1	0.77
Virissa Lenters (2016)	1	1	1	1	0.75	0.5	1	1	0.75	0.25	1	0.75	0.83
Yan Wang (2016)	1	1	1	1	1	1	1	1	0.75	0.75	0.5	0.75	0.90
Jillian Ashley-Martin (2017)	1	1	1	1	0.75	0.5	1	1	0.75	0.5	1	0.75	0.85
Mei-Huei Chen (2017)	1	1	1	1	1	0.5	1	1	0.75	0.5	0.5	0.75	0.83
Hilde B. Lauritzen (2017)	1	0.75	1	1	1	1	1	1	0.75	0.75	0.75	0.75	0.90
Meng Li (2017)	1	0.25	1	1	1	0.5	1	1	0.75	0.75	0.75	0.75	0.81

Cyntia B. Manzano-Salgado (2017)	1	1	1	1	1	1	1	1	0.75	0.5	0.5	0.75	0.88
Machiko Minatoya (2017)	1	1	1	1	0.75	0.5	1	1	0.75	0.75	0.75	0.75	0.85
Yu Shi (2017)	1	0.25	1	1	1	1	0.25	1	0.75	0.75	1	0.75	0.81
Anne P. Starling (2017)	1	1	1	1	0.75	0.5	1	1	0.75	1	0.5	0.75	0.85
Damaskini Valvi (2017)	1	1	1	1	1	1	1	1	0.75	0.75	0.5	0.75	0.90
Wencheng Cao (2018)	1	1	1	1	0.75	1	1	1	0.75	0.5	0.75	1	0.90
Qi Meng (2018)	1	1	1	1	1	1	1	1	0.75	0.5	0.75	0.75	0.90
Lisa B. Rokoff (2018)	1	1	1	1	0.75	0.5	1	1	0.75	0.5	0.5	0.75	0.81

Sharon K. Sagiv* (2018)	1	1	1	1	0.75	1	1	1	0.75	1	0.5	0.75	0.90
Jessica Shoaff (2018)	1	1	1	1	1	0.5	1	1	0.75	1	0.75	0.75	0.90
Christian Bjerregaard-Olesen (2019)	1	1	1	1	0.75	1	1	1	0.75	0.5	0.5	0.75	0.85
Geetika Kalloo (2019)	1	1	1	1	1	1	1	1	0.75	0.75	0	0.75	0.85
Kristin J. Marks (2019)	1	1	1	1	0.75	1	1	1	0.75	0.5	0.5	0.75	0.85
Hexing Wang (2019)	1	0.25	1	1	1	1	1	1	0.75	1	0.75	0.75	0.88
Chenye Xu (2019)	0.5	1	1	1	0.75	0.5	1	1	0.75	0.75	1	0.75	0.83
Stephanie M. Eick (2020)	1	1	1	1	0.75	1	1	1	0.75	0.5	0.75	0.75	0.88

Marie Harthøj Hjermitslev (2020)	1	0.25	1	1	1	0.5	1	1	0.75	0.75	1	1	0.85
Xiaona Huo (2020)	1	1	1	1	1	0.5	1	1	0.75	0.75	0.75	1	0.90
Ikuko Kashino (2020)	1	1	1	1	0.75	1	1	1	0.75	0.75	0.75	0.75	0.90
Xiaotu Liu (2020)	1	0.75	1	1	1	0.5	1	1	0.75	1	0.75	0.75	0.88
Marilia Cristina Oliveira Souza (2020)	1	0.75	1	1	1	0.5	1	1	0.75	0.1	0.75	1	0.82
Sverre Wikström (2020)	1	1	1	1	0.75	0.5	1	1	0.75	0.75	0.75	0.75	0.85
Lin Chen (2021)	1	1	1	1	0.75	1	1	1	0.75	1	0.75	0.75	0.92

Note: Sample size: 1 – completely satisfactory/justified by power analysis; 0.5 – somewhat satisfactory; 0 – not sufficient/not justified.

Design: 1 – cohort; 0.75 – case-control; 0.25 – cross-sectional/ecological.
Study country: 1 – with good working and living conditions/high socio-economic standard; 0.5 – difficult conditions/lower socio-economic standard; 0.25 – very difficult conditions/very low socio-economic standard; 0 – not reported.
Timeframe: 1 – reported; 0 – not reported.
Response rate: 1 – ≥ 80%/considerable part of the population; 0.75 – 60 to 80 %; 0.5 – < 60%; 0 – not reported.
Definition of outcome: 1 – clearly define all outcomes; 0.5 – define partly outcomes; 0 – not reported.
Assessment of outcome: 1 – medical records/physician's exam/valid database; 0.25 – self-report; 0 – not specified.
Assessment of PFAS exposure: 1 – Testing by body fluids (serum indicators); 0.75 –The diet-history questionnaire or environmental measurements in living (occupationally exposed) areas report ; 0.5 – not study-specific official records; 0.25 – self-report.
Adjustments for personal covariates (cov): 1 – 12 to 17 (cov) including parity or maternal age or maternal education; 0.75 – 6 to 12 (cov) including parity or maternal age or maternal education; 0.5 – 1 to 6 (cov) including parity or maternal age or maternal education; 0.25 – 6 to 17 (cov) without parity or maternal age or maternal education; 0.1 – 1 to 6 (cov) without parity or maternal age or maternal education; 0 – no covariates (According to the directed acyclic graph (DAG) (A and B) described by (Perfluoroalkyl Acids in Maternal Serum and Indices of Fetal Growth: The Aarhus Birth Cohort Cathrine Carlsen Bach, 2016) which portrays the most reliable causal network connecting exposure (PFAS), outcome (A illustrating the birth weight, also applies to the outcomes birth length and head circumference at birth; B illustrating the preterm birth, also applies to the continuous gestational age outcome), relevant confounders for adverse outcomes are: parity or maternal age or maternal education or infant sex or multiple pregnancy or fetal growth or maternal smoking or gestational age at birth, maternal pre-pregnancy BMI, ethnicity; marital status/single mother; obstetric history; air pollution/noise; urbanization; greenness of residencey. The scoring above indicates the score, given the number of covariates adjusted for, and whether age and gender was one of them).
Adjustments for environmental covariates: 1 – organochlorine pesticides (OCPs) + polybrominated diphenylethers (PBDEs) + polychlorinated biphenyls (PCBs); 0.75 – organochlorine pesticides (OCPs) + polybrominated diphenylethers (PBDEs); 0.5 –

one or two of OCPs + PBDEs + PCBs, 0 – no adjustments.
Effect size calculation for meta-analysis: 1 – no transformations and no data imputation; 0.75 – mild transformation and no data imputation; 0.5 – several transformations and no data imputation, 0.25 – considerable transformations and data imputation.

Table S3. Criteria for the risk of bias assessment of each included study, adapted from the OHAT and Navigation Guide tool.

Bias		Risk of Bias Domains and Ratings	Answer
**Key Criteria**	**Detection bias, exposure assessment**	Can we be confident in the exposure characterization?	-LOW risk: There is high confidence that the exposure to residential greenness is the true average population exposure.<br><br>-PROBABLY LOW: There is indirect evidence that suggests low risk of bias.<br><br>-PROBABLY HIGH risk: There is insufficient information to permit a judgment of high risk of bias, but there is indirect evidence that suggests high risk of bias.<br><br>-HIGH risk: There is direct evidence of high risk of misclassification bias.

	**Detection bias, outcome assessment**	Can we be confident in the outcome assessment?	-LOW risk: Outcome was classified based on diagnosis standard criteria (International Classification System code) and provided by a national or regional database.<br><br>-PROBABLY LOW: Outcome was assessed based on diagnosis standard criteria and collected by researcher<br><br>-PROBABLY HIGH risk: Outcome was not assessed based on standard diagnosis criteria AND is accompanied by validation sub-study or sensitivity analysis to suggest that the risk is minimum.<br><br>-HIGH risk: Outcome was assessed based on self-reports (parents, family) and data collected by the researcher.

	**Confounding bias**	Did the study design or analysis account for important confounding and modifying variables?	-LOW risk: Study accounted for all important confounders which were measured consistently<br><br>-PROBABLY LOW: Study accounted for most of confounders AND is not expected to introduce bias<br><br>-PROBABLY HIGH risk: Study accounted for some but not all of confounders AND is expected to introduce bias<br><br>-HIGH risk: Study did not account for potential confounders OR were inappropriately measured
**Other Criteria**	**Selection bias**	Did selection of study participants result in appropriate comparison groups?	-LOW risk: The descriptions of the studied population were sufficiently detailed to support the assertion that risk of selection effects was minimal.

			-PROBABLY LOW risk: There is insufficient information about population selection to permit a judgment of low risk of bias, but there is indirect evidence that suggests low risk of bias.<br><br>-PROBABLY HIGH risk: There is insufficient information about population selection to permit a judgment of high risk of bias, but there is indirect evidence that suggests high risk of bias.<br><br>- HIGH risk: There were indications from descriptions of the studied population of high risk of bias.
	**Attrition/exclusion bias**	Were outcome data complete without attrition or exclusion from analysis?	-LOW risk: There were no missing outcome data or missing data unrelated to true outcome

			-PROBABLY LOW: There was insufficient information about incomplete data to judge for low risk, but indirect evidence that suggests low risk of bias<br><br>-PROBABLY HIGH risk: There was insufficient information about incomplete data to judge for high risk, but indirect evidence that suggests high risk<br><br>-HIGH risk: Missing outcome data is related to true outcome
	**Selective reporting bias**	Were all measured outcomes reported?	-LOW risk: All of the studies pre-specified outcomes and findings are reported<br><br>-PROBABLY LOW: There was insufficient information about selective outcome to judge for low risk, but

			indirect evidence that suggests study was free of selective report<br><br>-PROBABLY HIGH risk: There was insufficient information about selective reporting to judge for high risk, but indirect evidence suggests that study was not free of selective reporting<br><br>-HIGH risk: Not all pre-specified outcomes and findings were reported, or one/more of the primary outcomes or analyses were assessed or executed with other methods than the pre-specified one, or one/more of the reported outcomes/findings was/were not pre-specified
	**Conflict of interest**	Potential source of bias in reporting through source of funding	-LOW risk: The study did not receive funding from an entity with financial interest in the outcome of study

			-PROBABLY LOW: There is insufficient information to judge for low risk, but indirect evidence suggests study was free of financial interest<br><br>-PROBABLY HIGH risk: There is insufficient information to judge for high risk, but indirect evidence suggests study was not free of financial interest<br><br>-HIGH risk: The study received support from an entity with financial interest in the outcome of study
	**Other source of bias**	Bias due to other problems not covered elsewhere (statistical methods were appropriate and researchers adhere to the study protocol)	-LOW risk: No other sources of bias<br><br>-PROBABLY LOW: There is insufficient information to judge for low risk, but indirect evidence suggests study was free of other problems

			-PROBABLY HIGH risk: There is insufficient information to judge for high risk, but indirect evidence suggests study was not free of other problems<br><br>-HIGH risk: There was at least one important risk of bias

Table S4. Details of risk of bias assessment for each study included in the systematic review and meta-analysis.

Study ID	Domain	Judgement	Comment
**Benjamin J. Apelberg (2007)**	**DETECTION BIAS [Key element]:** can we be confident in the exposure characterization?	Probably low	Cord serum samples were analyzed for PFOS and PFOA by online solid-phase extraction.
	**DETECTION BIAS [Key element]:** can we be confident in the outcome assessment?	Probably low	The author abstracted maternal and infant characteristics from clinical data bases maintained by the hospital.
	**CONFOUNDING BIAS [Key element]:** did the study design or analysis account for important confounding and modifying variables?	Definitely low	Analysis were adjusted to parity, gestational age, Maternal age, BMI, race, smoking, baby sex, height, net weight gain, diabetes, and hypertension. For head circumference: adjusted model also includes delivery mode (C-section/vaginal).
	**SELECTION BIAS:** did selection of study participants result in appropriate comparison groups?	Definitely high	The author conducted a hospital-based cross-sectional epidemiologic study of singleton deliveries in Baltimore, Maryland.

	**ATTRITION/EXCLUSION BIAS:** were outcome data incomplete due to attrition or exclusion from analysis?	Probably low	There is no evidence of exclusions that may bias the results reported in the manuscript.
	**SELECTIVE REPORTING BIAS:** were all measured outcomes reported?	Probably low	The authors stated: "Despite relatively low cord serum concentrations, we observed small negative associations between both PFOS and PFOA concentrations and birth weight and size.", so all outcomes have been reported.
	**CONFLICT OF INTEREST**	Probably low	The authors declare they have no competing financial interests.
**Chunyuan Fei （2007）**	**DETECTION BIAS [Key element]:** can we be confident in the exposure characterization?	Probably low	We used analyses of variance and linear regression to assess the associations between birth weight and length of gestation and maternal plasma PFOS and PFOA levels.
	**DETECTION BIAS [Key element]:** can we be confident in the outcome assessment?	Probably low	The authors obtained birth outcome information from medical records.
	**CONFOUNDING BIAS [Key element]:** did the study design or analysis account for important confounding and modifying	Probably low	Analysis were adjusted to infant sex, maternal age, socio-occupational status, pregnancy BMI and cigarette smoking during pregnancy, parity, gestational age.

	variables?		
	**SELECTION BIAS:** did selection of study participants result in appropriate comparison groups?	Definitely low	This study randomly selected 1,400 women and their infants from the Danish National Birth Cohort.
	**ATTRITION/EXCLUSION BIAS:** were outcome data incomplete due to attrition or exclusion from analysis?	Probably low	There is no evidence of exclusions that may bias the results reported in the manuscript. The cross-sectional design prevents the loss of follow-up of participants.
	**SELECTIVE REPORTING BIAS:** were all measured outcomes reported?	Probably low	The authors stated: "Our nationwide cohort data suggest an inverse association between maternal plasma PFOA levels and birth weight.", so all outcomes have been reported.
	**CONFLICT OF INTEREST**	Probably low	The authors stated:"J.K.M. and R.E.T. are employed by IEI, which has received funding from 3M. The other authors declare they have no competing financial interests".
**Chunyuan Fei （2008）**	**DETECTION BIAS [Key element]:** can we be confident in the exposure characterization?	Probably low	Blood was then separated and stored in freezers or in liquid nitrogen. Plasma concentrations of PFOS and PFOA were measured blindly at the 3M Toxicology Laboratory (St. Paul, Minnesota) using high performance liquid chromatography-tandem mass spectrometry based on the methods described by Ehresman et al.

	**DETECTION BIAS [Key element]:** can we be confident in the outcome assessment?	Probably low	Data were also extracted from the National Hospital Discharge Register as recorded by the midwives and were usually based on early ultrasound estimates.
	**CONFOUNDING BIAS [Key element]:** did the study design or analysis account for important confounding and modifying variables?	Definitely low	Analysis adjusted for parity, gestational age, quadratic gestational age, infant sex (male or female), maternal age (<25, 25–29, 30–34, or 35 years), sociooccupational status (high, middle, or low), cigarette smoking (nonsmoker, former smoker, smoker of 1–9 cigarettes/day, or smoker of 10 cigarettes/day), pre-pregnancy body mass index (weight(kg)/squared height (m2); <18.5, 18.5–24.9, 25.0–29.9, or 30.0), and gestational week at blood drawing.
	**SELECTION BIAS:** did selection of study participants result in appropriate comparison groups?	Definitely low	The authors stated: "We used data from the Danish National Birth Cohort, which was created in 1996–2002 for a nationwide study of pregnant women and their offspring". Thus, selection bias may be ruled out.
	**ATTRITION/EXCLUSION BIAS:** were outcome data incomplete due to attrition or exclusion from analysis?	Probably low	There is no evidence of exclusions that may bias the results reported in the manuscript. The cross-sectional design prevents the loss of follow-up of participants.
	**SELECTIVE REPORTING BIAS:** were all measured	Probably low	The authors stated: "In conclusion, our data showed that maternal blood PFOA levels in early pregnancy were associated with small decreases in birth length and abdominal circumference, but no

	outcomes reported?		association was found between PFOS exposure and any measure of fetal growth.". So, all the outcomes have been reported.
	**CONFLICT OF INTEREST**	Probably low	None declared conflict of interest.
**Noriaki Washino （2009）**	**DETECTION BIAS [Key element]:** can we be confident in the exposure characterization?	Probably low	The author analyzed maternal serum samples for PFOS and PFOA by liquid chromatography–tandem mass spectrometry (LC/MS/MS).
	**DETECTION BIAS [Key element]:** can we be confident in the outcome assessment?	Probably low	The authors stated: "We obtained characteristics of the mothers and infants from self-administered questionnaire surveys and from medical records".
	**CONFOUNDING BIAS [Key element]:** did the study design or analysis account for important confounding and modifying variables?	Definitely low	Analysis adjusted for maternal age, maternal educational level, smoking status during pregnancy, maternal BMI, infant sex, and blood sampling period, delivery mode (caesarean section/vaginal), parity, gestational age.
	**SELECTION BIAS:** did selection of study participants result in appropriate comparison groups?	Definitely low	The author conducted a hospital-based prospective cohort study between July 2002 and October 2005 in Sapporo, Japan.

	**ATTRITION/EXCLUSION BIAS:** were outcome data incomplete due to attrition or exclusion from analysis?	Probably low	The authors stated: "We obtained the information for exclusion criteria.". There is no evidence of exclusion bias.
	**SELECTIVE REPORTING BIAS:** were all measured outcomes reported?	Probably low	The authors stated: "Our results indicate that in utero exposure to relatively low levels of PFOS was negatively correlated with birth weight.". So all the outcomes have been reported.
	**CONFLICT OF INTEREST**	Probably low	The authors declare they have no competing financial interests.
**Michele P. Hamm （2010）**	**DETECTION BIAS [Key element]:** can we be confident in the exposure characterization?	Probably low	The analysis of PFAs in serum was performed in-house using methods previously described in detail.
	**DETECTION BIAS [Key element]:** can we be confident in the outcome assessment?	Probably low	Data were collected from delivery records that are completed at the time of birth for 499% of all hospital and midwife-attended births in the province of Alberta. This information was obtained from the Alberta Perinatal Health Program (APHP; www.aphp.com).
	**CONFOUNDING BIAS [Key element]:** did the study design or analysis account for important confounding and modifying	Probably high	Analysis adjusted for gestational age, maternal age, maternal race, gravida, maternal weight and height, and smoking infant gender.

	variables?		
	**SELECTION BIAS:** did selection of study participants result in appropriate comparison groups?	Definitely low	The study population was a cohort of 252 pregnant women who elected to undergo a second trimester prenatal “triple screen” at 15–16 weeks of gestation for Down’s syndrome, trisomy 18, and open spina bifida in the (former) Capital Health administrative region in and around the city of Edmonton, Alberta, Canada.
	**ATTRITION/EXCLUSION BIAS:** were outcome data incomplete due to attrition or exclusion from analysis?	Probably low	There is no evidence of exclusion bias.
	**SELECTIVE REPORTING BIAS:** were all measured outcomes reported?	Probably low	The authors stated: “These results do not suggest that maternal PFA exposure has a substantial effect on fetal weight and length of gestation.”. So all the outcomes have been reported.
	**CONFLICT OF INTEREST**	Probably low	The authors declare no conflict of interest.
**Mei-Huei Chen （2012）（2010）**	**DETECTION BIAS [Key element]:** can we be confident in the exposure characterization?	Definitely low	Cord blood was collected for PFOA, PFOS, PFNA, and PFUA analysis by ultra-high-performance liquid chromatography/tandem mass spectrometry.
	**DETECTION BIAS [Key element]:** can we be confident in	Probably low	Demographic data were obtained by interviewing mothers using a structured questionnaire and birth outcomes were extracted from medical records.

	the outcome assessment?		
	**CONFOUNDING BIAS [Key element]:** did the study design or analysis account for important confounding and modifying variables?	Probably low	Maternal age, pre-pregnancy body mass index, education level, log (Ln)-transformed cord blood cotinine levels, type of delivery and infant sex birth length, head circumference, and Ponderal index, parity, gestational age.
	**SELECTION BIAS:** did selection of study participants result in appropriate comparison groups?	Definitely low	The study subjects were from the Taiwan Birth Panel Study (TBPS), a longitudinal birth cohort study that was conducted at one medical center in Taipei and one local hospital and two clinics in New Taipei from April 2004 to January 2005.
	**ATTRITION/EXCLUSION BIAS:** were outcome data incomplete due to attrition or exclusion from analysis?	Probably low	There is no evidence of exclusion bias.
	**SELECTIVE REPORTING BIAS:** were all measured outcomes reported?	Probably high	The authors stated: "An adverse dose-dependent association was observed between prenatal PFOS exposure and birth outcomes. However, no associations were found for the other examined PFCs." .
	**CONFLICT OF INTEREST**	Probably low	The authors have declared that no competing interests exist.

**Mildred Maisonet (2012)**	**DETECTION BIAS [Key element]:** can we be confident in the exposure characterization?	Probably low	We used this sample to study associations of maternal exposures to PFCs, estimated from the serum concentrations, with fetal and postnatal growth.
	**DETECTION BIAS [Key element]:** can we be confident in the outcome assessment?	Definitely low	Date were abstracted from medical record and health professionals as part of the routine infant health surveillance program.
	**CONFOUNDING BIAS [Key element]:** did the study design or analysis account for important confounding and modifying variables?	Probably high	Adjust for maternal smoking during pregnancy, maternal prepregnancy BMI, previous live births, and gestational age.
	**SELECTION BIAS:** did selection of study participants result in appropriate comparison groups?	Probably high	The authors stated: "We studied a sample of 447 singleton girls and their mothers participating in the Avon Longitudinal Study of Parents and Children (ALSPAC)".
	**ATTRITION/EXCLUSION BIAS:** were outcome data	Probably high	The authors stated: "Results of such analyses suggested that exclusion of subjects missing values for theses covariates had the potential to introduce bias because changes in the magnitude and direction of bivariate associations were observed after these exclusions.".

	incomplete due to attrition or exclusion from analysis?		
	**SELECTIVE REPORTING BIAS:** were all measured outcomes reported?	Probably low	Girls with higher prenatal exposure to each of the PFCs examined were smaller at birth than those with lower exposure. In addition, those with higher exposure to PFOS were larger at 20 months. So all the outcomes have been reported.
	**CONFLICT OF INTEREST**	Probably low	The authors declare they have no actual or potential competing financial interests.
**David A. Savitz (2012)**	**DETECTION BIAS [Key element]:** can we be confident in the exposure characterization?	Probably low	Serum PFOA was measured, and reproductive and residential histories were obtained during 2005–2006.
	**DETECTION BIAS [Key element]:** can we be confident in the outcome assessment?	Probably high	Self-reported information on pregnancy outcomes allowed.
	**CONFOUNDING BIAS [Key element]:** did the study design or analysis account for important confounding and modifying variables?	Definitely high	Analysis adjusted for exposure year, maternal age, parity, education level at interview, smoking status at interview.
	**SELECTION BIAS:** did	Definitely	The authors stated: "The study population is derived from enrollees in the C8

	selection of study participants result in appropriate comparison groups?	low	Health Project". Thus, selection bias may be not ruled out.
	**ATTRITION/EXCLUSION BIAS:** were outcome data incomplete due to attrition or exclusion from analysis?	Probably low	There is no evidence of exclusions that may bias the results reported in the manuscript. The cross-sectional design prevents the loss of follow-up of participants.
	**SELECTIVE REPORTING BIAS:** were all measured outcomes reported?	Probably low	The authors stated: "In this large, population-based study in a region with markedly elevated PFOA exposure, we found no associations between estimated serum PFOA levels and adverse pregnancy outcomes other than possibly preeclampsia.". So all the outcomes haven been reported.
	**CONFLICT OF INTEREST**	Probably low	The authors reported no other financial interests related to this research.
**Kristina W. Whitworth (2012)**	**DETECTION BIAS [Key element]:** can we be confident in the exposure characterization?	Definitely low	PFC concentrations were measured in150lL of plasma by using high-performance liquid chromatography/tandem mass spectrometry at the Norwegian Institute of Public Health.
	**DETECTION BIAS [Key element]:** can we be confident in the outcome assessment?	Probably low	MoBa pregnancies were linked with the Medical Birth Registry of Norway, which includes pregnancy and birth records reported by the physician or midwife attending the childbirth.

	**CONFOUNDING BIAS [Key element]:** did the study design or analysis account for important confounding and modifying variables?	Probably high	Analysis adjusted for maternal age, pre-pregnancy body mass index, albumin concentration, maternal education, interpregnancy interval, quadratic interpregnancy interval, and consumption of lean fish, parity.
	**SELECTION BIAS:** did selection of study participants result in appropriate comparison groups?	Definitely low	This study was based on the Norwegian Mother and Child Cohort Study (MoBa) conducted by the Norwegian Institute of Public Health. Enrollment for MoBa was from 1999 to 2008.
	**ATTRITION/EXCLUSION BIAS:** were outcome data incomplete due to attrition or exclusion from analysis?	Probably low	There is no evidence of exclusions that may bias the results reported in the manuscript.
	**SELECTIVE REPORTING BIAS:** were all measured outcomes reported?	Probably low	The authors stated: "our analysis suggests an inverse association between maternal plasma levels of PFCs and birth weight among Norwegian women". The outcome is unique and has been reported.
	**CONFLICT OF INTEREST**	Probably low	Authors declare that they have no conflicts of interests to disclose.
**Kusheng Wu**	**DETECTION BIAS [Key**	Probably low	The authors investigation included analyses of maternal serum samples, health effect examinations, and

(2012)	**element]:** can we be confident in the exposure characterization?		other relevant factors. Questionnaires were administered and maternal serum samples were collected for 167 pregnant women.
	**DETECTION BIAS [Key element]:** can we be confident in the outcome assessment?	Probably low	The authors obtained birth outcome information from medical records.
	**CONFOUNDING BIAS [Key element]:** did the study design or analysis account for important confounding and modifying variables?	Probably high	Adjust for maternal age, educational level, smoking, husband smoking, catching cold during pregnant, premature delivery history, and spontaneous abortion history, baby sex, gestational age.
	**SELECTION BIAS:** did selection of study participants result in appropriate comparison groups?	Definitely high	The study was a cross-sectional study.
	**ATTRITION/EXCLUSION BIAS:** were outcome data incomplete due to attrition or exclusion from analysis?	Probably low	There is no evidence of exclusion bias.

	**SELECTIVE REPORTING BIAS:** were all measured outcomes reported?	Probably low	The authors stated: "Mothers from Guiyu were exposed to higher levels of PFOA than those from control areas. Prenatal exposure to PFOA was associated with decreased neonatal physical development and adverse birth outcomes". So all outcomes have been reported.
	**CONFLICT OF INTEREST**	Probably high	The authors do not mention whether there is a financial conflict of interest.
**Lyndsey A. Darrow (2013)**	**DETECTION BIAS [Key element]:** can we be confident in the exposure characterization?	Definitely low	The authors stated: "Women provided serum for PFOA and PFOS measurement in 2005–2006 and reported reproductive histories in subsequent follow-up interviews".
	**DETECTION BIAS [Key element]:** can we be confident in the outcome assessment?	Probably low	The authors obtained birth outcome information from medical records.
	**CONFOUNDING BIAS [Key element]:** did the study design or analysis account for important confounding and modifying variables?	Probably high	maternal age, educational level (< 12 years, 12, 13–15, ≥ 16), smoking status (current, former, non), parity (0, ≥ 1), BMI (underweight, normal, overweight, obese), self-reported diabetes, time between conception and serum measurement (year strata), indicator variables for gestational week (37, 38, 39, 40, ≥ 41), parity.
	**SELECTION BIAS:** did selection of study participants result in appropriate comparison	Probably low	Participants who enrolled in the C8 Health Project between 2005 and 2006.

	groups?		
	**ATTRITION/EXCLUSION BIAS:** were outcome data incomplete due to attrition or exclusion from analysis?	Probably low	After exclusions, there were 1,630 singleton births included in the analyses. There is no evidence of exclusions that may bias the results reported in the manuscript.
	**SELECTIVE REPORTING BIAS:** were all measured outcomes reported?	Definitely low	The authors stated: "Results provide some evidence of positive associations between measured serum perfluorinated compounds and pregnancy-induced hypertension and a negative association between PFOS and birth weight among full-term infants". So all the outcomes have been reported.
	**CONFLICT OF INTEREST**	Probably low	The authors declare they have no actual or potential competing financial interests.
**Candace A. Robledo (2015)**	**DETECTION BIAS [Key element]:** can we be confident in the exposure characterization?	Probably low	The authors stated: "Biospecimens were collected from each partner during the baseline home visit. Approximately 20 mL of nonfasting blood were collected to measure concentrations of environmental chemicals".
	**DETECTION BIAS [Key element]:** can we be confident in the outcome assessment?	Definitely low	The authors stated: "Information recorded on the delivery cards included infant sex, birth weight (in grams or pounds and ounces) (n = 230), length (in centimeters or inches) (n = 229), and head circumference (in centimeters or inches) (n = 181)".
	**CONFOUNDING BIAS [Key element]:** did the study design or analysis account for important	Definitely high	Analysis adjusted for maternal and paternal serum lipids, serum cotinine, maternal pre-pregnancy BMI (kg/m2), maternal age, difference in parental age, infant sex, and the individual and partner sum of remaining chemical concentrations in each chemical's respective class

	confounding and modifying variables?		
	**SELECTION BIAS:** did selection of study participants result in appropriate comparison groups?	Definitely low	The Longitudinal Investigation of Fertility and the Environment (LIFE) Study was a prospective cohort study conducted between 2005 and 2009.
	**ATTRITION/EXCLUSION BIAS:** were outcome data incomplete due to attrition or exclusion from analysis?	Probably low	There is no evidence of exclusions that may bias the results reported in the manuscript.
	**SELECTIVE REPORTING BIAS:** were all measured outcomes reported?	Probably high	Pre-conceptional maternal and paternal concentrations of several POPs were associated with statistically significant differences in birth size among offspring.
	**CONFLICT OF INTEREST**	Probably low	The authors declare they have no actual or potential competing financial interests.
**Eman Alkhalawi (2016)**	**DETECTION BIAS [Key element]:** can we be confident in the exposure characterization?	Definitely low	The authors stated: "maternal blood was collected mainly at wk-32 pregnancy and cord blood at delivery. Separation of plasma occurred after centrifugation.".
	**DETECTION BIAS [Key**	Definitely	Comprehensive questionnaires were conducted on the first postnatal visit 2 wks after delivery.

	**element]:** can we be confident in the outcome assessment?	high	
	**CONFOUNDING BIAS [Key element]:** did the study design or analysis account for important confounding and modifying variables?	Probably high	Adjusted for birth by caesarean section (yes vs. no), pregnancy duration (wk), maternal BMI before pregnancy (kg/m2), maternal height (cm), lead in blood, log2-transformed (µg/L) (maternal blood), new born sex (male), mothers born in/outside Germany (yes), and smoking during pregnancy (yes).
	**SELECTION BIAS:** did selection of study participants result in appropriate comparison groups?	Probably low	The authors stated: "The "Duisburg Birth Cohort Study" was initiated in 2000 and endorsed by the North Rhine Westphalia State Environment Agency".
	**ATTRITION/EXCLUSION BIAS:** were outcome data incomplete due to attrition or exclusion from analysis?	Probably low	The authors stated that they exclude these population from the analysis. There is no evidence of exclusion bias.
	**SELECTIVE REPORTING BIAS:** were all measured outcomes reported?	Probably low	The authors stated: "PFOA, PFOS, and PFHxS were generally within German background exposurelevels. There was a significant association between PFOA, PFOS, and PFHxS concentration quartiles and decrease in ponderal index at birth but not weight or height. A nonsignificant negative

			association between exposure to all three compounds and birth weight was noted. Follow-up showed no sustained effect of the PFAA on anthropometric measures during the first year". So all the outcomes have been reported.
	**CONFLICT OF INTEREST**	Probably high	The authors do not mention whether there is a financial conflict of interest.
**Cathrine Carlsen Bach (2016)**	**DETECTION BIAS [Key element]:** can we be confident in the exposure characterization?	Probably low	The author measured the levels of 16 PFAAs in maternal serum.
	**DETECTION BIAS [Key element]:** can we be confident in the outcome assessment?	Probably low	The outcomes available in the cohort included birth weight (continuous and z-score), birth length, and head circumference at birth. z-Scores were calculated by standardization of birth weight for gestational age according to the most recent (1996) Scandinavian fetal reference.
	**CONFOUNDING BIAS [Key element]:** did the study design or analysis account for important confounding and modifying variables?	Probably high	Adjusted to maternal age, pre-pregnancy BMI, educational level and gestational age.
	**SELECTION BIAS:** did selection of study participants result in appropriate comparison	Definitely low	The author studied 1,507 mothers and their children from the Aarhus Birth Cohort (2008–2013).

	groups?		
	**ATTRITION/EXCLUSION BIAS:** were outcome data incomplete due to attrition or exclusion from analysis?	Definitely low	The levels of PFAAs were similar in samples taken before and after 13 gestational weeks (data not shown), and the exclusion of 67 participants with a blood sample drawn after 13 completed gestational weeks did not change the results (data not shown).
	**SELECTIVE REPORTING BIAS:** were all measured outcomes reported?	Probably low	The authors stated: "we did not find strong or consistent associations between PFAAs and birth weight or other indices of fetal growth, though estimated mean birth weights were lower among those with exposures above the lowest quartile for some compounds.", so all outcomes have been reported.
	**CONFLICT OF INTEREST**	Probably low	The authors declare they have no actual or potential competing financial interests.
**A.C. Callan (2016)**	**DETECTION BIAS [Key element]:** can we be confident in the exposure characterization?	Probably low	Ninety eight whole blood samples were available for PFAA analysis.
	**DETECTION BIAS [Key element]:** can we be confident in the outcome assessment?	Probably low	Participants were requested to check the birth outcomes recorded for their child by the medical team at delivery when completing their questionnaire.
	**CONFOUNDING BIAS [Key element]:** did the study design or analysis account for important	Probably low	Analysis were adjusted to maternal height, gestational age, pre-pregnancy BMI, weight gain during pregnancy (%) and sex of infant.

	confounding and modifying variables?		
	**SELECTION BIAS:** did selection of study participants result in appropriate comparison groups?	Definitely high	They were collected in the AMETS study, a cross-sectional study conducted between 2008 and 2011.
	**ATTRITION/EXCLUSION BIAS:** were outcome data incomplete due to attrition or exclusion from analysis?	Probably low	There is no evidence of exclusions that may bias the results reported in the manuscript.
	**SELECTIVE REPORTING BIAS:** were all measured outcomes reported?	Probably low	The authors stated: "Despite the low concentrations of PFAAs measured in maternal whole blood, relationships were observed between concentrations and birth weight.", so all outcomes have been reported.
	**CONFLICT OF INTEREST**	Probably high	The authors do not mention whether there is a financial conflict of interest.
**Eun Jin Kwon (2016)**	**DETECTION BIAS [Key element]:** can we be confident in the exposure characterization?	Probably low	We analyzed the level of eight PFCs in cord blood and two genetic polymorphisms in maternal blood of 268 subjects.

	**DETECTION BIAS [Key element]:** can we be confident in the outcome assessment?	Probably low	Birth weight and length were obtained from medical records at birth.
	**CONFOUNDING BIAS [Key element]:** did the study design or analysis account for important confounding and modifying variables?	Probably low	Analysis adjusted for parity, gestational age, mother's age, pre-pregnancy BMI, past history of alcohol consumption and child's gender.
	**SELECTION BIAS:** did selection of study participants result in appropriate comparison groups?	Definitely low	The authors stated: "We conducted the Ewha Birth & Growth Retrospective Cohort (EBGRC) study, which included blood samples and birth outcome data from the Ewha Birth & Growth Cohort study conducted at Ewha Womans University Hospital, Seoul, Korea,<br>between 2006 and 2010". Thus, selection bias may be ruled out.
	**ATTRITION/EXCLUSION BIAS:** were outcome data incomplete due to attrition or exclusion from analysis?	Probably low	There is no evidence of exclusions that may bias the results reported in the manuscript. The cross-sectional design prevents the loss of follow-up of participants.
	**SELECTIVE REPORTING BIAS:** were all measured	Probably low	The authors stated: "Our findings indicated that GSTM1 polymorphism might affect the association between exposure to PFCs and birth weight, suggesting the effect of genetic susceptibility on the

	outcomes reported?		relationship between prenatal PFCs exposure and birth outcomes". So, all the outcomes have been reported.
	**CONFLICT OF INTEREST**	Probably low	The authors declare no conflicts of interest.
**Eung-Sun Lee (2016)**	**DETECTION BIAS [Key element]:** can we be confident in the exposure characterization?	Probably low	The cord serum samples were extracted using a modifiedion-pairing extraction that has been described previously.
	**DETECTION BIAS [Key element]:** can we be confident in the outcome assessment?	Definitely low	We obtained information on the mothers and infants, including birth weight, gestational age, infant gender, maternal age at birth, and their medical attendants, from their medical records.
	**CONFOUNDING BIAS [Key element]:** did the study design or analysis account for important confounding and modifying variables?	Probably high	Analysis adjusted for maternal age, gestational age, infant gender, and clinician A.
	**SELECTION BIAS:** did selection of study participants result in appropriate comparison groups?	Definitely high	The author conducted a cross-sectional survey of newborns at the Cheil Woman's Hospital in Seoul, South Korea, between June and November 2008.

	**ATTRITION/EXCLUSION BIAS:** were outcome data incomplete due to attrition or exclusion from analysis?	Probably low	There is no evidence of exclusion bias.
	**SELECTIVE REPORTING BIAS:** were all measured outcomes reported?	Probably low	The authors stated: "Our results indicate that in utero exposure to relatively low levels of PFOS was negatively correlated with birth weight.". So all the outcomes have been reported.
	**CONFLICT OF INTEREST**	Probably low	The authors declare they have no competing financial interests.
**Virissa Lenters (2016)**	**DETECTION BIAS [Key element]:** can we be confident in the exposure characterization?	Probably low	PFASs and phthalates, along with cotinine and vitamin D, were simultaneously determined in 100-µL aliquots of serum by liquid chromatography–tandem mass spectrometry, following an optimized protocol based on Lindh et al.
	**DETECTION BIAS [Key element]:** can we be confident in the outcome assessment?	Definitely low	Birth outcome data were extracted from hospital maternity records by medical personnel.
	**CONFOUNDING BIAS [Key element]:** did the study design or analysis account for important confounding and modifying	Definitely high	Analysis adjusted for total lipids: –134.22 (95% CI: 191.43, –77.02), –99.91 (95% CI: –153.30, –46.52), –67.16 (95% CI: –119.80, –14.51).

	variables?		
	**SELECTION BIAS:** did selection of study participants result in appropriate comparison groups?	Probably low	Pregnant women were enrolled between June 2002 and May 2004 during routine antenatal care visits at a) local hospitals or clinics in 19 municipalities and settlements throughout Greenland, b) three hospitals and eight antenatal clinics in Kharkiv, Ukraine, and c) a large central hospital in Warsaw, Poland.
	**ATTRITION/EXCLUSION BIAS:** were outcome data incomplete due to attrition or exclusion from analysis?	Probably low	There is no evidence of exclusion bias.
	**SELECTIVE REPORTING BIAS:** were all measured outcomes reported?	Probably low	The authors stated: "This study suggests that several of the environmental contaminants, belonging to three chemical classes, may be independently associated with impaired fetal growth. These results warrant follow-up in other cohorts".
	**CONFLICT OF INTEREST**	Probably low	The authors declare they have no actual or potential competing financial interests.
**Yan Wang (2016)**	**DETECTION BIAS [Key element]:** can we be confident in the exposure characterization?	Definitely low	For 223 Taiwanese mothers and their term infants, the author measured PFOA and four long-chain PFCAs (ng/mL) in third-trimester maternal serum.
	**DETECTION BIAS [Key element]:** can we be confident in	Probably low	Birth weight (kg), length (cm), and head circumference (cm) were measured soon after delivery by nurses in the clinics.

	the outcome assessment?		
	**CONFOUNDING BIAS [Key element]:** did the study design or analysis account for important confounding and modifying variables?	Probably high	Family annual income, maternal age at delivery, maternal education, maternal previous live children, and maternal pre-pregnancy BMI.
	**SELECTION BIAS:** did selection of study participants result in appropriate comparison groups?	Probably low	The Taiwan Maternal and Infant Cohort Study is a longitudinal cohort study designed to examine prenatal environmental exposures and growth and development in infants and children.
	**ATTRITION/EXCLUSION BIAS:** were outcome data incomplete due to attrition or exclusion from analysis?	Probably low	There is no evidence of exclusion bias.
	**SELECTIVE REPORTING BIAS:** were all measured outcomes reported?	Probably low	The authors stated: "Prenatal exposure to long-chain PFCAs may interfere with fetal and childhood growth in girls, and childhood growth in boys".
	**CONFLICT OF INTEREST**	Probably low	The authors declare they have no actual or potential competing financial interests.

**Jillian Ashley-Martin (2017)**	**DETECTION BIAS [Key element]:** can we be confident in the exposure characterization?	Probably low	Three PFAS (PFOA, PFOS, and PFHxS) were measured in first trimester plasma using ultra-high-pressure liquid chromatography (ACQUITY UPLC System.
	**DETECTION BIAS [Key element]:** can we be confident in the outcome assessment?	probably low	Birth weight was recorded in each study participant's chart and extracted for inclusion in the MIREC database.
	**CONFOUNDING BIAS [Key element]:** did the study design or analysis account for important confounding and modifying variables?	Definitely high	Adjust for maternal age, pre-pregnancy body mass index, household income, and smoking.
	**SELECTION BIAS:** did selection of study participants result in appropriate comparison groups?	probably low	Data and biospecimens were obtained from the Maternal Infant Research on Environmental Chemicals (MIREC) Study, a trans-Canada cohort study of 2,001 pregnant women.
	**ATTRITION/EXCLUSION BIAS:** were outcome data	Definitely low	The authors stated: "We observed that parameter estimates did not change when we used the gestational-age-specific z scores, probably because of the exclusion of preterm births".

	incomplete due to attrition or exclusion from analysis?		
	**SELECTIVE REPORTING BIAS:** were all measured outcomes reported?	Probably low	The relationship between PFOA and birth weight was consistently inverse in both the Bayesian and frequentist models and regardless of whether the outcome was birth weight z score or birth weight. So all the outcomes have been reported.
	**CONFLICT OF INTEREST**	Probably low	The authors declare they have no actual or potential competing financial interests.
**Mei-Huei Chen (2017)**	**DETECTION BIAS [Key element]:** can we be confident in the exposure characterization?	Probably low	PFASs in umbilical cord blood were analyzed by ultra-high-performance liquid chromatography/tandem mass spectrometry.
	**DETECTION BIAS [Key element]:** can we be confident in the outcome assessment?	Probably low	These children were followed serially and growth data were collected through face to face interviews and records in Child Healthcare Handbooks until 108 months of age.
	**CONFOUNDING BIAS [Key element]:** did the study design or analysis account for important confounding and modifying variables?	Probably high	Maternal age, pregnancy body mass index, education level, log (Ln) transformed cord blood continent levels, infant sex and preterm birth.
	**SELECTION BIAS:** did	Definitely	The study subjects were from the Taiwan Birth Panel Study (TBPS)(Hsieh et al., 2011), a longitudinal

	selection of study participants result in appropriate comparison groups?	low	birth cohort study that was conducted at one medical center in Taipei and one local hospital and two clinics in New Taipei from April 2004 to January 2005.
	**ATTRITION/EXCLUSION BIAS:** were outcome data incomplete due to attrition or exclusion from analysis?	Probably low	There is no evidence of exclusions that may bias the results reported in the manuscript. The cross-sectional design prevents the loss of follow-up of participants.
	**SELECTIVE REPORTING BIAS:** were all measured outcomes reported?	Probably low	The authors stated: "we observed a negative association between PFOS levels in cord blood plasma and fetal growth in consideration of potential confounders". So all the outcomes haven been reported.
	**CONFLICT OF INTEREST**	Definitely low	The authors declare that they have no actual or potential conflict of interest including any financial, personal or other relationship with other people or organizations that could inappropriately influence, or be perceived to influence, their work.
**Hilde B. Lauritzen (2017)**	**DETECTION BIAS [Key element]:** can we be confident in the exposure characterization?	Probably low	Serum samples were collected in second trimester (gestational week 17–20) in the SGA study (1986–1988) and serum was stored at −80° C for later analysis.
	**DETECTION BIAS [Key element]:** can we be confident in	Definitely low	Indices of fetal growth including birth weight (continuous; grams (g)), birth length (continuous; centimeters (cm)) and head circumference (continuous; cm) were measured and recorded at birth.

	the outcome assessment?		
	**CONFOUNDING BIAS [Key element]:** did the study design or analysis account for important confounding and modifying variables?	Probably high	maternal age (years), height (cm), pre-pregnancy BMI (kg/m2), education (<9, 9–12, >12 y), smoking status at conception (0, 1–9, >10 cig/d), interpregnancy interval (<18, 19–60, >60 mo) and offspring sex (male/female).
	**SELECTION BIAS:** did selection of study participants result in appropriate comparison groups?	Probably high	In the current study, we used a case–cohort design to study the association between maternal serum levels of PFASs and OCs and indices of fetal growth including birth weight, birth length, head circumference, and SGA birth in Scandinavian women from Norway and Sweden.
	**ATTRITION/EXCLUSION BIAS:** were outcome data incomplete due to attrition or exclusion from analysis?	Probably low	There is no evidence of exclusions that may bias the results reported in the manuscript.
	**SELECTIVE REPORTING BIAS:** were all measured outcomes reported?	Probably low	The authors stated: "Some populations may be more vulnerable to EDCs, possibly due to differences in exposure levels, exposure sources and/or modifiable lifestyle factors. Male offspring may be more vulnerable to endocrine disruption ". The outcome is unique and has been reported.
	**CONFLICT OF INTEREST**	Probably low	Authors declare that they have no conflicts of interests to disclose.

**Meng Li (2017)**	**DETECTION BIAS [Key element]:** can we be confident in the exposure characterization?	Probably low	Cord blood samples were collected immediately after delivery and centrifuged within 3 h. Next, cord serum was kept at −80 °C in 2-mL methanolrinsed polypropylene cryovials.
	**DETECTION BIAS [Key element]:** can we be confident in the outcome assessment?	Probably low	Outcomes examined in the present study (obtained from medical records) included gestational age (weeks), birth weight (grams), preterm birth, low birth weight (LBW).
	**CONFOUNDING BIAS [Key element]:** did the study design or analysis account for important confounding and modifying variables?	Probably low	Adjust for delivery, education, infant sex, maternal age, PIH, GDM, anemia, birth weight, parity, gestational age.
	**SELECTION BIAS:** did selection of study participants result in appropriate comparison groups?	Definitely high	This study is a cross-sectional study.
	**ATTRITION/EXCLUSION BIAS:** were outcome data	Probably low	There is no evidence of exclusion bias.

	incomplete due to attrition or exclusion from analysis?		
	**SELECTIVE REPORTING BIAS:** were all measured outcomes reported?	Probably low	The authors stated: "in cord serum and weight at birth. We also found that the branched PFOS isomer compounds displayed the most robust associations to lower birth weight. Additionally, we observed that male infants may be more vulnerable than females to PFASs exposure". So all outcomes have been reported.
	**CONFLICT OF INTEREST**	Probably low	The authors have declared no existing conflicts of interest in completing this study.
**Cyntia B. Manzano-Salgado (2017)**	**DETECTION BIAS [Key element]:** can we be confident in the exposure characterization?	Probably low	Maternal blood samples were collected during the first trimester of pregnancy.
	**DETECTION BIAS [Key element]:** can we be confident in the outcome assessment?	Definitely low	Birth weight (grams) was measured by trained midwives at delivery.Birth length (cm) and head circumference (cm) were measured within the first 12 h-of-life by a nurse when the newborn arrived at the hospital ward.
	**CONFOUNDING BIAS [Key element]:** did the study design or analysis account for important confounding and modifying variables?	Probably high	Adjust for maternal age, pre-pregnancy BMI, parity, and fish intake during pregnancy.

	**SELECTION BIAS:** did selection of study participants result in appropriate comparison groups?	Probably low	In this study we used data from the INMA (Environment and Childhood - INfancia y Medio Ambiente) birth cohort.
	**ATTRITION/EXCLUSION BIAS:** were outcome data incomplete due to attrition or exclusion from analysis?	Probably low	There is no evidence of exclusions that may bias the results reported in the manuscript.
	**SELECTIVE REPORTING BIAS:** were all measured outcomes reported?	Probably low	The authors stated: "In this study, PFAS showed little association with birth outcomes. Higher PFHxS, PFOA, and PFNA concentrations were non-significantly associated with reduced birth weight". So all the outcomes have been reported.
	**CONFLICT OF INTEREST**	Probably low	The authors declare no conflict of interest.
**Machiko Minatoya (2017)**	**DETECTION BIAS [Key element]:** can we be confident in the exposure characterization?	Definitely low	PFOS and PFOA concentrations in maternal serum were measured by liquid chromatography-tandem mass spectrometry (LC/MS/MS).
	**DETECTION BIAS [Key element]:** can we be confident in the outcome assessment?	Probably low	The authors stated: "Maternal anthropometric measurement data and medical history were obtained from medical record and birth weight and length were collected from birth records ".

	**CONFOUNDING BIAS [Key element]:** did the study design or analysis account for important confounding and modifying variables?	Probably high	Analysis adjusted for maternal BMI, parity, gestational age smoking during pregnancy, blood sampling period and infant sex.
	**SELECTION BIAS:** did selection of study participants result in appropriate comparison groups?	Probably low	This prospective birth cohort study was based on the Sapporo Cohort, the Hokkaido Study on Environment and Children's Health.
	**ATTRITION/EXCLUSION BIAS:** were outcome data incomplete due to attrition or exclusion from analysis?	Probably low	There is no evidence of exclusions that may bias the results reported in the manuscript.
	**SELECTIVE REPORTING BIAS:** were all measured outcomes reported?	Probably low	Our findings provided some evidences of possible adverse effects of prenatal exposure to PFASs on metabolic function at birth and birth size.
	**CONFLICT OF INTEREST**	Probably low	The authors declare they have no actual or potential competing financial interests.

Yu Shi (2017)	**DETECTION BIAS [Key element]:** can we be confident in the exposure characterization?	Probably low	Cord serum samples were thawed at ambient temperature before extraction. PFASs were extracted from serum by ion-pairing method.
	**DETECTION BIAS [Key element]:** can we be confident in the outcome assessment?	Probably low	In this study, maternal age, maternal weight before pregnancy, height, infant gender, parity, birth weight (in grams), birth length (in centimeters), and gestational time (in days) were abstracted from medical records.
	**CONFOUNDING BIAS [Key element]:** did the study design or analysis account for important confounding and modifying variables?	Probably low	Adjusted for maternal age, pre-pregnancy BMI, parity, gestational age, fetus gender and maternal height.
	**SELECTION BIAS:** did selection of study participants result in appropriate comparison groups?	Definitely high	170 pregnant women were recruited from Haidian Maternal and Child Health Hospital in Beijing from Feb. 2012 to Jun. 2012 and this study is a cross-sectional study.
	**ATTRITION/EXCLUSION BIAS:** were outcome data	Probably low	There is no evidence of exclusion bias.

	incomplete due to attrition or exclusion from analysis?		
	**SELECTIVE REPORTING BIAS:** were all measured outcomes reported?	Probably low	The authors stated: "Although the associations between prenatal PFHxS, PFOS, PFOA, PFNA, PFDA or PFUnA and birth outcomes were not significant, the high detection rate of these chemicals in cord blood plasma suggest fetal exposure, and the possibility of other health effects still need further investigation. We suggest cautious interpretation of this study until the finding can be replicated in other populations". So all the outcomes have been reported.
	**CONFLICT OF INTEREST**	Probably high	The authors do not mention whether there is a financial conflict of interest
**Anne P. Starling (2017)**	**DETECTION BIAS [Key element]:** can we be confident in the exposure characterization?	Probably low	Within the Healthy Start prospective cohort, concentrations of 11 PFAS, fasting glucose, and lipids were measured in maternal mid-pregnancy serum.
	**DETECTION BIAS [Key element]:** can we be confident in the outcome assessment?	Probably low	Birth weight was measured by clinical personnel at birth using a calibrated scale.
	**CONFOUNDING BIAS [Key element]:** did the study design or analysis account for important	Definitely low	Adjusted for maternal age, parity, gestational age, pre-pregnancy body mass index (BMI), race/ethnicity, education, gestational weight gain, smoking during pregnancy, gravidity, gestational age at blood draw, infant sex.

	confounding and modifying variables?		
	**SELECTION BIAS:** did selection of study participants result in appropriate comparison groups?	Probably low	Healthy Start is a prospective cohort study that enrolled 1,410 pregnant women from obstetrics clinics at the University of Colorado Hospital from 2009–2014.
	**ATTRITION/EXCLUSION BIAS:** were outcome data incomplete due to attrition or exclusion from analysis?	Probably low	There is no evidence of exclusions that may bias the results reported in the manuscript.
	**SELECTIVE REPORTING BIAS:** were all measured outcomes reported?	Probably low	The authors stated: "Follow-up of offspring will determine the potential long-term consequences of lower weight and adiposity at birth associated with prenatal PFAS exposure ", so all outcomes have been reported.
	**CONFLICT OF INTEREST**	Probably low	The authors declare they have no actual or potential competing financial interests.
**Damaskini Valvi (2017)**	**DETECTION BIAS [Key element]:** can we be confident in the exposure characterization?	Probably low	OC concentrations in maternal serum were measured using gas chromatography with electron capture detection as the standard at the time.

	**DETECTION BIAS [Key element]:** can we be confident in the outcome assessment?	Probably low	Information about maternal age at delivery, gestational age and child sex was extracted from the obstetric and medical records.
	**CONFOUNDING BIAS [Key element]:** did the study design or analysis account for important confounding and modifying variables?	Probably high	maternal age at delivery, education, parity, pre-pregnancy BMI (continuous), smoking during pregnancy and child sex.
	**SELECTION BIAS:** did selection of study participants result in appropriate comparison groups?	Definitely low	The author used information from 604 of the mother-child pairs recruited at 34 weeks of gestation at the National Hospital in Torshavn in the Faroe Islands between 1997 and 2000.
	**ATTRITION/EXCLUSION BIAS:** were outcome data incomplete due to attrition or exclusion from analysis?	Probably low	The cross-sectional design prevents the loss of follow-up of participants.
	**SELECTIVE REPORTING BIAS:** were all measured	Probably low	The authors stated: "increases in offspring head circumference, while an indication of sex-dimorphic associations with birth weight and head circumference was found for PFAS exposures", so all

	outcomes reported?		outcomes have been reported.
	**CONFLICT OF INTEREST**	Probably low	The authors have no competing interests to declare, financial or otherwise.
**Wencheng Cao (2018)**	**DETECTION BIAS [Key element]:** can we be confident in the exposure characterization?	Probably low	Umbilical cord blood was collected to measure eleven PFASs by liquid chromatography-mass spectrometry.
	**DETECTION BIAS [Key element]:** can we be confident in the outcome assessment?	Definitely low	Measurements on gestational growth, including infants' gender, birth weight (g), birth length (cm), and ponderal index (PI), were extracted from the birth records in the hospital. Measurements on postnatal infant's data, included age (months), postnatal weight (g), postnatal length (cm), and postnatal head circumference (cm). Postnatal length and weight were measured by a nurse at the follow-up interview. PI was calculated by the following formula: PI = [weight (g) / length3(cm3)] × 100.
	**CONFOUNDING BIAS [Key element]:** did the study design or analysis account for important confounding and modifying variables?	Probably high	Adjusted for maternal age, parity, maternal education, household income, smoking of father, drinking of father, infant's gender.
	**SELECTION BIAS:** did selection of study participants result in appropriate comparison groups?	Probably low	We recruited the study population in a longitudinal birth cohort established from November 2013 to December 2015 in Zhoukou City, China.

	**ATTRITION/EXCLUSION BIAS:** were outcome data incomplete due to attrition or exclusion from analysis?	Probably low	There is no evidence of exclusions that may bias the results reported in the manuscript.
	**SELECTIVE REPORTING BIAS:** were all measured outcomes reported?	Probably low	The authors stated: "Higher PFOA concentrations in cord blood were connected with reduced gestational and postnatal growth. PFUdA were positively associated with indications of gestational growth and postnatal growth. Increased PFDoA and PFHxS concentrations were associated with decreased birth size, but increased postnatal growth (mean 19 month)". So, all the outcomes have been reported.
	**CONFLICT OF INTEREST**	Probably low	The authors declare they have no actual or potential competing financial interest.
**Qi Meng (2018)**	**DETECTION BIAS [Key element]:** can we be confident in the exposure characterization?	Probably low	All blood samples collected in the DNBC were sent by mail to Statens Serum Institute in Copenhagen, separated and stored in freezers at −20 °C or −80 °C.
	**DETECTION BIAS [Key element]:** can we be confident in the outcome assessment?	Probably low	The authors stated: "Birth weight (grams) and gestational age at birth were obtained from the National Hospital Discharge Register at the National Board of Health in Denmark".

	**CONFOUNDING BIAS [Key element]:** did the study design or analysis account for important confounding and modifying variables?	Probably low	Analysis adjusted for infant sex, infant birth year, parity, gestational week of blood draw, maternal age, socio-occupational status, pre-pregnancy body mass index (BMI), smoking and alcohol intake during pregnancy.
	**SELECTION BIAS:** did selection of study participants result in appropriate comparison groups?	Definitely low	A previous study in the Danish National Birth Cohort (DNBC).
	**ATTRITION/EXCLUSION BIAS:** were outcome data incomplete due to attrition or exclusion from analysis?	Probably low	There is no evidence of exclusions that may bias the results reported in the manuscript.
	**SELECTIVE REPORTING BIAS:** were all measured outcomes reported?	Probably low	The authors stated: "Our findings strengthen the evidence that in-utero PFASs exposures might affect fetal growth. Exposure levels of PFOS and PFOA are declining in some western countries but they are still widely detected". So all the outcomes have been reported.
	**CONFLICT OF INTEREST**	Probably low	The authors declare they have no competing financial interests.
**Lisa B. Rokoff**	**DETECTION BIAS [Key**	Probably low	The author also measured maternal plasma concentrations of several PFASs.

(2018)	**element]:** can we be confident in the exposure characterization?		
	**DETECTION BIAS [Key element]:** can we be confident in the outcome assessment?	Probably low	The author abstracted birth weight (in grams) and date of delivery from the hospital medical record.
	**CONFOUNDING BIAS [Key element]:** did the study design or analysis account for important confounding and modifying variables?	Probably high	Analysis adjusted for maternal age, parity, race/ethnicity, education, pre-pregnancy body mass index.
	**SELECTION BIAS:** did selection of study participants result in appropriate comparison groups?	Probably low	Between 1999 and 2002, we recruited pregnant women into the Project Viva cohort during their initial prenatal visit (median 9.9 weeks gestation) at Atrius Harvard Vanguard Medical Associates, a multi-specialty group practice with offices throughout eastern Massachusetts.
	**ATTRITION/EXCLUSION BIAS:** were outcome data incomplete due to attrition or exclusion from analysis?	Probably low	There is no evidence of exclusion bias.

	**SELECTIVE REPORTING BIAS:** were all measured outcomes reported?	Probably low	The authors stated: "Concurrent prenatal exposures to maternal smoking, black carbon, and PFOS are additively associated with lower fetal growth, whereas PFNA may attenuate associations of smoking and black carbon with lower fetal growth. It is important to examine interactions between multiple exposures in relation to health outcomes, as effects may not always be additive and may shed light on biological pathways". So all the outcomes have been reported.
	**CONFLICT OF INTEREST**	Probably low	The authors declare that they have no competing interests.
**Sharon K. Sagiv* (2018)**	**DETECTION BIAS [Key element]:** can we be confident in the exposure characterization?	Definitely low	The author measured plasma concentrations of 4 PFAS in early pregnancy (median length of gestation, 9 weeks) among 1,645 women in Project Viva.
	**DETECTION BIAS [Key element]:** can we be confident in the outcome assessment?	Probably low	Project Viva staff abstracted birth weight (in grams) from hospital medical records.
	**CONFOUNDING BIAS [Key element]:** did the study design or analysis account for important confounding and modifying variables?	Definitely low	Adjusted for maternal age at enrollment, parity, gestational age, race/ethnicity, education, prenatal smoking, history of breastfeeding, pre-pregnancy body mass index, paternal education, household income, child's sex
	**SELECTION BIAS:** did selection of study participants	Probably low	Project Viva is a prospective prebirth cohort study in which mothers were recruited between 1999 and 2002 at their first prenatal visit to one of 8 obstetrical clinics of Atrius Harvard Vanguard Medical

	result in appropriate comparison groups?		Associates, a multispecialty group practice ineastern Massachusetts
	**ATTRITION/EXCLUSION BIAS:** were outcome data incomplete due to attrition or exclusion from analysis?	Probably low	There is no evidence of exclusion bias.
	**SELECTIVE REPORTING BIAS:** were all measured outcomes reported?	Probably low	The authors stated: "our results do suggest that studies that examine associations of birth outcomes with PFAS in serum/plasma drawn early in pregnancy are unlikely to be substantially confounded by pregnancy hemodynamics" .
	**CONFLICT OF INTEREST**	Probably low	The authors have declared that no competing interests exist.
**Jessica Shoaff**	**DETECTION BIAS [Key element]:** can we be confident in the exposure characterization?	Probably low	The author quantified perfluorooctanoic acid (PFOA), perfluorooctane sulfonic acid (PFOS), perfluorononanoic acid (PFNA), and perfluorohexane sulfonic acid (PFHxS) in pregnant women's serum.
	**DETECTION BIAS [Key element]:** can we be confident in the outcome assessment?	Probably low	The author abstracted neonatal weight (grams) and gestational age (weeks) from hospital records.
	**CONFOUNDING BIAS [Key element]:** did the study design or	Probably	Adjusted for maternal age at delivery, parity, race, marital status, insurance, income, education, serum cotinine, depressive symptoms, mid-pregnancy BMI, food security, fruit/vegetable and fish

	analysis account for important confounding and modifying variables?	high	consumption during pregnancy, and prenatal vitamin use.
	**SELECTION BIAS:** did selection of study participants result in appropriate comparison groups?	Definitely low	The authors stated: "We analyzed data collected from an ongoing prospective pregnancy and birth cohort, the Health Outcomes and Measures of the Environment (HOME) study".
	**ATTRITION/EXCLUSION BIAS:** were outcome data incomplete due to attrition or exclusion from analysis?	Probably low	There is no evidence of exclusion bias.
	**SELECTIVE REPORTING BIAS:** were all measured outcomes reported?	Probably low	The author observed inverse associations between prenatal serum PFAS concentrations and anthropometry until 2 years of age. Prenatal serum PFAS concentrations were not associated with growth rate in the first 2 years of life.
	**CONFLICT OF INTEREST**	Probably low	The authors declare that they have no conflicts of interest with regard to the content of this report.
**Christian Bjerregaard-Olesen (2019)**	**DETECTION BIAS [Key element]:** can we be confident in the exposure characterization?	Probably low	The author extracted the actual mixture of PFAAs from the serum of 702 Danish pregnant women (gestational wk 11–13) enrolled in the Aarhus Birth Cohort (ABC) using solid phase extraction, high-performance liquid chromatography (HPLC), and weak anion exchange.

**DETECTION BIAS [Key element]:** can we be confident in the outcome assessment?	Probably low	The attending midwives recorded information on the pregnancy outcomes immediately after birth using a structured registration form.
**CONFOUNDING BIAS [Key element]:** did the study design or analysis account for important confounding and modifying variables?	Probably high	Maternal age, gestational age, body mass index (BMI), educational level, smoking, and alcohol intake, and sensitivity analyses.
**SELECTION BIAS:** did selection of study participants result in appropriate comparison groups?	Probably low	The present study is a part of the FETOTOX project, which includes pregnant women from five birth cohorts.
**ATTRITION/EXCLUSION BIAS:** were outcome data incomplete due to attrition or exclusion from analysis?	Definitely low	Due to missing data, a few participants were excluded from the analyses involving BW (n= 8), birth length (n= 9), and head circumference (n= 12). One implausible combination of BW and GA was identified.

	**SELECTIVE REPORTING BIAS:** were all measured outcomes reported?	Probably low	The authors stated: "Higher-serum PFAA-induced xenoestrogenic activities were associated with lower BW and length in offspring, suggesting that PFAA mixtures may affect fetal growth by disrupting ER function". So all the outcomes haven been reported.
	**CONFLICT OF INTEREST**	Probably low	The authors declare that they have no conflicts of interest with regard to the content of this report.
**Geetika Kalloo （2019）**	**DETECTION BIAS [Key element]:** can we be confident in the exposure characterization?	Probably low	The author measured exposure to environmental chemicals, which included both chemical compounds and metals, during pregnancy using biomarker concentrations of > 100 analytes in urine, serum, or blood at 16 or 26 weeks of gestation.
	**DETECTION BIAS [Key element]:** can we be confident in the outcome assessment?	Probably low	The extracted birth weight, length, head circumference, gestational age, and the method for determining gestational age (last menstrual period, n = 368; antenatal ultrasound, n = 6; or Ballard Maturational Assessment, n = 3) from newborn medical charts.
	**CONFOUNDING BIAS [Key element]:** did the study design or analysis account for important confounding and modifying variables?	Probably low	Maternal age, race, fresh fruit and vegetable consumption, parity, gestational age income, education, marital status and prenatal vitamin use, BMI.
	**SELECTION BIAS:** did selection of study participants result in appropriate comparison groups?	Definitely low	This study was based on HOME Study, a prospective pregnancy and birth cohort in the United States.

	**ATTRITION/EXCLUSION BIAS:** were outcome data incomplete due to attrition or exclusion from analysis?	Probably low	There is no evidence of exclusions that may bias the results reported in the manuscript.
	**SELECTIVE REPORTING BIAS:** were all measured outcomes reported?	Probably low	The authors stated: "All three methods of characterizing multiple chemical exposures in this cohort identified inverse associations of select organochlorine compounds, phenols, and cadmium with birth length, but not other neonatal outcomes". The outcome is unique and has been reported.
	**CONFLICT OF INTEREST**	Probably high	The authors declare the following financial interests/personal relationships which may be considered as potential competing interests: Joseph M. Braun was financially compensated for serving as an expert witness for plaintiffs in litigation related to tobacco smoke exposures.
**Kristin J. Marks (2019)**	**DETECTION BIAS [Key element]:** can we be confident in the exposure characterization?	Definitely low	Maternal serum samples were held in storage facilities at the University of Bristol until they were transferred under controlled conditions to the National Center for Environmental Health (NCEH) of the Centers for Disease Control and Prevention (CDC) in the United States for analysis in 2015.
	**DETECTION BIAS [Key element]:** can we be confident in the outcome assessment?	Probably low	Birth weight (g) was abstracted from infant medical records.
	**CONFOUNDING BIAS [Key element]:** did the study design or	Probably high	Adjust for maternal age, parity, maternal pre-pregnancy BMI, folic acid use, smoking alcohol use during pregnancy.

	analysis account for important confounding and modifying variables?		
	**SELECTION BIAS:** did selection of study participants result in appropriate comparison groups?	Probably low	ALSPAC recruited pregnant women with expected delivery dates between April 1st, 1991 and December 31st, 1992.
	**ATTRITION/EXCLUSION BIAS:** were outcome data incomplete due to attrition or exclusion from analysis?	Probably low	There is no evidence of exclusion bias.
	**SELECTIVE REPORTING BIAS:** were all measured outcomes reported?	Probably low	The authors stated: "British boys born to mothers with higher serum concentrations of PFOS during pregnancy appear to weigh less, have a shorter crown to heel length, and a smaller head circumference at birth. Other PFAS under study also showed some evidence of inverse associations with birth size outcomes. ". So all outcomes have been reported.
	**CONFLICT OF INTEREST**	Probably low	The authors declare they have no actual or potential competing financial interests.
**Hexing Wang (2019)**	**DETECTION BIAS [Key element]:** can we be confident in	Probably low	Two typical PFASs, perfluorooctyl sulfonic acid (PFOS) and perfluorooctanoic acid (PFOA), and three typical estrogens, estrone (E1), b-estradiol (E2), and estriol (E3), were measured in cord serum.

	the exposure characterization?		
	**DETECTION BIAS [Key element]:** can we be confident in the outcome assessment?	Probably low	Body weight (g), body length (mm), and head circumference (mm) of infants at birth were measured by obstetric nurses to be nearest 1 g, 1 mm, and 1 mm, respectively.
	**CONFOUNDING BIAS [Key element]:** did the study design or analysis account for important confounding and modifying variables?	Definitely low	Adjusted for pregnant age, parity, gestational age, family income, maternal education level, maternal career, husband's smoking, energy daily intake, daily physical activity, pre-pregnant maternal body mass index, gestational diabetes mellitus, infant sex, delivery mode, and gestational weight gain;
	**SELECTION BIAS:** did selection of study participants result in appropriate comparison groups?	Definitely high	This study is a cross-sectional study.
	**ATTRITION/EXCLUSION BIAS:** were outcome data incomplete due to attrition or exclusion from analysis?	Probably low	There is no evidence of exclusions that may bias the results reported in the manuscript.

	**SELECTIVE REPORTING BIAS:** were all measured outcomes reported?	Probably low	The authors stated: "Serum PFOS and PFOA in cord were found to be differentially related to serum estrogens and reversely related to some birth size indices. Serum estrogens were related to birth size and E3 mediated the effect of serum PFOS on body weight". So all the outcomes have been reported.
	**CONFLICT OF INTEREST**	Probably low	The authors declare they have no actual or potential competing financial interests.
**Chenye Xu (2019)**	**DETECTION BIAS [Key element]:** can we be confident in the exposure characterization?	Probably low	The authors stated: "Descriptive statistical analyses were employed to characterize PFASs in the cord serum samples".
	**DETECTION BIAS [Key element]:** can we be confident in the outcome assessment?	Definitely low	Birth weight (kg), length (cm), ponderal index (birth weight in grams divided by length in cubic centimeters g cm-3) and head circumference (cm) were extracted from birth records at hospital.
	**CONFOUNDING BIAS [Key element]:** did the study design or analysis account for important confounding and modifying variables?	Probably low	Analysis adjusted for maternal age, maternal BMI, pregnancy weight gain. education, job, abortion times, times, birth gender and drinking water.
	**SELECTION BIAS:** did selection of study participants result in appropriate comparison groups?	Probably low	A total of 110 maternal-neonatal pairs were recruited from September 2016 to August 2017 at the Women's Hospital of the School of Medicine of Zhejiang University in Hangzhou, China.

	**ATTRITION/EXCLUSION BIAS:** were outcome data incomplete due to attrition or exclusion from analysis?	Probably low	There is no evidence of exclusions that may bias the results reported in the manuscript.
	**SELECTIVE REPORTING BIAS:** were all measured outcomes reported?	Probably high	Prenatal exposure to PFOS appeared to negatively affect birth weight and ponderal index.
	**CONFLICT OF INTEREST**	Probably low	The authors declare they have no actual or potential competing financial interests.
**Stephanie M. Eick (2020)**	**DETECTION BIAS [Key element]:** can we be confident in the exposure characterization?	Probably low	PFAS and PBDEs were measured in serum obtained during the second trimester of pregnancy.
	**DETECTION BIAS [Key element]:** can we be confident in the outcome assessment?	Probably low	Participants' medicals records were abstracted by trained study staff for the following measures: final gestational age at delivery in weeks, birth weight in grams, infant length, and infant head circumference.
	**CONFOUNDING BIAS [Key element]:** did the study design or analysis account for important confounding and modifying	Probably high	Models adjusted for maternal age, parity maternal race/ethnicity, pre-pregnancy BMI, maternal education, smoking status.

	variables?		
	**SELECTION BIAS:** did selection of study participants result in appropriate comparison groups?	Definitely low	Women included in the analysis were enrolled in the Chemicals in our Bodies (CIOB) study, a prospective birth cohort.
	**ATTRITION/EXCLUSION BIAS:** were outcome data incomplete due to attrition or exclusion from analysis?	Probably low	The authors stated that they exclude these population from the analysis. There is no evidence of exclusion bias.
	**SELECTIVE REPORTING BIAS:** were all measured outcomes reported?	Probably low	The authors stated: "certain PFAS were slightly associated with PTB".
	**CONFLICT OF INTEREST**	Probably low	The authors declare that they have no competing interests.
**Marie Harthøj Hjermitslev (2020)**	**DETECTION BIAS [Key element]:** can we be confident in the exposure characterization?	Probably low	Serum blood samples were analysed for 11 OCPs, 14 PCBs and 10PBDEs at Le Centre de Toxicologie du Québec in Canada and for 16 PFASs at the Department of Environmental Science, Aarhus University in Denmark.
	**DETECTION BIAS [Key element]:** can we be confident in	Probably low	The outcome data of the new-borns were carried out by midwifes, and the research group obtained the data from the Greenlandic Doctors Office.

	the outcome assessment?		
	**CONFOUNDING BIAS [Key element]:** did the study design or analysis account for important confounding and modifying variables?	Probably low	Adjusting for mother age, parity, gestational age, pre-pregnancy BMI, the smoking biomarker cotinine and consumption of alcohol during pregnancy.
	**SELECTION BIAS:** did selection of study participants result in appropriate comparison groups?	Definitely high	The present study was conducted based on a cross-sectional study. Recruitment of the pregnant women for the mother-child cohort occurred between August 2010 and August 2011 and between June 2013 and September 2015.
	**ATTRITION/EXCLUSION BIAS:** were outcome data incomplete due to attrition or exclusion from analysis?	Probably low	There is no evidence of exclusions that may bias the results reported in the m anuscript.
	**SELECTIVE REPORTING BIAS:** were all measured outcomes reported?	Probably low	The authors stated: "We found significant inverse associations between PFOA and birth outcome indices, which were only significant among female offspring after stratification for gender", so all outcomes have been reported.
	**CONFLICT OF INTEREST**	Probably low	The authors do not mention whether there is a conflict of interest.

**Xiaona Huo (2020)**	**DETECTION BIAS [Key element]:** can we be confident in the exposure characterization?	Probably low	Ten PFAS in maternal plasma in early pregnancy (gestational age, median (interquartile range): 15 (13–16) weeks) were measured.
	**DETECTION BIAS [Key element]:** can we be confident in the outcome assessment?	Definitely low	Clinically relevant characteristics of the current pregnancy such as pregnancy complications, chronic diseases during current pregnancy, number of fetus, infant sex and gestational age at birth were extracted from medical records.
	**CONFOUNDING BIAS [Key element]:** did the study design or analysis account for important confounding and modifying variables?	Probably low	Adjusted for maternal age (years), parity, gestational age, pre-pregnancy BMI (kg/m2), parental educational levels (≤ 12, > 12 years), pregnancy complicating with chronic diseases (no, yes), infant sex (male, female).
	**SELECTION BIAS:** did selection of study participants result in appropriate comparison groups?	Probably low	The study subjects in the current analysis were from the Shanghai Birth Cohort, a large prospective study in Shanghai, China, from 2013 to 2016.
	**ATTRITION/EXCLUSION BIAS:** were outcome data	Probably low	There is no evidence of exclusions that may bias the results reported in the manuscript.

	incomplete due to attrition or exclusion from analysis?		
	**SELECTIVE REPORTING BIAS:** were all measured outcomes reported?	Probably low	The authors stated: "In this prospective cohort study, we did not observe significant associations between maternal plasma PFAS concentrations in early pregnancy and gestational length, overall PTB, spontaneous or indicated PTB", so all outcomes have been reported.
	**CONFLICT OF INTEREST**	Probably low	The authors declare that they have no competing interests.
**Ikuko Kashino**	**DETECTION BIAS [Key element]:** can we be confident in the exposure characterization?	Probably low	The author analyzed concentrations of 11 PFAS [PFHxS, perfluorohexanoic acid (PFHxA), perfluoroheptanoic acid (PFHpA), PFOS, PFOA, PFNA, PFDA, PFUnDA, perfluorododecanoic acid (PFDoDA), PFTrDA, and perfluorotetradecanoic acid (PFTeDA)] in maternal plasma samples.
	**DETECTION BIAS [Key element]:** can we be confident in the outcome assessment?	Definitely low	Maternal and infant characteristics were compiled from the baseline questionnaire that was self-administered during the first trimester of pregnancy, medical records from hospitals, maternal plasma, and the questionnaire administered at 4 months post-delivery.
	**CONFOUNDING BIAS [Key element]:** did the study design or analysis account for important confounding and modifying variables?	Probably low	Maternal age, pre-pregnancy BMI, parity, gestational ag, infant sex, maternal educational level, and plasma cotinine concentration during pregnancy.
	**SELECTION BIAS:** did	Definitely	The study design was a prospective cohort study in Hokkaido, Japan. The study is a part of an ongoing

	selection of study participants result in appropriate comparison groups?	low	birth cohort study: the Hokkaido Study on Environment and Children's Health, initiated in February 2003.
	**ATTRITION/EXCLUSION BIAS:** were outcome data incomplete due to attrition or exclusion from analysis?	Probably low	There is no evidence of exclusions that may bias the results reported in the manuscript. The cross-sectional design prevents the loss of follow-up of participants.
	**SELECTIVE REPORTING BIAS:** were all measured outcomes reported?	Probably low	The authors stated: "Our findings suggest that prenatal, maternal exposure to PFAS with longer carbon chains tends to be inversely associated with birth size of newborn infants, which may indicate that these commercially used compounds have an adverse effect on fetal growth". So, all the outcomes have been reported.
	**CONFLICT OF INTEREST**	Probably low	The authors declare that they have no known competing financial interests or personal relationships that could have appeared to influence the work reported in this paper.
**Xiaotu Liu (2020)**	**DETECTION BIAS [Key element]:** can we be confident in the exposure characterization?	Probably low	Approximately 100 □ L of plasma was spiked with isotopically labeled standards and 133 vortexed.
	**DETECTION BIAS [Key element]:** can we be confident in	Probably low	Follow-up questionnaires about prenatal care, 105 maternal and neonatal complications, and outcomes were recorded in a maternal health care 106 booklet by healthcare staff.

	the outcome assessment?		
	**CONFOUNDING BIAS [Key element]:** did the study design or analysis account for important confounding and modifying variables?	Probably low	Sampling time, pre-pregnancy BMI, parity, gestational age occupation, gravidity, spontaneous abortion history, maternal age,child 636 gender, folic acid use, passive smoking, fasting status and medication use.
	**SELECTION BIAS:** did selection of study participants result in appropriate comparison groups?	Probably high	This nested case-control study was conducted in a prospective 100 cohort in Shanxi Province, China, between December 2009 to December 2013.
	**ATTRITION/EXCLUSION BIAS:** were outcome data incomplete due to attrition or exclusion from analysis?	Probably low	There is no evidence of exclusion bias.
	**SELECTIVE REPORTING BIAS:** were all measured outcomes reported?	Probably low	The authors stated: "Our study demonstrated that maternal exposure to low 381 levels of PFASs (i.e., plasma PFOA and PFOS below 2 ng/mL) unlikely elevate the SPB risk 382 in pregnant women ". So all the outcomes have been reported.
	**CONFLICT OF INTEREST**	Probably low	The authors declare they have no competing financial interests.

**Marilia Cristina Oliveira Souza (2020)**	**DETECTION BIAS [Key element]:** can we be confident in the exposure characterization?	Probably low	Concentrations of thirteen PFASs were determined in whole blood collected during the second trimester from 252 pregnant Brazilian women.
	**DETECTION BIAS [Key element]:** can we be confident in the outcome assessment?	Probably low	Birth outcome data were extracted from hospital maternity records by medical personnel.
	**CONFOUNDING BIAS [Key element]:** did the study design or analysis account for important confounding and modifying variables?	Definitely high	Adjusted GM.
	**SELECTION BIAS:** did selection of study participants result in appropriate comparison groups?	Probably high	This is a nested case-control study from a cohort of 1400 women with singleton pregnancies who were recruited, and interviewed between 22 and 26 weeks of gestational age in the city of Ribeirão Preto, Brazil during 2010-2011.
	**ATTRITION/EXCLUSION BIAS:** were outcome data	Probably low	There is no evidence of exclusion bias.

	incomplete due to attrition or exclusion from analysis?		
	**SELECTIVE REPORTING BIAS:** were all measured outcomes reported?	Probably low	The authors stated: "PFOS and PFOA concentrations in pregnant women were positively associated with IUGR ($p < 0.05$)".
	**CONFLICT OF INTEREST**	Probably low	The authors declare they have no actual or potential competing financial interests.
**Sverre Wikström (2020)**	**DETECTION BIAS [Key element]:** can we be confident in the exposure characterization?	Probably low	Eight PFASs were analyzed in maternal serum (median: 10 weeks of pregnancy).
	**DETECTION BIAS [Key element]:** can we be confident in the outcome assessment?	Definitely low	Data were retrieved from the Swedish Medical Birth Register, where Swedish births are reported by healthcare professionals after delivery regarding infant BW and gestational age (GA), as well as maternal weight, age, and parity.
	**CONFOUNDING BIAS [Key element]:** did the study design or analysis account for important confounding and modifying variables?	Probably low	All analyses were adjusted for maternal weight,and cotinine levels. Analyses including both boys and girls were in addition adjusted for se, parity, gestational age.
	**SELECTION BIAS:** did	Probably low	SELMA is a longitudinal pregnancy cohort study designed to investigate the impacts of early life

	selection of study participants result in appropriate comparison groups?		exposure to environmental factors on growth, development, and chronic diseases in children.
	**ATTRITION/EXCLUSION BIAS:** were outcome data incomplete due to attrition or exclusion from analysis?	Definitely low	Note that GA may constitute an intermediate along a pathway from PFAS exposure to lower BW. Therefore, all associations were evaluated here by adjustments for GA but also utilizing BW-SDS (i.e., BW for GA) and SGA birth as separate outcome measures and by a statistical mediator analysis18 on this association. Regression models of PFASs as a predictor of BW were also rerun after exclusion of preterm (<37 weeks) born infants (n = 56), as well as without adjustment for GA.
	**SELECTIVE REPORTING BIAS:** were all measured outcomes reported?	Probably low	The authors stated: "Our data suggested that prenatal exposures to PFOS, PFOA, PFNA, PFDA, and PFUnDA contribute to low BW as well as to being born SGA and that these associations were more pronounced and statistically significant only in girls".
	**CONFLICT OF INTEREST**	Probably low	The authors declare no competing interests
**Lin Chen (2021)**	**DETECTION BIAS [Key element]:** can we be confident in the exposure characterization?	Probably low	The author quantified 10 PFAS with high-performance liquid chromatography/tandem mass spectrometry (HPLCMS/MS) in maternal plasma at three trimesters and cord blood at delivery.
	**DETECTION BIAS [Key element]:** can we be confident in the outcome assessment?	probably low	All birth outcomes were measured immediately after birth by a midwife.

	**CONFOUNDING BIAS [Key element]:** did the study design or analysis account for important confounding and modifying variables?	probably low	Pre-pregnancy body mass index (BMI), educational level, occupation, family income, smoking and alcohol use, and fetal sex, maternal age parity, gestational age.
	**SELECTION BIAS:** did selection of study participants result in appropriate comparison groups?	probably low	The SBC is a multicenter prospective study that examines the influence of environmental exposure on maternal and child health.
	**ATTRITION/EXCLUSION BIAS:** were outcome data incomplete due to attrition or exclusion from analysis?	probably high	The authors stated: "During the analysis, we excluded some women with multiple gestations or pregnant complications. Such selection and exclusion might have resulted in some selection bias in our study".
	**SELECTIVE REPORTING BIAS:** were all measured outcomes reported?	Probably low	The concentrations of most PFAS we observed in the maternal circulation declined during pregnancy while PFHxS level remained unchanged and PFBS level increased. Several PFAS were negatively associated with birth length and the sensitive window of exposure appeared to be the first trimester. The associations were stronger for female fetuses.
	**CONFLICT OF INTEREST**	Probably low	The authors declare no conflicts of interest.

Table S5. Sensitivity analyses of the associations between PFAS with diverse indicators of birth outcome for per 1 ng/ml increment of exposure.

**PFAS and birth outcome indicators pairs**	**Study omitted**	**Effect estimates (95% CI)**	**Egger test**
**Birth weight (BW)**			
PFOS	Alkhalawi et al. 2016	-0.040 (-0.098,0.017)	0.225
	Bach et al. 2016	-0.034 (-0.075,0.007)	
	Bach et al. 2016	-0.035 (-0.079,0.009)	
	Valvi et al. 2017	-0.034 (-0.076,0.007)	
	Meng et al. 2018	-0.030 (-0.064,0.003)	
	Bjerregaard et al.2019	-0.035 (-0.081,0.010)	
	Marks et al. 2019	-0.034 (-0.076,0.008)	
	Shoaff et al. 2018	-0.034 (-0.078,0.008)	

	Marks et al. 2019	-0.035 (-0.080,0.009)	
	Bach et al. 2016	-0.035 (-0.080,0.009)	
	Bjerregaard et al. 2019	-0.030 (-0.064,0.003)	
	Bach et al. 2016	-0.0344 (-0.077,0.008)	
	Bjerregaard et al. 2019	-0.035 (-0.080,0.010)	
	Ashley et al. 2017 male	-0.035 (-0.081,0.010)	
	Ashley et al. 2017 female	-0.034 (-0.078,0.008)	
	Whitworth et al. 2012	-0.038 (-0.095,0.017)	
	Wang et al. 2016 male	-0.040 (-0.097,0.017)	
	Sagiv et al. 2018	-0.046 (-0.108,0.016)	
	Sagiv et al. 2018	-0.022 (-0.059,0.014)	
PFOA	Alkhalawi et al. 2016	-0.020 (-0.050,0.010)	0.466
	Bach et al. 2016	-0.020 (-0.048,0.006)	

	Shoaff et al. 2018	-0.020 (-0.048,0.006)	
	Marks et al. 2019	-0.020 (-0.048,0.006)	
	Ashley et al. 2017 male	-0.020 (-0.048,0.006)	
	Ashley et al. 2017 female	-0.020 (-0.048,0.006)	
	Whitworth et al. 2012	-0.019 (-0.049,0.010)	
	Wang et al. 2016 male	-0.019 (-0.040,0.010)	
	Sagiv et al. 2018	-0.027 (-0.065,0.011)	
PFHxS	Alkhalawi et al. 2016	0.000(-0.010,0.011)	0.411
	Bach et al. 2016	-0.000(-0.011,0.010)	
	Bjerregaard et al. 2019	-0.002(-0.030,0.025)	
	Marks et al. 2019	-0.001(-0.022,0.019)	
	Ashley et al. 2017 male	-0.001(-0.026,0.022)	
	Ashley et al. 2017 female	-0.003(-0.033,0.027)	

	Ashley et al. 2017	-0.021(-0.090,0.048)	
	Sagiv et al. 2018	-0.019(-0.103,0.064)	
PFNA	Bach et al. 2016	-20.130 (-52.323,12.062)	0.236
	Valvi et al. 2017	-6.373 (-30.397,17.651)	
	Meng et al. 2018	-0.149 (-.2749, -0.024)	
	Bjerregaard et al. 2019	-12.895 (-42.161,16.371)	
	Marks et al. 2019	-8.076 (-31.094,014.941)	
	Sagiv et al. 2018	-20.747 (-60.843,19.348)	
PFUnDA	Bach et al. 2016	-122.727 (-238.636, -6.818)	-
	Bjerregaard et al. 2019	-20.000(-90.000,50.000)	
PFDA	Bach et al. 2016	-31.250(-156.250,93.750)	-
	Bjerregaard et al. 2019	50.000(-35.000,135.000)	
PFHpS	insufficient	insufficient	-

Gestational age (GA)			
PFOS	Apelberg et al. 2007	-0.328(-0.666,0.009)	0.959
	Maisonet et al. 2012	-0.377(-0.897,0.141)	
	Meng et al. 2018	-0.242(-0.445, -0.039)	
	Sagiv et al. 2018	-0.299(-0.864,0.266)	
	Eick et al. 2020	-0.376(-0.824,0.071)	
PFOA	Apelberg et al. 2007	-0.304 (-0.706,0.097)	0.658
	Maisonet et al. 2012	-0.246 (-0.856,0.364)	
	Meng et al. 2018	-0.122 (-0.338,0.094)	
	Sagiv et al. 2018	-0.367 (-0.993,0.2581)	
	Eick et al. 2020	-0.318(-0.873,0.236)	
PFHxS	Maisonet et al. 2012	-0.088 (-0.328, 0.150)	0.516
	Meng et al. 2018	-0.115 (-0.320,0.089)	

	Sagiv et al. 2018	-0.172 (-0.479,0.134)	
	Eick et al. 2020	-0.165 (-0.382,0.052)	
PFHpS	Meng et al. 2018	-2.000 (-3.300, -0.700)	-
PFNA	insufficient	insufficient	-
PFDA	insufficient	insufficient	-
**Birth length（BL）**			
PFOS	Alkhalawi et al.2016	-0.034 (-0. 063, -0.006)	0.193
	Bjerregaard et al.2019	-0.038 (-0.073, -0.003)	
	Marks et al. 2019	-0.021 (-0.069,0.026)	
PFOA	Maisonet et al. 2012	0.009 (-0.078,0.097)	0.625
	Alkhalawi et al. 2016	-0.021 (-0.139,0.096)	
	Bjerregaard et al. 2019	-0.028(-0.267,0.209)	
	Marks et al. 2019	-0.024 (-0.244,0.195)	

PFHxS	Alkhalawi et al. 2016	0.161(-0.290,0.612)	0.446
	Bjerregaard et al. 2019	-0.019 (-0.059,0.020)	
	Marks et al. 2019	0.251 (-0.167,0.669)	
PFNA	Bjerregaard et al. 2019	-0.790 (-2.024,0.444)	-
	Marks et al. 2019	0.000(-0.441,0.441)	
PFUnDA	Bjerregaard et al. 2019	0.000(-0.909,0.454)	-
PFDA	Bjerregaard et al. 2019	0.000(-0.625,0.625)	-
PFHpS	Bjerregaard et al. 2019	-1.666(-3.333,0.833)	-
**Head circumference (HC)**			
PFOS	Bjerregaard et al. 2019	-0.02(-0.039, -0.001)	-
	Marks et al. 2019	-0.024 (-0.060,0.012)	
PFOA	Bjerregaard et al. 2019	0.030(-0.070,0.130)	-
	Marks et al. 2019	0.108 (2.513e-09,0.217)	

PFUnDA	Bjerregaard et al. 2019	0.000(-0.909,0.454)	-
PFHxS	Bjerregaard et al. 2019	-0.001(-0.02,0.024)	-
	Marks et al. 2019	0.000(-0.454,0.454)	
PFNA	Bjerregaard et al. 2019	0.000(-0.294,0.588)	-
	Marks et al. 2019	-0.510(-1.260,0.250)	
PFDA	Bjerregaard et al. 2019	0.000(-0.62,0.625)	-
PFHpS	Bjerregaard et al. 2019	0.000(-1.66,0.833)	-
**Preterm birth (PTB)**			
PFOA	Savitz et al. 2012	1.000(0.800,1.100)	-
	Sagiv et al. 2018	1.000(0.972,1.071)	
PFOS	Sagiv et al. 2018	1.006(1.000,1.016)	-
PFHxS	Sagiv et al. 2018	1.000(0.953,1.044)	-
PFNA	Sagiv et al. 2018	1.577(1.000,2.319)	-

**Low birth weight (LBW)**			
PFOA	Savitz et al. 2012	0.999(0.997,1.001)	-
**Ponderal index (PI)**			
PFOA	Alkhalawi et al. 2016	-0.412(-0.788, -0.037)	-
	Bach et al. 2016	0.000(0.000,0.000)	
PFOS	Alkhalawi et al. 2016	-0.355(-0.702, -0.008)	-
	Bach et al. 2016	0.000(0.000,0.000)	
PFHxS	Alkhalawi et al. 2016	-0.552(-1.110,0.006)	-
	Bach et al. 2016	-0.100(-0.300,0.000)	
PFNA	Bach et al. 2016	0.100(0.000,0.200)	-
PFUnDA	Bach et al. 2016	0.000(-0.200,0.100)	-
PFDA	Bach et al. 2016	0.100(-1.100,0.300)	-
PFHpS	Bach et al. 2016	-0.400(-0.900,0.200)	-

Table S6. Sensitivity analyses of the associations between PFAS with diverse indicators of birth outcome for per 1 ln(ng/ml) increment of exposure.

PFAS and birth outcome indicators pairs	Study omitted	Effect estimates (95% CI)	Egger test
**Birth weight (BW)**			
PFOS	Apelberg et al. 2007	-0.398 (-1.349, 0.552)	0.000
	Washino et al. 2009|	-0.381 (-1.324, 0.562)	
	Chen et al. 2012	-0.347 (-1.230, 0.536)	
	Darrow et al. 2013	-0.391 (-1.346, 0.564)	
	Callan et al. 2016	-0.417 (-1.385, 0.551)	
	Kwon et al. 2016	-0.380 (-1.318, 0.558)	
	Lee et al. 2016	-0.407 (-1.362, 0.548)	
	Chen et al. 2017	-0.390 (-1.325, 0.544)	
	Lauritzen et al. 2017 Noway	-0.419 (-1.378, 0.539)	
	Lauritzen et al. 2017 Sweden	-0.378 (-1.289, 0.533)	
	Li et al. 2017<br>Manzano et al. 2017	-0.349 (-1.240, 0.541)<br>-0.424 (-1.398, 0.550)	
	Minatoya et al. 2017	-0.422 (-1.395, 0.552)	
	Shi et al. 2017	-0.424 (-1.370, 0.523)	

	Starling et al. 2017	-0.413 (-1.383, 0.558)	
	Valvi et al. 2017	-0.401 (-1.351, 0.548)	
	Meng et al. 2018	-0.355 (-1.264, 0.554)	
	Wang et al. 2019	-0.411 (-1.375, 0.553)	
	Xu et al. 2019	-0.387 (-1.310, 0.535)	
	Hjermitslev et al. 2020	-0.323 (-1.288, 0.642)	
	Kashino et al. 2020	-0.398 (-1.366, 0.570)	
	Wikstrom et al. 2020	-0.378 (-1.315, 0.560)	
	Chen et al. 2021	-0.424 (-1.397, 0.550)	
	Shoaff et al. 2018	-25.201 (-37.477, -12.926)	
	Shoaff et al. 2018	-25.204 (-37.485, -12.924)	

PFOA	Apelberg et al. 2007	-0.122 (-0.536, 0.293)	0.000
	Washino et al. 2009	-0.122 (-0.543, 0.300)	
	Chen et al. 2012	-0.124 (-0.549, 0.300)	
	Wu et al. 2012	-0.122 (-0.536, 0.291)	
	Darrow et al. 2013	-0.123 (-0.548, 0.303)	
	Callan et al. 2016	-0.126 (-0.552, 0.300)	
	Kwon et al. 2016	-0.121 (-0.533, 0.292)	
	Lee et al. 2016	-0.126 (-0.553, 0.301)	
	Lenters et al. 2016	-0.119 (-0.530, 0.292)	
	Lauritzen et al. 2017 Noway	-0.127 (-0.553, 0.299)	

	Lauritzen et al. 2017 Sweden	-0.118 (-0.512, 0.277)	
	Li et al. 2017	-0.109 (-0.483, 0.264)	
	Manzano et al. 2017	-0.125 (-0.551, 0.301)	
	Minatoya et al. 2017	-0.120 (-0.532, 0.293)	
	Shi et al. 2017	-0.127 (-0.550, 0.296)	
	Starling et al. 2017	-0.118 (-0.527, 0.291)	
	Valvi et al. 2017	-0.126 (-0.553, 0.301)	
	Meng et al. 2018	-0.117 (-0.525, 0.291)	
	Wang et al. 2019	-0.126 (-0.552, 0.301)	
	Xu et al. 2019	-0.125 (-0.546, 0.296)	

	Hjermitslev et al. 2020	-0.117 (-0.514, 0.281)	
	Kashino et al. 2020	-0.122 (-0.547, 0.304)	
	Wikstrom et al. 2020	-0.113 (-0.506, 0.280)	
	Chen et al. 2021	-0.127 (-0.553, 0.299)	
	Wang et al. 2016 female	-0.123 (-0.540, 0.295)	
	Wang et al. 2016 male	-0.127 (-0.551, 0.297)	
	Shoaff et al. 2018	-0.198 (-0.819, 0.424)	
	Wang et al. 2016 female	-0.142 (-0.696, 0.412)	
	Wang et al. 2016 male	-0.234 (-0.890, 0.421)	
	Shoaff et al. 2018	-0.146 (-0.723, 0.431)	
PFHxS	Callan et al. 2016	-0.093 (-0.197, 0.012)	0.285

	Kwon et al. 2016	-0.093 (-0.197, 0.012)	
	Lee et al. 2016	-0.093 (-0.197, 0.011)	
	Li et al. 2017	-0.093 (-0.197, 0.012)	
	Manzano et al. 2017	-0.093 (-0.197, 0.012)	
	Shi et al. 2017	-0.093 (-0.198, 0.011)	
	Starling et al. 2017	-0.093 (-0.197, 0.012)	
	Valvi et al. 2017	-0.093 (-0.197, 0.011)	
	Meng et al. 2018	-0.093 (-0.197, 0.011)	
	Xu et al. 2019	-0.093 (-0.197, 0.011)	
	Hjermitslev et al. 2020	-0.093 (-0.197, 0.011)	
	Kashino et al. 2020	-0.093 (-0.197, 0.012)	

	Wikstrom et al. 2020	-0.093 (-0.197, 0.011)	
	Chen et al. 2021	-0.093 (-0.197, 0.011)	
	Shoaff et al. 2018	-0.109 (-0.272, 0.054)	
	Shoaff et al. 2018	-0.082 (-0.218, 0.054)	
PFNA	Chen et al. 2012	-0.076 (-0.380, 0.228)	0.001
	Callan et al. 2016	-0.075 (-0.380, 0.231)	
	Kwon et al. 2016	-0.068 (-0.331, 0.194)	
	Lee et al. 2016	-0.074 (-0.372, 0.224)	
	Li et al. 2017	-0.072 (-0.365, 0.221)	
	Manzano et al. 2017	-0.073 (-0.376, 0.229)	
	Shi et al. 2017	-0.075 (-0.380, 0.230)	

	Starling et al. 2017	-0.069 (-0.337, 0.200)	
	Xu et al. 2019	-0.074 (-0.370, 0.223)	
	Hjermitslev et al. 2020	-0.072 (-0.368, 0.223)	
	Kashino et al. 2020	-0.065 (-0.323, 0.193)	
	Wikstrom et al. 2020	-0.070 (-0.347, 0.208)	
	Chen et al. 2021	-0.074 (-0.375, 0.227)	
	Wang et al. 2016 female	-0.071 (-0.353, 0.211)	
	Wang et al. 2016 male	-0.075 (-0.379, 0.230)	
	Shoaff et al. 2018	-0.100 (-0.518, 0.318)	
	Wang et al. 2016 female	-0.041 (-0.449, 0.368)	
	Wang et al. 2016 male	-0.123 (-0.600, 0.353)	

	Shoaff et al. 2018	-0.099 (-0.488, 0.290)	
PFUnDA	Chen et al. 2012	-0.079 (-0.355, 0.198)	0.051
	Callan et al. 2016	-0.082 (-0.363, 0.200)	
	Kwon et al. 2016	-0.076 (-0.317, 0.165)	
	Lee et al. 2016	-0.084 (-0.384, 0.217)	
	Li et al. 2017	-0.081 (-0.372, 0.209)	
	Shi et al. 2017	-0.082 (-0.380, 0.216)	
	Xu et al. 2019	-0.083 (-0.379, 0.213)	
	Hjermitslev et al. 2020	-0.080 (-0.369, 0.209)	
	Kashino et al. 2020	-0.075 (-0.336, 0.186)	
	Wikstrom et al. 2020	-0.082 (-0.378, 0.214)	

	Chen et al. 2021	-0.083 (-0.384, 0.217)	
	Wang et al. 2016 female	-0.075 (-0.315, 0.165)	
	Wang et al. 2016 male	-0.084 (-0.384, 0.216)	
	Wang et al. 2016 female	-12.768 (-24.019, -1.517)	
	Wang et al. 2016 male	-12.757 (-23.942, -1.572)	
PFHpS	Meng et al. 2018	-269.000 (-607.800, 69.800)	-
	Hjermitslev et al. 2020	-52.871 (-98.742, -7.000)	
PFDA	Kwon et al. 2016	-21.531 (-34.833, -8.228)	0.376
	Lee et al. 2016	-24.664 (-40.112, -9.216)	
	Li et al. 2017	-23.812 (-39.770, -7.855)	
	Shi et al. 2017	-27.366 (-41.579, -13.152)	

	Valvi et al. 2017	-23.808 (-39.362, -8.255)	
	Meng et al. 2018	-27.150 (-44.374, -9.927)	
	Xu et al. 2019	-24.809 (-39.693, -9.925)	
	Hjermitslev et al. 2020	-25.659 (-42.640, -8.678)	
	Kashino et al. 2020	-29.781 (-51.139, -8.423)	
	Wikstrom et al. 2020	-20.285 (-34.021, -6.550)	
	Chen et al. 2021	-26.094 (-42.064 -10.123)	
PFDoDA	Kwon et al. 2016	-0.132 (-0.655, 0.390)	0.030
	Lee et al. 2016	-0.138 (-0.698, 0.422)	
	Li et al. 2017	-0.133 (-0.667, 0.401)	

	Xu et al. 2019	-0.137 (-0.685, 0.412)	
	Kashino et al. 2020	-0.120 (-0.627, 0.386)	
	Chen et al. 2021	-0.133 (-0.651, 0.386)	
	Wang et al. 2016 female	-0.123 (-0.562, 0.316)	
	Wang et al. 2016 male	-0.138 (-0.682, 0.405)	
	Wang et al. 2016 female	-17.439 (-39.494, 4.616)	
	Wang et al. 2016 male	-17.413 (-39.273, 4.447)	
PFTrDA	Kwon et al. 2016	-11.471 (-29.575, 6.633)	-
	Kashino et al. 2020	-48.620 (-119.080, 21.840)	
PFHpA	Li et al. 2017	14.716 (-115.963, 145.395)	0.678
	Xu et al. 2019	-8.541 (-102.664, 85.583)	

	Wikstrom et al. 2020	-41.443 (-243.281, 160.394)	
	Chen et al. 2021	-52.383 (-152.395, 47.629)	
PFBA	insufficient	insufficient	-
PFDeA	Starling et al. 2017	-0.104 (-0.637, 0.428)	0.586
	Wang et al. 2016 female	-0.085 (-0.430, 0.260)	
	Wang et al. 2016 male	-0.103 (-0.625, 0.418)	
	Wang et al. 2016 female	-4.110 (-41.393, 33.174)	
	Wang et al. 2016 male	-4.325 (-41.592, 32.942)	
PFBS	insufficient	insufficient	-
PFOSA	Robledo et al. 2015 girl	-1.159 (-2.159, -0.159)	0.443
	Robledo et al. 2015 boy	-0.104 (-1.104, 0.896)	

Gestational age (GA)			
PFOS	Apelberg et al. 2007	-0.071 (-0.163, 0.020)	0.270
	Chen et al. 2012	-0.028 (-0.105, 0.048)	
	Lauritzen et al. 2017 Norway	-0.063 (-0.157, 0.031)	
	Lauritzen et al. 2017 Sweden	-0.050 (-0.138, 0.037)	
	Li et al. 2017	-0.082 (-0.178, 0.014)	
	Manzano et al. 2017	-0.057 (-0.155, 0.042)	
	Meng et al. 2018	-0.025 (-0.106, 0.056)	
	Hjermitslev et al. 2020	-0.078 (-0.200, 0.045)	
	Huo et al. 2020	-0.078 (-0.190, 0.034)	
PFOA	Apelberg et al. 2007	-0.007 (-0.133, 0.120)	0.896
	Chen et al. 2012	-0.001 (-0.140, 0.138)	
	Wu et al. 2012	0.040 (-0.070, 0.149)	
	Lauritzen et al. 2017 Norway	0.022 (-0.106, 0.150)	
	Lauritzen et al. 2017 Sweden	0.020 (-0.107, 0.145)	

	Li et al. 2017	-0.015 (-0.148, 0.117)	
	Manzano et al. 2017	0.018 (-0.125, 0.161)	
	Meng et al. 2018	0.021 (-0.124, 0.165)	
	Hjermitslev et al. 2020	-0.028 (-0.132, 0.077)	
	Huo et al. 2020	0.006 (-0.143, 0.155)	
PFHxS	Li et al. 2017	-0.034 (-0.104, 0.036)	0.436
	Manzano et al. 2017	-0.008 (-0.114, 0.099)	
	Meng et al. 2018	0.002 (-0.103, 0.107)	
	Hjermitslev et al. 2020	0.002 (-0.063, 0.066)	
	Huo et al. 2020	-0.005 (-0.112, 0.101)	
PFHpS	Meng et al. 2018	-0.150 (-1.345, 1.045)	-
	Hjermitslev et al. 2020	-0.233 (-0.369, -0.097)	
PFNA	Chen et al. 2012	-0.038 (-0.126, 0.051)	0.540
	Li et al. 2017	-0.021 (-0.107, 0.066)	
	Manzano et al. 2017	-0.024 (-0.113, 0.064)	
	Meng et al. 2018	0.021 (-0.038, 0.079)	
	Hjermitslev et al. 2020	-0.023 (-0.107, 0.061)	
	Huo et al. 2020	-0.034 (-0.126, 0.057)	
PFDA	Li et al. 2017	-0.016 (-0.125, 0.094)	0.997

	Meng et al. 2018	0.049 (-0.028, 0.126)	
	Hjermitslev et al. 2020	0.002 (-0.115, 0.119)	
	Huo et al. 2020	-0.013 (-0.155, 0.129)	
PFUnDA	Chen et al. 2012	0.041 (-0.011, 0.093)	0.990
	Li et al. 2017	0.024 (-0.027, 0.076)	
	Hjermitslev et al. 2020	0.040 (-0.030, 0.111)	
	Huo et al. 2020	0.018 (-0.041, 0.077)	
PFHpA	Li et al. 2017	0.020 (-0.045, 0.085)	-
	Huo et al. 2020	0.140 (-0.170, 0.450)	
PFBA	insufficient	insufficient	-
PFBS	insufficient	insufficient	-
PFDoDA	Li et al. 2017	0.080 (-0.025, 0.185)	-
	Huo et al. 2020	0.070 (-0.150, 0.290)	
**Birth length（BL）**			
PFOS	Apelberg et al. 2007	-0.055 (-0.146, 0.035)	0.172
	Washino et al. 2009	-0.049 (-0.147, 0.049)	
	Chen et al. 2012	-0.037 (-0.128, 0.055)	
	Callan et al. 2016	-0.046 (-0.136, 0.044)	

	Chen et al. 2017	-0.026 (-0.115, 0.064)	
	Lauritzen et al. 2017 Noway	-0.050 (-0.143, 0.042)	
	Lauritzen et al. 2017 Sweden	-0.030 (-0.101, 0.040)	
	Manzano et al. 2017	-0.059 (-0.156, 0.037)	
	Shi et al. 2017	-0.071 (-0.162, 0.020)	
	Valvi et al. 2017	-0.051 (-0.142, 0.040)	
	Wang et al. 2019	-0.050 (-0.143, 0.042)	
	Kashino et al. 2020	-0.064 (-0.167, 0.039)	
	Chen et al. 2021	-0.024 (-0.108, 0.059)	
PFOA	Apelberg et al. 2007	-0.030 (-0.125, 0.065)	0.118
	Washino et al. 2009	-0.034 (-0.142, 0.074)	
	Chen et al. 2012	-0.037 (-0.140, 0.065)	
	Wu et al. 2012	-0.014 (-0.070, 0.041)	
	Callan et al. 2016	-0.033 (-0.127, 0.061)	

	Lauritzen et al. 2017 Noway	-0.030 (-0.125, 0.064)	
	Lauritzen et al. 2017 Sweden	-0.014 (-0.076, 0.047)	
	Manzano et al. 2017	-0.037 (-0.140, 0.067)	
	Shi et al. 2017	-0.048 (-0.145, 0.049)	
	Valvi et al. 2017	-0.035 (-0.131, 0.060)	
	Wang et al. 2019	-0.036 (-0.131, 0.059)	
	Kashino et al. 2020	-0.046 (-0.164, 0.073)	
	Chen et al. 2021	-0.015 (-0.104, 0.074)	
	Wang et al. 2016 female	-0.024 (-0.116, 0.067)	
	Wang et al. 2016 male	-0.039 (-0.128, 0.051)	
PFHxS	Manzano et al. 2017	-0.010 (-0.113, 0.093)	0.228
	Shi et al. 2017	-0.042 (-0.113, 0.029)	

	Valvi et al. 2017	0.002 (-0.087, 0.090)	
	Kashino et al. 2020	-0.045 (-0.177, 0.087)	
	Chen et al. 2021	-0.009 (-0.100, 0.083)	
PFNA	Chen et al. 2012	-0.089 (-0.176, -0.002)	0.509
	Callan et al. 2016	-0.051 (-0.182, 0.080)	
	Manzano et al. 2017	-0.059 (-0.210, 0.092)	
	Shi et al. 2017	-0.064 (-0.212, 0.085)	
	Valvi et al. 2017	-0.052 (-0.189, 0.085)	
	Kashino et al. 2020	-0.019 (-0.154, 0.116)	
	Chen et al. 2021	-0.011 (-0.135, 0.113)	
	Wang et al. 2016 female	-0.027 (-0.155, 0.101)	
	Wang et al. 2016 male	-0.039 (-0.173, 0.094)	

PFUnDA	Chen et al. 2012	-0.068 (-0.155, 0.020)	0.549
	Callan et al. 2016	-0.055 (-0.126, 0.017)	
	Shi et al. 2017	-0.065 (-0.178, 0.048)	
	Kashino et al. 2020	-0.078 (-0.174, 0.019)	
	Chen et al. 2021	-0.036 (-0.089, 0.018)	
	Wang et al. 2016 female	-0.037 (-0.090, 0.017)	
	Wang et al. 2016 male	-0.054 (-0.135, 0.027)	
PFDoDA	Kashino et al. 2020	-0.146 (-0.265, -0.027)	0.891
	Chen et al. 2021	-0.156 (-0.417, 0.105)	
	Wang et al. 2016 female	-0.139 (-0.254, -0.025)	
	Wang et al. 2016 male	-0.159 (-0.275, -0.043)	
PFTrDA	insufficient	insufficient	-

PFDA	Callan et al. 2016	-0.039 (-0.116, 0.039)	0.886
	Shi et al. 2017	-0.071 (-0.207, 0.065)	
	Valvi et al. 2017	-0.042 (-0.138, 0.054)	
	Kashino et al. 2020	-0.054 (-0.213, 0.104)	
	Chen et al. 2021	-0.015 (-0.081, 0.051)	
PFHpA	insufficient	insufficient	-
PFBS	insufficient	insufficient	-
PFDeA	Wang et al. 2016 female	-0.060 (-0.685, 0.565)	-
	Wang et al. 2016 male	-0.470 (-1.235, 0.295)	
**Head circumference (HC)**			
PFOS	Apelberg et al. 2007	-0.043 (-0.124, 0.038)	0.212
	Washino et al. 2009	-0.073 (-0.178, 0.032)	
	Chen et al. 2012	-0.038 (-0.120, 0.044)	

	Callan et al. 2016	-0.059 (-0.147, 0.029)	
	Lauritzen et al. 2017 Noway	-0.082 (-0.163, -0.002)	
	Lauritzen et al. 2017 Sweden	-0.054 (-0.140, 0.031)	
	Manzano et al. 2017	-0.084 (-0.190, 0.021)	
	Valvi et al. 2017	-0.072 (-0.166, 0.023)	
	Wang et al. 2019	-0.076 (-0.172, 0.021)	
	Kashino et al. 2020	-0.085 (-0.196, 0.027)	
	Chen et al. 2021	-0.068 (-0.159, 0.023)	
PFOA	Apelberg et al. 2007	-0.032 (-0.102, 0.038)	0.054
	Washino et al. 2009	-0.070 (-0.169, 0.029)	
	Chen et al. 2012	-0.060 (-0.151, 0.032)	
	Callan et al. 2016	-0.047 (-0.128, 0.033)	
	Lauritzen et al. 2017 Noway	-0.066 (-0.149, 0.016)	
	Lauritzen et al. 2017 Sweden	-0.047 (-0.127, 0.033)	
	Manzano et al. 2017	-0.052 (-0.145, 0.042)	
	Valvi et al. 2017	-0.061 (-0.149, 0.026)	
	Wang et al. 2019	-0.034 (-0.107, 0.039)	
	Kashino et al. 2020	-0.079 (-0.177, 0.020)	
	Chen et al. 2021	-0.054 (-0.133, 0.025)	

	Wang et al. 2016 female	-0.064 (-0.149, 0.021)	
	Wang et al. 2016 male	-0.065 (-0.151, 0.022)	
PFUnDA	Chen et al. 2012	-0.061 (-0.141, 0.019)	0.379
	Callan et al. 2016	-0.054 (-0.121, 0.013)	
	Kashino et al. 2020	-0.062 (-0.179, 0.055)	
	Chen et al. 2021	-0.058 (-0.124, 0.009)	
PFHxS	Callan et al. 2016	0.020 (-0.078, 0.117)	0.875
	Manzano et al. 2017	0.003 (-0.167, 0.172)	
	Valvi et al. 2017	-0.039 (-0.099, 0.022)	
	Kashino et al. 2020	0.023 (-0.135, 0.181)	
	Chen et al. 2021	0.004 (-0.105, 0.113)	
PFNA	Chen et al. 2012	-0.074 (-0.140, -0.008)	0.433
	Callan et al. 2016	-0.034 (-0.106, 0.037)	
	Manzano et al. 2017	-0.033 (-0.118, 0.053)	
	Valvi et al. 2017	-0.042 (-0.112, 0.028)	
	Kashino et al. 2020	-0.006 (-0.077, 0.065)	
	Chen et al. 2021	-0.036 (-0.107, 0.035)	
	Wang et al. 2016 female	-0.020 (-0.073, 0.033)	
	Wang et al. 2016 male	-0.032 (-0.105, 0.041)	

PFBS	insufficient	insufficient	-
PFDA	Callan et al. 2016	-0.009 (-0.155, 0.136)	0.612
	Valvi et al. 2017	-0.065 (-0.149, 0.019)	
	Kashino et al. 2020	0.118 (-0.108, 0.345)	
	Chen et al. 2021	-0.015 (-0.151, 0.121)	
PFDoDA	Kashino et al. 2020	-0.183 (-0.411, 0.046)	0.475
	Chen et al. 2021	-0.107 (-0.247, 0.032)	
	Wang et al. 2016 female	-0.070 (-0.151, 0.011)	
	Wang et al. 2016 male	-0.144 (-0.349, 0.060)	
PFTrDA	insufficient	insufficient	-
PFHpA	insufficient	insufficient	-
PFDeA	Wang et al. 2016 female	-0.120 (-0.480, 0.240)	-
	Wang et al. 2016 male	-0.370 (-0.845, 0.105)	
**Ponderal index (PI)**			
PFHxS	Callan et al. 2016	-0.004 (-0.007, -0.000)	0.913
	Shi et al. 2017	-0.009 (-0.037, 0.019)	
	Xu et al. 2019	-0.008 (-0.059, 0.043)	
PFOA	Apelberg et al. 2007	-0.006 (-0.026, 0.014)	0.080

	Chen et al. 2012	-0.017 (-0.048, 0.013)	
	Wu et al. 2012	-0.016 (-0.040, 0.008)	
	Callan et al. 2016	-0.011 (-0.033, 0.012)	
	Minatoya et al. 2017	-0.009 (-0.028, 0.010)	
	Shi et al. 2017	-0.020 (-0.046, 0.007)	
	Wang et al. 2019	-0.007 (-0.029, 0.015)	
	Xu et al. 2019	-0.022 (-0.055, 0.011)	
PFOS	Apelberg et al. 2007	-0.007 (-0.029, 0.015)	0.205
	Chen et al. 2012	-0.021 (-0.052, 0.009)	
	Callan et al. 2016	-0.017 (-0.043, 0.008)	
	Minatoya et al. 2017	-0.014 (-0.035, 0.007)	
	Shi et al. 2017	-0.030 (-0.062, 0.002)	
	Wang et al. 2019	-0.014 (-0.041, 0.013)	
	Xu et al. 2019	-0.028 (-0.070, 0.013)	

PFUnDA	Chen et al. 2012	0.001 (-0.008, 0.010)	0.977
	Callan et al. 2016	-0.002 (-0.010, 0.007)	
	Shi et al. 2017	-0.004 (-0.014, 0.006)	
	Xu et al. 2019	-0.002 (-0.013, 0.009)	
PFNA	Chen et al. 2012	-0.003 (-0.007, 0.001)	0.538
	Callan et al. 2016	-0.009 (-0.022, 0.005)	
	Shi et al. 2017	-0.010 (-0.025, 0.005)	
	Xu et al. 2019	-0.018 (-0.030, -0.006)	
PFDA	Callan et al. 2016	-0.000 (-0.005, 0.004)	0.255
	Shi et al. 2017	-0.001 (-0.019, 0.016)	
	Xu et al. 2019	-0.004 (-0.023, 0.015)	

PFHpA	insufficient	insufficient	-
PFDoDA	insufficient	insufficient	-
**Preterm birth (PTB)**			
PFOA	Chen et al. 2012	1.044 (0.762, 1.327)	0.288
	Darrow et al. 2013	1.038 (0.657, 1.418)	
	Manzano et al. 2017	1.006 (0.708, 1.305)	
	Meng et al. 2018	0.954 (0.650, 1.257)	
	Huo et al. 2020	1.012 (0.686, 1.337)	
	Liu et al. 2020	0.976 (0.708, 1.245)	
	Souza et al. 2020	0.924 (0.788, 1.059)	
PFOS	Chen et al. 2012	1.215 (0.924, 1.505)	0.079
	Darrow et al. 2013	1.601 (1.093, 2.109)	
	Manzano et al. 2017	1.507 (1.093, 1.920)	
	Meng et al. 2018	1.399 (1.000, 1.798)	
	Huo et al. 2020	1.594 (1.151, 2.036)	
	Liu et al. 2020	1.610 (1.064, 2.157)	

	Souza et al. 2020	1.330 (0.990, 1.670)	
PFDA	Meng et al. 2018	0.880 (0.594, 1.166)	-
	Huo et al. 2020	2.057 (1.558, 2.556)	
PFHxS	Manzano et al. 2017	1.150 (0.864, 1.437)	0.898
	Meng et al. 2018	0.984 (0.633, 1.335)	
	Huo et al. 2020	0.960 (0.631, 1.290)	
PFUnDA	Chen et al. 2012	0.820 (0.525, 1.115)	-
	Huo et al. 2020	0.870 (0.573, 1.167)	
PFNA	Chen et al. 2012	1.042 (0.628, 1.455)	0.370
	Manzano et al. 2017	1.018 (0.687, 1.348)	
	Meng et al. 2018	0.867 (0.695, 1.040)	
	Huo et al. 2020	1.033 (0.653, 1.413)	
PFHpS	insufficient	insufficient	-
PFHpA	insufficient	insufficient	-
PFBS	insufficient	insufficient	-
PFDoDA	insufficient	insufficient	-
**Low birth weight (LBW)**			
PFOA	Chen et al. 2012	0.923 (0.693, 1.154)	0.327

	Darrow et al. 2013	0.890 (0.553, 1.227)	
	Manzano et al. 2017	0.917 (0.663, 1.171)	
	Meng et al. 2018	0.885 (0.635, 1.134)	
PFOS	Chen et al. 2012	1.174 (0.923, 1.425)	0.068
	Darrow et al. 2013	1.508 (0.870, 2.146)	
	Manzano et al. 2017	1.564 (0.932, 2.195)	
	Meng et al. 2018	1.403 (0.786, 2.019)	
PFHxS	Manzano et al. 2017	1.281 (0.769, 1.793)	-
	Meng et al. 2018	0.919 (0.546, 1.293)	
PFNA	Chen et al. 2012	1.237 (0.338, 2.136)	0.238
	Manzano et al. 2017	1.219 (0.265, 2.173)	

	Meng et al. 2018	0.791 (0.474, 1.108)	
PFUnDA	insufficient	insufficient	-
PFHpS	insufficient	insufficient	-
PFDA	insufficient	insufficient	-
**Small for gestational (SGA)**			
PFOA	Chen et al. 2012	1.900 (0.830, 2.971)	0.271
	Wang et al. 2016 male	2.003 (1.038, 2.968)	
	Lauritzen et al. 2017 Sweden	1.105 (0.772, 1.438)	
	Manzano et al. 2017	2.017 (0.865, 3.170)	
	Wikstrom et al. 2020	1.888 (0.661, 3.114)	
PFOS	Chen et al. 2012	1.163 (0.698, 1.629)	0.259
	Wang et al. 2016 male	1.610 (0.963, 2.257)	
	Lauritzen et al. 2017 Sweden	1.236 (0.703, 1.769)	
	Manzano et al. 2017	1.593 (0.867, 2.320)	
	Wikstrom et al. 2020	1.528 (0.707, 2.349)	

PFHxS	Manzano et al. 2017	0.960 (0.676, 1.244)	-
	Wang et al. 2016 male	0.973 (0.703, 1.243)	
PFUnDA	Chen et al. 2012	1.252 (0.817, 1.688)	0.557
	Wang et al. 2016 male	1.152 (0.663, 1.641)	
	Wang et al. 2016 female	1.037 (0.803, 1.271)	
	Wang et al. 2016 male	1.266 (0.862, 1.670)	
PFNA	Chen et al. 2012	1.218 (0.771, 1.665)	0.430
	Wang et al. 2016 male	1.247 (0.880, 1.614)	
	Wikstrom et al. 2020	1.049 (0.704, 1.393)	
	Wang et al. 2016 female	1.037 (0.768, 1.307)	
	Wang et al. 2016 male	1.178 (0.809, 1.548)	
PFDA	insufficient	insufficient	-
PFHpA	insufficient	insufficient	-
PFDeA	Wang et al. 2016 female	0.710 (-0.054, 1.474)	-
	Wang et al. 2016 male	3.140 (2.065, 4.215)	
PFDoDA	Wang et al. 2016 female	0.780 (0.183, 1.377)	-
	Wang et al. 2016 male	1.980 (0.986, 2.974)	

Table S7. Sensitivity analyses of the associations between PFAS with diverse indicators of birth outcome for highest versus lowest of exposure.

PFAS and birth outcome indicators pairs	Study omitted	Effect estimates (95% CI)	Egger test
**Birth weight (BW)**			
PFOS	Apelberg et al. 2007	-0.149 (-0.390, 0.091)	0.000
	Fei et al. 2008	-0.151 (-0.4054, 0.103)	
	Maisonet et al. 2012	-0.147 (-0.361, 0.067)	
	Darrow et al. 2013	-0.150 (-0.397, 0.094)	
	Bach et al 2016	-0.148 (-0.383, 0.086)	
	Starling et al 2017	-0.1498 (-0.384, 0.087)	
	Cao et al 2018	-0.151 (-0.405, 0.1093)	
	Meng et al 2018	-0.150 (-0.3869, 0.089)	

	Marks et al. 2019	-0.150 (-0.395, 0.095)	
	Wikstrom et al. 2020	-0.148 (-0.372, 0.076)	
	Whitworth et al. 2012	-0.148(-0.472, 0.176)	
	Shoaff et al. 2018	-0.162 (-0.481, 0.156)	
	Eick et al. 2020	-0.196 (-0.502, 0.111)	
	Sagiv et al. 2018	-0.166 (-0.540, 0.208)	
	Rokoff et al. 2018	-0.151 (-0.400, 0.099)	
	Shoaff et al. 2018	-0.114 (-0.404, 0.1764)	
PFOA	Apelberg et al. 2007	-0.154 (-0.424, 0.115)	0.004
	Fei et al. 2008	-0.153 (-0.425, 0.118)	
	Maisonet et al. 2012	-0.153 (-0.412, 0.105)	
	Darrow et al. 2013	-0.157 (-0.439, 0.125)	
	Bach et al. 2016	-0.157 (-0.439, 0.124)	

	Starling et al. 2017	-0.153 (-0.413, 0.106)	
	Cao et al. 2018	-0.157 (-0.438, 0.124)	
	Meng et al. 2018	-0.146 (-0.358, 0.065)	
	Marks et al. 2019	-0.157 (-0.437, 0.123)	
	Wikstrom et al. 2020	-0.156 (-0.430, 0.119)	
	Whitworth et al. 2012	-0.148 (-0.492, 0.196)	
	Shoaff et al. 2018	-0.170 (-0.572, 0.233)	
	Eick et al. 2020	-0.225 (-0.551, 0.101)	
	Sagiv et al. 2018	-0.191 (-0.597, 0.214)	
	Shoaff et al. 2018	-0.092 (-0.400, 0.217)	
PFHxS	Maisonet et al. 2012	-0.019 (-0.119, 0.088)	0.031
	Bach et al. 2016	-0.018 (-0.134, 0.097)	
	Starling et al. 2017	-0.025 (-0.178, 0.129)	

	Cao et al. 2018	-0.026 (-0.188, 0.137)	
	Meng et al. 2018	-0.026 (-0.190, 0.138)	
	Marks et al. 2019	-0.024 (-0.177, 0.128)	
	Wikstrom et al. 2020	-0.026 (-0.190, 0.139)	
	Shoaff et al. 2018	-0.005 (-0.187, 0.197)	
	Eick et al. 2020	-0.068 (-0.233, 0.097)	
	Sagiv et al. 2018	-0.044 (-0.297, 0.209)	
	Shoaff et al. 2018	-0.002 (-0.176, 0.179)	
PFNA	Bach et al. 2016	-0.107 (-0.399, 0.185)	0.091
	Starling et al. 2017	-0.105 (-0.306, 0.097)	
	Cao et al. 2018	-0.107 (-0.386, 0.172)	
	Meng et al. 2018	-0.105 (-0.346, 0.136)	
	Marks et al. 2019	-0.106 (-0.365, 0.153)	

	Wikstrom et al. 2020	-0.106 (-0.390, 0.177)	
	Shoaff et al. 2018	-0.136 (-0.542, 0.270)	
	Eick et al. 2020	-0.130 (-0.514, 0.253)	
	Sagiv et al. 2018	-0.097 (-0.565, 0.371)	
	Shoaff et al. 2018	-0.084 (-0.453, 0.285)	
PFUnDA	Bach et al. 2016	23.806 (-130.675, 178.29)	0.295
	Cao et al. 2018	-28.150 (-71.175, 14.875)	
	Wikstrom et al. 2020	37.158 (-83.568, 157.883)	
PFHpS	Bach et al. 2016	-102.600 (-169.000, -36.200)	-
	Meng et al. 2018	-43.000 (-100,.000 14.000)	
PFDA	Bach et al. 2016	-17.299 (-86.827, 52.229)	0.408
	Cao et al. 2018	-26.146 (-67.917, 15.626)	
	Meng et al. 2018	-8.958 (-80.748, 62.831)	

	Wikstrom et al. 2020	-3.097 (-37.186, 43.380)	
PFDoDA	insufficient	insufficient	-
PFTrDA	insufficient	insufficient	-
PFHpA	insufficient	insufficient	-
PFDS	insufficient	insufficient	-
PFDeA	insufficient	insufficient	-
PFTeA	insufficient	insufficient	-
PFHxA	insufficient	insufficient	-
Me-PFOSA-AcOH	insufficient	insufficient	-
**Gestational age (GA)**			
PFOS	Apelberg et al. 2007	-0.328 (-0.666, 0.010)	0.959
	Maisonet et al. 2012	-0.378 (-0.897, 0.141)	
	Meng et al. 2018	-0.243 (-0.446, -0.040)	

	Sagiv et al. 2018	-0.299 (-0.8651, 0.267)	
	Eick et al. 2020	-0.376 (-0.824, 0.071)	
PFOA	Apelberg et al. 2007	-0.305 (-0.707, 0.098)	0.658
	Maisonet et al. 2012	-0.246 (-0.857, 0.365)	
	Meng et al. 2018	-0.122 (-0.339, 0.095)	
	Sagiv et al. 2018	-0.368 (-0.994, 0.258)	
	Eick et al. 2020	-0.318 (-0.873, 0.237)	
PFHxS	Maisonet et al. 2012	-0.089 (-0.328, 0.151)	0.516
	Meng et al. 2018	-0.115 (-0.320, 0.090)	
	Sagiv et al. 2018	-0.173 (-0.480, 0.135)	
	Eick et al. 2020	-0.165 (-0.383, 0.052)	
PFHpS	insufficient	insufficient	-
PFNA	Meng et al. 2018	-0.639 (-2.011, 0.733)	0.421

	Sagiv et al. 2018	-0.194 (-0.409, 0.021)	
	Eick et al. 2020	-0.721 (-1.854, 0.413)	
PFDA	insufficient	insufficient	-
Et-PFOSA-AcOH	insufficient	insufficient	-
**Birth length (BL)**			
PFOS	Apelberg et al. 2007	-0.210 (-0.555, 0.135)	0.021
	Fei et al. 2008	-0.200 (-0.566, 0.166)	
	Maisonet et al. 2012	-0.007 (-0.224, 0.210)	
	Cao et al. 2018	-0.199 (-0.553, 0.154)	
	Marks et al. 2019	-0.057 (-0.333, 0.220)	
PFOA	Apelberg et al. 2007	-0.425 (-0.643, -0.207)	0.973
	Fei et al. 2008	-0.224 (-0.485, 0.036)	
	Cao et al. 2018	-0.246 (-0.542, 0.050)	

	Marks et al. 2019	-0.325 (-0.604, -0.046)	
PFHxS	Maisonet et al. 2012	-0.163 (-0.453, 0.128)	0.485
	Cao et al. 2018	-0.625 (-1.045, -0.205)	
	Marks et al. 2019	-0.427 (-1.161, 0.308)	
PFNA	Cao et al. 2018	-0.300 (-0.900, 0.300)	-
	Marks et al. 2019	-0.330 (-0.017, 0.677)	
PFUnDA	insufficient	insufficient	-
PFDoDA	insufficient	insufficient	-
PFTrDA	insufficient	insufficient	-
PFDA	insufficient	insufficient	-
PFTeA	insufficient	insufficient	-
PFDS	insufficient	insufficient	-
**Head circumference (HC)**			

PFOS	Apelberg et al. 2007	-0.087 (-0.357, 0.184)	0.968
	Fei et al. 2008	-0.264 (-0.440, -0.088)	
	Marks et al. 2019	-0.125 (-0.419, 0.169)	
PFOA	Apelberg et al. 2007	-0.108 (-0.312, 0.096)	0.050
	Fei et al. 2008	-0.187 (-0.353, -0.021)	
	Marks et al. 2019	-0.198 (-0.350, -0.046)	
PFHxS	insufficient	insufficient	-
PFNA	insufficient	insufficient	-
**Preterm birth (PTB)**			
PFOA	Fei et al. 2007	1.245 (0.890, 1.600)	0.218
	Savitz et al. 2012	1.367 (0.943, 1.790)	
	Whitworth et al. 2012	1.324 (0.982, 1.667)	
	Darrow et al. 2013	1.331 (0.930, 1.732)	

	Meng et al. 2018	1.512 (0.847, 1.4556)	
	Sagiv et al. 2018	1.301 (0.896, 1.706)	
	Eick et al. 2020	1.162 (0.877, 1.447)	
	Liu et al. 2020	1.329 (0.929, 1.729)	
PFOS	Fei et al. 2007	1.256 (0.592, 1.920)	0.012
	Whitworth et al. 2012	1.416 (0.973, 1.859)	
	Darrow et al. 2013	1.370 (0.574, 2.167)	
	Meng et al. 2018	1.150 (0.513, 1.786)	
	Sagiv et al. 2018	1.098 (0.510, 1.686)	
	Eick et al. 2020	1.219 (0.575, 1.862)	
	Liu et al. 2020	1.269 (0.545, 1.993)	
PFDA	insufficient	insufficient	-
PFHxS	Meng et al. 2018	1.270 (0.598, 1.943)	0.776

	Sagiv et al. 2018	1.039 (0.265, 1.813)	
	Eick et al. 2020	1.177 (0.601, 1.753)	
PFNA	Meng et al. 2018	1.750 (0.778, 2.724)	0.143
	Sagiv et al. 2018	1.768 (0.643, 2.894)	
	Eick et al. 2020	1.700 (0.896, 2.504)	
Me-PFOSA-AcOH	insufficient	insufficient	-
PFHpS	insufficient	insufficient	-
**Low birth weight (LBW)**			
PFOA	Fei et al. 2007	1.030 (0.621, 1.139)	0.222
	Savitz et al. 2012	1.283 (0.707, 1.858)	
	Darrow et al. 2013	1.232 (0.541, 1.923)	
	Meng et al. 2018	0.921 (0.453, 1.390)	
PFOS	Fei et al. 2007	1.248 (0.532, 1.963)	0.169

	Darrow et al. 2013	1.207 (0.308, 2.106)	
	Meng et al. 2018	1.342 (0.164, 2.520)	
PFHxS	insufficient	insufficient	-
PFNA	insufficient	insufficient	-
PFHpS	insufficient	insufficient	-
PFDA	insufficient	insufficient	-
**Small for gestational (SGA)**			
PFHxS	insufficient	insufficient	-
PFOA	Fei et al. 2007	1.282 (0.664, 1.899)	0.973
	Wang et al. 2016 male	1.122 (0.689, 1.554)	
	Whitworth et al. 2012	1.129 (0.683, 1.576)	
	Wikstrom et al. 2020	0.976 (0.474, 1.479)	
PFOS	Fei et al. 2007	0.980 (-0.064, 2.024)	0.288

	Wang et al. 2016 male	1.259 (0.864, 1.654)	
	Whitworth et al. 2012	0.906 (0.111, 1.700)	
	Wikstrom et al. 2020	0.684 (0.485, 1.319)	
PFUnDA	insufficient	insufficient	-
PFNA	insufficient	insufficient	-
PFDA	insufficient	insufficient	-
PFHpA	insufficient	insufficient	-
**Ponderal index (PI)**			
PFOA	Apelberg et al. 2007	0.034 (-0.017, 0.085)	0.115
	Maisonet et al. 2012	0.004 (-0.066, 0.075)	
	Bach et al. 2016	0.003 (-0.056, 0.063)	
	Cao et al. 2018	-0.014 (-0.059, 0.030)	
PFOS	Apelberg et al. 2007	-0.019 (-0.110, 0.071)	0.951

	Maisonet et al. 2012	-0.062 (-0.099, -0.026)	
	Bach et al. 2016	-0.018 (-0.094, 0.057)	
	Cao et al. 2018	-0.032 (-0.121, 0.058)	
PFHxS	Maisonet et al. 2012	-0.045 (-0.139, 0.048)	-
	Bach et al. 2016	-0.010 (-0.063, 0.043)	
	Cao et al. 2018	-0.040 (-0.130, 0.050)	
PFNA	Bach et al. 2016	0.100 (-0.075, 0.095)	-
	Cao et al. 2018	0.020 (-0.115, 0.155)	
PFUnDA	Bach et al. 2016	0.030 (-0.065, 0.125)	-
	Cao et al. 2018	0.003 (-0.143, 0.137)	
PFDA	Bach et al. 2016	0.300 (-0.060, 0.120)	-
	Cao et al. 2018	0.020 (-0.115, 0.155)	
PFDS	insufficient	insufficient	-

PFHxA	insufficient	insufficient	-
PFHpS	insufficient	insufficient	-
PFDoDA	insufficient	insufficient	-
PFTrDA	insufficient	insufficient	-
PFTeA	insufficient	insufficient	-

Table S8. Confidence in the body of evidence and level of evidence for each exposure-outcome combination.

**Exposure – Outcome Combination**	**Factors Decreasing Confidence "---" If No Concern; "↓" If Serious Concern to Downgrade Confidence**					**Factors Increasing Confidence "---" If Not Present; "↑" If Sufficient to Upgrade Confidence**					
Initial confidence for each body of evidence	Risk of Bias	Unexplained Inconsistency	Indirectness	Imprecision	Publication bias	Large magnitude	Dose Response	Environmental Confounding	Consistency	Final Confidence Rating	Level of evidence
**(A) continuous exposure ((a) log-transformed (per 1 ln (ng/ml) increment))**											
**Birth weight (BW)**											
PFOS<br>Moderate (23 studies)	↓	---	---	---	---	↑	---	---	---	Moderate	Moderate
PFOA<br>Moderate (30 studies)	---	---	---	---	---	---	---	---	---	Moderate	Moderate
PFHxS<br>Moderate (3 studies)	↓	---	---	---	↑	---	---	---	---	Moderate	Moderate

PFNA<br>Moderate (3 studies)	---	---	---	↓	---	↑	---	---	---	Moderate	Moderate
PFUnDA<br>Moderate (15 studies)	↓	---	---	---	---	---	---	---	---	Low	Inadequate
PFHpS<br>Moderate (2 studies)	↓	---	---	---	---	---	↑	---	---	Moderate	Inadequate
PFDA<br>Moderate (11 studies)	---	---	---	↓	---	---	---	---	---	Low	Inadequate
PFDoDA<br>Moderate (11 studies)	---	↓	---	---	---	↑	---	---	---	Moderate	Moderate
PFHpA<br>Moderate (4 studies)	---	---	---	---	---	↓	---	---	---	Low	Inadequate
PFTrDA<br>Moderate (2 studies)	---	---	---	---	---	---	---	---	---	Moderate	Moderate
PFDeA<br>Moderate (5 studies)	↑	---	---	---	---	---	↓	---	---	Moderate	Moderate

PFOSA<br>Moderate (2 studies)	---	---	---	---	---	---	---	---	---	Moderate	Moderate
**Gestational age (GA)**											
PFOS<br>Moderate (9 studies)	↓	---	---	---	---	↑	---	---	---	Moderate	Moderate
PFOA<br>Moderate (8 studies)	---	---	---	↓	---	---	---	---	---	Low	Inadequate
PFHxS<br>Moderate (1 study)	---	---	---	---	↓	---	---	---	---	Low	Inadequate
PFNA<br>Moderate (6 studies)	---	---	↓	---	---	↑	---	---	---	Moderate	Moderate
PFUnDA<br>Moderate (4 studies)	---	---	---	---	---	---	---	---	---	Moderate	Moderate
PFHpS<br>Moderate (2 studies)	↓	---	---	---	---	---	---	---	---	Low	Inadequate
PFDA	---	---	---	---	---	---	---	---	---	Moderate	Moderate

Moderate (4 studies)											
PFDoDA<br>Moderate (2 studies)	---	---	---	↓	---	---	---	---	---	Low	Inadequate
PFHpA<br>Moderate (2 studies)	---	---	---	---	---	---	---	---	---	Moderate	Moderate
**Birth length (BL)**											
PFOS<br>Moderate (13 studies)	---	---	---	---	---	---	---	---	---	Moderate	Moderate
PFOA<br>Moderate (15 studies)	↑	---	---	---	---	---	---	↓	---	Moderate	Moderate
PFHxS<br>Moderate (5 study)	---	---	---	---	↓	---	---	---	---	Low	Inadequate
PFNA<br>Moderate (9 studies)	---	---	↑	---	↓	---	---	---	---	Moderate	Moderate
PFUnDA<br>Moderate (7 studies)	---	---	---	---	↓	---	---	---	---	Low	Inadequate

PFHpS<br>Moderate (1 studies)	---	---	↓	---	↑	---	---	---	---	Moderate	Moderate
PFDA<br>Moderate (5 studies)	---	---	---	---	---	---	---	---	---	Moderate	Moderate
PFDoDA<br>Moderate (4 studies)	---	---	---	---	---	---	---	---	---	Moderate	Moderate
PFHpA<br>Moderate (1 studies)	---	---	---	---	---	---	-↓	---	---	Low	Inadequate
PFTrDA<br>Moderate (1 studies)	↓	---	---	---	---	---	---	---	---	Low	Inadequate
PFDeA<br>Moderate (2 studies)	---	---	---	---	---	---	---	---	---	Moderate	Moderate
**Head circumference (HC)**											
PFOS<br>Moderate (11 studies)	↓	---	---	---	---	---	---	---	---	Low	Inadequate
PFOA	---	---	---	---	---	---	---	---	---	Moderate	Moderate

Moderate (13 studies)											
PFHxS<br>Moderate (5 study)	---	---	---	---	↓	---	---	---	---	Low	Inadequate
PFNA<br>Moderate (9 study)	---	↓	---	---	---	---	↑	---	---	Moderate	Moderate
PFUnDA<br>Moderate (4 study)	---	---	---	---	↓	---	---	---	---	Low	Inadequate
PFHpS<br>Moderate (1 study)	---	---	---	---	---	---	---	↓	---	Low	Inadequate
PFDA<br>Moderate (4 studies)	---	---	---	---	---	---	---	---	---	Moderate	Moderate
PFDoDA<br>Moderate (4 studies)	---	---	---	---	---	---	---	---	---	Moderate	Moderate
PFHpA<br>Moderate (1 studies)	---	---	↓	---	---	---	---	---	---	Low	Inadequate
PFTrDA	↓	---	---	---	---	↑	---	---	---	Moderate	Moderate

Moderate (1 study)											
PFDeA<br>Moderate (2 studies)	---	---	---	---	---	---	---	---	---	Moderate	Moderate
**Ponderal index (PI)**											
PFOS<br>Moderate (7 studies)	↓	---	---	---	---	---	---	---	---	Low	Inadequate
PFOA<br>Moderate (8 studies)	---	---	---	---	---	---	---	---	---	Moderate	Moderate
PFHxS<br>Moderate (3 study)	---	---	---	---	↓	---	---	↑	---	Moderate	Moderate
PFNA<br>Moderate (4 study)	---	---	---	---	---	---	---	---	---	Moderate	Moderate
PFUnDA<br>Moderate (4 study)	---	---	---	---	↓	---	---	---	---	Low	Inadequate
PFHpS<br>Moderate (1 study)	---	---	---	---	↓	---	---	---	---	Low	Inadequate

PFDA<br>Moderate (3 studies)	---	---	---	---	---	---	---	---	---	Moderate	Moderate
PFDoDA<br>Moderate (1 studies)	---	---	↓	---	---	---	---	---	---	Low	Inadequate
PFHpA<br>Moderate (1 studies)	---	---	---	---	---	---	---	---	---	Moderate	Moderate
PFOSA<br>Moderate (2 study)	---	---	---	---	---	---	---	---	---	Moderate	Moderate
**Preterm birth (PTB)**											
PFOS<br>Moderate (7 studies)	---	---	---	---	---	---	---	---	---	Moderate	Moderate
PFOA<br>Moderate (7 studies)	---	---	---	---	---	---	---	---	---	Moderate	Moderate
PFHxS<br>Moderate (3 study)	↓	---	---	---	↓	---	---	↑	---	Low	Inadequate
PFNA	---	---	---	---	---	---	---	---	---	Moderate	Moderate

Moderate (4 study)											
PFUnDA<br>Moderate (2 study)	---	---	---	---	↓	---	---	---	---	Low	Inadequate
PFHpS<br>Moderate (1 study)	↓	---	---	---	---	---	---	---	---	Low	Inadequate
PFDA<br>Moderate (3 studies)	---	---	---	---	---	---	---	---	---	Moderate	Moderate
PFDoDA<br>Moderate (1 studies)	---	---	↓	---	---	---	---	---	---	Low	Inadequate
PFHpA<br>Moderate (1 studies)	---	---	---	---	---	---	---	---	---	Moderate	Moderate
PFOSA<br>Moderate (1 study)	---	---	↓	---	---	---	---	---	---	Low	Inadequate
**Low birth weight (LBW)**											
PFOS<br>Moderate (4 studies)	---	↓	---	---	---	---	---	---	---	Low	Inadequate

PFOA<br>Moderate (4 studies)	---	---	---	---	---	---	---	---	---	Moderate	Moderate
PFHxS<br>Moderate (2 study)	↓	---	---	---	↑	---	---	---	---	Moderate	Moderate
PFNA<br>Moderate (3 study)	---	---	---	---	---	---	---	---	---	Moderate	Moderate
PFUnDA<br>Moderate (1 study)	---	---	---	---	---	---	---	---	---	Moderate	Moderate
PFHpS<br>Moderate (1 study)	↓	---	---	---	---	---	---	---	---	Low	Inadequate
PFDA<br>Moderate (1 studies)	---	---	---	---	---	---	↓	---	---	Low	Inadequate
**Small for gestational (SGA)**											
PFOS<br>Moderate (5 studies)	---	---	---	---	---	---	---	---	---	Moderate	Moderate
PFOA	---	---	---	---	---	---	---	---	---	Moderate	Moderate

Moderate (4 studies)											
PFHxS<br>Moderate (2 study)	---	---	---	---	↓	---	---	---	---	Low	Inadequate
PFNA<br>Moderate (5 study)	---	---	---	---	---	---	---	---	---	Moderate	Moderate
PFUnDA<br>Moderate (4 study)	↓	---	---	---	---	↑	---	---	---	Moderate	Moderate
PFDA<br>Moderate (1 studies)	---	---	---	---	---	---	↓	---	---	Low	Inadequate
PFDoDA<br>Moderate (2 studies)	---	---	---	---	---	---	---	---	---	Moderate	Moderate
PFHpA<br>Moderate (1 studies)	---	---	---	---	↓	---	---	---	---	Low	Inadequate
PFDeA<br>Moderate (2 studies)	---	---	---	---	↓	---	---	---	---	Low	Inadequate
**(A) continuous exposure ((b) untransformed (per 1 ng/ml increment))**											

**Birth weight (BW)**											
PFOS<br>Moderate (12 studies)	↓	---	---	---	---	---	---	---	---	Low	Inadequate
PFOA<br>Moderate (9 studies)	---	---	---	---	---	---	---	---	---	Moderate	Moderate
PFHxS<br>Moderate (6 studies)	---	---	↓	---	↑	---	---	---	---	Moderate	Moderate
PFNA<br>Moderate (6 studies)	↓	---	---	↓	---	↑	---	---	---	Low	Inadequate
PFUnDA<br>Moderate (2 studies)	---	---	---	---	---	---	---	---	---	Moderate	Moderate
PFHpS<br>Moderate (2 studies)	↓	---	---	---	---	---	↑	---	---	Moderate	Inadequate
PFDA<br>Moderate (2 studies)	---	---	---	---	---	---	---	---	---	Moderate	Inadequate

**Gestational age (GA)**											
PFOS<br>Moderate (1 studies)	↓	---	---	---	---	---	---	---	---	Low	Inadequate
PFOA<br>Moderate (1 studies)	---	---	---	---	---	---	---	---	---	Moderate	Moderate
PFHxS<br>Moderate (1 study)	---	---	---	---	↓	---	---	---	---	Low	Inadequate
PFNA<br>Moderate (1 studies)	---	---	↓	---	---	↑	---	---	---	Moderate	Moderate
**Birth length (BL)**											
PFOS<br>Moderate (3 studies)	---	---	---	---	↓	---	---	---	---	Low	Inadequate
PFOA<br>Moderate (4 studies)	↑	---	---	---	---	---	---	↓	---	Moderate	Moderate
PFHxS<br>Moderate (3 study)	---	---	---	---	↓	---	---	---	---	Low	Inadequate

PFNA<br>Moderate (2 studies)	---	---	↑	---	↓	---	---	---	---	Moderate	Moderate
PFUnDA<br>Moderate (1 studies)	---	---	---	---	---	---	---	---	---	Moderate	Moderate
PFDA<br>Moderate (1 studies)	---	---	---	---	---	---	---	---	---	Moderate	Moderate
**Head circumference (HC)**											
PFOS<br>Moderate (2 studies)	↓	---	---	---	↑	---	---	---	---	Moderate	Moderate
PFOA<br>Moderate (2 studies)	---	---	---	---	---	---	---	---	---	Moderate	Moderate
PFHxS<br>Moderate (2 study)	---	---	---	---	↓	---	---	---	---	Low	Inadequate
PFNA<br>Moderate (2 study)	---	↓	---	---	---	---	↑	---	---	Moderate	Moderate
PFUnDA	---	---	---	---	↓	---	---	---	---	Low	Inadequate

Moderate (1 study)											
PFDA<br>Moderate (1 studies)	---	---	---	---	---	---	---	---	---	Moderate	Moderate
**Ponderal index (PI)**											
PFOS<br>Moderate (2 studies)	↓	---	---	---	---	↑	---	---	---	Moderate	Moderate
PFOA<br>Moderate (2 studies)	---	---	---	---	---	---	---	---	---	Moderate	Moderate
PFHxS<br>Moderate (2 study)	---	---	---	---	↓	---	---	↑	---	Moderate	Moderate
PFNA<br>Moderate (1 study)	---	---	---	---	---	---	---	---	---	Moderate	Moderate
PFUnDA<br>Moderate (1 study)	---	---	---	---	↓	---	---	---	---	Low	Inadequate
PFDA<br>Moderate (1 studies)	---	↓	---	---	---	---	---	---	---	Low	Inadequate

**Preterm birth (PTB)**											
PFOS<br>Moderate (1 studies)	---	---	---	---	↓	---	---	---	---	Low	Inadequate
PFOA<br>Moderate (2 studies)	---	---	---	---	---	---	---	---	---	Moderate	Moderate
PFHxS<br>Moderate (1 study)	↓	---	---	---	↓	---	---	↑	---	Low	Inadequate
PFNA<br>Moderate (1 study)	---	↓	---	---	---	---	↑	---	---	Moderate	Moderate
PFDA<br>Moderate (3 studies)	---	---	---	---	---	---	---	---	---	Moderate	Moderate
**Low birth weight (LBW)**											
PFOA<br>Moderate (1 studies)	---	---	---	---	---	---	---	---	---	Moderate	Moderate
**(B) Categorical exposure (high versus low)**											
**Birth weight (BW)**											

PFOS<br>Moderate (16 studies)	↓	---	↓	---	---	↑	---	---	---	Low	Inadequate
PFOA<br>Moderate (15 studies)	---	---	---	---	---	---	---	---	---	Moderate	Moderate
PFHxS<br>Moderate (11 studies)	---	---	---	---	---	---	---	---	---	Moderate	Moderate
PFNA<br>Moderate (10 studies)	↓	---	---	---	---	↑	---	---	---	Moderate	Moderate
PFUnDA<br>Moderate (3 studies)	↓	---	---	---	---	---	---	---	---	Low	Inadequate
PFHpS<br>Moderate (2 studies)	---	---	---	---	---	---	↓	---	---	Low	Inadequate
PFDA<br>Moderate (4 studies)	---	---	---	↓	---	---	---	---	---	Low	Inadequate
PFDoDA<br>Moderate (1 studies)	---	↓	---	---	---	↑	---	---	---	Moderate	Moderate

PFHpA<br>Moderate (1 studies)	---	---	---	---	---	↓	---	---	---	Low	Inadequate
PFTrDA<br>Moderate (1 studies)	---	---	---	---	---	---	---	---	---	Moderate	Moderate
PFDeA<br>Moderate (1 studies)	↑	---	---	---	---	---	↓	---	---	Moderate	Moderate
PFOSA<br>Moderate (1 studies)	---	---	---	---	---	---	---	---	---	Moderate	Moderate
**Gestational age (GA)**											
PFOS<br>Moderate (5 studies)	↓	---	---	---	---	↑	---	---	---	Moderate	Moderate
PFOA<br>Moderate (5 studies)	---	---	---	↓	---	---	---	---	---	Low	Inadequate
PFHxS<br>Moderate (4 study)	---	---	---	---	↓	---	---	---	---	Low	Inadequate
PFNA	---	---	↓	---	---	↑	---	---	---	Moderate	Moderate

Moderate (3 studies)											
PFHpS<br>Moderate (1 studies)	↓	---	---	---	---	---	---	---	---	Low	Inadequate
PFDA<br>Moderate (1 studies)	---	---	---	---	---	---	---	---	---	Moderate	Moderate
PFOSA<br>Moderate (1 studies)	↓	---	---	---	---	---	---	↑	---	Moderate	Moderate
**Birth length (BL)**											
PFOS<br>Moderate (5 studies)	---	---	---	---	---	---	---	---	---	Moderate	Moderate
PFOA<br>Moderate (4 studies)	↑	---	---	---	---	---	---	↓	---	Moderate	Moderate
PFHxS<br>Moderate (3 study)	---	---	---	---	↓	---	---	---	---	Low	Inadequate
PFNA<br>Moderate (29 studies)	---	---	↑	---	↓	---	---	---	---	Moderate	Moderate

PFUnDA<br>Moderate (1 studies)	---	---	---	---	---	---	---	---	---	Moderate	Moderate
PFDA<br>Moderate (1 studies)	---	---	↓	---	---	---	---	---	---	Low	Inadequate
PFDoDA<br>Moderate (1 studies)	---	---	---	---	---	---	---	---	---	Moderate	Moderate
PFTrDA<br>Moderate (1 studies)	↓	---	---	---	---	---	---	---	---	Low	Inadequate
**Head circumference (HC)**											
PFOS<br>Moderate (3 studies)	---	---	---	---	---	---	---	---	---	Moderate	Moderate
PFOA<br>Moderate (3 studies)	---	↓	---	---	↑	---	---	---	---	Moderate	Moderate
PFHxS<br>Moderate (1 study)	---	---	---	---	↓	---	---	---	---	Low	Inadequate
PFNA	---	↓	---	---	---	---	↑	---	---	Moderate	Moderate

Moderate (1 study)											
PFUnDA<br>Moderate (1 study)	---	---	---	---	↓	---	---	---	---	Low	Inadequate
**Ponderal index (PI)**											
PFOS<br>Moderate (4 studies)	↓	---	---	---	---	---	---	↑	---	Moderate	Moderate
PFOA<br>Moderate (4 studies)	---	---	---	---	---	---	---	---	---	Moderate	Moderate
PFHxS<br>Moderate (3 study)	---	---	---	---	↓	---	---	↑	---	Moderate	Moderate
PFNA<br>Moderate (2 study)	---	---	---	---	---	---	---	---	---	Moderate	Moderate
PFUnDA<br>Moderate (1 study)	---	↓	---	---	---	---	---	---	---	Low	Inadequate
PFHpS<br>Moderate (1 study)	---	---	---	---	↓	---	---	---	---	Low	Inadequate

PFDA<br>Moderate (2 studies)	---	---	---	---	---	---	---	---	---	Moderate	Moderate
PFDoDA<br>Moderate (1 studies)	---	---	↓	---	---	---	↑	---	---	Moderate	Moderate
PFTrDA<br>Moderate (1 study)	---	---	---	---	---	---	---	---	---	Moderate	Moderate
**Preterm birth (PTB)**											
PFOS<br>Moderate (7 studies)	---	↓	---	---	---	---	---	---	---	Low	Inadequate
PFOA<br>Moderate (8 studies)	---	---	---	---	---	---	---	---	---	Moderate	Moderate
PFHxS<br>Moderate (3 study)	---	---	---	---	↓	---	---	↑	---	Moderate	Moderate
PFNA<br>Moderate (3 study)	---	---	---	---	---	---	---	---	---	Moderate	Moderate
PFHpS	↓	---	---	---	---	---	---	---	---	Low	Inadequate

Moderate (1 study)											
PFDA<br>Moderate (1 studies)	---	---	↓	---	---	---	---	---	---	Low	Inadequate
PFOSA<br>Moderate (1 study)	---	---	---	---	---	---	---	---	---	Moderate	Moderate
**Low birth weight (LBW)**											
PFOS<br>Moderate (3 studies)	---	↓	---	---	---	---	↑	---	---	Moderate	Moderate
PFOA<br>Moderate (4 studies)	---	---	---	---	---	---	---	---	---	Moderate	Moderate
PFHxS<br>Moderate (1 study)	↓	---	---	---	↑	---	---	---	---	Moderate	Moderate
PFNA<br>Moderate (1 study)	---	---	---	---	---	---	---	---	---	Moderate	Moderate
PFHpS<br>Moderate (1 study)	↓	---	---	---	---	---	---	---	---	Low	Inadequate

PFDA<br>Moderate (1 studies)	---	---	---	---	---	---	↓	---	---	Low	Inadequate
**Small for gestational (SGA)**											
PFOS<br>Moderate (4 studies)	---	---	---	---	---	---	---	---	---	Moderate	Moderate
PFOA<br>Moderate (4 studies)	---	---	---	---	---	---	---	---	---	Moderate	Moderate
PFHxS<br>Moderate (1 study)	---	---	---	---	↓	---	---	---	---	Low	Inadequate
PFNA<br>Moderate (1 study)	---	---	---	---	---	---	---	---	---	Moderate	Moderate
PFUnDA<br>Moderate (1 study)	↓	---	---	---	---	↑	---	---	---	Moderate	Moderate
PFDA<br>Moderate (1 studies)	---	---	---	---	---	---	↓	---	---	Low	Inadequate
PFHpA	---	---	---	---	↓	---	---	---	---	Low	Inadequate

Moderate (1 studies)											

Table S9. Summary regression coefficients (β) or odds ratio (OR) and 95% CI by random effects meta-analysis stratified by different study characteristics (PFOA and PFOS were shown in table 4).

Birth Outcome indicators	Study characteristics		N	Summary β or OR (95% CI)	P value	I-squared	Tau-squared	N	Summary β or OR (95% CI)	P value	I-squared	Tau-squared
			PFUnDA					PFDoDA				
**BW/g**	**Gender** Male Female	A(a)	8	-0.032 (-0.082, 0.017)	0.185	30.5%	0.0012	8	-0.123 (-0.296, 0.050)	0.000	78.1%	0.0292
			4	0.010 (-0.033, 0.053)	0.881	0.0%	0.0000	4	0.046 (-0.023, 0.115)	0.971	0.0%	0.0000
			4	**-0.073 (-0.125, -0.022)**	0.383	1.9%	0.0001	4	-0.430 (-0.863, 0.004)	0.000	83.4%	0.1175
		A(b)	2	-13.663 (-118.169, 90.844)	0.785	0.0%	0.0000	0	-	-	-	-
			1	*-16.000 (-32.500, 0.500)*	-	0.0%	0.0000	0	-	-	-	-
			1	*-12.000 (-50.000, 26.000)*	-	0.0%	0.0000	0	-	-	-	-
		B	4	-29.392 (-76.887, 18.103)	0.426	0.0%	0.0000	0	-	-	-	-
			2	-1.169 (-71.808, 69.471)	0.756	0.0%	0.0000	0	-	-	-	-
			2	-52.432 (-132.786, 27.922	0.210	36.2%	1217.9903	0	-	-	-	-

	**Timing Category**<br>First trimester<br>Second trimester<br>Third trimester<br>Cord blood	A(a)	6<br>0<br>0<br>1<br>5	-18.483 (-57.186, 20.220)<br>-<br>-<br>*102.000 (-41.000, 245.000)*<br>-23.889 (-55.556, 7.779)	0.180<br>-<br>-<br>-<br>0.294	34.2%<br>-<br>-<br>0.0%<br>19.0%	708.7221<br>-<br>-<br>0.0000<br>277.0852	8<br>1<br>1<br>1<br>5	**-65.776 (-110.504, -21.048)**<br>*-123.700 (-284.000, 36.600)*<br>*-78.700 (-214.050, 56.650)*<br>*-114.400 (-316.150, 87.350)*<br>**-54.895 (-106.074, -3.716)**	0.978<br>-<br>-<br>-<br>0.954	0.0%<br>0.0%<br>0.0%<br>0.0%<br>0.0%	0.0000<br>0.0000<br>0.0000<br>0.0000<br>0.0000
		A(b)	1<br>0<br>1<br>0<br>0	*20.000 (-90.000, 50.000)*<br>-<br>*20.000 (-90.000, 50.000)*<br>-<br>-	-<br>-<br>-<br>-<br>-	0.0%<br>-<br>0.0%<br>-<br>-	0.0000<br>-<br>0.0000<br>-<br>-	0<br>0<br>0<br>0<br>0	-<br>-<br>-<br>-<br>-	-<br>-<br>-<br>-<br>-	-<br>-<br>-<br>-<br>-	-<br>-<br>-<br>-<br>-
		B	2<br>0	37.158 (-83.568, 157.883)	0.075<br>-	68.5%	5.4e+03	1<br>0	*5.500 (-116.900, 127.900)*	-<br>-	0.0%<br>-	0.0000<br>-

			1<br>0<br>1	-<br>*-13.000 (-71.500, 45.500)*<br>-<br>*112.800 (-12.650, 238.250)*	-<br>-<br>-	0.0%<br><br>0.0%	0.0000<br><br>0.0000	0<br>0<br>1	-<br>-<br>-<br>*5.500 (-116.900, 127.900)*	-<br>-<br>-	-<br>-<br>0.0%	-<br>-<br>0.0000
	**Study design**<br>Cross-sectional<br>Case-control<br>Cohort	A(a)	1<br>5<br>6<br>0<br>9	-0.082 (-0.374, 0.209)<br>-15.714 (-37.952, 6.524)<br>-<br>-0.074 (-0.320, 0.172)	0.019<br>0.222<br>-<br>0.029	48.3%<br>28.4%<br>-<br>53.1%	0.0411<br>199.4235<br>-<br>0.0285	10<br>3<br>0<br>7	-0.136 (-0.679, 0.406)<br>-56.550 (-117.101, 4.002)<br>-<br>-0.131 (-0.662, 0.401)	0.007<br>0.878<br>-<br>0.004	60.6%<br>0.0%<br>-<br>68.8%	0.1433<br>0.0000<br>-<br>0.1370
		A(b)	2<br>0<br>0<br>2	-60.550 (-158.964, 37.865)<br><br>-60.550 (-158.964, 37.865)	0.137<br><br>0.137	54.8%<br>-<br>-<br>54.8%	2.9e+03<br>-<br>-<br>2.9e+03	0<br>0<br>0<br>0	-<br>-<br>-<br>-	-<br>-<br>-<br>-	-<br>-<br>-<br>-	-<br>-<br>-<br>-
		B	3<br>0	-0.798 (-69.870, 68.274)	0.086<br>-	59.2%<br>-	2.1e+03<br>-	1<br>0	*5.500 (-116.900, 127.900)*	-<br>-	0.0%<br>-	0.0000<br>-

			0	-	-	-	-	0	-	-	0.0%	-
			3	-	0.086	59.2%	2.1e+03	1	-	-		0.0000
				-0.798 (-69.870,					*5.500 (-116.900,*			
				68.274)					*127.900)*			
			1	-0.082 (-0.374, 0.209)								
			5	-11.797 (-29.485,	0.019	48.3%	0.0411	10	-0.136 (-0.697, 0.406)	0.007	60.6%	0.1433
			2	5.890)	0.939	0.0%	0.0000	0	-	-	-	-
		A(a)	1	-0.079 (-0.363, 0.205)	0.015	53.0%	0.0390	10	-0.136 (-0.697, 0.406)	0.007	60.6%	0.1433
			2	-	-	-	-	0	-	-	-	-
	**Setting**											
			0	*102.000 (-41.000,*	-	0.0%	0.0000	0	-	-	-	-
	Europe											
			1	*245.000)*								
	Asia											
				-60.550 (-158.964,								
	North America											
			2	37.865)	0.137	54.8	2.9e+03	0	-	-	-	-
	Other											
			2	-60.550 (-158.964,	0.137	54.8	2.9e+03	0	-	-	-	-
		A(b)	0	37.865)	-	-	-	0	-	-	-	-
			0	-	-	-	-	0	-	-	-	-
			0	-	-	-	-	0	-	-	-	-
				-								

		B	3<br>2<br>1<br>0<br>0	-0.798 (-69.870, 68.274)<br>-28.150 (-71.175, 14.875)<br>*112.800 (-12.650, 238.250)*<br>-<br>-	0.86<br>0.454<br>-<br>-<br>-	59.2%<br>0.0%<br>0.0%<br>-<br>-	2134.0792<br>0.0000<br>0.0000<br>-<br>-	1<br>0<br>1<br>0<br>0	*5.500 (-116.900, 127.900)*<br>-<br>*5.500 (-116.900, 127.900)*<br>-<br>-	-<br>-<br>-<br>-	0.0%<br>-<br>0.0%<br>-<br>-	0.0000<br>-<br>0.0000<br>-<br>-
	**Matrix**<br>Serum<br>Plasma<br>Whole blood	A(a)	1<br>5<br>1<br>1<br>3<br>1	-0.082 (-0.374, 0.209)<br>-0.072 (-0.316, 0.172)<br>**-18.636 (-33.477, -3.796)**<br>*102.000 (-41.000, 245.000)*	0.019<br>0.041<br>0.908<br>-	48.3%<br>47.1%<br>0.0%<br>0.0%	0.0411<br>0.0280<br>0.0000<br>0.0000	10<br>8<br>2<br>0	-0.136 (-0.679, 0.406)<br>-0.118 (-0.598, 0.363)<br>-39.074 (-126.576, 48.428)<br>-	0.007<br>0.014<br>0.189<br>-	60.6%<br>60.3%<br>42.1%<br>-	0.1433<br>0.1102<br>2465.5960<br>-
		A(b)	2<br>2<br>0<br>0	-60.550 (-158.964, 37.8650)<br>-60.550 (-158.964, 37.8650)<br>-	0.137<br>0.137<br>-<br>-	54.8%<br>54.8%<br>-<br>-	2.9e+03<br>2.9e+03<br>-<br>-	0<br>0<br>0<br>0	-<br>-<br>-<br>-	-<br>-<br>-<br>-	-<br>-<br>-<br>-	-<br>-<br>-<br>-

				-								
		B	3<br>3<br>0<br>0	-0.798 (-69.870,<br>68.274)<br>-0.798 (-69.870,<br>68.274)<br>-<br>-	0.086<br>0.086<br>-<br>-	59.2%<br>59.2%<br>-<br>-	2.1e+03<br>2.1e+03<br>-<br>-	1<br>1<br>0<br>0	*5.500 (-116.900,*<br>*127.900)*<br>*5.500 (-116.900,*<br>*127.900)*<br>-<br>-	-<br>-<br>-<br>-	0.0%<br>0.0%<br>-<br>-	0.0000<br>0.0000<br>-<br>-
	**Adjusted for: GA**<br>Yes<br>No	A(a)	1<br>5<br>1<br>1<br>4	-0.082 (-0.374, 0.209)<br>**-15.741 (-25.886, -**<br>**5.595)**<br>-0.067 (-0283, 0.149)	0.019<br>0.616<br>0.021	48.3%<br>0.0%<br>69.2%	0.0411<br>0.0000<br>0.0212	10<br>6<br>4	-0.136 (-0.697, 0.406)<br>**-20.170 (-37.122, -**<br>**3.218)**<br>-0.114 (-0.588, 0.361)	0.007<br>0.539<br>0.004	60.6%<br>0.0%<br>77.5%	0.1433<br>0.0000<br>0.1072
		A(b)	2<br>2<br>0	-60.550 (-158.964,<br>37.865)<br>-60.550 (-158.964,<br>37.865)<br>-	0.137<br>0.137<br>-	54.8%<br>54.8%<br>-	2.9e+03<br>2.9e+03<br>-	0<br>0<br>0	-<br>-<br>-	-<br>-<br>-	-<br>-<br>-	-<br>-<br>-

		B	3<br>2<br>1	-0.798 (69.870, 68.274)<br>-28.150 (-71.175, 14.875)<br>*112.800 (-12.650, 238.25)*	0.086<br>0.454<br>-	59.2%<br>0.0%<br>0.0%	2134.0792<br>0.0000<br>0.0000	1<br>0<br>1	*5.500 (-116.900, 127.900)*<br>-<br>*5.500 (-116.900, 127.900)*	-<br>-<br>-	0.0%<br>-<br>0.05	0.0000<br>-<br>0.0000
		A(a)	1<br>5<br>9<br>6	-0.082 (-0.374, 0.209)<br>**-16.446 (-26.633, -6.260)**<br>-0.067 (-0.283, 0.149)	0.019<br>0.718<br>0.038	48.3%<br>0.0%<br>57.5%	0.0411<br>0.0000<br>0.0212	10<br>5<br>5	-0.136 (-0.679, 0.406)<br>**-21.573 (-41.107, -2.038)**<br>**-0.113 (-0.567, -0.341)**	0.007<br>0.397<br>0.010	60.6%<br>1.6%<br>70.1%	0.1433<br>29.9977<br>0.0971
	**Adjusted for :**<br>**Parity**<br>Yes<br>No	A(b)	2<br>0<br>2	-60.550 (-158.964, 37.865)<br>-<br>-60.550 (-158.964, 37.865)	0.137<br>-<br>0.137	54.8%<br>-<br>54.8%	2.9e+03<br>-<br>2.9e+03	0<br>0<br>0	-<br>-<br>-	-<br>-<br>-	-<br>-<br>-	-<br>-<br>-
		B	3<br>2<br>1	-0.798 (-69.870, 68.274)<br>23.806 (-130.675, 178.287)	0.086<br>0.027<br>-	59.2%<br>79.6%<br>0.0%	2134.0792<br>10035.4850<br>0.0000	1<br>1<br>0	*5.500 (-116.900, 127.900)*<br>*5.500 (-116.900, 127.900)*<br>-	-<br>-<br>-	0.0%<br>0.0%<br>-	0.0000<br>0.0000<br>-

				-13.000 (-71.500, 45.500)									
**GA(weeks)**	**Gender**	A(a)	2	0.088 (-0.075, 0.252)	0.639	0.0%	0.0000	2	0.050 (-0.173, 0.273)	0.860	0.0%	0.0000
	Male		1	*0.120 (-0.090, 0.330)*	-	0.0%	0.0000	1	*0.070 (-0.245, 0.385)*	-	0.0%	0.0000
	Female		1	*0.040 (-0.220, 0.300)*	-	0.0%	0.0000	1	*0.030 (-0.285, 0.345)*	-	0.0%	0.0000
	**Timing Category**	A(a)	2	0.067 (-0.011, 0.146)	0.709	0.0%	0.0000	2	0.078 (-0.017, 0.173)	0.936	0.0%	0.0000
	First trimester		0	-	-	-	-	0	-	-	-	-
	Second trimester		0	-	-		-	0	-	-		-
	Third trimester		1	*0.060 (-0.030, 0.150)*	-	0.0%	0.0000	1	*0.080 (-0.025, 0.185)*	-	0.0%	0.0000
	Cord blood		1	*0.090 (-0.070, 0.250)*	-	0.0%	0.0000	1	*0.070 (-0.150, 0.290)*	-	0.0%	0.0000
	**Study design**	A(a)	4	0.030 (-0.019, 0.080)	0.529	0.0%	0.0000	2	0.087 (-0.017, 0.173)	0.936	0.0%	0.0000
	Cross-sectional		2	0.031 (-0.033, 0.095)	0.432	0.0%	0.0000	1	*0.070 (-0.150, 0.290)*	-	0.0%	0.0000
	Case-control		0	-	-	-	-	0	-	-	-	-
	Cohort		2	0.020 (0.083, 0.124)	0.206	37.3%	0.0023	1	*0.080 (-0.025, 0.185)*	-	0.0%	0.0000
	**Setting**	A(a)	4	0.030 (-0.019, 0080)	0.529	0.0%	0.0000	2	0.078 (-0.017, 0.173)	0.936	0.0%	0.0000
	Europe		1	*0.020 (-0.050, 0.090)*	-	0.0%	0.0000	0	-	-	-	-
	Asia		3	0.040 (-0.030, 0.111)	0.360	2.2%	0.0001	2	0.078 (-0.017, 0.173)	0.936	0.0%	0.0000
	America		0	-	-	-	-	0	-	-	-	-
	Other		0	-	-	-	-	0	-	-	-	-

	**Matrix**	A(a)	4	0.030 (-0.019, 0.080)	0.529	0.0%	0.0000	2	0.078 (-0.017, 0.173)	0.936	0.0%	0.0000
	Serum		2	0.031 (-0.003, 0.095)	0.432	0.0%	0.0000	1	*0.070 (-0.150, 0.290)*	-	0.0%	0.0000
	Plasma		1	*-0.050 (-0.195, 0.095)*	-	0.0%	0.0000	0	-	-	-	-
	Whole blood		1	*0.060 (-0.030, 0.150)*	-	0.0%	0.0000	1	*0.080 (-0.025, 0.185)*	-	0.0%	0.0000
	**Adjusted for: GA**	A(a)	4	0.030 (-0.019, 0.080)	0.529	0.0%	0.0000	2	0.078 (-0.017, 0.173)	0.936	0.0%	0.0000
	Yes		3	0.024 (-0.027, 0.076)	0.0443	0.0%	0.0000	1	*0.080 (-0.025, 0.185)*	-	0.0%	0.0000
	No		1	*0.090 (-0.070, 0.250)*	-	0.0%	0.0000	1	*0.070 (-0.150, 0.290)*	-	0.0%	0.0000
	**Adjusted for: Parity**	A(a)	4	0.030 (-0.019, 0.080)	0.529	0.0%	0.0000	2	0.078 (-0.017, 0.173)	0.936	0.0%	0.0000
	Yes		4	0.030 (-0.019, 0.080)	0.529	0.0%	0.0000	2	0.078 (-0.017, 0.173)	0.936	0.0%	0.0000
	No		0	-	-	-	-	0	-	-	-	-
**BL/cm**	**Gender**	A(a)	2	-0.185 (-0.430, 0.060)	0.264	19.9%	0.0062	2	-0.141 (-0.568, 0.287)	0.269	18.1%	0.0175
	Male		1	*-0.727 (-1.510, 0.056)*	-	0.0%	0.0000	1	*0.050 (-0.455, 0.555)*	-	0.0%	0.0000
	Female		1	*0.591 (-0.196, 1.377)*	-	0.0%	0.0000	1	*-0.390 (-0.985, 0.205)*	-	0.0%	0.0000
	**Timing Category**	A(a)	2	-0.030 (-0.203, 0.136)	0.294	9.3%	0.0000	4	**-0.172 (-0.279, -0.064)**	0.582	0.0%	0.0000
	First trimester		0	-	-	-	-	1	***-0.147 (-0.272, -0.022)***	-	0.0%	0.0000
	Second trimester		0	-	-	-	-	1	*-0.300 (-0.690, 0.090)*	-	0.0%	0.0000
	Third trimester		1	*0.320 (-0.375, 1.015)*	-	0.0%	0.0000	1	*-0.520 (-1.105, 0.065)*	-	0.0%	0.0000
	Cord blood		1	*-0.054 (-0.128, 0.019)*	-	0.0%	0.0000	1	*-0.150 (-0.430, 0.130)*	-	0.0%	0.0000

			1	***0.410 (0.055, 0.765)***	-	0.0%	0.0000	1	*-0.120 (-0.465, 0.225)*	-	0.0%	0.0000
			0	-	-	-	-	0	-	-	-	-
		B	0	-	-	-	-	0	-	-	-	-
			0	-	-	-	-	0	-	-	-	-
			1	***0.410 (0.055, 0.765)***	-	0.0%	0.0000	1	*-0.120 (-0.465, 0.225)*	-	0.0%	0.0000
			7	-0.051 (-0.122, 0.020)	0.235	25.4%	0.0021	4	**-0.148 (-0.261, -0.036)**	0.742	0.0%	0.0000
		A(a)	4	-0.078 (-0.200, 0.044)	0.301	18.0%	0.0041	2	-0.141 (-0.568, 0.287)	0.269	18.1%	0.0175
			0	-	-	-	-	0	-	-	-	-
			3	-0.042 (-0.164, 0.081)	0.157	46.0%	0.0054	2	**-0.150 (-0.268, -0.032)**	0.887	0.0%	0.0000
	**Study design**		1	*0.000 (-0.682, 0.682)*	-	0.0%	0.0000	0	-	-	-	-
	Cross-sectional	A(b)	0	-	-	-	-	0	-	-	-	-
	Case-control		0	-	-	-	-	0	-	-	-	-
	Cohort		1	*0.000 (-0.682, 0.682)*	-	0.0%	0.0000	0	-	-	-	-
			1	*0.410 (0.055, 0.765)*	-	0.0%	0.0000	1	*-0.120 (-0.465, 0.225)*	-	0.0%	0.0000
		B	0	-	-	-	-	0	-	-	-	-
			0	-	-	-	-	0	-	-	-	-
			1	*0.410 (0.055, 0.765)*	-	0.0%	0.0000	1	*-0.120 (-0.465, 0.225)*	-	0.0%	0.0000
	**Setting**	A(a)	7	-0.051 (-0.122, 0.020)	0.235	25.4%	0.0021	4	**-0.148 (-0.261, -0.036)**	0.742	0.0%	0.0000
	Europe		0	-	-	-	-	0	-		-	-

	Asia		6	-0.055 (-0.126, 0.017)	0.222	28.4%	0.0021	4	**-0.148 (-0.261, -0.036)**	0.742	0.0%	0.0000
	North America		0	-	-	-	-	0	-		-	-
	Other		1	*0.320 (-0.375, 1.015)*	-	0.0%	0.0000	0	-		-	-
			1	*0.000 (-0.682, 0.682)*	-	0.0%	0.0000	0	-	-	-	-
			1	*0.000 (-0.682, 0.682)*	-	0.0%	0.0000	0	-	-	-	-
		A(b)	0	-	-	-	-	0	-	-	-	-
			0	-	-	-	-	0	-	-	-	-
			0	-	-	-	-	0	-	-	-	-
			1	*0.410 (0.055, 0.765)*	-	0.0%	0.0000	1	*-0.120 (-0.465, 0.225)*	-	0.0%	0.0000
			0	-	-	-	-	0	-	-	-	-
		B	1	*0.410 (0.055, 0.765)*	-	0.0%	0.0000	1	*-0.120 (-0.465, 0.225)*	-	0.0%	0.0000
			0	-	-	-	-	0	-	-	-	-
			0	-	-	-	-	0	-	-	-	-
			7	-0.051 (-0.122, 0.020)	0.235	25.4%	0.0021	4	**-0.148 (-0.261, -0.036)**	0.742	0.0%	0.0000
	**Matrix**	A(a)	3	-0.087 (-0.202, 0.029)	0.290	19.2%-	0.0032	2	-0.141 (-0.568, 0.287)	0.269	18.1%	0.0175
	Serum		3	-0.042 (-0.164, 0.081)	0.157	46.0%	0.0054	2	**-0.150 (-0.268, -0.032)**	0.887	0.0%	0.0000
	Plasma		1	*0.320 (-0.375, 1.015)*	-	0.0%	0.0000	0	-	-	-	0.0000
	Whole blood	A(b)	1	*0.000 (-0.682, 0.682)*	-	0.0%	0.0000	0	-	-	-	-
			1	*0.000 (-0.682, 0.682)*	-	0.0%	0.0000	0	-	-	-	-

			0	-	-	-	-	0	-	-	-	-
			0	-	-	-	-	0	-	-	-	-
			1	***0.410 (0.055, 0.765)***	-	0.0%	0.0000	1	*-0.120 (-0.465, 0.225)*	-	0.0%	0.0000
		B	1	***0.410 (0.055, 0.765)***	-	0.0%	0.0000	1	*-0.120 (-0.465, 0.225)*	-	0.0%	0.0000
			0		-	-	-	0		-	-	-
			0	-	-	-	-	0	-	-	-	-
			7	-0.051 (-0.122, 0.020)	0.235	25.4%	0.0021	4	**-0.148 (-0.261, -0.036)**	0.742	0.0%	0.0000
		A(a)	7	-0.051 (-0.122, 0.020)	0.235	25.4%	0.0021	4	**-0.148 (-0.261, -0.036)**	0.742	0.0%	0.0000
			0	-	-	-	-	0	-	-	-	-
	**Adjusted for: GA**		1	*0.000 (-0.682, 0.682)*	-	0.0%	0.0000	0	-	-	-	-
	Yes	A(b)	1	*0.000 (-0.682, 0.682)*	-	0.0%	0.0000	0	-	-	-	-
	No		0	-	-	-	-	0	-	-	-	-
			1	***0.410 (0.055, 0.765)***	-	0.0%	0.0000	1	*-0.120 (-0.465, 0.225)*	-	0.0%	0.0000
		B	0	-	-	-	-	0	-	-	-	-
			1	***0.410 (0.055, 0.765)***	-	0.0%	0.0000	1	*-0.120 (-0.465, 0.225)*	-	0.0%	0.0000
	**Adjusted for :**		7	-0.051 (-0.122, 0.020)	0.235	25.4%	0.0021	4	**-0.148 (-0.261, -0.036)**	0.742	0.0%	0.0000
	**Parity**	A(a)	6	-0.055 (-0.126, 0.017)	0.222	28.4%	0.0021	4	**-0.148 (-0.261, -0.036)**	0.742	0.0%	0.0000
	Yes		1	*0.320 (-0.375, 1.015)*	-	0.0%	0.0000	0	-	-	-	-

	No		1	*0.000 (-0.682, 0.682)*	-	0.0%	0.0000	0	-	-	-	-
		A(b)	0	-	-	-	-	0	-	-	-	-
			1	*0.000 (-0.682, 0.682)*	-	0.0%	0.0000	0	-	-	-	-
			1	***0.410 (0.055, 0.765)***	-	0.0%	0.0000	1	*-0.120 (-0.465, 0.225)*	-	0.0%	0.0000
		B	1	***0.410 (0.055, 0.765)***	-	0.0%	0.0000	1	*-0.120 (-0.465, 0.225)*	-	0.0%	0.0000
			0	-	-	-	-	0	-	-	-	-
**HC/cm**	**Gender**		2	-0.097 (-0.229, 0.035)	0.555	0.0%	0.0000	2	-0.198(-0.520, 0.124)	0.165	48.2%	0.0263
	Male	A(a)	1	*-0.060 (-0.240, 0.120)*	-	0.0%	0.0000	1	*-0.050 (-0.345, 0.245)*	-	0.0%	0.0000
	Female		1	*-0.140 (-0.335, 0.055)*	-	0.0%	0.0000	1	***-0.380 (-0.740, -0.020)***	-	0.0%	0.0000
	**Timing Category**		0	-	-	-	-	4	-25.495 (-66.044, 15.055)	0.613-	0.0%	0.0000
	First trimester		0	-	-	-	-	1	*-8.100 (-107.950, 91.750)*	-	0.0%	0.0000
	Second trimester	A(a)	0	-	-	-	-	1	*13.200 (-70.100, 96.500)*	-	0.0%	0.0000
	Third trimester		0	-	-	-	-	1	*-15.000 (-136.700, 106.700)*	-	0.0%	0.0000
	Cord blood		0	-	-	-	-	1	*-52.600 (-110.700, 5.500)*		0.0%	0.0000

	**Study design**<br>Cross-sectional<br>Case-control<br>Cohort	A(a)	4	-0.058 (-0.124, 0.009)	0.856	0.0%	0.0000	4	**-0.08 (-0.164, -0.006)**	0.431	0.0%	0.0000
			1	*-0.290 (-0.815, 0.235)*	-	0.0%	0.0000	0	-	-	-	-
			0	-	-	-	-	0	-	-	-	-
			3	-0.054 (-0.121, 0.013)	0.997	0.0%	0.0000	4	**-0.08 (-0.164, -0.006)**	0.431	0.0%	0.0000
		A(b)	1	*0.000 (-0.682, 0.682)*	-	0.0%	0.0000	0	-	-	-	-
			0	-	-	-	-	0	-	-	-	-
			0	-	-	-	-	0	-	-	-	-
			1	*0.000 (-0.682, 0.682)*	-	0.0%	0.0000	0	-	-	-	-
	**Setting**<br>Europe<br>Asia<br>America<br>Other	A(a)	4	-0.058 (-0.124, 0.009)	0.856	0.0%	0.0000	4	**-0.085 (-0.164, -0.006)**	0.431	0.0%	0.0000
			0	-	-		-	0	-	-	-	-
			3	-0.054 (-0.121, 0.013)	0.997	0.0-%	0.0000	4	**-0.085 (-0.164, -0.006)**	0.431	0.0-%	0.0000
			0	-	-	-	-	0	-	-	-	-
			1	*-0.290 (-0.815, 0.235)*	-	0.0%	0.0000	0	-	-	-	-
		A(b)	1	*0.000 (-0.682, 0.682)*	-	0.0%	0.0000	0	-	-	-	-
			1	*0.000 (-0.682, 0.682)*	-	0.0%	0.0000	0	-	-	-	-
			0	-	-	-	-	0	-	-	-	-
			0	-	-	-	-	0	-	-	-	-
			0	-	-	-	-	0	-	-	-	-

	**Matrix**<br>Serum<br>Plasma<br>Whole blood	A(a)	4	-0.058 (-0.124, 0.009)	0.856	0.0%	0.0000	4	**-0.085 (-0.164, -0.006)**	0.431	0.0%	0.0000
			0	-	-	0.0%	0.0000	2	-0.198 (-0.520, 0.124)	0.165	48.2%	0.0263
			3	-0.054 (-0.121, 0.013)	0.997	0.0%	0.0000	2	-0.072 (-0.156, 0.012)	0.875	0.0%	0.0000
			1	*-0.290 (-0.815, 0.235)*	-	0.0%	0.0000	0	-	-	-	-
		A(b)	1	*0.000 (-0.682, 0.682)*	-	0.0%	0.0000	0	-	-	-	-
			1	*0.000 (-0.682, 0.682)*	-	0.0%	0.0000	0	-	-	-	-
			0	-	-	-	-	0	-	-	-	-
			0	-	-	-	-	0	-	-	-	-
	**Adjusted for: GA**<br>Yes<br>No	A(a)	4	-0.058 (-0.124, 0.009)	0.856	0.0%	0.0000	4	**-0.085 (-0.164, -0.006)**	0.431	0.0%	0.0000
			4	-0.058 (-0.124, 0.009)	0.856	0.0%	0.0000	2	-0.072 (-0.156, 0.012)	0.875	0.0%	0.0000
			0	-	-	-	-	2	-0.198 (-0.520, 0.124)	0.165	48.2%	0.0263
		A(b)	1	*0.000 (-0.682, 0.682)*	-	0.0%	0.0000	0	-	-	-	-
			1	*0.000 (-0.682, 0.682)*	-	0.0%	0.0000	0	-	-	-	-
			0	-	-	-	-	0	-	-	-	-
	**Adjusted for: Parity**<br>Yes<br>No	A(a)	4	-0.058 (-0.124, 0.009)	0.856	0.0%	0.0000	4	**-0.085 (-0.164, -0.006)**	0.431	0.0%	0.0000
			3	-0.054 (-0.121, 0.013)	0.997	0.0%	0.0000	2	-0.072 (-0.156, 0.012)	0.875	0.0%	0.0000
			1	*-0.290 (-0.815, 0.235)*	-	0.0%	0.0000	2	-0.198 (-0.520, 0.124)	0.165	48.2%	0.0263
		A(b)	1	*0.000 (-0.682, 0.682)*	-	0.0%	0.0000	0	-	-	-	-
			0	-	-	-	-	0	-	-	-	-

			1	*0.000 (-0.682, 0.682)*	-	0.0%	0.0000	0	-	-	-	-
			3	0.001 (-0.008, 0.010)	0.910	0.0%	0.0000	1	*0.000 (-0.004, 0.004)*	-	0.0%	0.0000
			1	*0.010 (-0.090, 0.110)*	-	-	-	0	-	-	-	-
		A(a)	0	-	-	-	-	0	-	-	-	-
			0	-	-	0.0%	0.0000	0	-	-	-	-
			2	0.001 (-0.008, 0.010)	0.693	0.0%	0.0000	1	*0.000 (-0.004, 0.004)*	-	0.0%	0.0000
	**Timing Category**		1	*0.0 (-0.150, 0.150)*	-	0.0%	0.0000	0	-	-	-	-
	First trimester		0		-	-	-	0	-	-	-	-
**PI/ g.cm3**	Second trimester	A(b)	1	*0.000 (-0.150, 0.150)*	-	0.0%	0.0000	0	-	-	-	-
	Third trimester		0	-	-	-	-	0	-	-	-	-
	Cord blood		0	-	-	-	-	0	-	-	-	-
			2	0.020 (-0.059, 0.098)	0.702	0.0%	0.0000	1	*0.020 (-0.370, 0.410)*	-	0.0%	0.0000
			0	-	-	-	-	0	-	-	-	-
		B	1	*-0.003 (-0.143, 0.137)*	-	0.0%	0.0000	0	-	-	-	-
			0	-	-	-	-	0	-	-	-	-
			1	*0.030 (-0.065, 0.125)*	-	0.0%	0.0000	1	*0.020 (-0.370, 0.410)*	-	0.0%	0.0000
	**Study design**	A(a)	4	-0.002 (-0.010, 0.007)	0.721	0.0%	0.0000	1	*0.000 (-0.004, 0.004)*	-	0.0%	0.0000
	Cross-sectional		2	0.003 (-0.011, 0.016)	0.888	0.0%	0.0000	0	-	-	-	-

	Case-control		0	-	-	-	-	0	-	-	-	-
	Cohort		2	-0.004 (-0.014, 0.006)	0.412	0.0%	0.0000	1	*0.000 (-0.004, 0.004)*	-	0.0%	0.0000
		A(b)	1	*0.000 (-0.150, 0.150)*	-	0.0%	0.0000	0	-	-	-	-
			0	-	-	-	-	0	-	-	-	-
			0	-	-	-	-	0	-	-	-	-
			1	*0.000 (-0.150, 0.150)*	-	0.0%	0.0000	0	-	-	-	-
		B	2	0.020 (-0.059, 0.098)	0.702	0.0%	0.0000	1	*0.020 (-0.370, 0.410)*	-	0.0%	0.0000
			0	-	-	-	-	0	-	-	-	-
			0	-	-	-	-	0	-	-	-	-
			2	0.020 (-0.059, 0.098)	0.702	0.0%	0.0000	1	*0.020 (-0.370, 0.410)*	-	0.0%	0.0000
		A(a)	4	-0.002 (-0.010, 0.007)	0.721	0.0%	0.0000	1	*0.000 (-0.004, 0.004)*	-	0.0%	0.0000
			0	-	-	-	-	0	-	-	-	-
	**Setting**		3	-0.002 (-0.010, 0.007)	0.527	0.0%	0.0000	1	*0.000 (-0.004, 0.004)*	-	0.0%	0.0000
	Europe		0	-	-	-	-	0	-	-	-	-
	Asia		1	*0.010 (-0.090, 0.110)*	-	0.0%	0.0000	0	-	-	-	-
	North America	A(b)	1	*0.000 (-0.150, 0.150)*	-	0.0%	0.0000	0	-	-	-	-
	Other		1	*0.000 (-0.150, 0.150)*	-	0.0%	0.0000	0	-	-	-	-
			0	-	-	-	-	0	-	-	-	-
			0	-	-	-	-	0	-	-	-	-

			0	-	-	-	-	0	-	-	-	-
			2	0.020 (-0.059, 0.098)	0.702	0.0%	0.0000	1	*0.020 (-0.670, 0.110)*	-	0.0%	0.0000
			1	*-0.003 (-0.170, 0.110)*	-	0.0%	0.0000	0	-	-	-	-
		B	1	*0.030 (-0.070, 0.120)*	-	0.0%	0.0000	1	*0.020 (-0.670, 0.110)*	-	0.0%	0.0000
			0	-	-	-	-	0	-	-	-	-
			0	-	-	-	-	0	-	-	-	-
			4	-0.002 (-0.010, 0.007)	0.721	0.0%	0.0000	1	*0.000 (-0.004, 0.004)*	-	0.0%	0.0000
		A(a)	2	0.001 (-0.008, 0.010)	0.693	0.0%	0.0000	1	*0.000 (-0.004, 0.004)*	-	0.0%	0.0000
			1	*-0.010 (-0.027, 0.007)*	-	-	-	0	-	-	-	-
			1	*0.010 (-0.090, 0.110)*	-	-	-	0	-	-	-	-
	**Matrix**		1	*0.000 (-0.200, 0.100)*	-	0.0%	0.0000	0	-	-	-	-
	Serum	A(b)	1	*0.000 (-0.200, 0.100)*	-	0.0%	0.0000	0	-	-	-	-
	Plasma		0	-	-	-	-	0	-	-	-	-
	Whole blood		0	-	-	-	-	0	-	-	-	-
			2	0.020 (-0.059, 0.098)	0.702	0.0%	0.0000	1	*0.020 (-0.670, 0.110)*	-	0.0%	0.0000
		B	2	0.020 (-0.059, 0.098)	0.702	0.0%	0.0000	1	*0.020 (-0.670, 0.110)*	-	0.0%	0.0000
			0	-	-	-	-	0	-	-	-	-
			0	-	-	-	-	0	-	-	-	-

			4	-0.002 (-0.010, 0.007)	0.721	0.0%	0.0000	1	*0.000 (-0.004, 0.004)*	-	0.0%	0.0000
		A(a)	4	-0.002 (-0.010, 0.007)	0.721	0.0%	0.0000	1	*0.000 (-0.004, 0.004)*	-	0.0%	0.0000
			0	-	-	-	-	0	-	-	-	-
	**Adjusted for: GA**		1	*0.000 (-0.200, 0.100)*	-	0.0%	0.0000	0	-	-	-	-
	Yes	A(b)	1	*0.000 (-0.200, 0.100)*	-	0.0%	0.0000	0	-	-	-	-
	No		0	-	-	-	-	0	-	-	-	-
			2	0.020 (-0.059, 0.098)	0.721	0.0%	0.0000	1	*0.020 (-0.670, 0.110)*	-	0.0%	0.0000
		B	1	*-0.003 (-0.170, 0.110)*	-	0.0%	0.0000	0	-	-	-	-
			1	*0.030 (-0.070, 0.120)*	-	0.0%	0.0000	1	*0.020 (-0.670, 0.110)*	-	0.0%	0.0000
			4	-0.002 (-0.010, 0.007)	0.721	0.0%	0.0000	1	*0.000 (-0.004, 0.004)*	-	0.0%	0.0000
		A(a)	3	-0.002 (-0.010, 0.007)	0.527	0.0%	0.0000	1	*0.000 (-0.004, 0.004)*	-	0.0%	0.0000
			1	*0.010 (-0.090, 0.110)*	-	0.0%	0.0000	0	-	-	-	-
	**Adjusted for:**		1	*0.000 (-0.200, 0.100)*	-	0.0%	0.0000	0	-	-	-	-
	**Parity**	A(b)	0	-	-	-	-	0	-	-	-	-
	Yes		1	*0.000 (-0.200, 0.100)*	-	0.0%	0.0000	0	-	-	-	-
	No		2	0.020 (-0.059, 0.098)	0.721	0.0%	0.0000	1	*0.020 (-0.670, 0.110)*	-	0.0%	0.0000
		B	1	*0.030 (-0.070, 0.120)*	-	0.0%	0.0000	1	*0.020 (-0.670, 0.110)*	-	0.0%	0.0000
			1	*-0.003 (-0.170, 0.110)*	-	0.0%	0.0000	0	-	-	-	-

PTB	**Gender**	A(a)	2	0.822 (0.610, 1.106)	0.937	0.0%	0.0000	2	0.722 (0.504, 1.034)	0.495	0.0%	0.0000
	Male		1	*0.830 (1.560, 1.220)*	-	0.0%	0.0000	1	*0.810 (0.500, 1.330)*	-	0.0%	0.0000
	Female		1	*0.810 (0.510, 1.280)*	-	0.0%	0.0000	1	*0.630 (0.370, 1.070)*	-	0.0%	0.0000
	**Timing Category**	A(a)	1	*0.820 (0.610, 1.100)*	-	0.0%	0.0000	1	*0.730 (0.510, 1.040)*	-	0.0%	0.0000
	First trimester		0	-	-	-	-	0	-	-	-	-
	Second trimester		0	-	-	-	-	0	-	-	-	-
	Third trimester		1	*0.820 (0.610, 1.100)*	-	0.0%	0.0000	1	*0.730 (0.510, 1.040)*	-	0.0%	0.0000
	Cord blood		0	-	-	-	-	0	-	-	-	-
	**Study design**	A(a)	2	0.844 (0.685, 1.041)	0.782	0.0%	0.0000	1	*0.730 (0.510, 1.040)*	-	0.0%	0.0000
	Cross-sectional		0		-	-	-	0	-	-	-	-
	Case-control		0		-	-	-	0	-	-	-	-
	Cohort		2	0.844 (0.685, 1.041)	0.782	0.0%	0.0000	1	*0.730 (0.510, 1.040)*	-	0.0%	0.0000
	**Setting**	A(a)	2	*0.844 (0.685, 1.041)*	0.782	0.0%	0.0000	1	*0.730 (0.510, 1.040)*	0.782	0.0%	0.0000
	Europe		0	-	-	-	-	0	-	-	-	-
	Asia		2	*0.844 (0.685, 1.041)*	0.782	0.0%	0.0000	1	*0.730 (0.510, 1.040)*	0.782	0.0%	0.0000
	North America		0	-	-	-	-	0	-	-	-	-
	Other		0	-	-	-	-	0	-	-	-	-
	**Matrix**	A(a)	2	*0.844 (0.685, 1.041)*	0.782	0.0%	0.0000	1	*0.730 (0.510, 1.040)*	-	0.0%	0.0000
	Serum		0	-	-	-	-	0	-	-	-	-

	Plasma		1	*0.870 (0.640, 1.160)*	-	0.0%	0.0000	0	-	-	-	-
	Whole blood		1	*0.820 (0.610, 1.100)*	-	0.0%	0.0000	1	*0.730 (0.510, 1.040)*	-	0.0%	0.0000
	**Adjusted for: GA**		2	0.844 (0.685, 1.041)	0.782	0.0%	0.0000	1	*0.730 (0.510, 1.040)*	-	0.0%	0.0000
	Yes	A(a)	2	0.844 (0.685, 1.041)	0.782	0.0%	0.0000	1	*0.730 (0.510, 1.040)*	-	0.0%	0.0000
	No		0		-	-	-	0	-	-	-	-
	**Adjusted for:**											
	**Parity**		2	0.844 (0.685, 1.041)	0.782	0.0%	0.0000	1	*0.730 (0.510, 1.040)*	-	0.0%	0.0000
		A(a)	2	0.844 (0.685, 1.041)	0.782	0.0%	0.0000	1	*0.730 (0.510, 1.040)*	-	0.0%	0.0000
	Yes		0		-	-	-	0	-	-	-	-
	No											
	**Study design**		1	*1.010 (0.530, 1.910)*	-	0.0%	0.0000	0	-	-	-	-
	Cross-sectional		0	-	-	-	-	0	-	-	-	-
	Case-control	A(a)	0	-	-	-	-	0	-	-	-	-
	Cohort		1	*1.010 (0.530, 1.910)*	-	0.0%	0.0000	0	-	-	-	-
**LBW**	**Setting**		1	*1.010 (0.530, 1.910)*	-	0.0%	0.0000	0	-	-	-	-
	Europe		0	-	-	-	-	0	-	-	-	-
	Asia	A(a)	1	*1.010 (0.530, 1.910)*	-	0.0%	0.0000	0	-	-	-	-
	North America		0	-	-	-	-	0	-	-	-	-
	Other		0	-	-	-	-	0	-	-	-	-

	**Matrix**	A(a)	1	*1.010 (0.530, 1.910)*	-	0.0%	0.0000	0	-	-	-	-
	Serum		0	-	-	-	-	0	-	-	-	-
	Plasma		1	*1.010 (0.530, 1.910)*	-	0.0%	0.0000	0	-	-	-	-
	Whole blood		0	-	-	-	-	0	-	-	-	-
	**Adjusted for: GA**	A(a)	1	*1.010 (0.530, 1.910)*	-	0.0%	0.0000	0	-	-	-	-
	Yes		1	*1.010 (0.530, 1.910)*	-	0.0%	0.0000	0	-	-	-	-
	No		0	-	-	-	-	0	-	-	-	-
	**Adjusted for: Parity**	A(a)	1	*1.010 (0.530, 1.910)*	-	0.0%	0.0000	0	-	-	-	-
	Yes		1	*1.010 (0.530, 1.910)*	-	0.0%	0.0000	0	-	-	-	-
	No		0	-	-	-	-	0	-	-	-	-
**SGA**	**Gender**	A(a)	4	1.157 (0.873, 1.532)	0.170	40.3%	0.0329	2	1.138 (0.464, 2.788)	0.115	59.7%	0.2589
	Male		2	1.048 (0.713, 1.541)	0.152	51.4%	0.0684	1	*0.780 (0.430, 1.420)*	-	0.0%	0.0000
	Female		2	1.350 (0.810, 2.249)	0.161	49.2%	0.0399	1	*1.980 (0.730, 5.330)*	-	0.0%	0.0000
		B	2	1.535 (0.956, 2.464)	0.495	0.0%	0.0000	0	-	-	-	-
			1	*1.360 (0.760, 2.460)*	-	0.0%	0.0000	0	-	-	-	-
			1	*1.920 (0.860, 4.250)*	-	0.0%	0.0000	0	-	-	-	-
	**Study design**	A(a)	4	1.104 (0.849, 1.434)	0.114	49.6%	0.0346	2	1.138 (0.464, 2.788)	0.115	59.7%	0.2589
	Cross-sectional		3	1.184 (0.835, 1.677)	0.086	59.2%	0.0550	2	1.138 (0.464, 2.788)	0.115	59.7%	0.2589

	Case-control		0	-	-	-	-	0	-	-	-	-
	Cohort		1	*0.930 (0.150, 1.330)*	-	0.0%	0.0000	0	-	-	-	-
		B	1	*1.520 (0.950, 2.440)*	-	0.0%	0.0000	0	-	-	-	-
			0	-	-	-	-	0	-	-	-	-
			0	-	-	-	-	0	-	-	-	-
			1	*1.520 (0.950, 2.440)*	-	0.0%	0.0000	0	-	-	-	-
	**Setting**<br>Europe<br>Asia<br>North America<br>Other	A(a)	4	1.104 (0.849, 1.434)	0.114	49.6%	0.0346	4	1.138 (0.464, 2.788)	0.115	59.7%	0.2589
			0	-	-	-	-	0	-	-	-	-
			4	1.104 (0.849, 1.434)	0.114	49.6%	0.0346	4	1.138 (0.464, 2.788)	0.115	59.7%	0.2589
			0	-	-	-	-	0	-	-	-	-
			0	-	-	-	-	0	-	-	-	-
		B	1	*1.520 (0.950, 2.440)*	-	0.0%-	0.0000	0	-	-	-	-
			1	*1.520 (0.950, 2.440)*	-	0.0%	0.0000	0	-	-	-	-
			0	-	-	-	-	0	-	-	-	-
			0	-	-	-	-	0	-	-	-	-
			0	-	-	-	-	0	-	-	-	-
	**Matrix**	A(a)	4	1.104 (0.849, 1.434)	0.114	49.6%	0.0346	2	1.138 (0.464, 2.788)	0.115	59.7%	0.2589-
	Serum		3	1.184 (0.835, 1.6770	0.086	59.2%	0.0550	2	1.138 (0.464, 2.788)	0.115	59.7%	0.2589
	Plasma		1	*0.930 (0.650, 1.330)*	-	0.0%	0.0000	0	-	-	-	-

	Whole blood		0	-	-	-	-	0	-	-	-	-
			1	*1.520 (0.950, 2.440)*	-	0.0%	0.0000	0	-	-	-	-
		B	1	*1.520 (0.950, 2.440)*	-	0.0%	0.0000	0	-	-	-	-
			0	-	-	-	-	0	-	-	-	-
			0	-	-	-	-	0	-	-	-	-
			4	1.104 (0.849, 1.434)	0.114	49.6%	0.0346	2	1.138 (0.464, 2.788)	0.115	59.7%	0.2589-
	**Adjusted for: GA**	A(a)	4	1.104 (0.849, 1.434)	0.114	49.6%	0.0346	2	1.138 (0.464, 2.788)	0.115	59.7%	0.2589
	Yes		0	-	-	-	-	0	-	-	-	-
	No		1	*1.520 (0.950, 2.440)*	-	0.0%	0.0000	0	-	-	-	-
		B	1	*1.520 (0.950, 2.440)*	-	0.0%	0.0000	0	-	-	-	-
			0	-	-	-	-	0	-	-	-	-
			4	1.104 (0.849, 1.434)	0.114	49.6%	0.0346	2	1.138 (0.464, 2.788)	0.115	59.7%	0.2589-
	**Adjusted for:**	A(a)	4	1.104 (0.849, 1.434)	0.114	49.6%	0.0346	2	1.138 (0.464, 2.788)	0.115	59.7%	0.2589
	**Parity**		0	-	-	-	-	0	-	-	-	-
	Yes		1	*1.520 (0.950, 2.440)*	-	0.0%	0.0000	0	-	-	-	-
	No	B	1	*1.520 (0.950, 2.440)*	-	0.0%	0.0000	0	-	-	-	-
			0	-	-	-	-	0	-	-	-	-

Birth Outcome indicators	Study characteristics		N	Summary β or OR (95% CI)	P value	I-squared	Tau-squared	N	Summary β or OR (95% CI)	P value	I-squared	Tau-squared
			PFHxS					PFNA				
BW/g	**Gender**<br>Male<br>Female	A(a)	1<br>2<br>6<br>6	-0.101(-0.385,0.182)<br>-0.149(-0.609,0.311)<br>-0.071(-0.431,0.288)	0.927<br>0.631<br>0.906	0.0%<br>0.0%<br>0.0%	0.000<br>0.000<br>0.000	14<br>7<br>7	-0.069(-0.170,0.032)<br>-0.175(-0.356,0.007)<br>0.013(-0.077,0.104)	0.087<br>0.197<br>0.375	36.1%<br>30.2%<br>6.9%	0.008<br>0.014<br>0.001
		A(b)	6<br>3<br>3	-0.006(-0.051,0.040)<br>-21.399(-74.019,31.340)<br>-0.006(-0.051,0.040)	0.264<br>0.098<br>0.403	56.9%<br>0.0%<br>22.6%	0.001<br>1290.327<br>0.000	**5**<br>3<br>2	**-0.060(-0.112,-0.009)**<br>18.886(-47.912,85.684)<br>-0.055(-0.120,0.011)	**0.370**<br>0.125<br>0.821	**6.4%**<br>51.9%<br>0.0%	**0.000**<br>1777.163<br>0.000
		B	5<br>3<br>2	-<br>20.383(065.440,24.674)<br>-18.136(-78.479,42.207)<br>-20.383(-65.440,24.674)	0.964<br>0.774<br>0.799	0.0%<br>0.0%<br>0.0%	0.000<br>0.000<br>0.000	5<br>3<br>2	-33.211(-84.828,08.406)<br>-21.267(-<br>115.920,73.385)<br>-51.621(-<br>114.959,11.717)	0.263<br>0.113<br>0.632	23.8%<br>54.1%<br>0.0%	825.906<br>3776.845<br>0
	**Timing Category**<br>First trimester<br>Second trimester<br>Third trimester<br>Cord blood	A(a)	1<br>2<br>3<br>*1*<br>2<br>6	-6.207(-23.294,10.880)<br>-4.225(-28.281,19.830)<br>*13.000(-55.300,81.300)*<br>-36.832(-<br>149.490,75.826)<br>-12.338(-45.373,20.697)	0.538<br>0.687<br>-<br>0.113<br>0.307	0.0%<br>0.0%<br>-<br>60.2%<br>16.6%	0.000<br>0.000<br>*0.000*<br>287.18915<br>4057.412	12<br>3<br>*1*<br>2<br>6	-6.207(-23.294,10.880)<br>-4.225(-28.281,19.830)<br>*-6.201(-23.294,10.880)*<br>-36.832(-<br>149.490,75.826)<br>-12.338(-45.373,20.697)	0.538<br>0.687<br>-<br>0.113<br>0.307	0.0%<br>0.0%<br>-<br>60.2%<br>16.6%	0.000<br>0.000<br>*0.000*<br>4057.413<br>287.189
		A(b)	*1*<br>*0*<br>*1*<br>*0*	*-50.000(-<br>110.000,10.000)*<br>-	-	-	-	2<br>*1*<br>*1*<br>0	-14.390(-82.337,53.557)<br>*-49.337(-95.955,-2.718)*<br>*20.000(-20.000,70.000)*<br>-	0.036<br>-<br>-<br>-	77.3%<br>-<br>-<br>-	1.9e+03<br>-<br>-<br>-

			0	*-50.000(-110.000,10.000)*<br>-<br>-				0	-	-	-	-
		B	5<br>1<br>3<br>0<br>*1*	-0.142(-0.343,0.059)<br>8.600(-59.700,76.900)<br>-0.144(-0.399,0.112)<br>-<br>*-25.100(-149.100,98.900)*	-<br>0.594<br>-<br>-<br>*0.277*	0.0%<br>-<br>22.1%<br>-<br>-	0.0%<br>0.0%<br>0.013%<br>-<br>*0.0%*	5<br>*1*<br>3<br>0<br>*1*	-0.108(-0.560,0.343)<br>*-81.200(-147.050,-15.350)*<br>-0.089(-0.284,0.105)<br>-<br>*80.400(-44.400,205.200)*	0.090<br>-<br>0.735<br>-<br>-	50.3%<br>-<br>0.0%<br>-<br>-	0.085<br>*0.000*<br>0.000<br>-<br>*0.000*
	**Study design**<br>Cross-sectional<br>Case-control<br>Cohort	A(a)	1<br>6<br>**6**<br>0<br>1<br>0	-0.074（-0.374, 0.226）<br>**-41.098（-71.084, -11.113）**<br>-<br>-0.067（-0.328, 0.194）	0.002<br>**0.443**<br>-<br>0.007	54.4%<br>**0.0%**<br>-<br>56.4%	0.080<br>**0.000**<br>-<br>0.058	19<br>**6**<br>0<br>13	-0.074（-0.374, 0.226）<br>**-41.098（-0.374，-11.113）**<br>-<br>-0.067（-0.328, 0.194）	0.002<br>**0.443**<br>-<br>0.007	54.4%<br>**0.0%**<br>-<br>56.4%	0.081<br>**0.000**<br>-<br>0.058
		A(b)	8<br>0<br>0<br>8	-0.002（-0.026, 0.022）<br>-<br>-<br>-0.002（-0.026, 0.022）	0.350	10.3%	0.0002	6<br>0<br>0<br>6	-9.725（-33.730, 14.280）<br>-<br>-<br>-9.725（-33.730, 14.280）	0.157<br>-<br>-<br>0.157	37.4%<br>-<br>-<br>37.4%	305.450<br>-<br>-<br>305.450

		B	1<br>1<br>0<br>*1*<br>1<br>0	-0.024（-0.173, 0.125）<br>-<br>***-107.930（-206.175，-9.685）***<br>-0.016（-0.119, 0.088）	0.288<br>-<br>**-**<br>0.603	16.4%<br>-<br>**-**<br>0.0%	0.009<br>0.000<br>**-**<br>0.000	10<br>-<br>-<br>10	-0.107（-0.391, 0.177）<br>-<br>-<br>-0.107（-0.391, 0.177）	0.006<br>-<br>-<br>0.006	60.9%<br>-<br>-<br>60.9%	0.0675<br>-<br>-<br>0.0675
	**Setting**<br>Europe<br>Asia<br>North America<br>Other	A(a)	1<br>6<br>6<br>6<br>3<br>*1*	-0.093(-0.197,0.012)<br>-3.150(-20.708,14.407)<br>0.876(-13.560,15.313)<br>-0.093(-0.197,0.011)<br>*-103.000(-221.000,15.000)*	0.383<br>0.786<br>0.414<br>0.755<br>-	0.0%<br>0.0%<br>0.2%<br>0.0%<br>-	0.000<br>0.000<br>2.129<br>0.000<br>*0.000*	19<br>**3**<br>13<br>2<br>*1*	-0.074(-0.374,0.226)<br>**-30.266(-55.690, -4.842)**<br>-0.081(-0.428,0.267)<br>-23.936(-79.540,31.669)<br>*4.000(-161.500,169.500)*	0.002<br>**0.514**<br>0.008<br>0.015<br>-	54.4%<br>**83.1%**<br>0.0%<br>55.2%<br>-	0.081<br>**0.000**<br>0.082<br>1376.477<br>*0.000*
		A(b)	8<br>4<br>0<br>4<br>0	-0.002(-0.026,0.022)<br>-1.822(-8.041,4.397)<br>-<br>0.001(-0.010,0.012)<br>-	0.350<br>0.223<br>-<br>0.714<br>-	10.3%<br>31.5%<br>-<br>0.0%<br>-	0.000<br>14.360<br>-<br>0.000<br>-	6<br>*1*<br>0<br>**5**<br>0	-9.725(-33.730,14.280)<br>*-20.748(-60.844,19.348)*<br>-<br>**-0.150(-0.275,-0.025)**<br>-	0..314<br>*0.161*<br>-<br>**0.157**<br>-	37.4%<br>*39.0%*<br>-<br>**-**<br>-	305.450<br>*0.000*<br>-<br>**265.296**<br>-
		B	1<br>1<br>5<br>5<br>*1*<br>0	-0.024(-0.173,0.125)<br>-31.856(-68.452,4.741)<br>-25.100(-149.100,98.900)<br>*-0.016(-0.119,0.088)*<br>-	0.288<br>0.315<br>-<br>*0.443*<br>-	16.4%<br>15.6%<br>-<br>*0.0%*<br>-	0.009<br>275.344<br>0.000<br>*0.000*<br>-	10<br>4<br>*1*<br>5<br>0	-0.107（-0.391, 0.177）<br>-46.251（-95.765, 3.264）<br>*80.400（-44.400, 205.200）*	0.006<br>0.133<br>-<br>0.031<br>-	60.9%<br>46.4%<br>-<br>62.5%<br>-	0.067<br>1148.479<br>*0.000*<br>0.032<br>-

									-0.104（-0.319, 0.110）<br>-			
	**Matrix**<br>Serum<br>Plasma<br>Whole blood	A(a)	1<br>5<br>1<br>0<br>4<br>*1*	-0.093(-0.197,0.011)<br>-0.093(-0.197,0.012)<br>-1.826(-14.925,11.272)<br>*-103.000(-221.000,15.000)*	0.748<br>0.699<br>0.848<br>-	0.0%<br>0.0%<br>0.0%<br>-	0.000<br>0.000<br>0.000<br>*0.000*	19<br>14<br>4<br>*1*	-0.074(.0374,0.226)<br>-0.066(-0.340,0.209)<br>-12.967(-32.819,6.884)<br>*4.000(-161.500,169.500)*	0.002<br>0.004<br>0.173<br>-	54.4%<br>57.4%<br>39.8%<br>-	0.081<br>0.066<br>154.15<br>*0.000*
		A(b)	8<br>3<br>4<br>*1*	-0.002(-0.026,0.022)<br>0.001(-0.010,0.012)<br>-8.144(-34.035,17.747)<br>*-0.068(-0.162,0.026)*	0.350<br>0.714<br>0.238<br>-	10.3%<br>0.0%<br>30.3%<br>-	0.000<br>0.000<br>223.038<br>*0.000*	6<br>4<br>2<br>-	-9.725(-33.730,14.280)<br>-4.529(-47.672,38.613)<br>-18.992(-65.858,27.873)<br>-	0.157<br>0.294<br>0.039<br>-	37.4%<br>1902%<br>76.6%<br>-	305.450<br>407.567<br>926.797<br>-
		B	1<br>1<br>9<br>2<br>0	-0.024(-0.173,0.125)<br>-0.046(-0.322,0.231)<br>0.000(-0.135,0.135)<br>-	0.771<br>0.162<br>0.805<br>-	16.4%<br>32.0%<br>0.0%<br>-	0.009<br>0.389<br>0.000<br>-	10<br>8<br>2<br>0	-0.107（-0.391, 0.177）<br>-0.090（-0.484, 0.304）<br>-33.711（-111.958, 44.535）<br>-	0.006<br>0.019<br>0.016<br>-	60.9%<br>58.1%<br>82.8%<br>-	0.067<br>0.100<br>2720.150<br>-
	**Adjusted for: GA**<br>Yes<br>No	A(a)	1<br>5<br>1<br>0<br>5	-0.093(-0.197,0.011)<br>-3.377(-15.461,8.706)<br>-0.093(-0.197,0.012)	0.748<br>0.497<br>0.843	0.0%<br>0.0%<br>0.0%	0.000<br>0.000<br>0.000	19<br>**12**<br>7	-0.074(-0.374,0.226)<br>**-30.480(-49.458, -11.502)**<br>-0.051(-0.174,0.072)	0.002<br>**0.099**<br>0.300	54.4%<br>**36.4%**<br>17.0%	0.081<br>**315.879**<br>0.004

		A(b)	8<br>3<br>5	-0.002(-0.026,0.022)<br>-4.737(-32.332,22.858)<br>-0.013(-0.074,0.047)	0.350<br>0.328<br>0.206	10.3%<br>36.7%<br>13.5%	0.0001<br>287.165<br>0.001	6<br>3<br>**3**	-9.725(-33.730,14.280)<br>-0.150(-0.275,0.025)<br>**-53.529(-94.510, -12.547)**	0.157<br>0.680<br>**0.704**	37.4%<br>0.0%<br>**0.0%**	305.450<br>0.000<br>**0.000**
		B	1<br>1<br>4<br>7	-0.024(-0.173,0.125)<br>-3.728(-17.427,9.971)<br>-0.043(-0.288,0.202)	0.288<br>0.347<br>0.202	16.4%<br>9.3%<br>29.7%	0.009<br>55.597<br>0.261	10<br>4<br>6	-0.107（-0.391, 0.177）<br>-25.472（-66.406, 15.461）<br>-0.086（-0.431, 0.259）	0.006<br>0.015<br>0.035	60.9%<br>715.5%<br>58.3%	0.067<br>1163.103<br>0.072
		A(a)	1<br>5<br>1<br>3<br>2	-0.093(-0.197,0.012)<br>-0.093(-0.197,0.012)<br>-71.547(-171.788,28.694)	0.748<br>0.835<br>0.748	0.0%<br>0.0%<br>0.0%	0.000<br>0.000<br>0.000	19<br>13<br>6	-0.074(-0.374,0.226)<br>-0.139(-0.696,0.691)<br>-0.085(-0.374,0.166)	0.002<br>0.002<br>0.163	54.4%<br>62.0%<br>36.5%	0.084<br>0.343<br>0.025
	**Adjusted for :**<br>**Parity**<br>Yes<br>No	A(b)	8<br>5<br>3	-0.002(-0.026,0.022)<br>0.001(-0.010,0.012)<br>-4.775(-32.331,22.781)	0.350<br>0.623<br>0.207	10.3%<br>0.0%<br>36.6%	0.0002<br>0.000<br>286.095	6<br>4<br>2	-9.725(-33.730,14.280)<br>-28.179(-70.634,14.277)<br>14.550(-23.832,52.933)	0.157<br>0.065<br>0.649	37.4%<br>58.4%<br>0.0%	305.450<br>922.488<br>0.000
		B	1<br>1<br>9<br>**2**	-0.024(-0.173,0.125)<br>-0.016(-0.119,0.088)<br>**-65.098(-116.563, -13.632)**	0.288<br>0.782<br>**0.316**	16.4%<br>0.0%<br>**0.7%**	0.009<br>0.000<br>**11.496**	10<br>9<br>*1*	-0.107（-0.391, 0.177）<br>-0.107（-0.399, 0.185）<br>*3.000（-54.500, 60.500）*	0.006<br>0.003<br>-	60.9%<br>65.2%<br>-	0.067<br>0.072<br>*0.000*

**GA**<br>**(weeks)**	**Gender**<br>Male<br>Female	A(a)	0	-	-	-	-	2<br>*1*<br>*1*	-0.274(-0.629,0.081)<br>*-0.170(-0.670,0.330)*<br>*-0.380(-0.880,0.130)*	0.562<br>-<br>-	0.0%<br>-<br>-	0.000<br>-<br>-
		A(b)	*1*<br>*1*<br>0	*-0.011(-0.032,0.011)*<br>*-0.011(-0.032,0.011)*<br>-	-	-	-	*1*<br>*1*<br>-	*-1.975(-5.063,1.112)*<br>*-1.975(-5.050,1.125)*<br>-	-	-	-
		B	*1*<br>*1*<br>0	*-0.390(-0.930,0.150)*<br>*-0.390(-0.930,0.150)*<br>-	-	-	-	*1*<br>*1*<br>-	*-0.300(-0.900,0.300)*<br>*-0.300(-0.900,0.300)*<br>-	-	-	-
	**Timing Category**<br>First trimester<br>Second trimester<br>Third trimester<br>Cord blood	A(a)	1<br>0<br>4<br>0<br>**2**<br>4	-0.028(-0.097,0.041)<br>0.070(-0.106,0.246)<br>-<br>**-0.089(-0.168, -0.010)**<br>-0.009(-0.121,0.103)	0.013<br>0.288<br>-<br>**0.154**<br>0.048	57.0%<br>20.2%<br>-<br>**50.8%**<br>62.0%	0.007<br>0.001<br>-<br>**0.001**<br>0.008	12<br>2<br>2<br>2<br>**6**	-17.763(-37.708,1.781)<br>-17.594(-53.355,18.167)<br>-0.027(-0.258,0.204)<br>-24.287(-119.085,7.51<br>**-44.740(-81.749, -7.730)**	0.228<br>0.569<br>0.747<br>0.683<br>**0.354**	21.9%<br>0.0%<br>0.0%<br>0.0%<br>**9.7%**	218.295<br>0.000<br>0.000<br>0.000<br>**219.448**
		A(b)	6<br>2<br>*1*<br>*1*<br>2	-0.011(-0.091,0.068)<br>-0.099(-0.234,0.035)<br>*-0.040(-0.240,0.160)*<br>*-0.080(-0.320,0.160)*<br>0.076(-0.028,0.179)	0.335<br>0.663<br>-<br>-<br>0.358	12.5%<br>0.0%<br>-<br>-<br>0.0%	0.001<br>0.000<br>*0.000*<br>*0.000*<br>0.000	0	-	-	-	-
		B	5<br>*1*<br>3<br>0<br>*1*	-0.142(-0.343,0.059)<br>*8.600(-59.700,76.900)*<br>-0.144(-0.399,0.112)<br>-	0.594<br>-<br>0.277<br>-<br>-	0.0%<br>-<br>22.1%<br>-<br>-	0.000<br>*0.000*<br>0.013<br>-<br>*0.000*	5<br>***1***<br>3<br>0<br>*1*	-0.108(-0.560,0.343)<br>***-81.200(-147.050, -15.350)***<br>-0.089(-0.284,0.105)<br>-	0.090<br>**-**<br>0.735<br>-<br>-	50.3%<br>**-**<br>0.0%<br>-<br>-	0.085<br>***0.000***<br>0.000<br>-<br>*0.000*

				-25.100(-149.100,98.900)					*80.400(-44.400,205.200)*			
	**Study design**<br>Cross-sectional<br>Case-control<br>Cohort	A(a)	5<br>*1*<br>0<br>4	-0.021（-0.108, 0.066）<br>*0.103（-0.016, 0.223）*<br>-<br>-0.042（-0.113, 0.029）	0.123<br>-<br>-<br>0.363	44.9%<br>-<br>-<br>5.9%	0.004<br>*0.000*<br>-<br>0.0003	9<br>4<br>0<br>5	-0.045（-0.172, 0.082）<br>-<br>-0.025（-0.190, 0.139）<br>-0.041（-0.209, 0.127）	0.005<br>0.414<br>-<br>0.001	63.3%<br>0.0%<br>-<br>78.8%	0.018<br>0.000<br>-<br>0.025
		A(b)	3<br>0<br>0<br>3	0.094（-0.175, 0.363）<br>-<br>-<br>0.094（-0.175, 0.363）	0.123<br>-<br>-<br>0.123	52.2%<br>-<br>-<br>52.2%	0.032<br>-<br>-<br>0.032	6<br>0<br>0<br>6	-9.725(-33.730,14.280)<br>-<br>-<br>-9.725(-33.730,14.280)	0.157<br>-<br>-<br>0.157	37.4%<br>-<br>-<br>37.4%	305.450<br>-<br>-<br>305.450
		B	3<br>0<br>***1***<br>2	-0.405（-0.866, 0.128）<br>-<br>***-0.820（-1.295，-0.345）***<br>-0.163（-0.453, 0.128）	0.043<br>-<br>-<br>0.328	68.3%<br>-<br>-<br>0.0%	0.112<br>-<br>***0.000***<br>0.000	10<br>0<br>0<br>10	-0.107(-0.391,0.177)<br>-<br>-<br>-0.107(-0.391,0.177)	0.006<br>-<br>-<br>0.006	60.9%<br>-<br>-<br>60.9%	0.0.68<br>-<br>-<br>0.0.68

	**Setting**<br>Europe<br>Asia<br>America<br>Other	A(a)	5<br>3<br>2<br>0<br>0	-0.021（-0.108, 0.125）<br>-0.104（-0.223, 0.015）<br>0.024（-0.077, 0.125）<br>-<br>-	0.123<br>0.658<br>0.176<br>-<br>-	44.9%<br>0.0%<br>42.5%<br>-<br>-	0.004<br>0.000<br>0.003<br>-<br>-	9<br>2<br>6<br>0<br>*1*	-0.045（-0.172, 0.082）<br>0.002（-0.157, 0.162）<br>-0.080（-0.253, 0.093）<br>-<br>*0.200（-0.685, 1.085）*	0.005<br>0.949<br>0.001<br>-<br>-	63.3%<br>0.0%<br>76.7%<br>-<br>-	0.018<br>0.000<br>0.029<br>0.000<br>-
		A(b)	3<br>3<br>0<br>0<br>0	0.094（-0.175, 0.363）<br>0.094（-0.175, 0.363）<br>-<br>-<br>-	0.123<br>0.123<br>-<br>-<br>-	52.2%<br>52.2%<br>-<br>-<br>-	0.032<br>0.032<br>-<br>-<br>-	2<br>2<br>0<br>0<br>0	-0.176（-0.820, 0.468）<br>-0.176（-0.820, 0.468）<br>-<br>-<br>-	0.238<br>0.238<br>-<br>-<br>-	28.3%<br>28.3%<br>-<br>-<br>-	0.088<br>0.088<br>-<br>-<br>-
		B	3<br>***1***<br>2<br>0<br>0	-0.405（-0.866, 0.055）<br>***-0.625（-1.045，-0.205）***<br>-0.070（-0.415, 0.275）<br>-<br>-	0.043<br>***0.241***<br>-<br>-<br>-	68.3%<br>***27.2%***<br>-<br>-<br>-	0.112<br>***0.025***<br>0.000<br>-<br>-	10<br>4<br>*1*<br>5<br>0	-0.107(-0.391,0.177)<br>-46.251(-95.765,3.264)<br>*80.400(-44.400,205.200)*<br>-0.104(-0.319,0.110)<br>-	0.006<br>0.133<br>-<br>0.031<br>-	60.9%<br>46.4%<br>-<br>62.5%<br>-	0.067<br>1148.479<br>*0.000*<br>0.032<br>-

	**Matrix**<br>Serum<br>Plasma<br>Whole blood	A(a)	5<br>2<br>3<br>0	-0.021 (-0.108, 0.066)<br>-0.006 (-0.239, 0.228)<br>-0.023 (-0.094, 0.049)<br>-	0.123<br>0.032<br>0.383<br>-	44.9%<br>78.2%<br>0.0%<br>-	0.004<br>0.022<br>0.000<br>-	9<br>4<br>4<br>*1*	-0.045 (-0.172, 0.082)<br>-0.026 (-0.179, 0.127)<br>-0.050 (-0.238, 0.138)<br>*0.200 (-0.685, 1.085)*	0.005<br>0.449<br>0.000<br>-	63.3%<br>0.0%<br>84.1%<br>-	0.018<br>0.000<br>0.029<br>*0.000*
		A(b)	3<br>2<br>0<br>*1*	0.094 (-0.175, 0.363)<br>0.161 (-0.291, 0.613)<br>-<br>*0.027 (-0.464, 0.518)*	0.123<br>0.042<br>-<br>-	52.2%<br>75.9%<br>-<br>-	*0.031*<br>*0.085*<br>-<br>*0.000*	2<br>2<br>0<br>0	-0.176 (-0.820, 0.468)<br>-0.176 (-0.820, 0.468)<br>-<br>-	0.238<br>0.238<br>-<br>-	28.3%<br>28.3%<br>-<br>-	0.088<br>0.088<br>-<br>-
		B	3<br>3<br>0<br>0	-0.405 (-0.866, 0.055)	0.043	68.3%	0.112	10<br>8<br>2<br>-	-0.107(-0.391,0.177)<br>-0.090(-0.484,0.304)<br>-33.711(-111.958,44.535)<br>-	0.006<br>0.019<br>0.016<br>-	60.9%<br>58.1%<br>82.8%<br>-	0.067<br>0.100<br>2720.151<br>-
	**Adjusted for: GA**<br>Yes<br>No	A(a)	5<br>3<br>2	-0.021 (-0.108, 0.066)<br>0.024 (-0.077, 0.125)	0.123<br>0.176<br>0.658	44.9%<br>42.5%<br>0.0%	0.004<br>0.003<br>0.000	9<br>7<br>2	-0.045 (-0.172, 0.082)<br>-0.070 (-0.236, 0.097)	0.005<br>0.001<br>0.949	63.3%<br>72.4%<br>0.0%	0.018<br>0.027<br>0.000

				-0.104（-0.223,<br>0.015）					0.002（-0.157,<br>0.162）			
		A(b)	3<br>*1*<br>2	0.094（-0.175,<br>0.363）<br>*0.455（0.000,<br>0.909）*<br>-0.020（-0.060,<br>0.020）	0.123<br>-<br>0.852	52.2%<br>-<br>0.0%	0.032<br>*0.000*<br>0.000	6<br>**3**<br>**3**	-9.725(-33.730,14.280)<br>**-0.150(-0.275, -0.025)**<br>**-53.529(-94.510, -<br>12.547)**	0.157<br>**0.680**<br>**0.704**	37.45<br>**0.05**<br>**0.05**	305.450<br>**0.000**<br>**0.000**
		B	3<br>*1*<br>2	-0.405（-0.866,<br>0.055）<br>*-0.820（-1.295, -<br>0.345）*<br>-0.163（-0.453,<br>0.128）	68.3%<br>-<br>0.0%	68.3%<br>-<br>0.0%	0.112<br>*0.000*<br>0.000	10<br>4<br>6	-0.107(-0.391,0.177)<br>-25.472(-66.406,15.461)<br>-0.086(-0.431,0.259)	0.006<br>0.015<br>0.035	60.9%<br>71.5%<br>58.3%	0.067<br>1163.103<br>0.072
	**Adjusted for:**<br>**Parity**<br>Yes<br>No	A(a)	5<br>5<br>0	-0.021（-0.108,<br>0.066）<br>-0.021（-0.108,<br>0.066）<br>-	0.123<br>-<br>-	44.9%<br>44.9%<br>-	0.0042<br>0.0042<br>-	9<br>8<br>*1*	-0.045（-0.172,<br>0.082）<br>-0.051（-0.182,<br>0.080）<br>*0.200（-0.685,<br>1.085）*	0.005<br>0.003<br>-	63.3%<br>67.5%<br>-	0.018<br>0.019<br>*0.000*
		A(b)	3<br>*1*	0.094（-0.175,<br>0.363）	0.123<br>-	52.2%<br>-	0.031<br>*0.000*	6<br>4	-9.725(-33.730,14.280)<br>-28.179(-70.634,14.277)	0.157<br>0.065	37.4%<br>58.4%	305.450<br>922.487

			2	*-0.020（-0.060, 0.020）*<br>0.251（-0.167, 0.670）	0.210	36.2%	0.000	2	14.550(-23.832,52.933)	0.649	0.0%	0.000
		B	3<br>2<br>*1*	-0.405（-0.866, 0.055）<br>-0.163（-0.453, 0.128）<br>*-0.820（-1.295, -0.345）*	0.043<br>0.328<br>-	68.3%<br>0.0%<br>-	0.112<br>0.000<br>*0.000*	10<br>9<br>*1*	-0.107(-0.391,0.177)<br>-0.107(-0.399,0.185)<br>*3.000(-54.500,60.500)*	0.006<br>0.003<br>-	60.9%<br>65.2%<br>-	0.067<br>0.072<br>*0.000*
**BL/cm**	**Gender**<br>Male<br>Female	A(a)	1<br>2<br>6<br>6	-0.101（-0.385, 0.182）<br>-0.149（-0.609, 0.311）<br>-0.071（-0.431, 0.288）	0.927<br>0.631<br>0.906	0.0%<br>0.0%<br>0.0%	0.000<br>0.000<br>0.000	14<br>7<br>7	-0.069（-0.170, 0.032）<br>0.013（-0.077, 0.104）<br>-0.175（-0.356, 0.007）	0.087<br>0.375<br>0.197	36.1%<br>6.9%<br>30.2%	0.008<br>0.001<br>0.014
		A(b)	6<br>3<br>3	-0.060（-0.112, -0.009）<br>18.886（-47.912, 85.684）<br>-0.055（-0.120, 0.011）	0.370<br>0.125<br>0.821	6.4%<br>51.9%<br>0.0%	0.000<br>1777.163<br>0.0005	**5**<br>3<br>2	**-0.060（-0.112，-0.009）**<br>18.886（-47.912, 85.684）<br>-0.055（-0.120, 0.011）	**0.370**<br>0.125<br>0.821	**6.4%**<br>0.0%<br>51.9%	**0.000**<br>0.000<br>1777.164

		B	5<br>3<br>2	-20.383（-65.440, 24.674）<br>-18.136（-78.479, 42.207）<br>-23.213（-90.949, 44.522）	0.964<br>0.774<br>0.799	0.0%<br>0.0%<br>0.0%	0.000<br>0.000<br>0.0000	5<br>3<br>2	-33.211（-84.828, 18.406）<br>-21.267（-115.920, 73.385）<br>-51.621（-114.959, 11.717）	0.263<br>0.113<br>0.632	23.8%<br>54.1%<br>0.0%	825.905<br>3776.845<br>0.000
	**Study design**<br>Cross-sectional<br>Case-control<br>Cohort	A(a)	5<br>*1*<br>0<br>4	0.004（-0.098, 0.107）<br>*-0.310（-0.740, 0.120）*<br>-<br>0.020（-0.078, 0.117）	0.076<br>-<br>-<br>0.090	52.7%<br>-<br>-<br>53.8%	0.005<br>*0.000*<br>-<br>0.005	8<br>5<br>3<br>0	-0.033（-0.098, 0.031）<br>-0.017（-0.085, 0.051）<br>-0.181（-0.382, 0.019）<br>-	0.297<br>0.260<br>0.698<br>-	16.9%<br>24.2%<br>0.0%<br>-	0.001<br>0.001<br>0.000<br>-
		A(b)	2<br>-<br>-<br>2	-0.001（-0.026, 0.024）	0.997	0.0%	0.000	2<br>-<br>-<br>2	-0.159（-0.622, 0.304）<br>-<br>-<br>-0.159（-0.622, 0.304）	0.253<br>-<br>-<br>0.253	23.5%<br>-<br>-<br>23.5%	0.030<br>-<br>-<br>0.030
		B	*1*<br>*0*<br>*0*<br>*1*	*-0.050(-0.375,0.275)*<br>-<br>-<br>*-0.050(-0.370,0.280)*	-	-	-	*1*<br>0<br>0<br>*1*	*-0.240(-0.605,0.125)*<br>-<br>-<br>*-0.240(-0.600,0.130)*	-	-	-

	**Setting**<br>Europe<br>Asia<br>North America<br>Other	A(a)	5<br>2<br>2<br>0<br>*1*	0.004（-0.098, 0.107）<br>0.064（-0.096, 0.224）<br>-0.042（-0.116, 0.032）<br>-<br>*-0.310（-0.740, 0.120）*	0.076<br>0.058<br>0.860<br>-<br>-	52.7%<br>72.1%<br>0.0%<br>-<br>-	0.006<br>0.009<br>0.000<br>-<br>*0.000*	8<br>2<br>5<br>0<br>*1*	-0.033（-0.098, 0.031）<br>-0.030（-0.142, 0.081）<br>-0.050（-0.157, 0.056）<br>-<br>*-0.140（-0.800, 0.520）*	0.297<br>0.361<br>0.113<br>-<br>-	16.9%<br>0.0%<br>46.5%<br>-<br>-	0.001<br>0.000<br>0.005<br>-<br>*0.000*
		A(b)	2<br>2<br>-<br>-<br>-	-0.001（-0.026, 0.024）<br>-0.001（-0.026, 0.024）<br>-<br>-<br>-	0.997<br>0.997<br>-<br>-<br>-	0.0%<br>0.0%<br>-<br>-<br>-	0.000<br>0.000<br>-<br>-<br>-	2<br>2<br>0<br>0<br>0	-0.159（-0.622, 0.304）<br>-0.159（-0.622, 0.304）<br>-<br>-<br>-	0.253<br>0.253<br>-<br>-<br>-	23.5%<br>23.5%<br>-<br>-<br>-	0.0305<br>0.0305<br>-<br>-<br>-
		B	*1*<br>*1*<br>*0*<br>*0*<br>*0*	*-0.050(-0.375,0.275)*<br>*-0.050(-0.370,0.280)-*<br>-<br>-	-	-	-	*1*<br>*1*<br>0<br>0<br>0	*-0.240(-0.605,0.125)*<br>*-0.240(-0.600,0.130)*<br>-<br>-<br>-	-	-	-
	**Matrix**<br>Serum<br>Plasma	A(a)	5<br>***1***<br>3<br>*1*	0.004（-0.098, 0.107）<br>***0.150（0.020, 0.279）***	0.076<br>-<br>0.899<br>-	52.75<br>-<br>0.05<br>-	0.006<br>***0.000***<br>0.000<br>*0.000*	8<br>3<br>4<br>*1*	-0.033（-0.098, 0.031）<br>-0.089（-0.294, 0.117）	0.297<br>0.213<br>0.193<br>-	16.9%<br>35.4%<br>36.6%<br>-	0.001<br>0.011<br>0.002<br>*0.000*

	Whole blood			-0.033 (-0.094, 0.028)<br>*-0.310 (-0.740, 0.120)*					-0.024 (-0.101, 0.052)<br>*-0.140 (-0.800, 0.520)*			
		A(b)	2<br>2<br>0<br>0	-0.001 (-0.026, 0.024)	0.997	0.0%	0.000	2<br>2<br>0<br>0	-0.159 (-0.622, 0.304)<br>-0.159 (-0.622, 0.304)<br>-<br>-	0.253<br>0.253<br>-<br>-	23.5%<br>23.5%<br>-<br>-	0.0305<br>0.0305<br>-<br>-
		B	*1*<br>*1*<br>*0*<br>*0*	*-0.050(-0.375,0.275)*<br>*-0.050(-0.370,0.280)*<br>-<br>-	-	-	-	*1*<br>*1*<br>0<br>0	*-0.240(-0.605,0.125)*<br>*-0.240(-0.600,0.130)*<br>-<br>-	-	-	-
	**Adjusted for: GA**<br>Yes<br>No	A(a)	5<br>3<br>2	0.004 (-0.098, 0.107)<br>-0.050 (-0.123, 0.023)<br>0.064 (-0.096, 0.224)	0.076<br>0.477<br>0.058	52.7%<br>0.0%<br>72.1%	0.005<br>0.000<br>0.009	8<br>4<br>4	-0.033 (-0.098, 0.031)<br>-00.017 (-0.112, 0.079)<br>-0.066 (-0.170, 0.039)	0.297<br>0.211<br>0.366	16.9%<br>33.5%<br>5.3%	0.001<br>0.003<br>0.001
		A(b)	2<br>*1*<br>*1*	-0.001(-0.026,0.024)<br>*-0.001(-0.040,0.010)*<br>*0.000(-0.455,0.455)*	0.997<br>-<br>-	0.0%<br>-<br>-	0.000<br>-<br>-	2<br>*1*<br>*1*	-0.159(-0.622,0.304)<br>*0.000(-0.294,0.588)*<br>*-0.510(-1.260,0.250)*	0.253<br>-<br>-	23.5%<br>-<br>-	0.030<br>-<br>-

		B	1<br>-<br>1	*-0.050(-0.375,0.275)*<br>-<br>*-0.050(-0.370,0.280)*	-	-	-	*1*<br>0<br>*1*	*-0.240(-0.605,0.125)*<br>-<br>*-0.240(-0.600,0.130)*	-	-	-
	**Adjusted for : Parity**	A(a)	5<br>4<br>*1*	0.004（-0.098, 0.107）<br>0.020（-0.078, 0.117）<br>*-0.310（-0.740, 0.120）*	0.076<br>0.090<br>-	52.7%<br>53.8%<br>-	0.006<br>0.005<br>*0.000*	8<br>5<br>3	-0.033（-0.098, 0.031）<br>-0.017（-0.085, 0.051）<br>-0.181（-0.382, 0.019）	0.297<br>0.260<br>0.698	16.9%<br>24.2%<br>0.0%	0.001<br>0.001<br>0.000
	Yes<br>No	A(b)	2<br>*1*<br>*1*	-0.001(-0.026,0.024)<br>*0.000(-0.455,0.455)*<br>*-0.001(-0.040,0.010)*	0.997<br>-<br>-	0.0%<br>-<br>-	0.000<br>-<br>-	2<br>*1*<br>*1*	-0.159(-0.622,0.304)<br>*-0.510(-1.260,0.250)*<br>*0.000(-0.294,0.588)*	0.253<br>-<br>-	23.5%<br>-<br>-	0.030<br>-<br>-
		B	1<br>1<br>0	*-0.050(-0.375,0.275)*<br>*-0.050(-0.370,0.280)*<br>-	-	-	-	*1*<br>*1*<br>0	*-0.240(-0.605,0.125)*<br>*-0.240(-0.600,0.130)*<br>-	-	-	-
**HC/cm**	**Gender**<br>Male<br>Female	A(a)	1<br>2<br>6<br>6	-0.101(-0.385,0.182)<br>-0.071(-0.431,0.288)<br>-0.149(-0.609,0.311)	0.927<br>0.631<br>0.906	0.0%<br>0.0%<br>0.0%	0.000<br>0.000<br>0.000	14<br>7<br>7	-0.069(-0.0170,0.032)<br>0.013(-0.077,0.104)<br>-0.175(-0.356,0.007)	0.087<br>0.375<br>0.197	36.1%<br>6.9%<br>30.2%	0.008<br>0.001<br>0.014
		A(b)	6<br>3<br>3	-0.006(-0.051,0.040)<br>-0.006(-0.051,0.040)<br>-21.339(-74.019,31.340)	0.264<br>0.403<br>0.098	22.6%<br>0.0%<br>56.9%	0.001<br>0.000<br>1290.327	**5**<br>3<br>2	**-0.060(-0.112,-0.009)**<br>18.886(-47.912,85.684)<br>-0.055(-0.120,0.011)	**0.370**<br>0.125<br>0.821	**6.4%**<br>51.9%<br>0.0%	**0.000**<br>1777.163<br>0.000
		B	5<br>3<br>2	-20.383(-65.440,24.674)<br>-18.136(-78.479,42.207)<br>-23.213(-90.949,44.522)	0.964<br>0.774<br>0.799	0.0%<br>0.0%<br>0.0%	0.000<br>0.000<br>0.000	5<br>3<br>2	-33.211(-84.828,18.406)<br>-21.267(-115.920,73.385)	0.263<br>0.113<br>0.632	23.8%<br>54.1%<br>0.0%	825.906<br>3776.845<br>0.000

									-51.621(-114.959,11.717)			
	**Timing Category**	A(a)	4	0.002(-0.063,0.066)	0.388	0.8%	0.000	4	-0.044(-0.150,0.061)	0.073	57.05	0.007
	First trimester		2	-0.028(-0.114,0.059)	0.776	0.0%	0.000	2	-0.100(-0.290,0.091)	0.060	71.85	0.014
	Second trimester		0	-	-	-	-	0	-	-	-	0.000
	Third trimester		*1*	*-0.020(-0.145,0.105)*	-	-	*0.000*	*1*	*0.030(-0.075,0.135)*	-	-	-
	Cord blood		*1*	*0.120(-0.030,0.270)*	-	-	*0.000*	*1*	*-0.020(-0.195,0.155)*	-	-	*0.000*
		B	*1*	*-0.900(-2.300,0.500)*				***1***	***-0.291(-0.544, -0.039)***	**-**	**-**	**-**
			1	*-0.900(-2.300,0.500)*				***1***	***-0.291(-0.544, -0.039)***	**-**	**-**	**-**
			0	-	-	-	-	0	-	-	-	-
			0	-				0	-	-	-	-
			0	-				0	-	-	-	-
	**Study design**	A(a)	5	-0.007(-0.084,0.070)	0.244	26.7%	0.002	6	-0.019(-0.093,0.054)	0.126	41.9%	0.003
	Cross-sectional		2	-0.060(-0.483,0.364)	0.043	75.6%	0.073	2	-0.011(-0.144,0.122)	0.873	0.0%	0.000
	Case-control		0	-	-	-	-	0	-	-	-	-
	Cohort		3	-0.025(-0.096,0.046)	0.956	0.0%	0.000	4	-0.025(-0.127,0.077)	0.035	65.0%	0.007
		A(b)	*1*	*0.009(-0.016,0.034)*				*1*	*-0.175(-0.412,0.06)*	-	-	-
			0	-	-	-	-	0	-	-	-	-
			0	-				0	-	-	-	-
			1	*0.009(-0.018,0.032)*				*1*	-0.175(-0.425,0.050)	-	-	-
		B	4	-0.132(-0.334,0.071)	0.547	0.0%	0.000	3	-0.278(-0.644,0.088)	0.081	60.2%	0.055
			0	-	-	-	-	0	-	-	-	-
			1	*-0.240(-0.620,0.140)*	-	-	*0.000*	0	-	-	-	-
			3	-0.089(-0.328,0.151)	0.430	0.0%	0.000	3	-0.278(-0.644,0.088)	0.081	60.2%	0.055

	**Setting**<br>Europe<br>Asia<br>America<br>Other	A(a)	5<br>3<br>2<br>0<br>0	-0.007(-0.084,0.070)<br>-0.041(-0.128,00.45)<br>0.044(-0.093,0.180)<br>-<br>-	0.244<br>0.358<br>0.160<br>-<br>-	26.7%<br>2.6%<br>49.4%<br>-<br>-	0.002<br>0.000<br>0.005<br>-<br>-	6<br>3<br>3<br>0<br>0	-0.019(-0.093,0.054)<br>-0.074(-0.212,0.064)<br>0.027(-0.040,0.094)<br>-<br>-	0.126<br>0.110<br>0.842<br>-<br>-	41.9%<br>54.7%<br>0.0%<br>-<br>-	0.003<br>0.008<br>0.000<br>-<br>-
		A(b)	*1*<br>*0*<br>*0*<br>*1*<br>-	*0.009(-0.016,0.034)*<br>-<br>-<br>*0.009(-0.018,0.032)*<br>-	-	-	-	*1*<br>*0*<br>*0*<br>*1*<br>-	*-0.175(-0.412,0.06)*<br>-<br>-<br>*-0.175(-0.425,0.050)*<br>-	-	-	-
		B	4<br>2<br>-<br>2<br>-	-0.132(-0.334,0.071)<br>-0.285(-0.652,0.081)<br>-<br>-0.285(-0.652,0.081)<br>-	0.547<br>0.373<br>-<br>0.549<br>-	0.0%<br>0.0%<br>-<br>0.0%<br>-	0.000<br>0.000<br>-<br>0.000<br>-	3<br>2<br>*1*<br>0<br>0	-0.278(-0.644,0.088)<br>-0.291(-0.544, -0.039)<br>*-0.639(-0.644,0.733)*<br>-<br>-	0.081<br>-<br>*0.036*<br>-	60.2%<br>-<br>*77.4%*<br>-	0.055<br>0.000<br>*0.791*<br>-
	**Matrix**<br>Serum<br>Plasma<br>Whole blood	A(a)	5<br>2<br>2<br>*1*	-0.007(-0.084,0.070)<br>-0.060(-0.483,0.364)<br>-0.028(-0.114,0.059)<br>*-0.020(-0.145,0.105)*	0.244<br>0.043<br>0.776<br>-	26.7%<br>75.6%<br>0.0%<br>-	0.002<br>0.073<br>0.000<br>0.000<br>-	6<br>2<br>3<br>*1*	-0.019(-0.093,0.054)<br>-0.011(-0.144,0.122)<br>-0.048(-0.192,0.096)<br>*0.030(-0.093,0.054)*	0.126<br>0.873<br>0.022<br>-	41.9%<br>0.0%<br>73.8%<br>-	0.003<br>0.000<br>0.011<br>*0.000*
		A(b)	*1*<br>*0*<br>*1*<br>*0*	*0.009(-0.016,0.034)*<br>-<br>*0.009(-0.018,0.032)*<br>-	-	-	-	*1*<br>*0*<br>*1*<br>*0*	*-0.175(-0.412,0.06)*<br>-<br>*-0.175(-0.425,0.050)*<br>-	-	-	-
		B	4<br>2<br>2	-0.132(-0.334,0.310)<br>-0.136(-0.450,0.178)<br>-0.193(-0.695,0.310)	0.547<br>0.339<br>0.271	0.0%<br>0.0%<br>17.3%	0.000<br>0.000<br>0.055	3<br>*1*<br>2	-0.278(-0.644,0.088)<br>*-0.070(-0.365,0.225)*<br>-0.721(-0.185,0.413)	0.081<br>-<br>0.074	60.2%<br>-<br>68.8%	0.055<br>*0.000*<br>0.502

			0	-	-	-	-	-	-	-	-	-
	**Adjusted for: GA**<br>Yes<br>No	A(a)	5<br>2<br>3	-0.007(-0.084,0.070)<br>-0.108(-0.084,0.160)<br>0.013(-0.077,0.103)	0.244<br>0.160<br>0.238	26.7%<br>49.4%<br>30.3%	0.002<br>0.022<br>0.002	6<br>3<br>3	-0.019(-0.093,0.054)<br>0.032(-0.037,0.100)<br>-0.078(-0.206,0.051)	0.126<br>0.947<br>0.119	41.9%<br>0.0%<br>52.9%	0.003<br>0.000<br>0.007
		A(b)	*1*<br>*1*<br>*0*	*0.009(-0.016,0.034)*<br>*0.009(-0.018,0.032)*<br>-	-	-	-	*1*<br>*1*<br>*0*	*-0.175(-0.412,0.06)*<br>*-0.175(-0.425,0.050)*<br>-	-	-	-
		B	4<br>*1*<br>3	-0.132(-0.334,0.071)<br>*-0.100(-0.370,0.170)*<br>-0.173(-0.480,0.135)	0.547<br>-<br>0.367	0.0%<br>-<br>0.1%	0.000<br>*0.000*<br>0.0001	3<br>***1***<br>2	-0.278(-0.644,0.088)<br>***-1.500(-0.644, -0.200)***<br>-0.194(-0.409,0.021)	0.081<br>**-**<br>0.264	60.2%<br>**-**<br>19.8%	0.055<br>***0.000***<br>0.005
	**Adjusted for: Parity**<br>Yes<br>No	A(a)	5<br>5<br>0	-0.007(-0.084,0.070)<br>-0.007(-0.084,0.070)<br>-	0.244<br>0.244<br>-	26.7%<br>26.7%<br>-	0.002<br>0.002<br>-	6<br>6<br>0	-0.019(-0.093,0.054)<br>-0.019(-0.093,0.054)<br>-	0.126<br>0.126<br>-	41.9%<br>41.9%<br>-	0.003<br>0.003<br>-
		A(b)	*1*<br>*1*<br>*0*	*0.009(-0.016,0.034)*<br>*0.009(-0.018,0.032)*<br>-	-	-	-	*1*<br>*1*<br>*0*	*-0.175(-0.412,0.06)*<br>*-0.175(-0.425,0.050)*<br>-	-	-	-
		B	4<br>3<br>*1*	-0.132(-0.334,0.071)<br>-0.089(-0.328,0.151))<br>*-0.240(-0.620,0.140)*	0.547<br>0.430<br>-	0.0%<br>0.0%<br>-	0.000<br>0.000<br>*0.000*	3<br>3<br>0	-0.278(-0.644,0.088)<br>-0.278(-0.644,0.088)<br>-	0.081.6<br>0.081.6<br>-	0.2%<br>0.2%<br>-	0.056<br>0.056<br>-
	**Timing Category**<br>First trimester	A(a)	3<br>0<br>0<br>*1*	-0.003(-0.011,0.005)<br>-<br>-<br>*-0.050(-0.130,0.030)*	0.331<br>-<br>-<br>-	9.5%<br>-<br>-<br>-	0.000<br>-<br>-<br>*0.000*	3<br>0<br>0<br>*1*	-0.003(-0.007,0.001)<br>-<br>-<br>*-0.030(-0.155,0.095)*	0.876<br>-<br>-<br>-	0.0%<br>-<br>-<br>-	0.000<br>-<br>-<br>*0.000*

	Second trimester<br>Third trimester<br>Cord blood		2	-0.004(-0.007, -0.000)	0.335	0.0%	0.000	2	-0.003(-0.007,0.001)	0.770	0.0%	0.000
		A(b)	*1*<br>*0*<br>*1*<br>*0*<br>*0*	*-0.100(-0.250,0.050)*<br>-<br>*-0.100(-0.300,0.000)*<br>-<br>-	-	-	-	*1*<br>0<br>*1*<br>0<br>0	*0.100(0.000,0.200)*<br>-<br>*0.100(0.000,0.200)*<br>-<br>-	-	-	-
		B	2<br>0<br>*1*<br>0<br>*1*	-0.045(-0.139,0.048)<br>-<br>*-0.110(-0.250,0.030)*<br>-<br>*-0.010(-0.100,0.080)*	-	-	-	2<br>0<br>*1*<br>0<br>*1*	0.013(-0.059,0.085)<br>-<br>*0.020(-0.110,0.160)*<br>-<br>*0.010(-0.080,0.090)*	0.902<br>-<br>-<br>-<br>-	0.0%<br>-<br>-<br>-<br>-	0.000<br>-<br>-<br>-<br>-
	**Study design**	A(a)	3<br>2<br>0<br>***1***	-0.003(-0.011,0.005)<br>-0.008(-0.059,0.043)<br>-<br>***-0.004(-0.008, -0.000)***	0.331<br>0.173<br>-<br>-	9.5%<br>46.1%<br>-<br>***0.0%***	0.000<br>0.001<br>-<br>***0.000***	4<br>2<br>0<br>2	-0.009(-0.021,0.004)<br>-0.000(-0.021,0.036)<br>-<br>-0.010(-0.027,0.006)	0.098<br>0.624<br>-<br>0.014	52.4%<br>0.0%<br>-<br>83.3%	0.000<br>0.000<br>-<br>0.000
	Cross-sectional<br>Case-control<br>Cohort	A(b)	2<br>0<br>0<br>2	-0.243(-0.655,0.169)<br>-<br>-<br>-0.243(-0.655,0.169)	0.125	57.5%	0.059	*1*<br>0<br>0<br>*1*	*0.100(0.000,0.200)*<br>-<br>-<br>*0.100(0.000,0.200)*	-	-	-
		B	3<br>-<br>*1*<br>2	-0.022(-0.072,0.027)<br>-<br>*-0.010(-0.075,0.055)*<br>-0.045(-0.139,0.027)	0.424<br>-<br>-<br>0.239	0.0%<br>-<br>-<br>27.9%	0.000<br>-<br>*0.000*<br>0.001	2<br>-<br>-<br>2	0.013(-0.059,0.085)<br>-<br>-<br>0.013(-0.059,0.085)	0.902<br>-<br>-<br>0.902	0.0%<br>-<br>-<br>0.0%	0.000<br>-<br>-<br>0.000
	**Setting**	A(a)	3<br>-	-0.003(-0.011,0.005)<br>-	0.331<br>-	9.5%<br>-	0.000<br>-	4<br>-	-0.009(-0.021,0.004)<br>-	0.098<br>-	52.4%<br>-	0.000<br>-

	Europe		**2**	**-0.004(-0.007, -0.000)**	**0.335**	**0.0%**	**0.000**	3	-0.009(-0.022,0.005)	0.046	67.4%	0.000
	Asia		-	-	-	-	-	-	-	-	-	-
	North America		*1*	*-0.050(-0.130,0.030)*	-	-	*0.000*	*1*	*-0.030(-0.155,0.095)*	-	-	*0.000*
	Other	A(b)	2	-0.243(-0.655,0.169)	0.125	57.5%	0.059	*1*	*0.100(0.000,0.200)*	-	-	-
			2	-0.243(-0.655,0.169)	0.125	57.5%	0.059	*1*	*0.100(0.000,0.200)*			
			-	-	-	-	-	-	-			
			-	-	-	-	-	-	-			
			-	-	-	-	-	-	-			
		B	3	-0.022(-0.072,0.027)	0.424	0.0%	0.000	2	0.013(-0.059,0.085)	0.902	0.0%	0.000
			1	*-0.040(-0.130,0.050)*	*0.204*	*38.0%*	*0.001*	*1*	*0.020(-0.110,0.160)*	-	-	-
			2	-0.010(-0.100,0.080)	-	-	0.000	*1*	*0.010(-0.080,0.090)*	-	-	-
			-	-	-	-	-	-	-	-	-	-
			-	-	-	-	-	-	-	-	-	-
		A(a)	3	-0.003(-0.011,0.005)	0.331	9.5%	0.000	4	-0.009(-0.021,0.004)	0.098	52.4%	0.000
			**2**	**-0.004(-0.011,-0.000)**	**0.335**	**0.0%**	**0.000**	2	-0.003(-0.007,0.001)	0.770	0.0%	0.000
			-	-	-	-	-	***1***	***-0.020(-0.033,-0..007)***	**-**	**-**	***0.000***
	**Matrix**		*1*	*-0.050(-0.130,0.030)*	-	-	*0.000*	*1*	*-0.030(-0.155,0.095)*	-	-	*0.000*
		A(b)	2	-0.243(-0.655,0.169)	-	-	-	*1*	*0.100(0.000,0.200)*	-	-	-
	Serum		*1*	*-0.100(-0.300,0.000)*				*1*	*0.100(0.000,0.200)*			
			-	-				-	-			
	Plasma		*1*	*-0.552(-1.110,0.006)*				-	-			
	Whole blood	B	3	-0.022(-0.072,0.027)	0.424	0.0%	0.000	2	0.013(-0.059,0.085)	0.902	0.0%	0.000
			3	-0.022(-0.072,0.027)	0.424	0.0%	0.000	2	0.013(-0.059,0.085)	0.902	0.0%	0.000
			-	-	-	-	-	-	-	-	-	-
			-	-	-	-	-	-	-	-	-	-

		A(a)	3<br>3<br>0	-0.003(-0.011,0.005)<br>-0.003(-0.011,0.005)<br>-	0.331<br>0.331<br>-	9.5%<br>9.5%<br>-	0.000<br>0.000<br>-	4<br>4<br>0	-0.009(-0.021,0.004)<br>-0.009(-0.021,0.004<br>-	0.098<br>0.098<br>-	52.4%<br>52.4%<br>-	0.000<br>0.000<br>-
	**Adjusted for: GA**<br>Yes<br>No	A(b)	2<br>1<br>1	-0.243(-0.655,0.169)<br>*-0.100(-0.300,0.000)*<br>*-0.552(-1.110,0.006)*	-	-	-	*1*<br>*1*<br>-	*0.100(0.000,0.200)*<br>*0.100(0.000,0.200)*<br>-	-	-	-
		B	3<br>2<br>*1*	-0.022(-0.072,0.027)<br>-0.040(-0.130,0.050)<br>*-0.010(-0.100,0.080)*	0.424<br>0.204<br>-	0.0%<br>38.0%<br>-	0.000<br>0.001<br>*0.000*	2<br>*1*<br>*1*	0.013(-0.059,0.085)<br>*0.020(-0.110,0.160)*<br>*0.010(-0.080,0.090)*	0.902<br>-<br>-	0.0%<br>-<br>-	0.000<br>-<br>-
		A(a)	3<br>**2**<br>*1*	-0.003(-0.011,0.005)<br>**-0.004(-0.007, -0.000)**<br>*-0.050(-0.130,0.030)*	0.331<br>**0.335**<br>-	9.5%<br>**0.0%**<br>-	0.000<br>**0.000**<br>*0.000*	4<br>3<br>*1*	-0.009(-0.021,0.004)<br>-0.009(-0.022,0.005)<br>*-0.030(-0.155,0.095)*	0.098<br>0.046<br>-	52.4%<br>67.4%<br>-	0.000<br>0.000<br>*0.000*
	**Adjusted for: Parity**<br>Yes<br>No	A(b)	2<br>0<br>2	-0.243(-0.655,0.169)<br>-<br>-0.243(-0.655,0.169)	0.125<br>-<br>0.125	57.5%<br>-<br>57.5%	0.059<br>-<br>0.059	*1*<br>-<br>*1*	*0.100(0.000,0.200)*<br>-<br>*0.100(0.000,0.200)*	-	-	-
		B	3<br>*1*<br>2	-0.022(-0.072,0.027)<br>*-0.010(-0.100,0.080)*<br>-0.040(-0.130,0.050)	0.424<br>*0.204*<br>-	0.0%<br>-<br>38.0%	0.000<br>*0.000*<br>0.001	2<br>*1*<br>*1*	0.013(-0.059,0.085)<br>*0.010(-0.080,0.090)*<br>*0.020(-0.110,0.160)*	0.902<br>-<br>-	0.0%<br>-<br>-	0.000<br>-<br>-
**PTB**	**Gender**<br>Male<br>Female	A(a)	4<br>2<br>2	0.943(0.681,1.305)<br>0.987(0.662,1.473)<br>0.898(0.414,1.948)	0.252<br>0.946<br>0.046	26.7%<br>0.0%<br>75.0%	0.029<br>0.000<br>0.033	4<br>2<br>2	0.880(0.669,1.158)<br>0.892(0.631,1.261)<br>0.886(0.519,1.511)	0.617<br>0.471<br>0.263	0.0%<br>0.0%<br>20.2%	0.000<br>0.000<br>0.035
	**Timing Category**<br>First trimester	A(a)	*1*<br>-<br>*1*	*1.160(0.788,1.707)*<br>-<br>*1.160(0.790,1.710)*	-	-	-	0	-	-	-	-

	Second trimester<br>Third trimester<br>Cord blood		-<br>-	-<br>-								
	**Study design**<br>Cross-sectional<br>Case-control<br>Cohort	A(a)	3<br>-<br>-<br>3	0.017(0.802,1.289)<br>-<br>-<br>0.017(0.802,1.289)	0.352<br>-<br>-<br>0.352	4.3%<br>-<br>-<br>4.3%	0.001<br>-<br>-<br>0.001	4<br>-<br>-<br>4	0.924(0.755,1.131)<br>-<br>-<br>0.924(0.755,1.131)	0.274<br>-<br>-<br>0.274	22.7%<br>-<br>-<br>22.7%	0.010<br>-<br>-<br>0.010
		A(b)	1<br>-<br>-<br>1	1.000(0.955,1.047)<br>-<br>-<br>1.000(0.953,1.044)	-	-	-	***1***<br>-<br>-<br>***1***	***1.577(1.036,2.402)***<br>**-**<br>-<br>***1.577(1.000,2.319)***	-	-	-
		B	3<br>-<br>-<br>3	1.178(0.772,1.797)<br>-<br>-<br>1.178(0.772,1.797)	0.864<br>-<br>-<br>0.864	0.0%<br>-<br>-<br>0.0%	0.000<br>-<br>-<br>0.000	**3**<br>-<br>-<br>**3**	**1.753(1.151,2.669)**<br>-<br>-<br>**1.753(1.151,2.669)**	**0.948**<br>-<br>-<br>**0.948**	**0.0%**<br>-<br>-<br>**0.0%**	**0.000**<br>-<br>-<br>**0.000**
	**Setting**<br>Europe<br>Asia<br>North America<br>Other	A(a)	3<br>2<br>*1*<br>-<br>-	1.017(0.802,1.289)<br>0.947(0.672,0.334)<br>*0.160(0.788,1.707)*<br>-<br>-	0.352<br>0.239<br>-<br>-<br>-	4.3%<br>27.9%<br>-<br>-<br>-	0.001<br>0.017<br>*0.000*<br>-<br>-	4<br>2<br>2<br>-<br>-	0.924(0.755,1.131)<br>1.116(0.593,2.100)<br>0.874(0.725,1.053)<br>-<br>-	0.274<br>0.912<br>0.086<br>-<br>-	22.7%<br>66.2%<br>0.0%<br>-<br>-	0.010<br>0.038<br>0.000<br>-<br>-
		A(b)	1<br>1<br>-<br>-<br>-	1.000(0.955,1.047)<br>1.000(0.953,1.044)<br>-<br>-<br>-	-	-	-	***1***<br>***1***<br>-<br>-<br>-	***1.577(1.036,2.402)***<br>***1.577(1.000,2.319)***<br>-<br>-<br>-	-	-	-

		B	3<br>*1*<br>-<br>2<br>-	1.178(0.772,1.797)<br>*1.000(0.466,2.145)*<br>-<br>1.266(0.762,2.104)<br>-	0.864<br>-<br>-<br>0.846<br>-	0.0%<br>-<br>-<br>0.0%<br>-	0.000<br>*0.000*<br>-<br>0.000<br>-	**3**<br>*1*<br>-<br>**2**<br>-	**1.753(1.151,2.669)**<br>*1.700(0.837,3.453)*<br>-<br>**1.783(1.057,3.005)**<br>-	**0.948**<br>-<br>-<br>**0.756**<br>-	**0.0%**<br>-<br>-<br>**0.0%**<br>-	**0.000**<br>*0.000*<br>-<br>**0.000**<br>-
	**Matrix**<br>Serum<br>Plasma<br>Whole blood	A(a)	3<br>-<br>2<br>*1*	1.017(0.802,1.289)<br>-<br>0.947(0.672,1.334)<br>*1.160(0.788,1.707)*	0.352<br>-<br>0.239<br>-	4.3%<br>-<br>27.9%<br>-	0.002<br>-<br>0.017<br>*0.000*	4<br>-<br>3<br>*1*	0.924(0.755,1.131)<br>-<br>0.977(0.714,1.338)<br>*0.860(0.613,1.206)*	0.274<br>-<br>0.155<br>-	22.7%<br>-<br>46.5%<br>-	0.010<br>-<br>0.037<br>*0.000*
		A(b)	1<br>-<br>1<br>-	1.000(0.955,1.047)<br>-<br>1.000(0.953,1.044)<br>-	-	-	-	***1***<br>-<br>***1***<br>-	***1.577(1.036,2.402)***<br>-<br>***1.577(1.000,2.319)***<br>-	-	-	-
		B	3<br>*1*<br>2<br>-	1.178(0.772,1.797)<br>*1.150(0.384,3.445)*<br>1.183(0.748,1.870)<br>-	0.864<br>-<br>0.590<br>-	0.0%<br>-<br>0.0%<br>-	0.000<br>*0.000*<br>0.000<br>-	**3**<br>*1*<br>**2**<br>-	**1.753(1.151,2.669)**<br>*2.060(0.720,5.892)*<br>**1.700(1.075,2.690)**<br>-	**0.948**<br>-<br>**1.000**<br>-	**0.0%**<br>-<br>-<br>-	**0.000**<br>*0.000*<br>**0.000**<br>-
	**Adjusted for: GA**<br>Yes<br>No	A(a)	3<br>*1*<br>2	1.017(0.802,1.289)<br>*1.160(0.788,1.707)*<br>0.947(0.672,1.334)	0.352<br>-<br>0.239	4.3%<br>-<br>27.9%	0.002<br>*0.000*<br>0.017	4<br>2<br>2	0.924(0.755,1.131)<br>0.974(0.725,1.053)<br>1.116(0.593,2.100)	0.274<br>0.912<br>0.086	22.7%<br>0.0%<br>66.2%	0.010<br>0.000<br>0.138
		A(b)	1<br>1<br>-	1.000(0.955,1.047)<br>1.000(0.953,1.044)<br>-	-	-	-	***1***<br>***1***<br>-	***1.577(1.036,2.402)***<br>***1.577(1.000,2.319)***<br>-	-	-	-
		B	3<br>*1*<br>2	1.178(0.772,1.797)<br>*1.300(0.733,2.305)*<br>1.047(0.559,1.958)	0.864<br>-<br>0.838	0.0%<br>-<br>0.0%	0.000<br>*0.000*<br>0.000	**3**<br>*1*<br>**2**	**1.753(1.151,2.669)**<br>*1.700(0.931,3.104)*<br>**1.805(1.003,3.248)**	**0.948**<br>-<br>**0.766**	**0.0%**<br>-<br>**0.0%**	**0.000**<br>*0.000*<br>**0.000**

	**Adjusted for:**<br>**Parity**<br>Yes<br>No	A(a)	3<br>3<br>-	1.017(0.802,1.289)<br>1.017(0.802,1.289)<br>-	0.352<br>0.352<br>-	4.3%<br>4.3%<br>-	0.001<br>0.001<br>-	4<br>4<br>0	0.924(0.755,1.131)<br>0.924(0.755,1.131)<br>-	0.274<br>0.274<br>-	22.7%<br>22.7%<br>-	0.010<br>0.010<br>-
		A(b)	1<br>1<br>-	1.000(0.955,1.047)<br>1.000(0.953,1.044)<br>-	-	-	-	***1***<br>***1***<br>-	***1.577(1.036,2.402)***<br>***1.577(1.000,2.319)***<br>-	-	-	-
		B	3<br>3<br>0	1.178(0.772,1.797)<br>1.178(0.772,1.797)<br>-	0.864<br>0.864<br>-	0.0%<br>0.0%<br>-	0.000<br>0.000<br>-	**3**<br>**3**<br>0	**1.753(1.151,2.669)**<br>**1.753(1.151,2.669)**<br>-	**0.948**<br>**0.948**<br>-	**0.0%**<br>**0.0%**<br>-	**0.000**<br>**0.000**<br>-
**LBW**	**Gender**<br>Male<br>Female	A(a)	0	-	-	-	-	6<br>3<br>3	1.105(0.846,1.443)<br>1.081(0.789,1.481)<br>1.181(0.687,2.031)	0.130<br>0.258<br>0.055	41.4%<br>26.2%<br>65.6%	0.044<br>0.021<br>0.147
		B	0	-	-	-	-	2<br>*1*<br>*1*	1.231(0.765,1.983)<br>*1.240(0.660,1.983)*<br>*1.220(0.590,2.530)*	0.974<br>-<br>-	0.0%<br>-<br>-	0.000<br>-<br>-
	**Timing Category**<br>First trimester<br>Second trimester<br>Third trimester<br>Cord blood	A(a)	*1*<br>*1*<br>-<br>-<br>-	*0.973(0.743,1.274)*<br>*0.973(0.738,1.267)*<br>-<br>-<br>-	-	-	-	0	-	-	-	-
	**Study design**<br>Cross-sectional	A(a)	5<br>2<br>-<br>3	1.074(0.826,1.396)<br>1.036(0.646,1.663)<br>-<br>1.148(0.813,1.621)	0.061<br>0.109<br>-<br>0.087	55.7%<br>54.9%<br>-<br>65.9%	0.045<br>0.096<br>-<br>0.041	5<br>3<br>-<br>*1*	1.074(0.826,1.663)<br>1.036(0.646,1.663)<br>-<br>*1.148(0.813,1.621)*	0.061<br>0.109<br>-<br>*0.087*	55.7%<br>54.9%<br>-<br>*65.9%*	0.045<br>0.096<br>-<br>*0.041*

	Case-control<br>Cohort	B	*1*<br>*1*<br>-<br>-	*1.040(0.635,1.703)*<br>*1.040(0.630,1.690)*<br>-<br>-	-	-	-	*1*<br>-<br>-<br>*1*	*1.230(0.765,1.977)*<br>-<br>-<br>*1.230(0.770,1.990)*	-	-	-
	**Setting**<br>Europe<br>Asia<br>North America<br>Other	A(a)	5<br>*1*<br>4<br>-<br>-	1.074(0.826,1.396)<br>*1.380(1.019,1.869)*<br>0.965(0.748,1.245)<br>-<br>-	0.061<br>-<br>0.209<br>-<br>-	55.7%<br>-<br>33.9%<br>-<br>-	0.045<br>*0.000*<br>0.022<br>-<br>-	5<br>***1***<br>4<br>-<br>-	1.074(0.826,1.396)<br>***1.380(1.019,1.869)***<br>0.965(0.748,1.245)<br>-<br>-	0.061<br>-<br>0.209<br>-<br>-	55.75<br>-<br>33.95<br>-<br>-	***0.045***<br>0.000<br>0.022
		B	*1*<br>-<br>*1*<br>-<br>-	*1.040(0.635,1.703)*<br>-<br>*1.040(0.630,1.690)*<br>-<br>-	-	-	-	*1*<br>*1*<br>-<br>-<br>-	*1.230(0.765,1.977)*<br>*1.230(0.770,1.990)*<br>-<br>-<br>-	-	-	-
	**Matrix**<br>Serum<br>Plasma<br>Whole blood	A(a)	5<br>4<br>*1*<br>-	1.074(0.826,1.396)<br>1.135(0.778,1.657)<br>*0.970(0.743,1.266)*<br>-	0.061<br>0.036<br>-<br>-	55.7%<br>65.0%<br>-<br>-	0.045<br>0.088<br>0.000<br>-	5<br>4<br>*1*<br>-	1.074(0.826,1.396)<br>1.135(0.778,1.657)<br>*0.970(0.743,1.266)*<br>-	0.061<br>0.036<br>-<br>-	55.7%<br>65.0%<br>-<br>-	0.045<br>0.088<br>0.000<br>-
		B	*1*<br>*1*<br>-<br>-	*1.040(0.635,1.703)*<br>*1.040(0.630,1.690)*<br>-<br>-	-	-	-	*1*<br>*1*<br>-<br>-	*1.230(0.765,1.977)*<br>*1.230(0.770,1.990)*<br>-<br>-	-	-	-
	**Adjusted for: GA**<br>Yes	A(a)	2<br>*1*<br>*1*	0.967(0.795,1.176)<br>*0.960(0.720,1.270)*<br>*0.973(0.738,1.267)*	-	-	-	5<br>5<br>0	1.074(0.826,1.396)	0.061	55.7%	0.045

	No	B	1<br>1<br>-	1.040(0.635,1.703)<br>1.040(0.630,1.690)<br>-	-	-	-	1<br>1<br>-	1.230(0.765,1.977)<br>1.230(0.770,1.990)<br>-	-	-	-
	**Adjusted for: Parity**<br>Yes<br>No	A(a)	2<br>2<br>0	0.967(0.795,1.176)<br>0.967(0.795,1.176)<br>-	0.947<br>0.947<br>-	0.0%<br>0.0%<br>-	0.000<br>0.000<br>-	5<br>5<br>0	1.074(0.826,1.396)<br>1.074(0.826,1.396)<br>-	0.061<br>0.061<br>-	55.7%<br>55.7%<br>-	0.045<br>0.045<br>-
		B	1<br>1<br>-	1.040(0.635,1.703)<br>1.040(0.630,1.690)<br>-	-	-	-	1<br>1<br>-	1.230(0.765,1.977)<br>1.230(0.770,1.990)<br>-	-	-	-
**SGA**	**Gender**<br>Male<br>Female	A(a)	2<br>1<br>1	0.894(0.614, 1.302)<br>1.082(0.580,2.024)<br>0.802(0.499,1.281)	0.452	0.0%	0.000	2<br>1<br>1	0.815(0.525,1.265)<br>0.815(0.525,1.265)<br>0.701(0.390,1.224)	0.419<br>-<br>-	0.0%<br>-<br>-	0.000<br>-<br>-
	**Timing Category**<br>First trimester<br>Second trimester<br>Third trimester<br>Cord blood	A(a)	2<br>2<br>-<br>-<br>-	1.034(0.758,1.412)<br>1.034(0.758,1.412)<br>-<br>-<br>-	0.305<br>0.305<br>-<br>-<br>-	5.0%<br>5.0%<br>-<br>-<br>-	0.002<br>0.002<br>-<br>-<br>-	0	-	-	-	-
	**Study design**<br>Cross-sectional<br>Case-control	A(a)	2<br>0<br>0<br>2	1.034(0.758,1.412)<br>-<br>-<br>1.034(0.758,1.412)	0.305<br>-<br>-<br>0.305	5.0%<br>-<br>-<br>5.0%	0.002<br>-<br>-<br>0.002	3<br>-<br>3<br>-	0.963(0.621， 1.494)<br>-<br>0.963(0.621， 1.494)<br>-	0.111<br>-<br>0.111<br>-	54.6%<br>-<br>54.6%<br>-	0.081<br>-<br>0.081<br>-

	Cohort	B	*1*<br>-<br>-<br>*1*	*0.800(0.367,1.744)*<br>-<br>-<br>*0.800(0.400,1.900)*	-	-	-	*1*<br>*1*<br>-<br>-	*1.500(0.612,3.674)*<br>*1.500(0.600,3.600)*<br>-	-	-	-
	**Setting**<br>Europe<br>Asia<br>North America<br>Other	A(a)	2<br>2<br>-<br>-<br>-	1.034(0.758,1.412)<br>1.034(0.758,1.412)<br>-<br>-<br>-	0.305<br>0.305<br>-<br>-<br>-	5.0%<br>5.0%<br>-<br>-<br>-	0.002<br>0.002<br>-<br>-<br>-	3<br>**2**<br>*1*<br>-<br>-	0.963(0.621,1.494)<br>**1.135(0.544,2.369)**<br>*0.760(0.470,1.229)*<br>-<br>-	0.111<br>**0.060**<br>-<br>-<br>-	54.6%<br>**71.7%**<br>-<br>-<br>-	0.081<br>**0.204**<br>*0.000*<br>-<br>-
		B	*1*<br>*1*<br>-<br>-<br>-	*0.800(0.367,1.744)*<br>*0.800(0.400,1.900)*<br>-<br>-<br>-	-	-	-	*1*<br>*1*<br>-<br>-<br>-	*1.500(0.612,3.674)*<br>*1.500(0.600,3.600)*<br>-<br>-<br>-	-	-	-
	**Matrix**<br>Serum<br>Plasma<br>Whole blood	A(a)	2<br>-<br>2<br>-	1.034(0.758,1.412)<br>-<br>1.034(0.758,1.412)<br>-	0.305<br>-<br>0.305<br>-	5.0%<br>-<br>5.0%<br>-	0.002<br>-<br>0.002<br>-	3<br>-<br>3<br>-	0.963(0.621,1.494)<br>-<br>0.963(0.621,1.494)<br>-	0.111<br>-<br>0.111<br>-	54.6%<br>-<br>54.6%<br>-	0.0817<br>-<br>0.0817<br>-
		B	*1*<br>-<br>*1*<br>-	*0.800(0.367,1.744)*<br>-<br>*0.800(0.400,1.900)*<br>-	-	-	-	*1*<br>-<br>*1*<br>-<br>-	*1.500(0.612,3.674)*<br>-<br>*1.500(0.600,3.600)*<br>-<br>-	-	-	-
	**Adjusted for: GA**<br>Yes	A(a)	2<br>0<br>2	1.034(0.758,1.412)<br>-<br>1.034(0.758,1.412)	0.305<br>-<br>0.305	5.0%<br>-<br>5.0%	0.002<br>-<br>0.002	3<br>*1*<br>2	0.963(0.621,1.494)<br>*0.760(0.470,1.229)*<br>1.135(0.544,2.369)	0.111<br>-<br>0.060	54.6%<br>-<br>71.7%	0.081<br>*0.000*<br>0.204

	No	B	*1*<br>-<br>*1*	*0.800(0.367,1.744)*<br>-<br>*0.800(0.400,1.900)*	-	-	-	*1*<br>-<br>*1*	*1.500(0.612,3.674)*<br>-<br>*1.500(0.600,3.600)*	-	-	-
	**Adjusted for: Parity**	A(a)	2<br>2<br>0	1.034(0.758,1.412)<br>1.034(0.758,1.412)<br>-	0.305<br>0.305<br>-	5.0%<br>5.0%<br>-	0.002<br>0.002<br>-	3<br>3<br>0	0.963(0.621,1.494)<br>0.963(0.621,1.494)<br>-	0.111<br>0.111<br>-	54.6%<br>54.6%<br>-	0.0817<br>0.0817<br>-
	Yes<br>No	B	**1**<br>**1**<br>**-**	***0.800(0.367,1.744)***<br>***0.800(0.400,1.900)***<br>**-**	-	-	-	*1*<br>*1*<br>-	*1.500(0.612,3.674)*<br>*1.500(0.600,3.600)*<br>-	-	-	-
**Birth Outcome indicators**	**Study characteristics**		**N**	**Summary β or OR (95% CI)**	**P value**	**I-squared**	**Tau-squared**	**N**	**Summary β or OR (95% CI)**	**P value**	**I-squared**	**Tau-squared**
			**PFHpS**					**PFTrDA**				
**BW/g**	**Gender**	A(a)	2	-	-	-	-	0<br>0<br>0	-<br>-<br>-	-<br>-<br>-	-<br>-<br>-	-<br>-<br>-
	Male<br>Female	B	**5**<br>3<br>2	**-74.267(-118.616, -29.918)**<br>-45.073(-104.248,140102)<br>-74.267(-118.616, -29.918)	**0.439**<br>0.749<br>0.304	**0.0%**<br>5.3%<br>0.0%	**0.000**<br>0.000<br>133.581	0<br>0<br>0	-<br>-<br>-	-<br>-<br>-	-<br>-<br>-	-<br>-<br>-
	**Timing Category**<br>First trimester	A(a)	0	-	-	-	-	1<br>0<br>0<br>0	*-48.620 (-119.080, 21.840)*<br>-<br>-<br>-	-<br>-<br>-<br>-	0.0%<br>-<br>-<br>-	0.0000<br>-<br>-<br>-

	Second trimester<br>Third trimester<br>Cord blood							1	-<br>*-48.620 (-119.080, 21.840)*	-	0.0%	0.0000
		A(b)	1<br>-<br>1<br>-<br>-	-150.000(-355.000,55.000)<br>-<br>-150.000(-360.000,50.000)<br>-<br>-	-	-	-	0<br>0<br>0<br>0<br>0	-<br>-<br>-<br>-<br>-	-<br>-<br>-<br>-<br>-	-<br>-<br>-<br>-<br>-	-<br>-<br>-<br>-<br>-
		B	**2**<br>***1***<br>*1*<br>-<br>-	**-70.267(-128.462, -12.071)**<br>***-102.600(-169.000, -36.200)***<br>*-43.000(-100.000,14.000)*<br>-<br>-	**0.182**<br>-<br>-<br>-<br>-	**43.9%**<br>-<br>-<br>-<br>-	**779.328**<br>-<br>-<br>-<br>-	1<br>0<br>0<br>0<br>1	*-25.100 (129.700, 79.500)*<br>-<br>-<br>-<br>*-25.100 (129.700, 79.500)*	-<br>-<br>-<br>-<br>-	0.0%<br>-<br>-<br>-<br>0.0%	0.0000<br>-<br>-<br>-<br>0.0000
	**Study design**<br>Cross-sectional<br>Case-control<br>Cohort	A(a)	0	-	-	-	-	2<br>1<br>0<br>1	-13.799 (-31.447, 3.848)<br>*-48.620 (-119.080, 21.840)*<br>-<br>*-11.471 (-29.575, 6.633)*	0.317<br>-<br>-<br>-	0.2%<br>0.0%<br>-<br>0.05	1.1712<br>0.0000<br>-<br>0.0000
		B	0	-	-	-	-	1<br>0<br>0	*-25.100 (-129.700, 79.500)*<br>-	-<br>-<br>-	0.0%<br>-<br>-	0.0000<br>-<br>-

								1	-<br>*-25.100 (-129.700, 79.500)*	-	0.0%	0.0000
	**Setting**<br>Europe<br>Asia<br>North America<br>Other	A(a)	0	-	-	-	-	2<br>0<br>2<br>0<br>0	*-13.799 (-31.447, 3.848)*<br>-<br>*-13.799 (-31.447, 3.848)*<br>-<br>-	0.317<br>-<br>0.317<br>-<br>-	0.2%<br>-<br>0.2%<br>-<br>-	1.1712<br>-<br>1.1712<br>-<br>-
		B	0	-	-	-	-	1<br>0<br>1<br>0<br>0	*-25.100 (129.700, 79.500)*<br>-<br>*-25.100 (129.700, 79.500)*<br>-<br>-	-<br>-<br>-<br>-<br>-	0.0%<br>-<br>0.0%<br>-<br>-	0.0000<br>-<br>0.0000<br>-<br>-
	**Matrix**<br>Serum<br>Plasma<br>Whole blood	A(a)	0	-	-	-	-	2<br>1<br>1<br>0	-13.799 (-31.447, 3.848)<br>*-48.620 (-119.080, 21.840)*<br>*-11.471 (-29.575, 6.633)*<br>-	0.317<br>-<br>-<br>-	0.2%<br>0.0%<br>0.05<br>-	1.1712<br>0.0000<br>0.0000<br>-
		B	0	-	-	-	-	1<br>1<br>0<br>0	*-25.100 (129.700, 79.500)*<br>*-25.100 (129.700, 79.500)*<br>-<br>-	-<br>-<br>-<br>-	0.0%<br>0.0%<br>-<br>-	0.0000<br>0.0000<br>-<br>-

	**Adjusted for: GA**<br>Yes<br>No	A(a)	0	-	-	-	-	2<br>2<br>0	-13.799 (-31.447, 3.848)<br>-13.799 (-31.447, 3.848)<br>-	0.317<br>0.317<br>-	0.2%<br>0.2%<br>-	1.1712<br>1.1712<br>-
		B	0	-	-	-	-	1<br>0<br>1	*-25.100 (129.700, 79.500)*<br>-<br>*-25.100 (129.700, 79.500)*	-<br>-<br>-	0.0%<br>-<br>0.0%	0.0000<br>-<br>0.0000
	**Adjusted for: Parity**<br>Yes<br>No	A(a)	0	-	-	-	-	2<br>2<br>0	-13.799 (-31.447, 3.848)<br>-13.799 (-31.447, 3.848)<br>-	0.317<br>0.317<br>-	0.2%<br>0.2%<br>-	1.1712<br>1.1712<br>-
		B	0	-	-	-	-	1<br>1<br>0	*-25.100 (129.700, 79.500)*<br>*-25.100 (129.700, 79.500)*<br>-	-<br>-<br>-	0.0%<br>0.0%<br>-	0.0000<br>0.0000<br>-
		A(b)	***1***<br>-<br>***1***	***-0.820(-1.295, -0.345)***<br>-<br>***-0.820(-1.290, -0.340)***	-	-	-	0<br>0<br>0	-<br>-<br>-	-<br>-<br>-	-<br>-<br>-	-<br>-<br>-
		A(b)	*1*<br>-<br>-	*-1.667(-3.750,0.417)*<br>-<br>-	-	-	-	0<br>0<br>0	-<br>-<br>-	-<br>-<br>-	-<br>-<br>-	-<br>-<br>-

			1	*-1.667(-3.333,0.833)*				0	-	-	-	-
		A(b)	*1*<br>*1*<br>-<br>-<br>-	*-1.667(-3.750,0.417)*<br>*-1.667(-3.333,0.833)*<br>-<br>-<br>-	-	-	-	0<br>0<br>0<br>0<br>0	-<br>-<br>-<br>-<br>-	-<br>-<br>-<br>-<br>-	-<br>-<br>-<br>-<br>-	-<br>-<br>-<br>-<br>-
		A(b)	*1*<br>*1*<br>-<br>-	*-1.667(-3.750,0.417)*<br>*-1.667(-3.333,0.833)*<br>-<br>-	-	-	-	0<br>0<br>0<br>0	-<br>-<br>-<br>-	-<br>-<br>-<br>-	-<br>-<br>-<br>-	-<br>-<br>-<br>-
		A(b)	*1*<br>*1*<br>-	*-1.667(-3.750,0.417)*<br>*-1.667(-3.333,0.833)*<br>-	-	-	-	0<br>0<br>0	-<br>-<br>-	-<br>-<br>-	-<br>-<br>-	-<br>-<br>-
		A(b)	*1*<br>-<br>*1*	*-1.667(-3.750,0.417)*<br>-<br>*-1.667(-3.333,0.833)*	-	-	-	0<br>0<br>0	-<br>-<br>-	-<br>-<br>-	-<br>-<br>-	-<br>-<br>-
		B	0	-	-	-	-	1<br>0<br>0<br>0<br>1	*0.080 (-0.220, 0.370)*<br>-<br>-<br>-<br>*0.080 (-0.220, 0.370)*	-<br>-<br>-<br>-<br>-	0.0%<br>-<br>-<br>-<br>0.0%	0.0000<br>-<br>-<br>-<br>0.0000
	**Study design**<br>Cross-sectional	A(a)	0	-	-	-	-	1<br>0<br>0<br>1	*0.046 (-0.053, 0.144)*<br>-<br><br>*0.046 (-0.053, 0.144)*	-<br>-<br>-<br>-	0.0%<br>-<br>-<br>0.0%	0.0000<br>-<br>-<br>0.0000

	Case-control<br>Cohort	A(b)	*1*<br>-<br>-<br>*1*	*0.000(-1.250,1.250)*<br>-<br>-<br>*0.000(-1.667,0.833)*	-	-	-	0<br>0<br>0<br>0	-<br>-<br>-<br>-	-<br>-<br>-<br>-	-<br>-<br>-<br>-	-<br>-<br>-<br>-
		B	0	-	-	-	-	1<br>0<br>0<br>1	*0.080 (-0.220, 0.370)*<br>-<br>-<br>*0.080 (-0.220, 0.370)*	-<br>-<br>-<br>-	0.0%<br>-<br>-<br>0.0%	0.0000<br>-<br>-<br>0.0000
	**Setting**<br>Europe<br>Asia<br>North America<br>Other	A(a)	0	-	-	-	-	1<br>0<br>1<br>0<br>0	*0.046 (-0.053, 0.144)*<br>-<br>*0.046 (-0.053, 0.144)*<br>-<br>-	-<br>-<br>-<br>-<br>-	0.0%<br>-<br>0.0%<br>-<br>-	0.0000<br>-<br>0.0000<br>-<br>-
		A(b)	*1*<br>*1*<br>-<br>-	*0.000(-1.250,1.250)*<br>*0.000(-1.667,0.833)*<br>-<br>-	-	-	-	0<br>0<br>0<br>0<br>0	-<br>-<br>-<br>-<br>-	-<br>-<br>-<br>-<br>-	-<br>-<br>-<br>-<br>-	-<br>-<br>-<br>-<br>-
		B	0	-	-	-	-	1<br>0<br>1<br>0<br>0	*0.080 (-0.220, 0.370)*<br>-<br>*0.080 (-0.220, 0.370)*<br>-<br>-	-<br>-<br>-<br>-<br>-	0.0%<br>-<br>0.0%<br>-<br>-	0.0000<br>-<br>0.0000<br>-<br>-
	**Matrix**<br>Serum	A(a)	0	-	-	-	-	1<br>0<br>1<br>0	*0.046 (-0.053, 0.144)*<br>-<br>*0.046 (-0.053, 0.144)*<br>-	-<br>-<br>-<br>-	0.0%<br>-<br>0.0%<br>-	0.0000<br>-<br>0.0000<br>-

	Plasma<br>Whole blood	A(b)	*1*<br>*1*<br>-<br>-	*0.000(-1.250,1.250)*<br>*0.000(-1.667,0.833)*<br>-<br>-	-	-	-	0<br>0<br>0<br>0	-<br>-<br>-<br>-	-<br>-<br>-<br>-	-<br>-<br>-<br>-	-<br>-<br>-<br>-
		B	0	-	-	-	-	1<br>1<br>0<br>0	*0.080 (-0.220, 0.370)*<br>*0.080 (-0.220, 0.370)*<br>-<br>-	-<br>-<br>-<br>-	0.0%<br>0.0%<br>-<br>-	0.0000<br>0.0000<br>-<br>-
	**Adjusted for: GA**<br>Yes<br>No	A(a)	0	-	-	-	-	1<br>1<br>0	*0.046 (-0.053, 0.144)*<br>*0.046 (-0.053, 0.144)*<br>-	-<br>-<br>-	0.0%<br>0.0%<br>-	0.0000<br>0.0000<br>-
		A(b)	*1*<br>*1*<br>-	*0.000(-1.250,1.250)*<br>*0.000(-1.667,0.833)*<br>-	-	-	-	0<br>0<br>0	-<br>-<br>-	-<br>-<br>-	-<br>-<br>-	-<br>-<br>-
	**Adjusted for: Parity**<br>Yes<br>No	A(a)	0	-	-	-	-	1<br>1<br>0	*0.046 (-0.053, 0.144)*<br>*0.046 (-0.053, 0.144)*<br>-	-<br>-<br>-	0.0%<br>0.0%<br>-	0.0000<br>0.0000<br>-
		A(b)	*1*<br>-<br>*1*	*0.000(-1.250,1.250)*<br>-<br>*0.000(-1.667,0.833)*	-	-	-	0<br>0<br>0	-<br>-<br>-	-<br>-<br>-	-<br>-<br>-	-<br>-<br>-
		A(b)	*1*<br>-<br>*1*	*0.000(-1.250,1.250)*<br>-<br>*0.000(-1.667,0.833)*	-	-	-	0<br>0<br>0	-<br>-<br>-	-<br>-<br>-	-<br>-<br>-	-<br>-<br>-
	**Timing Category**	A(a)	*1*<br>*1*<br>-	*0.000(-1.250,1.250)*<br>*0.000(-1.667,0.833)*<br>-	-	-	-	0<br>0<br>0	-<br>-<br>-	-<br>-<br>-	-<br>-<br>-	-<br>-<br>-

	First trimester		-	-				0	-	-	-	-
	Second trimester		-	-				0	-	-	-	-
	Third trimester		***1***	***-2.000(-3.300, -0.700)***				0	-	-	-	-
			1	***-2.000(-3.300, -0.700)***				0	-	-	-	-
	Cord blood	B	-	-	-	-	-	0	-	-	-	-
			-	-				0	-	-	-	-
			-	-				0	-	-	-	-
	**Study design**	A(a)	**2**	**-0.232(-0.367,-0.097)**	**0.892**	**0.0%**	**0.000**	1	*-0.089 (-0.205, 0.375)*	-	0.0%	0.0000
	Cross-sectional		*1*	*-0.150(-1.340,1.050)*	-	-	-	0	-	-	-	-
	Case-control		-	-	-	-	-	0	-	-	-	-
	Cohort		*1*	*-0.233(-0.369,-0.097)*	-	-	-	1	*-0.089 (-0.205, 0.375)*	-	0.0%	0.0000
	**Setting**	A(a)	**2**	**-0.232(-0.367, -0.097)**	**0.892**	**0.0%**	**0.000**	1	*-0.089 (-0.205, 0.375)*	-	0.0%	0.0000
	Europe		**2**	**-0.232(-0.367, -0.097)**	-	-	-	0	-	-	-	-
	Asia		-	-	-	-	-	1	*-0.089 (-0.205, 0.375)*	-	0.0%	0.0000
	America		-	-	-	-	-	0	-	-	-	-
	Other		-	-	-	-	-	0	-	-	-	-
	**Matrix**	A(a)	**2**	**-0.232(-0.367, -0.097)**	**0.892**	**0.0%**	**0.000**	1	*-0.089 (-0.205, 0.375)*	-	0.0%	0.0000
	Serum		*1*	*-0.150(-1.340,1.050)*	-	-	-	0	-	-	-	-
	Plasma		***1***	***-0.233(-0.369, -0.097)***	-	-	-	1	*-0.089 (-0.205, 0.375)*	-	0.0%	0.0000
	Whole blood		-	-	-	-	-	0	-	-	-	-

	**Adjusted for: GA**<br>Yes<br>No	A(a)	**2**<br>*1*<br>***1***	**-0.232(-0.367,-0.097)**<br>*-0.150(-1.340,1.050)*<br>***-0.233(-0.369,-0.097)***	**0.892**<br>-<br>-	**0.0%**<br>-<br>-	**0.000**<br>-<br>-	1<br>1<br>0	*-0.089 (-0.205, 0.375)*<br>*-0.089 (-0.205, 0.375)*<br>-	-<br>-<br>-	0.0%<br>0.0%<br>-	0.0000<br>0.0000<br>-
	**Adjusted for: Parity**<br>Yes<br>No	A(a)	**2**<br>**2**<br>-	**-0.232(-0.367, -0.097)**<br>**-0.232(-0.367, -0.097)**<br>-	**0.892**<br>**0.892**<br>-	**0.0%**<br>**0.0%**<br>-	**0.000**<br>**0.000**<br>-	1<br>1<br>0	*-0.089 (-0.205, 0.375)*<br>*-0.089 (-0.205, 0.375)*<br>-	-<br>-<br>-	0.0%<br>0.0%<br>-	0.0000<br>0.0000<br>-
**PI/ g.cm3**	**Gender**<br>Male<br>Female	A(a)	0	-	-	-	-	0<br>0<br>0	-<br>-<br>-	-<br>-<br>-	-<br>-<br>-	-<br>-<br>-
		A(b)	*1*<br>-<br>*1*	*-0.010(-0.075,0.055)*<br>-<br>*-0.010(-0.080,0.050)*	-	-	-	0<br>0<br>0	-<br>-<br>-	-<br>-<br>-	-<br>-<br>-	-<br>-<br>-
	**Timing Category**<br>First trimester<br>Second trimester<br>Third trimester<br>Cord blood	A(a)	0	-	-	-	-	0<br>0<br>0<br>0<br>0	-<br>-<br>-<br>-<br>-	-<br>-<br>-<br>-<br>-	-<br>-<br>-<br>-<br>-	-<br>-<br>-<br>-<br>-
		A(b)	*1*<br>-<br>*1*<br>-<br>-	*-0.400(-0.950,0.150)*<br>-<br>*-0.400(-0.900,0.200)*<br>-<br>-	-	-	-	0<br>0<br>0<br>0<br>0	-<br>-<br>-<br>-<br>-	-<br>-<br>-<br>-<br>-	-<br>-<br>-<br>-<br>-	-<br>-<br>-<br>-<br>-

		B	*1*<br>-<br>*1*<br>-<br>-	*-0.100(-0.245,0.045)*<br>-<br>*-0.100(-0.250,0.040)*<br>-<br>-	-	-	-	1<br>0<br>0<br>0<br>1	*-0.050 (-0.130, 0.030)*<br>-<br>-<br>-<br>*-0.050 (-0.130, 0.030)*	-<br>-<br>-<br>-<br>-	0.0%<br>-<br>-<br>-<br>0.0%	0.0000<br>-<br>-<br>-<br>0.0000
	**Study design**<br>Cross-sectional<br>Case-control<br>Cohort	A(a)	0	-	-	-	-	0<br>0<br>0<br>0	-<br>-<br>-<br>-	-<br>-<br>-<br>-	-<br>-<br>-<br>-	-<br>-<br>-<br>-
		A(b)	*1*<br>-<br>-<br>*1*	*-0.400(-0.950,0.150)*<br>-<br>-<br>*-0.400(-0.900,0.200)*	-	-	-	0<br>0<br>0<br>0	-<br>-<br>-<br>-	-<br>-<br>-<br>-	-<br>-<br>-<br>-	-<br>-<br>-<br>-
		B	*1*<br>-<br>-<br>*1*	*-0.100(-0.245,0.045)*<br>-<br>-<br>*-0.100(-0.250,0.040)*	-	-	-	1<br>0<br>0<br>1	*-0.050 (-0.130, 0.030)*<br>-<br>-<br>*-0.050 (-0.130, 0.030)*	-<br>-<br>-<br>-	0.0%<br>-<br>-<br>0.0%	0.0000<br>-<br>-<br>0.0000
	**Setting**<br>Europe<br>Asia<br>North America<br>Other	A(a)	0	-	-	-	-	0<br>0<br>0<br>0<br>0	-<br>-<br>-<br>-<br>-	-<br>-<br>-<br>-<br>-	-<br>-<br>-<br>-<br>-	-<br>-<br>-<br>-<br>-
		A(b)	*1*<br>*1*<br>-<br>-<br>-	*-0.400(-0.950,0.150)*<br>*-0.400(-0.900,0.200)*<br>-<br>-<br>-	-	-	-	0<br>0<br>0<br>0<br>0	-<br>-<br>-<br>-<br>-	-<br>-<br>-<br>-<br>-	-<br>-<br>-<br>-<br>-	-<br>-<br>-<br>-<br>-

		B	*1*<br>*1*<br>-<br>-<br>-	*-0.100(-0.245,0.045)*<br>*-0.100(-0.250,0.040)*<br>-<br>-<br>-	-	-	-	1<br>0<br>1<br>0<br>0	*-0.050 (-0.130, 0.030)*<br>-<br>*-0.050 (-0.130, 0.030)*<br>-<br>-	-<br>-<br>-<br>-<br>-	0.0%<br>-<br>0.0%<br>-<br>-	0.0000<br>-<br>0.0000<br>-<br>-
	**Matrix**<br>Serum<br>Plasma<br>Whole blood	A(a)	0	-	-	-	-	0<br>0<br>0<br>0	-<br>-<br>-<br>-	-<br>-<br>-<br>-	-<br>-<br>-<br>-	-<br>-<br>-<br>-
		A(b)	*1*<br>*1*<br>-<br>-	*-0.400(-0.950,0.150)*<br>*-0.400(-0.900,0.200)*<br>-<br>-	-	-	-	0<br>0<br>0<br>0	-<br>-<br>-<br>-	-<br>-<br>-<br>-	-<br>-<br>-<br>-	-<br>-<br>-<br>-
		B	*1*<br>*1*<br>-<br>-	*-0.100(-0.245,0.045)*<br>*-0.100(-0.250,0.040)*<br>-<br>-	-	-	-	1<br>1<br>0<br>0	*-0.050 (-0.130, 0.030)*<br>*-0.050 (-0.130, 0.030)*<br>-<br>-	-<br>-<br>-<br>-	0.0%<br>0.0%<br>-<br>-	0.0000<br>0.0000<br>-<br>-
	**Adjusted for: GA**<br>Yes<br>No	A(a)	0	-	-	-	-	0<br>0<br>0	-<br>-<br>-	-<br>-<br>-	-<br>-<br>-	-<br>-<br>-
		A(b)	*1*<br>*1*<br>-	*-0.400(-0.950,0.150)*<br>*-0.400(-0.900,0.200)*<br>-	-	-	-	0<br>0<br>0	-<br>-<br>-	-<br>-<br>-	-<br>-<br>-	-<br>-<br>-
		B	*1*<br>*1*<br>-	*-0.100(-0.245,0.045)*<br>*-0.100(-0.250,0.040)*<br>-	-	-	-	1<br>0<br>1	*-0.050 (-0.130, 0.030)*<br>-<br>*-0.050 (-0.130, 0.030)*	-<br>-<br>-	0.0%<br>-<br>0.0%	0.0000<br>-<br>0.0000

	**Adjusted for:**<br>**Parity**<br>Yes<br>No	A(a)	0	-	-	-	-	0<br>0<br>0	-<br>-<br>-	-<br>-<br>-	-<br>-<br>-	-<br>-<br>-
		A(b)	*1*<br>-<br>*1*	*-0.400(-0.950,0.150)*<br>-<br>*-0.400(-0.900,0.200)*	-	-	-	0<br>0<br>0	-<br>-<br>-	-<br>-<br>-	-<br>-<br>-	-<br>-<br>-
		A(b)	*1*<br>-<br>*1*	*-0.100(-0.245,0.045)*<br>-<br>*-0.100(-0.250,0.040)*	-	-	-	1<br>1<br>0	*-0.050 (-0.130, 0.030)*<br>*-0.050 (-0.130, 0.030)*<br>-	-<br>-<br>-	0.0%<br>0.0%<br>-	0.0000<br>0.0000<br>-
**PTB**	**Study design**<br>Cross-sectional<br>Case-control<br>Cohort	A(a)	*1*<br>-<br>-<br>*1*	*0.950(0.782,1.154)*<br>-<br>-<br>*0.950(0.780,1.150)*	-	-	-	0<br>0<br>0<br>0	-<br>-<br>-<br>-	-<br>-<br>-<br>-	-<br>-<br>-<br>-	-<br>-<br>-<br>-
		B	*1*<br>-<br>-<br>*1*	*1.800(0.837,3.871)*<br>-<br>-<br>*1.800(0.800,3.700)*	-	-	-	0<br>0<br>0<br>0	-<br>-<br>-<br>-	-<br>-<br>-<br>-	-<br>-<br>-<br>-	-<br>-<br>-<br>-
	**Setting**<br>Europe<br>Asia<br>North America<br>Other	A(a)	*1*<br>*1*<br>-<br>-	*0.950(0.782,1.154)*<br>*0.950(0.780,1.150)*<br>-<br>-	-	-	-	0<br>0<br>0<br>0<br>0	-<br>-<br>-<br>-<br>-	-<br>-<br>-<br>-<br>-	-<br>-<br>-<br>-<br>-	-<br>-<br>-<br>-<br>-
		B	*1*<br>*1*<br>-<br>-	*0.950(0.782,1.154)*<br>*0.950(0.780,1.150)*<br>-<br>-	-	-	-	0<br>0<br>0<br>0	-<br>-<br>-<br>-	-<br>-<br>-<br>-	-<br>-<br>-<br>-	-<br>-<br>-<br>-

								0	-	-	-	-
	**Matrix**<br>Serum<br>Plasma<br>Whole blood	A(a)	*1*<br>-<br>*1*<br>-	*0.950(0.782,1.154)*<br>-<br>*0.950(0.780,1.150)*<br>-	-	-	-	0<br>0<br>0<br>0	-<br>-<br>-<br>-	-<br>-<br>-<br>-	-<br>-<br>-<br>-	-<br>-<br>-<br>-
		B	*1*<br>-<br>*1*<br>-	*0.950(0.782,1.154)*<br>-<br>*0.950(0.780,1.150)*<br>-	-	-	-	0<br>0<br>0<br>0	-<br>-<br>-<br>-	-<br>-<br>-<br>-	-<br>-<br>-<br>-	-<br>-<br>-<br>-
	**Adjusted for: GA**<br>Yes<br>No	A(a)	*1*<br>-<br>*1*	*0.950(0.782,1.154)*<br>-<br>*0.950(0.780,1.150)*	-	-	-	0<br>0<br>0	-<br>-<br>-	-<br>-<br>-	-<br>-<br>-	-<br>-<br>-
		B	*1*<br>-<br>*1*	*0.950(0.782,1.154)*<br>-<br>*0.950(0.780,1.150)*	-	-	-	0<br>0<br>0	-<br>-<br>-	-<br>-<br>-	-<br>-<br>-	-<br>-<br>-
	**Adjusted for: Parity**<br>Yes<br>No	A(a)	*1*<br>*1*<br>-	*0.950(0.782,1.154)*<br>*0.950(0.780,1.150)*<br>-	-	-	-	0<br>0<br>0	-<br>-<br>-	-<br>-<br>-	-<br>-<br>-	-<br>-<br>-
		A(b)	*1*<br>*1*<br>-	*0.950(0.782,1.154)*<br>*0.950(0.780,1.150)*<br>-	-	-	-	0<br>0<br>0	-<br>-<br>-	-<br>-<br>-	-<br>-<br>-	-<br>-<br>-
	**Timing Category**	A(a)	*1*<br>*1*<br>-	*1.000(0.537,1.864)*<br>*1.000(0.499,1.735)*<br>-	-	-	-	0<br>0<br>0	-<br>-<br>-	-<br>-<br>-	-<br>-<br>-	-<br>-<br>-

			-	-				0	-	-	-	-
			-	-				0	-	-	-	-
	First trimester	B	*1*	*0.500(0.189,1.323)*	-	-	-	0	-	-	-	-
	Second trimester		*1*	*0.500(0.200,1.400)*				0	-	-	-	-
	Third trimester		-	-				0	-	-	-	-
	Cord blood		-	-				0	-	-	-	-
			-	-				0	-	-	-	-
	**Study design**	A(a)	*1*	*1.000(0.537,1.864)*	-	-	-	0	-	-	-	-
	Cross-sectional		-	-				0	-	-	-	-
	Case-control		-	-				0	-	-	-	-
	Cohort		*1*	*1.000(0.499,1.735)*				0	-	-	-	-
		B	*1*	*0.500(0.189,1.323)*	-	-	-	0	-	-	-	-
			-	-				0	-	-	-	-
			-	-				0	-	-	-	-
			1	*0.500(0.200,1.400)*				0	-	-	-	-
	**Setting**	A(a)	*1*	*1.000(0.537,1.864)*	-	-	-	0	-	-	-	-
	Europe		*1*	*1.000(0.499,1.735)*				0	-	-	-	-
	Asia		-	-				0	-	-	-	-
	North America		-	-				0	-	-	-	-
	Other		-	-				0	-	-	-	-
		B	*1*	*0.500(0.189,1.323)*	-	-	-	0	-	-	-	-
			1	*0.500(0.200,1.400)*				0	-	-	-	-
			-	-				0	-	-	-	-
			-	-				0	-	-	-	-
			-	-				0	-	-	-	-

	**Matrix**<br>Serum<br>Plasma<br>Whole blood	A(a)	*1*<br>*1*<br>-<br>-	*1.000(0.537,1.864)*<br>*1.000(0.499,1.735)*<br>-<br>-	-	-	-	0<br>0<br>0<br>0	-<br>-<br>-<br>-	-<br>-<br>-<br>-	-<br>-<br>-<br>-	-<br>-<br>-<br>-
		B	*1*<br>*1*<br>-<br>-	*0.500(0.189,1.323)*<br>*0.500(0.200,1.400)*<br>-<br>-	-	-	-	0<br>0<br>0<br>0	-<br>-<br>-<br>-	-<br>-<br>-<br>-	-<br>-<br>-<br>-	-<br>-<br>-<br>-
	**Adjusted for: GA**<br>Yes<br>No	A(a)	*1*<br>-<br>*1*	*1.000(0.537,1.864)*<br>-<br>*1.000(0.499,1.735)*	-	-	-	0<br>0<br>0	-<br>-<br>-	-<br>-<br>-	-<br>-<br>-	-<br>-<br>-
		B	*1*<br>-<br>*1*	*0.500(0.189,1.323)*<br>-<br>*0.500(0.200,1.400)*	-	-	-	0<br>0<br>0	-<br>-<br>-	-<br>-<br>-	-<br>-<br>-	-<br>-<br>-
	**Adjusted for: Parity**<br>Yes<br>No	A(a)	*1*<br>*1*<br>-	*1.000(0.537,1.864)*<br>*1.000(0.499,1.735)*<br>-	-	-	-	0<br>0<br>0	-<br>-<br>-	-<br>-<br>-	-<br>-<br>-	-<br>-<br>-
		A(b)	*1*<br>*1*<br>-	*0.500(0.189,1.323)*<br>*0.500(0.200,1.400)*<br>-	-	-	-	0<br>0<br>0	-<br>-<br>-	-<br>-<br>-	-<br>-<br>-	-<br>-<br>-
		B	2<br>*1*<br>*1*	1.099(0.465,2.598)<br>*0.730(0.380,1.410)*<br>*1.760(0.790,3.900)*	0.095<br>-<br>-	64.1%<br>-<br>-	0.248<br>-<br>-	0<br>0<br>0	-<br>-<br>-	-<br>-<br>-	-<br>-<br>-	-<br>-<br>-

Birth Outcome indicators	Study characteristics		N	Summary β or OR (95% CI)	P value	I-squared组	Tau-squared	N	Summary β or OR (95% CI)	P value	I-squared	Tau-squared
			PFDA					PFDeA				
**BW/g**	**Gender**<br>Male<br>Female	A(a)	8<br>4<br>4	-0.285(-0.978,0.409)<br>-14.854(-43.548,13.841)<br>-30.877(-75.827,14.072)	0.097<br>0.254<br>0.094	42.2%<br>26.2%<br>53.0%	0.210<br>308.708<br>1.57.5947	0	-	-	-	-
		A(b)	2<br>*1*<br>*1*	66.101(-44.979,177.181)<br>*105.000(-35.000,245.000)*<br>*0.000(-180.000,185.000)*	0.371<br>-<br>-	0.0%<br>-<br>-	0.000<br>-<br>-	0	-	-	-	-
		B	6<br>**3**<br>3	24.406(-84.076,132.888)<br>**-89.800(-155.330,-24.270)**<br>-33.092(-11.751,45.566)	0.045<br>**0.124**<br>0.388	62.8%<br>**57.6%**<br>0.0%	4032.625<br>**3550.391**<br>0.000	0	-	-	-	-
	**Timing Category**<br>First trimester<br>Second trimester<br>Third trimester<br>Cord blood	A(a)	10<br>2<br>*1*<br>*1*<br>6	-14.330(-35.845,7.184)<br>-8.667(-50.155,32.821)<br>*29.700(-66.600,126.000)*<br>*7.800(-86.400,102.000)*<br>-32.548(-71.479,6.384)	0.507<br>0.739<br>-<br>-<br>0.239	0.0%<br>0.0%<br>-<br>-<br>26.0%	0.000<br>0.000<br>*0.000*<br>*0.000*<br>595.8	0	-	-	-	-
		A(b)	*1*<br>-<br>*1*<br>-	*50.000(-35.000,135.000)*<br>-	-	-	-	0	-	-	-	-

			-	*50.000(-40.000,130.000)*<br>-<br>-								
		B	3<br>*1*<br>*1*<br>-<br>*1*	3.097(-37.186,43.380)<br>*-16.000(-80.000,47.900)*<br>*2.000(-55.000,59.000)*<br>-<br>*81.400(-43.600,206.500)*	0.396<br>-<br>-<br>-<br>-	0.0%<br>-<br>-<br>-<br>-	0.000<br>-<br>-<br>-<br>-	0	-	-	-	-
	**Study design**<br>Cross-sectional<br>Case-control<br>Cohort	A(a)	**11**<br>5<br>-<br>6	**-24.252(-38.574,-9.930)**<br>-33.901(-71.624,3.821)<br>-<br>-23.579(-38.749, -8.408)	**0.404**<br>0.198<br>-<br>0.493	**4.0%**<br>33.5%<br>-<br>0.0%	**28.116**<br>600.627<br>-<br>0.000	6<br>-<br>-<br>6	-0.102(-0.609,0.406)<br>-<br>-<br>-0.102(-0.609,0.406)	0.027<br>-<br>-<br>0.027	63.5%<br>-<br>-<br>63.5%	0.117<br>-<br>-<br>0.117
		A(b)	2<br>-<br>-<br>2	22.831(-52.297,97.960)<br>-<br>-<br>22.831(-52.297,97.960)	0.292<br>-<br>-<br>0.292	9.9%<br>-<br>-<br>9.9%	326.650<br>-<br>-<br>326.650	0	-	-	-	-
		B	4<br>-<br>-<br>4	-14.138(-61.706,33.430)<br>-<br>-<br>-14.138(-61.706,33.430)	0.146<br>-<br>-<br>0.146	44.3%<br>-<br>-<br>44.3%	1.0e+03<br>-<br>-<br>1.0e+03	*1*<br>-<br>-<br>*1*	*0.400(-58.350,59.150)*<br>-<br>-<br>*0.400(-58.400,59.100)*	-	-	-

	**Setting**<br>Europe<br>Asia<br>North America<br>Other	A(a)	**11**<br>**4**<br>**7**<br>-<br>-	**-24.252(-38.574,9.930)**<br>**-36.679(-64.186,-**<br>**9.173)**<br>**-21.087(-42.036,-**<br>**0.138)**<br>-<br>-	**0.40**<br>**4**<br>**0.53**<br>**0**<br>**0.31**<br>**5**<br>-<br>-	**4.0%**<br>**0.0%**<br>**15.1%**<br>-<br>-	**28.116**<br>**0.000**<br>**130.768**<br>-<br>-	5<br>-<br>*1*<br>**4**<br>-	-0.102(-0.609,0.406)<br>-<br>*-0.104(-0.637,0.428)*<br>**11.500(37.350,60.350)**<br>-	0.027<br>-<br>*0.013*<br>-<br>-	63.5%<br>-<br>*72.1%*<br>-<br>-	0.117<br>-<br>*0.131*<br>**0.000**<br>-
		A(b)	2<br>2<br>-<br>-<br>-	22.831(-52.297,97.960)<br>22.831(-52.297,97.960)<br>-<br>-<br>-	0.29<br>2<br>0.29<br>2<br>-<br>-<br>-	9.9%<br>9.9%<br>-<br>-<br>-	326.650<br>326.650<br>-<br>-<br>-	0	-	-	-	-
		B	4<br>3<br>*1*<br>-<br>-	-14.138(-61.706,33.430)<br>-26.146(-67.917,15.626)<br>*81.400(-*<br>*43.650,206.450)*<br>-<br>-	0.14<br>6<br>0.24<br>9<br>-<br>-<br>-	44.3%<br>28.1%<br>-<br>-<br>-	1016.918<br>383.641<br>*0.000*<br>-<br>-	*1*<br>-<br>-<br>*1*<br>-	*0.400(-58.350,59.150)*<br>-<br>-<br>*0.400(-58.400,59.100)*<br>-	-	-	-
	**Matrix**<br>Serum<br>Plasma<br>Whole blood	A(a)	**11**<br>**8**<br>**3**<br>-	**-24.252(-38.574,-**<br>**9.930)**<br>**-38.435(-65.583,-**<br>**11.287)**<br>**-17.904(-34.350,-**<br>**1.458)**<br>-	**0.40**<br>**4**<br>**0.26**<br>**3**<br>**0.84**<br>**5**<br>-	**4.0%**<br>**20.9%**<br>**0.0%**<br>-	**28.116**<br>**306.971**<br>**0.000**<br>-	5<br>5<br>-<br>-	-0.102(-0.609,0.406)<br>-0.102(-0.609,0.406)<br>-<br>-	0.027<br>0.027<br>-<br>-	63.5%<br>63.5%<br>-<br>-	0.117<br>0.117<br>-<br>-

		A(b)	2<br>2<br>-<br>-	22.831(-52.297,97.960)<br>22.831(-52.297,97.960)<br>-<br>-	0.29<br>2<br>0.29<br>2<br>-<br>-	9.9%<br>9.9%<br>-<br>-	326.650<br>326.650<br>-<br>-	0	-	-	-	-
		B	4<br>*1*<br>3<br>-	-14.138(-61.706,33.430)<br>*-8.958(-80.748,62.831)*<br>-16.000(-79.950,47.950)<br>-	0.14<br>6<br>*0.06*<br>*8*<br>-<br>-	44.3%<br>*62.8%*<br>-<br>-	1016.918<br>*2435.597*<br>0.000<br>-	*1*<br>*1*<br>-<br>-	*0.400(-58.350,59.150)*<br>*0.400(-58.400,59.100)*<br>-<br>-	-	-	-
		A(a)	**11**<br>**9**<br>2	**-24.252(-38.574,-**<br>**9.930)**<br>**-26.634(-45.392,-**<br>**7.875)**<br>-22.898(-63.281,17.485)	**0.40**<br>**4**<br>**0.29**<br>**5**<br>0.36<br>4	**4.0%**<br>**16.6%**<br>0.0%	**28.116**<br>**134.071**<br>0.000	5<br>*1*<br>4	-0.102(-0.609,0.406)<br>*11.500(-37.350,60.350)*<br>-0.104(-0.637,0.428)	0.027<br>-<br>0.013	63.5%<br>-<br>72.1%	0.117<br>*0.000*<br>0.131
	**Adjusted for: GA**<br>Yes<br>No	A(b)	2<br>2<br>-	22.831(-52.297,97.960)<br>22.831(-52.297,97.960)<br>-	0.29<br>2<br>0.29<br>2<br>-	9.9%<br>9.9%<br>-	326.650<br>326.650<br>-	0	-	-	-	-
		B	4<br>2<br>2	-14.138(-61.706,33.430)<br>-32.064(-<br>101.586,37.458)<br>17.268(-<br>73.263,107.800)	0.14<br>6<br>0.10<br>3<br>0.17<br>4	44.3%<br>62.4%<br>45.9%	1016.918<br>1572.780<br>2175.721	*1*<br>*1*<br>-	*0.400(-58.350,59.150)*<br>*0.400(-58.400,59.100)*<br>-	-	-	-

	**Adjusted for:Parity**<br>Yes<br>No	A(a)	**11**<br>**10**<br>*1*	**-24.252(-38.574,-9.930)**<br>**-24.664(-40.112,-9.216)**<br>*-120.000(-385.000,145.000)*	**0.404**<br>**0.358**<br>-	**4.0%**<br>**9.2%**<br>-	**28.116**<br>**61.172**<br>*0.000*	5<br>*1*<br>4	-0.102(-0.609,0.406)<br>*11.500(-37.350,60.350)*<br>-0.104(-0.637,0.428)	0.027<br>-<br>0.013	63.5%<br>-<br>72.1%	0.117<br>*0.000*<br>0.131
		A(b)	2<br>-<br>2	22.831(-52.297,97.960)<br>-<br>22.831(-52.297,97.960)	0.292<br>-<br>0.292	9.9%<br>-<br>9.9%	326.650<br>-<br>326.650	0	-	-	-	-
		B	4<br>3<br>*1*	-14.138(-61.706,33.430)<br>-17.299(-86.827,52.229)<br>*2.000(-55.000,59.000)*	0.146<br>0.096<br>-	44.3%<br>57.2%<br>-	1016.918<br>2103.954<br>*0.000*	*1*<br>*1*<br>-	*0.400(-58.350,59.150)*<br>*0.400(-58.400,59.100)*<br>-	-	-	-
**Length/ cm**	**Gender**<br>Male<br>Female	A(a)	0	-	-	-	-	2<br>*1*<br>*1*	-0.224(-0.708,0.260)<br>*-0.470(-1.230,0.300)*<br>*-0.060(-0.680,0.570)*	0.416<br>-<br>-	0.0%<br>-<br>-	0.000<br>*0.000*<br>*0.000*
	**Study de**<br>Cross-sectional<br>Case-control<br>Cohort	A(a)	5<br>2<br>-<br>3	-0.034(-0.110,0.041)<br>0.004(-0.089,0.098)<br>-<br>-0.084(-0.221,0.054)	0.331<br>0.377<br>-<br>0.246	13.1%<br>0.0%<br>-<br>28.8%	0.001<br>0.000<br>-<br>0.005	2<br>2<br>-<br>-	-0.224(-0.708,0.260)<br>-0.224(-0.708,0.260)<br>-<br>-	0.416<br>0.416<br>-<br>-	0.0%<br>0.0%<br>-<br>-	0.000<br>0.000<br>-<br>-
		A(b)	*1*<br>-<br>-<br>*1*	*0.000(-0.625,0.625)*<br>-<br>-<br>*0.000(-0.625,0.625)*	-	-	-	0	-	-	-	-

		B	*1*<br>-<br>-<br>*1*	*0.210(-0.140,0.560)*<br>-<br>-<br>*0.210(-0.140,0.560)*	-	-	-	0	-	-	-	-
	**Setting**<br>Europe<br>Asia<br>North America<br>Other	A(a)	5<br>*1*<br>3<br>-<br>*1*	-0.034(-0.110,0.041)<br>*-0.014(-0.353,0.326)*<br>-0.048(-0.146,0.050)<br>-<br>*0.360(-0.435,1.155)*	0.33<br>1<br>-<br>0.16<br>1<br>-<br>-	13.1%<br>-<br>45.3%<br>-<br>-	0.001<br>*0.000*<br>0.003<br>-<br>*0.000*	2<br>-<br>2<br>-<br>-	-0.224(-0.708,0.260)<br>-<br>-0.224(-0.708,0.260)<br>-<br>-	0.416<br>-<br>0.416<br>-<br>-	0.0%<br>-<br>0.0%<br>-<br>-	0.000<br>-<br>0.000<br>-<br>-
		A(b)	*1*<br>*1*<br>-<br>-<br>-	*0.000(-0.625,0.625)*<br>*0.000(-0.625,0.625)*<br>-<br>-<br>-	-	-	-	0	-	-	-	-
		B	*1*<br>-<br>*1*<br>-<br>-	*0.210(-0.140,0.560)*<br>-<br>*0.210(-0.140,0.560)*<br>-<br>-	-	-	-	0	-	-	-	-
	**Matrix**<br>Serum<br>Plasma<br>Whole blood	A(a)	5<br>2<br>2<br>*1*	-0.034(-0.110,0.041)<br>-0.001(-0.092,0.089)<br>-0.121(-0.341,0.099)<br>*0.360(-0.435,1.155)*	0.33<br>1<br>0.94<br>2<br>0.09<br>9<br>-	13.1%<br>0.0%<br>63.3%<br>-	0.001<br>0.000<br>0017<br>*0.000*	2<br>2<br>-<br>-	-0.224(-0.708,0.260)<br>-0.224(-0.708,0.260)<br>-<br>-	0.416<br>0.416<br>-<br>-	0.0%<br>0.0%<br>-<br>-	0.000<br>0.000<br>-<br>-

		A(b)	*1*<br>*1*<br>-<br>-	*0.000(-0.625,0.625)*<br>*0.000(-0.625,0.625)*<br>-<br>-	-	-	-	0	-	-	-	-
		B	*1*<br>*1*<br>-<br>-	*0.210(-0.140,0.560)*<br>*0.210(-0.140,0.560)*<br>-<br>-	-	-	-	0	-	-	-	-
	**Adjusted for: GA**<br>Yes<br>No	A(a)	5<br>4<br>*1*	-0.034(-0.110,0.041)<br>-0.042(-0.138,0.054)<br>*-0.014(-0.353,0.326)*	0.33<br>1<br>0.20<br>4<br>-	13.1%<br>34.7%<br>--	0.001<br>0.003<br>*0.000*	2<br>2<br>-	-0.224(-0.708,0.260)<br>-0.224(-0.708,0.260)<br>-	0.416<br>0.416<br>-	0.0%<br>0.0%<br>-	0.000<br>0.000<br>-
		A(b)	*1*<br>*1*<br>-	*0.000(-0.625,0.625)*<br>*0.000(-0.625,0.625)*<br>-	-	-	-	0	-	-	-	-
		B	*1*<br>-<br>*1*	*0.210(-0.140,0.560)*<br>-<br>*0.210(-0.140,0.560)*	-	-	-	0	-	-	-	-
	**Adjusted for:Parity**<br>Yes<br>No	A(a)	5<br>4<br>*1*	-0.034(-0.110,0.041)<br>-0.039(-0.116,0.039)<br>*0.360(-0.435,1.155)*	0.33<br>1<br>0.30<br>0<br>-	13.1%<br>18.2%<br>-	0.001<br>0,001<br>-	2<br>2<br>-	-0.224(-0.708,0.260)<br>-0.224(-0.708,0.260)<br>-	0.416<br>0.416<br>-	0.0%<br>0.0%<br>-	0.000<br>0.000<br>-
		A(b)	*1*<br>-<br>*1*	*0.000(-0.625,0.625)*<br>-<br>*0.000(-0.625,0.625)*	-	-	-	0	-	-	-	-

		B	*1*<br>*1*<br>-	*0.210(-0.140,0.560)*<br>*0.210(-0.140,0.560)*<br>-	-	-	-	0	-	-	-	-
HC (cm)	**Gender**<br>Male<br>Female	A(a)	2<br>*1*<br>*1*	0.194(-0.386,0.774)<br>*0.491(0.136,0.847)*<br>*-0.101(-0.450,0.249)*	0.020<br>-<br>-	81.5%<br>-<br>-	0.142<br>-<br>-	2<br>*1*<br>*1*	-0.211(-0.498,0.076)<br>*-0.120(-0.480,0.240)*<br>*-0.370(-0.850,0.100)*	0.411<br>-<br>-	0.0%<br>-<br>-	0.000<br>-<br>-
HC (cm)	**Timing Category**<br>First trimester<br>Second trimester<br>Third trimester<br>Cord blood	A(a)	4<br>*1*<br>*1*<br>*1*<br>*1*	3.069(-24.570,30.708)<br>*-0.700(-55.200,53.800)*<br>*12.400(-45.500,70.200)*<br>*-5.400(-61.400,50.600)*<br>*6.400(-46.700,59.500)*	0.974<br>-<br>-<br>-<br>-	0.0%<br>-<br>-<br>-<br>-	0.000<br>-<br>-<br>-<br>-	0	-	-	-	-
HC (cm)	**Study design**<br>Cross-sectional<br>Case-control<br>Cohort	A(a)	4<br>*1*<br>-<br>3	-0.043(-0.122,0.037)<br>*-0.070(-0.670,0.530)*<br>-<br>-0.009(-0.155,0.136)	0.450<br>-<br>-<br>0.268	0.0%<br>-<br>-<br>24.2%	0.000<br>*0.000*<br>-<br>0.005	2<br>-<br>-<br>2	-0.211(-0.498,0.076)<br>-<br>-<br>-0.211(-0.498,0.076)	0.411<br>-<br>-<br>0.411	0.0%<br>-<br>-<br>0.0%	0.000<br>-<br>-<br>0.000
HC (cm)		A(b)	*1*<br>-<br>-<br>*1*	*0.000(-0.625,0.625)*<br>-<br>-<br>*0.000(-0.625,0.625)*	-	-	-	0	-	-	-	-
HC (cm)	**Setting**<br>Europe<br>Asia<br>North America<br>Other	A(a)	4<br>*1*<br>2<br>-<br>*1*	-0.043(-0.122,0.037)<br>*0.150(-0.095,0.394)*<br>-0.065(-0.150,0.020)<br>-<br>*-0.070(-0.670,0.530)*	0.450<br>-<br>0.982<br>-<br>-	0.0%<br>-<br>0.0%<br>-<br>-	0.000<br>*0.000*<br>0.000<br>-<br>*0.000*	2<br>-<br>2<br>-<br>-	-0.211(-0.498,0.076)<br>-<br>-0.211(-0.498,0.076)<br>-<br>-	0.411<br>-<br>0.411<br>-<br>-	0.0%<br>-<br>0.0%<br>-<br>-	0.000<br>-<br>0.000<br>-<br>-

		A(b)	*1*<br>*1*<br>-<br>-<br>-	*0.000(-0.625,0.625)*<br>*0.000(-0.625,0.625)*<br>-<br>-<br>-	-	-	-	0	-	-	-	-
	**Matrix**<br>Serum<br>Plasma<br>Whole blood	A(a)	4<br>*1*<br>2<br>*1*	-0.043(-0.122,0.037)<br>*0.150(-0.095,0.394)*<br>-0.065(-0.150,0.020)<br>*-0.070(-0.670,0.530)*	0.45<br>0<br>-<br>0.98<br>2<br>-	0.0%<br>-<br>0.0%<br>-	0.000<br>*0.000*<br>0.000<br>*0.000*	2<br>2<br>-<br>-	-0.211(-0.498,0.076)<br>-0.211(-0.498,0.076)<br>-<br>-	0.411<br>0.411<br>-<br>-	0.0%<br>0.0%<br>-<br>-	0.000<br>0.000<br>-<br>-
		A(b)	*1*<br>*1*<br>-<br>-	*0.000(-0.625,0.625)*<br>*0.000(-0.625,0.625)*<br>-<br>-	-	-	-	0	-	-	-	-
	**Adjusted for: GA**<br>Yes<br>No	A(a)	4<br>3<br>*1*	-0.043(-0.122,0.037)<br>-0.065(-0.149,0.019)<br>*0.150(-0.095,0.394)*	0.45<br>0<br>1.00<br>0<br>-	0.0%<br>0.0%<br>-	0.000<br>0.000<br>*0.000*	2<br>-<br>2	-0.211(-0.498,0.076)<br>-<br>-0.211(-0.498,0.076)	0.411<br>-<br>0.411	0.0%<br>-<br>0.0%	0.000<br>-<br>0.000
		A(b)	*1*<br>*1*<br>-	*0.000(-0.625,0.625)*<br>*0.000(-0.625,0.625)*<br>-	-	-	-	0	-	-	-	-
	**Adjusted for: Parity**<br>Yes<br>No	A(a)	4<br>3<br>*1*	-0.043(-0.122,0.037)<br>-0.009(-0.155,0.136)<br>*-0.070(-0.670,0.530)*	0.45<br>0<br>0.26<br>8<br>-	0.0%<br>24.2%<br>-	0.293<br>0.005<br>*0.000*	2<br>-<br>2	-0.211(-0.498,0.076)<br>-<br>-0.211(-0.498,0.076)	0.411<br>-<br>0.411	0.0%<br>-<br>0.0%	0.000<br>-<br>0.000

		A(b)	*1*<br>-<br>*1*	*0.000(-0.625,0.625)*<br>-<br>*0.000(-0.625,0.625)*	-	-	-	0	-	-	-	-
**Age(weeks)**	**Gender**<br>Male<br>Female	A(a)	2<br>*1*<br>*1*	0.096(-0.096,0.288)<br>*0.100(-0.150,0.350)*<br>*0.090(-0.210,0.390)*	0.960<br>-<br>-	0.0%<br>-<br>-	0.000<br>-<br>-	0	-	-	-	-
	**Timing Category**<br>First trimester<br>Second trimester<br>Third trimester<br>Cord blood	A(a)	3<br>*1*<br>-<br>*1*<br>*1*	0.002(-0.115,0.119)<br>*-0.116(-0.252,0.019)*<br>-<br>*0.040(-0.050,0.130)*<br>*0.100(-0.090,0.290)*	0.100<br>-<br>-<br>-<br>-	56.6%<br>-<br>-<br>-<br>-	0.006<br>-<br>-<br>-<br>-	0	-	-	-	-
		B	*1*<br>*1*<br>-<br>-<br>-	*-0.500(-1.800,0.800)*<br>*-0.500(-1.800,0.800)*<br>-<br>-<br>-	-	-	-	0	-	-	-	-
	**Study design**<br>Cross-sectional<br>Case-control<br>Cohort	A(a)	4<br>2<br>-<br>2	0.006(-0.086,0.098)<br>0.073(-0.076,0.222)<br>-<br>-0.030(-0.182, 0.123)	0.200<br>0.654<br>-<br>0.060	35.4%<br>71.8%<br>-<br>0.0%	0.003<br>0.008<br>-<br>0.000	0	-	-	-	-
		B	*1*<br>-<br>-<br>*1*	*-0.500(-1.800,0.800)*<br>-<br>-<br>*-0.500(-1.800,0.800)*	-	-	-	0	-	-	-	-

	**Setting**<br>Europe<br>Asia<br>North America<br>Other	A(a)	4<br>2<br>2<br>-<br>-	0.006(-0.086,0.098)<br>-0.078(-0.204,0.048)<br>0.051(-0.030,0.132)<br>-<br>-	35.4%<br>7.7%<br>0.0%<br>-<br>-	0.200<br>0.298<br>0.576<br>-<br>-	0.003<br>0.001<br>0.111<br>-<br>-	0	-	-	-	-
		B	*1*<br>*1*<br>-<br>-<br>-	*-0.500(-1.800,0.800)*<br>*-0.500(-1.800,0.800)*<br>-<br>-<br>-	-	-	-	0	-	-	-	-
	**Matrix**<br>Serum<br>Plasma<br>Whole blood	A(a)	*4*<br>*2*<br>*1*<br>*1*	*0.006(-0.086,0.098)*<br>*0.073(-0.076,0.222)*<br>*-0.116(-0.252,0.019)*<br>*0.040(-0.050,0.130)*	0.200<br>0.654<br>-<br>-	35.4%<br>0.0%<br>-<br>-	0.003<br>0.000<br>*0.000*<br>*0.000*	0	-	-	-	-
		B	*1*<br>-<br>*1*<br>-	*-0.500(-1.800,0.800)*<br>-<br>*-0.500(-1.800,0.800)*<br>-	-	-	-	0	-	-	-	-
	**Adjusted for: GA**<br>Yes<br>No	A(a)	4<br>2<br>2	0.006(-0.086,0.098)<br>0.039(-0.046,0.123)<br>-0.019(-0.230,0.192)	0.200<br>0.939<br>0.069	35.4%<br>0.0%<br>69.7%	0.003<br>0.000<br>0.016	0	-	-	-	-
		B	*1*<br>-<br>*1*	*-0.500(-1.800,0.800)*<br>-<br>*-0.500(-1.800,0.800)*	-	-	-	0	-	-	-	-

	**Adjusted for: Parity**<br>Yes<br>No	A(a)	4<br>4<br>0	0.006(-0.086,0.098)<br>0.006(-0.086,0.098)<br>-	0.20<br>0<br>0.20<br>0<br>-	35.4%<br>35.4%<br>-	0.003<br>0.003<br>-	0	-	-	-	-
		B	*1*<br>*1*<br>-	*-0.500(-1.800,0.800)*<br>*-0.500(-1.800,0.800)*<br>-	-	-	-	0	-	-	-	-
	**Timing Category**<br>First trimester<br>Second trimester<br>Third trimester<br>Cord blood	A(a)	3<br>-<br>-<br>*1*<br>2	-0.000(-0.005,0.004)<br>-<br>-<br>*-0.060(-0.175,0.055)*<br>-0.000(-0.005,0.004)	0.57<br>3<br>-<br>-<br>-<br>0.78<br>5	0.0%<br>-<br>-<br>-<br>0.0%	0.000<br>0.000<br>-<br>-<br>0.000	0	-	-	-	-
		A(b)	*1*<br>-<br>*1*<br>-<br>-	*0.100(-0.600,0.800)*<br>-<br>*0.100(-1.100,0.300)*<br>-<br>-	-	-	-	0	-	-	-	-
		B	2<br>-<br>*1*<br>-<br>*1*	0.027(-0.048,0.102)<br>-<br>*0.020(-0.120,0.150)*<br>-<br>*0.030(-0.060,0.120)*	0.90<br>4<br>-<br>-<br>-<br>-	0.0%<br>-<br>-<br>-<br>-	0.000<br>-<br>-<br>-<br>-	0	-	-	-	-
	**Study design**<br>Cross-sectional<br>Case-control<br>Cohort	A(a)	3<br>2<br>-<br>*1*	-0.000(-0.005,0.004)<br>-0.004(-0.023,0.015)<br>-<br>*0.000(-0.004,0.004)*	0.57<br>3<br>0.33<br>5	0.0%<br>0.0%<br>-<br>-	0.000<br>0.000<br>-<br>-	0	-	-	-	-

					-<br>-							
		A(b)	*1*<br>-<br>-<br>*1*	*0.100(-0.600,0.800)*<br>-<br>-<br>*0.100(-1.100,0.300)*	-	-	-	0	-	-	-	-
		B	2<br>-<br>-<br>2	0.027(-0.048,0.102)<br>-<br>-<br>0.027(-0.048,0.102)	0.90<br>4<br>-<br>-<br>0.90<br>4	0.0%<br>-<br>-<br>0.0%	0.000<br>-<br>-<br>0.000	0	-	-	-	-
	**Setting**<br>Europe<br>Asia<br>North America<br>Other	A(a)	3<br>-<br>2<br>-<br>*1*	-0.000(-0.005,0.004)<br>-<br>-0.000(-0.005,0.004)<br>-<br>*-0.060(-0.175,0.055)*	0.57<br>3<br>-<br>0.78<br>5<br>-<br>-	0.0%<br>-<br>0.0%<br>-<br>-	0.000<br>-<br>0.000<br>-<br>-	0	-	-	-	-
		A(b)	*1*<br>*1*<br>-<br>-<br>-	*0.100(-0.600,0.800)*<br>*0.100(-1.100,0.300)*<br>-<br>-<br>-	-	-	-	0	-	-	-	-
		B	2<br>*1*<br>*1*<br>-<br>-	0.027(-0.048,0.102)<br>*0.020(-0.120,0.150)*<br>*0.030(-0.060,0.120)*<br>-<br>-	0.90<br>4<br>-<br>-<br>-	0.0%<br>-<br>-<br>-<br>-	0.000<br>-<br>-<br>-<br>-	0	-	-	-	-

					-								
	**Matrix**<br>Serum<br>Plasma<br>Whole blood	A(a)	3<br>2<br>-<br>*1*	-0.000(-0.005,0.004)<br>-0.000(-0.005,0.004)<br>-<br>*-0.060(-0.175,0.055)*	0.57<br>3<br>0.78<br>5<br>-<br>-	0.0%<br>0.0%<br>-<br>-	0.000<br>0.000<br>-<br>-	0	-	-	-	-
		A(b)	*1*<br>*1*<br>-<br>-	*0.100(-0.600,0.800)*<br>*0.100(-1.100,0.300)*<br>-<br>-	-	-	-	0	-	-	-	-
		B	2<br>2<br>-	0.027(-0.048,0.102)<br>0.027(-0.048,0.102)<br>-	0.90<br>4<br>-<br>-	0.0%<br>-<br>-	0.000<br>-<br>-	0	-	-	-	-
	**Adjusted for: GA**<br>Yes<br>No	A(a)	3<br>3<br>0	-0.000(-0.005,0.004)<br>-0.000(-0.005,0.004)<br>-	0.57<br>3<br>0.57<br>3<br>-	0.0%<br>0.0%<br>-	0.000<br>-<br>-	0	-	-	-	-
		A(b)	*1*<br>*1*<br>-	*0.100(-0.600,0.800)*<br>*0.100(-1.100,0.300)*<br>-	-	-	-	0	-	-	-	-
		B	2<br>*1*<br>*1*	0.027(-0.048,0.102)<br>*0.020(-0.120,0.150)*<br>*0.030(-0.060,0.120)*	0.90<br>4<br>-<br>-	0.0%<br>-<br>-	0.000<br>-<br>-	0	-	-	-	-

	**Adjusted for:**<br>**Parity**<br>Yes<br>No	A(a)	3<br>2<br>*1*	-0.000(-0.005,0.004)<br>-0.000(-0.005,0.004)<br>*-0.060(-0.175,0.055)*	0.573<br>0.785<br>-	0.0%<br>0.0%<br>-	0.000<br>0.000<br>*0.000*	0	-	-	-	-
		A(b)	*1*<br>*1*<br>-	*0.100(-0.600,0.800)*<br>*0.100(-1.100,0.300)*<br>-	-	-	-	0	-	-	-	-
		B	2<br>*1*<br>*1*	0.027(-0.048,0.102)<br>*0.020(-0.120,0.150)*<br>*0.030(-0.060,0.120)*	0.904<br>-<br>-	0.0%<br>-<br>-	0.000<br>-<br>-	0	-	-	-	-
**PTB**	**Gender**<br>Male<br>Female	A(a)	2<br>*1*<br>*1*	0.874(0.658,1.162)<br>*0.870(0.600,1.280)*<br>*0.880(0.570,1.350)*	0.969<br>-<br>-	0.0%<br>-<br>-	0.000<br>-<br>-	0	-	-	-	-
	**Timing Category**<br>First trimester<br>Second trimester<br>Third trimester<br>Cord blood	A(a)	*1*<br>-<br>-<br>*1*<br>-	*0.880(0.661,1.172)*<br>-<br>-<br>*0.880(0.660,1.170)*<br>-	-	-	-	0	-	-	-	-
	**Study design**<br>Cross-sectional<br>Case-control<br>Cohort	A(a)	2<br>-<br>-<br>2	1.311(0.572,3.009)<br>-<br>-<br>1.311(0.572,3.009)	0.004<br>-<br>-<br>0.004	88.1%<br>-<br>-<br>88.1%	0.317<br>-<br>-<br>0.317	0	-	-	-	-
	**Setting**<br>Europe<br>Asia	A(a)	2<br>***1***<br>*1*	1.311(0.572,3.009)<br>***2.057(1.281,3.474)***<br>*0.880(0.660,1.170)*	0.004<br>-	88.1%<br>-<br>-	0.317<br>-<br>-	0	-	-	-	-

	North America<br>Other		-<br>-	-<br>-	-<br>-<br>-	-<br>-	-<br>-					
	**Matrix**<br>Serum<br>Plasma<br>Whole blood	A(a)	2<br>*1*<br>***1***	1.311(0.572,3.009)<br>*0.880(0.660,1.170)*<br>***2.057(1.281,3.474)***	0.00<br>4<br>-<br>-	88.1%<br>-<br>-	0.317<br>-<br>-	0	-	-	-	-
	**Adjusted for: GA**<br>Yes<br>No	A(a)	2<br>*1*<br>***1***	1.311(0.572,3.009)<br>*0.880(0.660,1.170)*<br>***2.057(1.281,3.474)***	0.00<br>4<br>-<br>-	88.1%<br>-<br>-	0.317<br>-<br>-	0	-	-	-	-
	**Adjusted for:Parity**<br>Yes<br>No	A(a)	2<br>*1*<br>***1***	1.311(0.572,3.009)<br>*0.880(0.660,1.170)*<br>***2.057(1.281,3.474)***	0.00<br>4<br>-<br>-	88.1%<br>-<br>-	0.317<br>-<br>-	0	-	-	-	-
**SGA**	**Gender**<br>Male<br>Female	A(a)	**2**<br>*1*<br>*1*	**1.461(1.060,2.012)**<br>*1.360(0.900,2.070)*<br>*1.620(0.980,2.670)*	**0.59**<br>**9**<br>-<br>-	**0.0%**<br>-<br>-	**0.000**<br>-<br>-	2<br>*1*<br>***1***	1.420(0.332,6.076)<br>*0.710(0.330,1.520)*<br>***3.140(1.070,9.190)***	0.027<br>-<br>-	79.5%<br>-<br>-	0.878<br>-<br>-
		B	**2**<br>*1*<br>*1*	**1.461(1.060,2.012)**<br>*1.360(0.900,2.070)*<br>*1.620(0.980,2.670)*	**0.59**<br>**9**<br>-<br>-	**0.0%**<br>-<br>-	**0.000**<br>-<br>-	0	-	-	-	-
	**Study design**<br>Cross-sectional<br>Case-control<br>Cohort	A(a)	***1***<br>-<br>-<br>***1***	***1.460(1.060,2.010)***<br>-<br>-<br>***1.460(1.060,2.010)***	-	-	-	2<br>2<br>-<br>-	1.420(0.332,6.076)<br>1.420(0.332,6.076)<br>-<br>-	0.027<br>0.027<br>-<br>-	79.5%<br>79.5%<br>-<br>-	0.878<br>0.878<br>-<br>-
		B	*1*<br>-<br>-	*1.500(0.943,2.387)*<br>-<br>-	-	-	-	0	-	-	-	-

			1	*1.500(0.940,2.380)*								
	**Setting**<br>Europe<br>Asia<br>North America<br>Other	A(a)	***1***<br>***1***<br>-<br>-<br>-	***1.460(1.060,2.010)***<br>***1.460(1.060,2.010)***<br>-<br>-<br>-	-	-	-	2<br>-<br>2<br>-<br>-	1.420(0.332,6.076)<br>-<br>1.420(0.332,6.076)<br>-<br>-	0.027<br>-<br>0.027<br>-<br>-	79.5%<br>-<br>79.5%<br>-<br>-	0.878<br>-<br>0.878<br>-<br>-
		B	*1*<br>*1*<br>-<br>-<br>-	*1.500(0.943,2.387)*<br>*1.500(0.940,2.380)*<br>-<br>-<br>-	-	-	-	0	-	-	-	-
	**Matrix**<br>Serum<br>Plasma<br>Whole blood	A(a)	***1***<br>***1***<br>-<br>-	***1.460(1.060,2.010)***<br>***1.460(1.060,2.010)***<br>-<br>-	-	-	-	2<br>2<br>-<br>-	1.420(0.332,6.076)<br>1.420(0.332,6.076)<br>-<br>-	0.027<br>0.027<br>-<br>-	79.5%<br>79.5%<br>-<br>-	0.878<br>0.878<br>-<br>-
		B	*1*<br>*1*<br>-<br>-	*1.500(0.943,2.387)*<br>*1.500(0.940,2.380)*<br>-<br>-	-	-	-	0	-	-	-	-
	**Adjusted for: GA**<br>Yes<br>No	A(a)	***1***<br>***1***<br>-	***1.460(1.060,2.010)***<br>***1.460(1.060,2.010)***<br>-	-	-	-	2<br>2<br>-	1.420(0.332,6.076)<br>1.420(0.332,6.076)<br>-	0.027<br>0.027<br>-	79.5%<br>79.5%<br>-	0.878<br>0.878<br>-
		B	*1*<br>*1*<br>-	*1.500(0.943,2.387)*<br>*1.500(0.940,2.380)*<br>-	-	-	-	0	-	-	-	-
	**Adjusted for: Parity**<br>Yes	A(a)	***1***<br>***1***<br>-	***1.460(1.060,2.010)***<br>***1.460(1.060,2.010)***<br>-	-	-	-	2<br>2<br>-	1.420(0.332,6.076)<br>1.420(0.332,6.076)<br>-	0.027<br>0.027<br>-	79.5%<br>79.5%<br>-	0.878<br>0.878<br>-

	No	B	*1*<br>*1*<br>-	*1.500(0.943,2.387)*<br>*1.500(0.940,2.380)*<br>-	-	-	-	0	-	-	-	-
	**Timing Category**<br>First trimester<br>Second trimester<br>Third trimester<br>Cord blood	A(a)	*1*<br>*1*<br>-<br>-<br>-	*1.281(0.712,2.306)*<br>*1.281(0.738,2.393)*<br>-<br>-<br>-	-	-	-	0	-	-	-	-
		B	*1*<br>*1*<br>-<br>-<br>-	*0.900(0.402,2.012)*<br>*0.900(0.400,2.000)*<br>-<br>-<br>-	-	-	-	0	-	-	-	-
	**Study design**<br>Cross-sectional<br>Case-control<br>Cohort	A(a)	*1*<br>-<br>-<br>*1*	*1.281(0.712,2.306)*<br>-<br>-<br>*1.281(0.738,2.393)*	-	-	-	0	-	-	-	-
		B	*1*<br>-<br>-<br>*1*	*0.900(0.402,2.012)*<br>-<br>-<br>*0.900(0.400,2.000)*	-	-	-	0	-	-	-	-
	**Setting**<br>Europe<br>Asia<br>North America<br>Other	A(a)	*1*<br>*1*<br>-<br>-<br>-	*1.281(0.712,2.306)*<br>*1.281(0.738,2.393)*<br>-<br>-<br>-	-	-	-	0	-	-	-	-
		B	*1*<br>*1*<br>-<br>-	*0.900(0.402,2.012)*<br>*0.900(0.400,2.000)*<br>-<br>-	-	-	-	0	-	-	-	-

			-	-								
	**Matrix**<br>Serum<br>Plasma<br>Whole blood	A(a)	*1*<br>-<br>*1*<br>-	*1.281(0.712,2.306)*<br>-<br>*1.281(0.738,2.393)*<br>-	-	-	-	0	-	-	-	-
		B	*1*<br>-<br>*1*<br>-	*0.900(0.402,2.012)*<br>-<br>*0.900(0.400,2.000)*<br>-	-	-	-	0	-	-	-	-
	**Adjusted for: GA**<br>Yes<br>No	A(a)	*1*<br>-<br>*1*	*1.281(0.712,2.306)*<br>-<br>*1.281(0.738,2.393)*	-	-	-	0	-	-	-	-
		B	*1*<br>-<br>*1*	*0.900(0.402,2.012)*<br>-<br>*0.900(0.400,2.000)*	-	-	-	0	-	-	-	-
	**Adjusted for: Parity**<br>Yes<br>No	A(a)	*1*<br>*1*<br>-	*1.281(0.712,2.306)*<br>*1.281(0.738,2.393)*<br>-	-	-	-	0	-	-	-	-
		B	*1*<br>*1*<br>-	*0.900(0.402,2.012)*<br>*0.900(0.400,2.000)*<br>-	-	-	-	0	-	-	-	-

Birth Outcome indicators	Study characteristics		N	Summary β or OR (95% CI)	P value	I-squared	Tau-squared	N	Summary β or OR (95% CI)	P value	I-squared	Tau-squared
			PFHpA					PFBS				
BW/g	**Gender**<br>Male<br>Female	A(a)	6<br>3<br>3	0.498(-1.031,2.027)<br>-19.700(-7.203,27.803)<br>0.467(-0.307,1.242)	0.054<br>0.940<br>0.005	53.9%<br>81.3%<br>0.0%	1.044<br>1147.176<br>0	0	-	-	-	-
		B	2<br>*1*<br>*1*	29.778(32.143,91.699)<br>*33.000(-58.000,124.000)*<br>*27.000(-57.000,112.000)*	0.925<br>-<br>-	0.0%<br>-<br>-	0.000<br>-<br>-	0	-	-	-	-
	**Timing Category**<br>First trimester<br>Second trimester<br>Third trimester<br>Cord blood	A(a)	10<br>2<br>*1*<br>*1*<br>6	-14.330(-35.845,7.184)<br>-8.667(-50.155,32.821)<br>*29.700(-66.600,126.000)*<br>*7.800(-86.400,102.000)*<br>-32.548(-71.479,6.384)	0.507<br>0.739<br>-<br>-<br>0.239	0.0%<br>0.0%<br>-<br>-<br>26.0%	0.000<br>0.000<br>*0.000*<br>*0.000*<br>595.857	0	-	-	-	-
	**Study design**<br>Cross-sectional<br>Case-control<br>Cohort	A(a)	4<br>*1*<br>-<br>3	-20.896(-116.043,74.251)<br>*14.716(-115.963,145.395)*<br>-<br>-103.700(-211.250,3.850)	0.063<br>-<br>-<br>0.144	59.0<br>-<br>-<br>48.3%%	4817.536<br>-<br>-<br>6951.374	*1*<br>-<br>-<br>*1*	*21.600(-44.400,87.600)*<br>-<br>-<br>*21.600(-44.400,87.600)*	-	-	-
		B	*1*<br>-<br>-<br>*1*	*31.000(-31.000,93.000)*<br>-<br>-	-	-	-	0	-	-	-	-

				31.000(-31.000,93.000)								
	**Setting**<br><br>Europe<br>Asia<br>North America<br>Other	A(a)	4<br>*1*<br>3<br>-<br>-	-20.896(-116.043,74.251)<br>*-1.000(-23.500,21.500)*<br>- -41.443(-243.281,160.394)<br>-	0.063<br>-<br>0.047<br>-<br>-	59.0%<br>-<br>67.2%<br>-<br>-	4817.536<br>-<br>20009.521<br>-<br>-	*1*<br>-<br>*1*<br>-<br>-	*21.600(-44.400,87.600)*<br>-<br>*21.600(-44.400,87.600)*<br>-<br>-	-	-	-
		B	*1*<br>*1*<br>-<br>-<br>-	*31.000(-31.000,93.000)*<br>*31.000(-31.000,93.000)*<br>-<br>-<br>-	-	-	-	0	-	-	-	-

	**Matrix**<br>Serum<br>Plasma<br>Whole blood	A(a)	4<br>3<br>*1*<br>-	-20.896(-116.043,74.251)<br>-52.383(-152.395,47.629)<br>*140.200(-44.550,324.950)*<br>-	0.063<br>0.085<br>-<br>-	59.0%<br>59.5%<br>-<br>-	4817.536<br>4302.607<br>-<br>-	*1*<br>-<br>*1*<br>-	*21.600(-44.400,87.600)*<br>-<br>*21.600(-44.400,87.600)*<br>-	-	-	-
		B	*1*<br>*1*<br>-<br>-	*31.000(-31.000,93.000)*<br>*31.000(-31.000,93.000)*<br>-<br>-	-	-	-	0	-	-	-	-
	**Adjusted for: GA**<br>Yes<br>No	A(a)	4<br>4<br>0	-20.896(-116.043,74.251)<br>-20.896(-116.043,74.251)<br>-	0.063<br>0.063<br>-	59.0%<br>59.0%<br>-	4.8e+03<br>4.8e+03<br>-	*1*<br>*1*<br>-	*21.600(-44.400,87.600)*<br>*21.600(-44.400,87.600)*<br>-	-	-	-
		B	*1*<br>*1*<br>-	*31.000(-31.000,93.000)*<br>*31.000(-31.000,93.000)*<br>-	-	-	-	0	-	-	-	-
	**Adjusted for: Parity**<br>Yes<br>No	A(a)	4<br>4<br>0	-20.896(-116.043,74.251)<br>-20.896(-116.043,74.251)<br>-	0.063<br>0.063<br>-	59.0%<br>59.0%<br>-	4.8e+03<br>4.8e+03<br>-	*1*<br>*1*<br>-	*21.600(-44.400,87.600)*<br>*21.600(-44.400,87.600)*<br>-	-	-	-

		B	*1*<br>*1*<br>-	*31.000(-31.000,93.000)*<br>*31.000(-31.000,93.000)*<br>-	-	-	-	0	-	-	-	-
	**Timing Category**<br>First trimester<br>Second trimester<br>Third trimester<br>Cord blood	A(a)	4<br>*1*<br>*1*<br>*1*<br>*1*	0.005(-0.158,0.168)<br>*0.420(-0.110 ,0.960)*<br>*0.170(-0.440 ,0.790)*<br>*-0.050(-0.240,0.150)*<br>*-0.070(-0.470,0.320)*	0.387<br>-<br>-<br>-<br>-	1.1%<br>-<br>-<br>-<br>-	0.000<br>-<br>-<br>-<br>-	4<br>*1*<br>*1*<br>*1*<br>*1*	0.001(-0.192,0.194)<br>*0.090(-0.100,0.280)*<br>*-0.310(-0.880,0.260)*<br>*-0.390(-1.100,0.320)*<br>*-0.080(-0.850,0.690)*	0.368<br>-<br>-<br>-<br>-	4.9%<br>-<br>-<br>-<br>-	0.003<br>-<br>-<br>-<br>-
	**Study design**<br>Cross-sectional<br>Case-control<br>Cohort	A(a)	*1*<br>*1*<br>-<br>-	*0.420(-0.115,0.955)*<br>*0.420(-0.110,0.960)*<br>-<br>-	-	-	-	*1*<br>*1*<br>-<br>-	*0.090(-0.100,0.280)*<br>*0.090(-0.100,0.280)*<br>-<br>-	-	-	-
	**Setting**<br>Europe<br>Asia<br>North America<br>Other	A(a)	*1*<br>-<br>*1*<br>-<br>-	*0.420(-0.115,0.955)*<br>-<br>*0.420(-0.110,0.960)*<br>-<br>-	-	-	-	*1*<br>-<br>*1*<br>-<br>-	*0.090(-0.100,0.280)*<br>-<br>*0.090(-0.100,0.280)*<br>-<br>-	-	-	-
	**Matrix**<br>Serum<br>Plasma<br>Whole blood	A(a)	*1*<br>-<br>*1*<br>-	*0.420(-0.115,0.955)*<br>-<br>*0.420(-0.110,0.960)*<br>-	-	-	-	*1*<br>-<br>*1*<br>-	*0.090(-0.100,0.280)*<br>-<br>*0.090(-0.100,0.280)*<br>-	-	-	-
	**Adjusted for: GA**<br>Yes<br>No	A(a)	*1*<br>*1*<br>-	*0.420(-0.115,0.955)*<br>*0.420(-0.110,0.960)*<br>-	-	-	-	*1*<br>*1*<br>-	*0.090(-0.100,0.280)*<br>*0.090(-0.100,0.280)*<br>-	-	-	-

	**Adjusted for: Parity**<br>Yes<br>No	A(a)	*1*<br>*1*<br>-	*0.420(-0.115,0.955)*<br>*0.420(-0.110,0.960)*<br>-	-	-	-	*1*<br>*1*<br>-	*0.090(-0.100,0.280)*<br>*0.090(-0.100,0.280)*<br>-	-	-	-
	**Timing Category**<br>First trimester<br>Second trimester<br>Third trimester<br>Cord blood	A(a)	4<br>*1*<br>*1*<br>*1*<br>*1*	-4.441(-34.768,25.886)<br>*-36.900(-144.500,70.600)*<br>*-0.150(-30.800,88.400)*<br>*13.100(-25.900,52.100)*<br>*-62.600(-143.400,18.200)*	0.371<br>-<br>-<br>-<br>-	4.3%<br>-<br>-<br>-<br>-	48.460<br>-<br>-<br>-<br>-	4<br>*1*<br>*1*<br>*1*<br>*1*	5.498(-28.398,39.393)<br>*11.400(-26.100,48.900)*<br>*-9.100(-124.600,106.500)*<br>*-27.200(-169.100,114.800)*<br>*-37.110(-206.700,132.500)*	0.896<br>-<br>-<br>-<br>-	0.0%<br>-<br>-<br>-<br>-	0.000<br>-<br>-<br>-<br>-
	**Study design**<br>Cross-sectional<br>Case-control<br>Cohort	A(a)	*1*<br>-<br>-<br>*1*	*-36.900(-144.450,70.650)*<br>-<br>-<br>*-36.900(-144.450,70.650)*	-	-	-	*1*<br>-<br>-<br>*1*	*11.400(-26.100,48.900)*<br>-<br>-<br>*11.400(-26.100,48.900)*	-	-	-
	**Setting**<br>Europe<br>Asia<br>North America<br>Other	A(a)	*1*<br>-<br>*1*<br>-<br>-	*-36.900(-144.450,70.650)*<br>-<br>*-36.900(-144.450,70.650)*<br>-<br>-	-	-	-	*1*<br>-<br>*1*<br>-<br>-	*11.400(-26.100,48.900)*<br>-<br>*11.400(-26.100,48.900)*<br>-<br>-	-	-	-
	**Matrix**<br>Serum<br>Plasma<br>Whole blood	A(a)	*1*<br>-<br>*1*<br>-	*-36.900(-144.450,70.650)*<br>-	-	-	-	*1*<br>-<br>*1*<br>-	*11.400(-26.100,48.900)*<br>-<br>*11.400(-26.100,48.900)*<br>-	-	-	-

				-36.900(-<br>144.450,70.650)<br>-								
	**Adjusted for: GA**<br><br>Yes<br>No	A(a)	*1*<br>*1*<br>-	*-36.900(-<br>144.450,70.650)*<br>*-36.900(-<br>144.450,70.650)*<br>-	-	-	-	*1*<br>*1*<br>-	*11.400(-26.100,48.900)*<br>*11.400(-26.100,48.900)*<br>-	-	-	-
	**Adjusted for:Parity**<br>Yes<br>No	A(a)	*1*<br>*1*<br>-	*-36.900(-<br>144.450,70.650)*<br>*-36.900(-<br>144.450,70.650)*<br>-	-	-	-	*1*<br>*1*<br>-	*11.400(-26.100,48.900)*<br>*11.400(-26.100,48.900)*<br>-	-	-	-
**Age(weeks)**	**Gender**<br>Male<br>Female	A(a)	*2*<br>***1***<br>*1*	*0.169(-0.467,0.806)*<br>***0.510(0.010,1.010)***<br>*-0.140(-0.560,0.270)*	0.050<br>-<br>-	74.0%<br>-<br>-	0.156<br>-<br>-	0	-	-	-	-
	**Timing Category**<br>First trimester<br>Second trimester<br>Third trimester<br>Cord blood	A(a)	2<br>-<br>-<br>*1*<br>*1*	0.025(-0.039,0.089)<br>-<br>-<br>*0.020(-0.050,0.080)*<br>*0.140(-0.170,0.450)*	0.458<br>-<br>-<br>-<br>-	0.0%<br>-<br>-<br>-<br>-	0.000<br>-<br>-<br>-<br>-	*1*<br>-<br>-<br>*1*<br>-	*0.020(-0.055,0.095)*<br>-<br>-<br>*0.020(-0.050,0.100)*<br>-	-	-	-
	**Study design**<br>Cross-sectional<br>Case-control<br>Cohort	A(a)	2<br>*1*<br>-<br>*1*	0.025(-0.039,0.089)<br>*0.140(-0.170,0.450)*<br>-<br>*0.020(-0.050,0.080)*	0.458<br>-<br>-<br>-	0.0%<br>-<br>-<br>-	0.000<br>-<br>-<br>-	*1*<br>-<br>-<br>*1*	*0.020(-0.055,0.095)*<br>-<br>-<br>*0.020(-0.050,0.100)*	-	-	-
	**Setting**<br>Europe<br>Asia<br>North America	A(a)	2<br>-<br>2<br>-	0.025(-0.039,0.089)<br>-<br>0.025(-0.039,0.089)<br>-	0.458<br>-<br>-<br>-	0.0%<br>-<br>-<br>-	0.000<br>-<br>-<br>-	*1*<br>-<br>*1*<br>-	*0.020(-0.055,0.095)*<br>-<br>*0.020(-0.050,0.100)*<br>-	-	-	-

	Other		-	-	-	-	-	-	-			
	**Matrix**<br>Serum<br>Plasma<br>Whole blood	A(a)	2<br>*1*<br>-<br>*1*	0.025(-0.039,0.089)<br>*0.140(-0.170,0.450)*<br>-<br>*0.020(-0.050,0.080)*	0.458<br>-<br>-<br>-	0.0%<br>-<br>-<br>-	0.000<br>-<br>-<br>-	*1*<br>-<br>-<br>*1*	*0.020(-0.055,0.095)*<br>-<br><br>*0.020(-0.050,0.100)*	-	-	-
	**Adjusted for: GA**<br>Yes<br>No	A(a)	2<br>*1*<br>*1*	0.025(-0.039,0.089)<br>*0.020(-0.050,0.080)*<br>*0.140(-0.170,0.450)*	0.458<br>-<br>-	0.0%<br>-<br>-	0.000<br>-<br>-	*1*<br>*1*<br>-	*0.020(-0.055,0.095)*<br>*0.020(-0.050,0.100)*<br>-	-	-	-
	**Adjusted for: Parity**<br>Yes<br>No	A(a)	2<br>2<br>0	0.025(-0.039,0.089)<br>0.025(-0.039,0.089)<br>-	0.458<br>0.458<br>-	0.0%<br>0.0%<br>-	0.000<br>0.000<br>-	*1*<br>*1*<br>-	*0.020(-0.055,0.095)*<br>*0.020(-0.050,0.100)*<br>-	-	-	-
	**Timing Category**<br>First trimester<br>Second trimester<br>Third trimester<br>Cord blood	A(a)	*1*<br>-<br>-<br>-<br>*1*	*-0.002(-0.005,0.001)*<br>-<br>-<br>-<br>*-0.002(-0.005,0.001)*	-	-	-	0	-	-	-	-
	**Study design**<br>Cross-sectional<br>Case-control<br>Cohort	A(a)	*1*<br>-<br>-<br>*1*	*-0.002(-0.005,0.001)*<br>-<br>-<br>*-0.002(-0.005,0.001)*	-	-	-	0	-	-	-	-
	**Setting**<br>Europe<br>Asia<br>North America	A(a)	*1*<br>-<br>*1*<br>-	*-0.002(-0.005,0.001)*<br>-<br>*-0.002(-0.005,0.001)*<br>-	-	-	-	0	-	-	-	-

	Other		-	-								
	**Matrix**<br>Serum<br>Plasma<br>Whole blood	A(a)	*1*<br>*1*<br>-<br>-	*-0.002(-0.005,0.001)*<br>*-0.002(-0.005,0.001)*<br>-<br>-	-	-	-	0	-	-	-	-
	**Adjusted for: GA**<br>Yes<br>No	A(a)	*1*<br>*1*<br>*0*	*-0.002(-0.005,0.001)*<br>*-0.002(-0.005,0.001)*<br>-	-	-	-	0	-	-	-	-
	**Adjusted for: Parity**<br>Yes<br>No	A(a)	*1*<br>*1*<br>*0*	*-0.002(-0.005,0.001)*<br>*-0.002(-0.005,0.001)*<br>-	-	-	-	0	-	-	-	-
**PTB**	**Gender**<br>Male<br>Female	A(a)	2<br>*1*<br>*1*	0.990(0.794,1.234)<br>*1.080(0.810,1.450)*<br>*0.880(0.570,1.120)*	0.368<br>-<br>-	0.0%<br>-<br>-	0.000<br>-<br>-	2<br>*1*<br>*1*	1.051(0.774,1.427)<br>*1.230(0.860,1.760)*<br>*0.900(0.630,1.280)*	0.224<br>-<br>-	32.3%<br>-<br>-	0.015<br>-<br>-
	**Timing Category**<br>First trimester<br>Second trimester<br>Third trimester<br>Cord blood	A(a)	*1*<br>-<br>-<br>*1*<br>-	*0.950(0.782,1.154)*<br>-<br>-<br>*0.950(0.780,1.150)*	-	-	-	*1*<br>-<br>-<br>*1*<br>-	*1.060(0.825,1.362)*<br>-<br>-<br>*1.060(0.830,1.370)*<br>-	-	-	-
	**Study design**<br>Cross-sectional<br>Case-control<br>Cohort	A(a)	*1*<br>-<br>-<br>*1*	*0.950(0.782,1.154)*<br>-<br>-<br>*0.950(0.780,1.150)*	-	-	-	*1*<br>-<br>-<br>*1*	*1.060(0.825,1.362)*<br>-<br>-<br>*1.060(0.830,1.370)*	-	-	-

	**Setting**<br>Europe<br>Asia<br>North America<br>Other	A(a)	*1*<br>-<br>*1*<br>-<br>-	*0.950(0.782,1.154)*<br>-<br>*0.950(0.780,1.150)*<br>-<br>-	-	-	-	*1*<br>-<br>*1*<br>-<br>-	*1.060(0.825,1.362)*<br>-<br>*1.060(0.830,1.370)*<br>-<br>-	-	-	-
	**Matrix**<br>Serum<br>Plasma<br>Whole blood	A(a)	*1*<br>-<br>-<br>*1*	*0.950(0.782,1.154)*<br>-<br>-<br>*0.950(0.780,1.150)*	-	-	-	*1*<br>-<br>-<br>*1*	*1.060(0.825,1.362)*<br>-<br>-<br>*1.060(0.830,1.370)*	-	-	-
	**Adjusted for: GA**<br>Yes<br>No	A(a)	*1*<br>*1*<br>-	*0.950(0.782,1.154)*<br>*0.950(0.780,1.150)*<br>-	-	-	-	*1*<br>*1*<br>-	*1.060(0.825,1.362)*<br>*1.060(0.830,1.370)*<br>-	-	-	-
	**Adjusted for: Parity**<br>Yes<br>No	A(a)	*1*<br>*1*<br>-	*0.950(0.782,1.154)*<br>*0.950(0.780,1.150)*<br>-	-	-	-	*1*<br>*1*<br>-	*1.060(0.825,1.362)*<br>*1.060(0.830,1.370)*<br>-	-	-	-
**SGA**	**Gender**<br>Male<br>Female	A(a)	2<br>*1*<br>*1*	1.065(0.899,1.262)<br>*1.070(0.860,1.370)*<br>*1.060(0.830,1.360)*	0.957<br>-<br>-	0.0%<br>-<br>-	0.000<br>-<br>-	0	-	-	-	-
		B	2<br>*1*<br>*1*	1.213(0.782,1.882)<br>*1.150(0.650,2.040)*<br>*1.310(0.660,2.600)*	0.775<br>-<br>-	0.0%<br>-<br>-	0.000<br>-<br>-	0	-	-	-	-
	**Study design**<br>Cross-sectional<br>Case-control<br>Cohort	A(a)	*1*<br>-<br>-<br>*1*	*1.060(0.899,1.249)*<br>-<br>-<br>*1.060(0.900,1.250)*	-	-	-	0	-	-	-	-
		B	*1*<br>-<br>-<br>*1*	*1.060(0.899,1.249)*<br>-<br>-<br>*1.060(0.900,1.250)*	-	-	-	0	-	-	-	-

	**Setting**<br>Europe<br>Asia<br>North America<br>Other	A(a)	*1*<br>*1*<br>-<br>-<br>-	*1.060(0.899,1.249)*<br>*1.060(0.900,1.250)*<br>-<br>-	-	-	-	0	-	-	-	-
		B	*1*<br>*1*<br>-<br>-<br>-	*1.060(0.899,1.249)*<br>*1.060(0.900,1.250)*<br>-<br>-	-	-	-	0	-	-	-	-
	**Matrix**<br>Serum<br>Plasma<br>Whole blood	A(a)	*1*<br>*1*<br>-<br>-	*1.060(0.899,1.249)*<br>*1.060(0.900,1.250)*<br>-<br>-	-	-	-	0	-	-	-	-
		B	*1*<br>*1*<br>-<br>-	*1.060(0.899,1.249)*<br>*1.060(0.900,1.250)*<br>-<br>-	-	-	-	0	-	-	-	-
	**Adjusted for: GA**<br>Yes<br>No	A(a)	*1*<br>*1*<br>-	*1.060(0.899,1.249)*<br>*1.060(0.900,1.250)*<br>-	-	-	-	0	-	-	-	-
		B	*1*<br>*1*<br>-	*1.060(0.899,1.249)*<br>*1.060(0.900,1.250)*<br>-	-	-	-	0	-	-	-	-
	**Adjusted for: Parity**<br>Yes<br>No	A(a)	*1*<br>*1*<br>-	*1.060(0.899,1.249)*<br>*1.060(0.900,1.250)*<br>-	-	-	-	0	-	-	-	-
		B	*1*<br>*1*<br>-	*1.060(0.899,1.249)*<br>*1.060(0.900,1.250)*<br>-	-	-	-	0	-	-	-	-

Birth Outcome indicators	Study characteristics		N	Summary β or OR (95% CI)	P value	I-squared	Tau-squared	N	Summary β or OR (95% CI)	P value	I-squared	Tau-squared
			PFOSA					PFBA				
BW/g	**Gender**	A(a)	2	-55.281 (-148.769, 38.208)	0.130	56.4%	2.6e+03	2	-41.310 (-126.259, 43.638)	0.198	39.8%	1.5e+03
	Male		1	*-104.230 (-194.160, 14.300)*	-	0.0%	0.0000	1	*-85.500 (-180.300, 9.200)*	-	0.0%	0.0000
	Female		1	*-8.800 (-93.550, 75.950)*	-	0.0%	0.0000	1	*1.200 (-90.600, 92.900)*	-	0.0%	0.0000
	**Timing Category**	A(a)	0	-	-	-	-	1	*-42.200 (-111.300, 19.000)*	-	0.0%	0.0000
	First trimester		0	-	-	-	-	0	-	-	-	-
	Second trimester		0	-	-	-	-	0	-	-	-	-
	Third trimester		0	-	-	-	-	0	-	-	-	-
	Cord blood		0	-	-	-	-	1	*-42.200 (-111.300, 19.000)*	-	0.0%	0.0000
	**Study design**	A(a)	2	-0.631 (-1.665, 0.403)	0.144	53.2%	0.2964	1	*-46.200 (-111.300, 19.300)*	-	0.0%	0.0000
	Cross-sectional		0	-	-	-	-	1	*-46.200 (-111.300, 19.300)*	-	0.0%	0.0000
	Case-control		0	-	-	-	-	0	-	-	-	-
	Cohort		2	-0.631 (-1.665, 0.403)	0.144	53.2%	0.2964	0	-	-	-	-
									-			
		B	1	*0.090 (-0.140, 0.320)*	-	0.0%	0.0000	0	-	-	-	-
			0	-	-	-	-	0	-	-	-	-

			0	-	-	-	-	0	-	-	-	-
			1	*0.090 (-0.140, 0.320)*	-	0.0%	0.0000	0	-	-	-	-
	**Setting**	A(a)	2	-0.631 (-1.665, 0.403)	0.144	53.2%	0.2964	1	*-46.200 (-111.300, 19.300)*	-	0.0%	0.0000
	Europe		0	-	-	-	-	0	-	-	-	-
	Asia		0	-	-	-	-	1	*-46.200 (-111.300, 19.300)*	-	0.0%	0.0000
	North America		2	-0.631 (-1.665, 0.403)	0.144	53.2%	0.2964	0	-	-	-	-
	Other		0	-	-	-	-	0	-	-	-	-
		B	1	*0.090 (-0.140, 0.320)*	-	0.0%	0.0000	0	-	-	-	-
			0	-	-	-	-	0	-	-	-	-
			0	-	-	-	-	0	-	-	-	-
			1	*0.090 (-0.140, 0.320)*	-	0.0%	0.0000	0	-	-	-	-
			0	-	-	-	-	0	-	-	-	-
	**Matrix**	A(a)	2	-0.631 (-1.665, 0.403)	0.144	53.2%	0.2964	1	*-46.200 (-111.300, 19.300)*	-	0.0%	0.0000
	Serum		2	-0.631 (-1.665, 0.403)	0.144	53.2%	0.2964	1	*-46.200 (-111.300, 19.300)*	-	0.0%	0.0000
	Plasma		0	-	-	-	-	0	-	-	-	-
	Whole blood		0	-	-	-	-	0		-	-	-

									-			
		B	1<br>1<br>0<br>0	*0.090 (-0.140, 0.320)*<br>*0.090 (-0.140, 0.320)*<br>-<br>-	-<br>-<br>-<br>-	0.0%<br>0.0%<br>-<br>-	0.0000<br>0.0000<br>-<br>-	0<br>0<br>0<br>0	-<br>-<br>-<br>-	-<br>-<br>-<br>-	-<br>-<br>-<br>-	-<br>-<br>-<br>-
	**Adjusted for: GA**<br>Yes<br>No	A(a)	2<br>0<br>2	-0.631 (-1.665, 0.403)<br>-<br>-0.631 (-1.665, 0.403)	0.144<br>-<br>0.144	53.2%<br>-<br>53.2%	0.2964<br>-<br>0.2964	1<br>1<br>0	*-46.200 (-111.300, 19.300)*<br>*-46.200 (-111.300, 19.300)*<br>-	-<br>-<br>-	0.0%<br>0.0%<br>-	0.0000<br>0.0000<br>-
		B	1<br>0<br>1	*0.090 (-0.140, 0.320)*<br>-<br>*0.090 (-0.140, 0.320)*	-<br>-<br>-	0.0%<br>-<br>0.0%	0.0000<br>-<br>0.0000	0<br>0<br>0	-<br>-<br>-	-<br>-<br>-	-<br>-<br>-	-<br>-<br>-
	**Adjusted for: Parity**<br>Yes<br>No	A(a)	2<br>0<br>2	-0.631 (-1.665, 0.403)<br>-<br>-0.631 (-1.665, 0.403)	0.144<br>-<br>0.144	53.2%<br>-<br>53.2%	0.2964<br>-<br>0.2964	1<br>1<br>0	*-46.200 (-111.300, 19.300)*<br>*-46.200 (-111.300, 19.300)*<br>-	-<br>-<br>-	0.0%<br>0.0%<br>-	0.0000<br>0.0000<br>-

			1	*0.090 (-0.140, 0.320)*	-	0.0%	0.0000	0	-	-	-	-
		B	0	-	-	-	-	0	-	-	-	-
			1	*0.090 (-0.140, 0.320)*	-	0.0%	0.0000	0	-	-	-	-
	**Gender**		0	-	-	-	-	2	-0.008 (-0.198, 0.182)	0.798	0.0%	0.0000
	Male	A(a)	0	-	-	-	-	1	*0.020 (-0.270, 0.300)*	-	0.0%	0.0000
	Female		0	-	-	-	-	1	*-0.030 (-0.290, 0.220)*	-	0.0%	0.0000
	**Timing Category**		0	-	-	-	-	1	*0.020 (-0.050, 0.100)*	-	0.0%	0.0000
	First trimester		0	-	-	-	-	0	-	-	-	-
	Second trimester	A(a)	0	-	-	-	-	0	-	-	-	-
	Third trimester		0	-	-	-	-	1	*0.020 (-0.050, 0.100)*	-	0.0%	0.0000
**GA**	Cord blood		0	-	-	-	-	0	-	-	-	-
**(weeks)**			0	-	-	-	-	1	*0.010 (-0.180, 0.200)*	-	0.0%	0.0000
		A(a)	0	-	-	-	-	1	*0.010 (-0.180, 0.200)*	-	0.0%	0.0000
	**Study design**		0	-	-	-	-	0	-	-	-	-
	Cross-sectional		0	-	-	-	-	0	-	-	-	-
	Case-control		1	*0.180 (-0.270, 0.630)*	-	0.0%	0.0000	0	-	-	-	-
	Cohort	B	0	-	-	-	-	0	-	-	-	-
			0	-	-	-	-	0	-	-	-	-
			1	*0.180 (-0.270, 0.630)*	-	0.0%	0.0000	0	-	-	-	-

	**Setting**<br>Europe<br>Asia<br>America<br>Other	A(a)	0	-	-	-	-	1	*0.010 (-0.180, 0.200)*	-	0.0%	0.0000
			0	-	-	-	-	0	-	-	-	-
			0	-	-	-	-	1	*0.010 (-0.180, 0.200)*	-	0.0%	0.0000
			0	-	-	-	-	0	-	-	-	-
			0	-	-	-	-	0	-	-	-	-
		B	1	*0.010 (-0.180, 0.200)*	-	0.0%	0.0000	0	-	-	-	-
			0	-	-	-	-	0	-	-	-	-
			0	-	-	-	-	0	-	-	-	-
			1	*0.010 (-0.180, 0.200)*	-	0.0%	0.0000	0	-	-	-	-
			0	-	-	-	-	0	-	-	-	-
	**Matrix**<br>Serum<br>Plasma<br>Whole blood	A(a)	0	-	-	-	-	1	*0.010 (-0.180, 0.200)*	-	0.0%	0.0000
			0	-	-	-	-	1	*0.010 (-0.180, 0.200)*	-	0.0%	0.0000
			0	-	-	-	-	0	-	-	-	-
			0	-	-	-	-	0	-	-	-	-
		B	1	*0.180 (-0.270, 0.630)*	-	0.0%	0.0000	0	-	-	-	-
			1	*0.180 (-0.270, 0.630)*	-	0.0%	0.0000	0	-	-	-	-
			0	-	-	-	-	0	-	-	-	-
			0	-	-	-	-	0	-	-	-	-

	**Adjusted for: GA**	A(a)	0	-	-	-	-	1	*0.010 (-0.180, 0.200)*	-	0.0%	0.0000
	Yes		0	-	-	-	-	0	-	-	-	-
	No		0	-	-	-	-	1	*0.010 (-0.180, 0.200)*	-	0.0%	0.0000
		B	1	*0.180 (-0.270, 0.630)*	-	0.0%	0.0000	0	-	-	-	-
			0	-	-	-	-	0	-	-	-	-
			1	*0.180 (-0.270, 0.630)*	-	0.0%	0.0000	0	-	-	-	-
	**Adjusted for: Parity**	A(a)	0	-	-	-	-	1	*0.010 (-0.180, 0.200)*	-	0.0%	0.0000
	Yes		0	-	-	-	-	1	*0.010 (-0.180, 0.200)*	-	0.0%	0.0000
	No		0	-	-	-	-	0	-	-	-	-
		B	1	*0.180 (-0.270, 0.630)*	-	0.0%	0.0000	0	-	-	-	-
			1	*0.180 (-0.270, 0.630)*	-	0.0%	0.0000	0	-	-	-	-
			0	-	-	-	-	0	-	-	-	-
**PTB**	**Study design**	A(a)	0	-	-	-	-	0	-	-	-	-
	Cross-sectional		0	-	-	-	-	0	-	-	-	-
	Case-control		0	-	-	-	-	0	-	-	-	-
	Cohort		0	-	-	-	-	0	-	-	-	-
		B	1	*1.140 (0.490, 2.630)*	-	0.0%	0.0000	0	-	-	-	-
			0	-	-	-	-	0	-	-	-	-
			0	-	-	-	-	0	-	-	-	-

			1	*1.140 (0.490, 2.630)*	-	0.0%	0.0000	0	-	-	-	-
	**Setting**<br>Europe<br>Asia<br>North America<br>Other	A(a)	0	-	-	-	-	0	-	-	-	-
			0	-	-	-	-	0	-	-	-	-
			0	-	-	-	-	0	-	-	-	-
			0	-	-	-	-	0	-	-	-	-
			0	-	-	-	-	0	-	-	-	-
		B	1	*1.140 (0.490, 2.630)*	-	0.0%	0.0000	0	-	-	-	-
			0	-	-	-	-	0	-	-	-	-
			0	-	-	-	-	0	-	-	-	-
			1	*1.140 (0.490, 2.630)*	-	0.0%	0.0000	0	-	-	-	-
			0	-	-	-	-	0	-	-	-	-
	**Matrix**<br>Serum<br>Plasma<br>Whole blood	A(a)	0	-	-	-	-	0	-	-	-	-
			0	-	-	-	-	0	-	-	-	-
			0	-	-	-	-	0	-	-	-	-
			0	-	-	-	-	0	-	-	-	-
		B	1	*1.140 (0.490, 2.630)*	-	0.0%	0.0000	0	-	-	-	-
			1	*1.140 (0.490, 2.630)*	-	0.0%	0.0000	0	-	-	-	-
			0	-	-	-	-	0	-	-	-	-

			0	-	-	-	-	0	-	-	-	-
			0	-	-	-	-	0	-	-	-	-
	**Adjusted for: GA**	A(a)	0	-	-	-	-	0	-	-	-	-
	Yes		0	-	-	-	-	0	-	-	-	-
	No		1	*1.140 (0.490, 2.630)*	-	0.0%	0.0000	0	-	-	-	-
		B	0	-	-	-	-	0	-	-	-	-
			1	*1.140 (0.490, 2.630)*	-	0.0%	0.0000	0	-	-	-	-
			0	-	-	-	-	0	-	-	-	-
	**Adjusted for:**	A(a)	0	-	-	-	-	0	-	-	-	-
	**Parity**		0	-	-	-	-	0	-	-	-	-
	Yes		1	*1.140 (0.490, 2.630)*	-	0.0%	0.0000	0	-	-	-	-
	No	B	1	*1.140 (0.490, 2.630)*	-	0.0%	0.0000	0	-	-	-	-
			0	-	-	-	-	0	-	-	-	-

Fig S1. Forest plot on the effect of PFOA on BW (birth weight)/g (quality effects model) for (A) per 1 ln(ng/ml) increment , (B) per 1 ng/ml increment and (C) for high versus low categories of exposure.

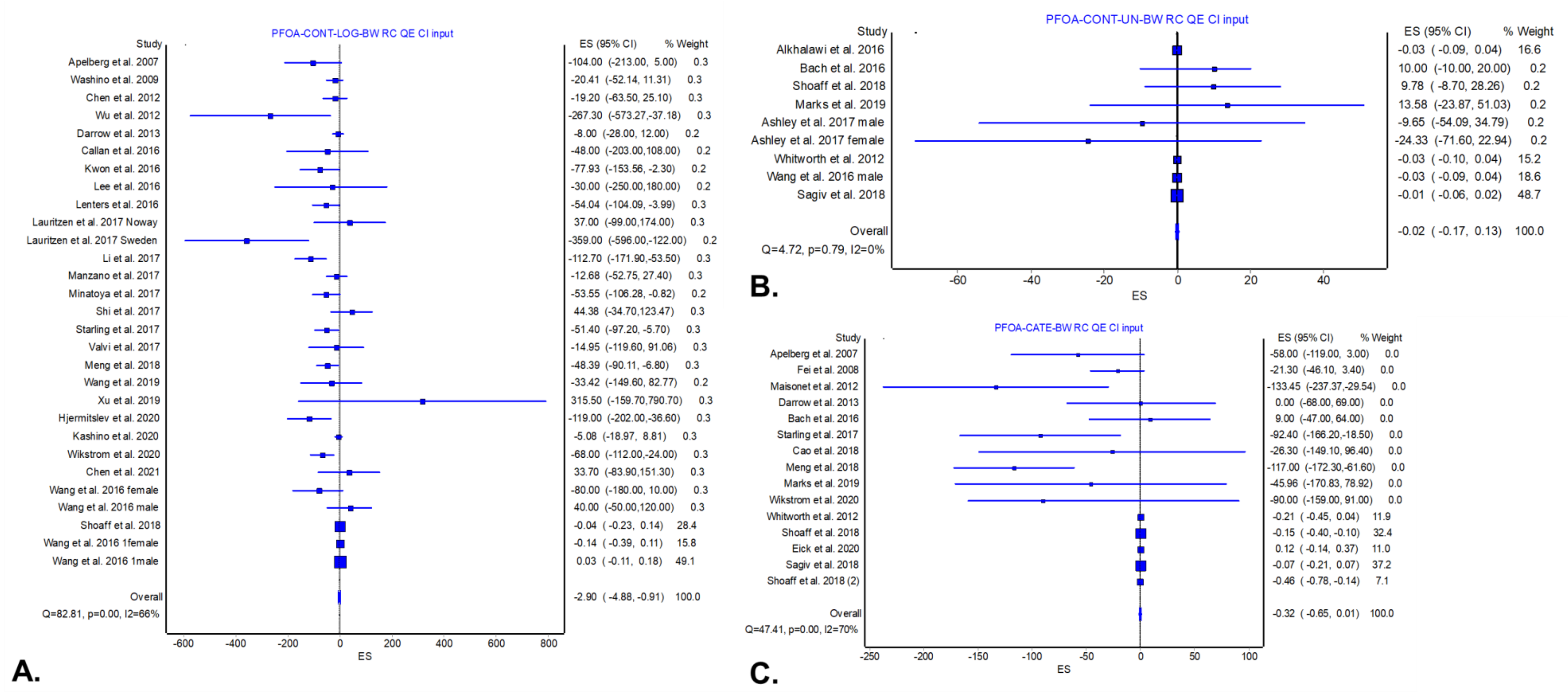

Fig S2. Forest plot on the effect of PFOA on BL (birth length)/cm (quality effects model) for (A) per 1 ln(ng/ml) increment , (B) per 1 ng/ml increment and (C) for high versus low categories of exposure.

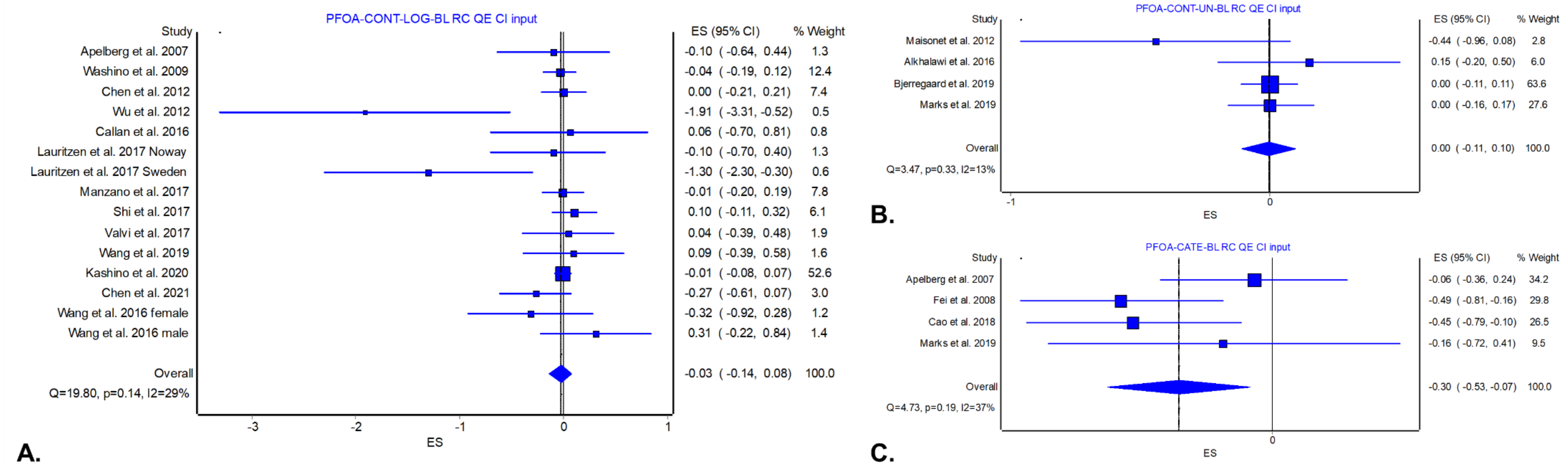

Fig S3. Forest plot on the effect of PFOA on HC (head circumference)/cm (quality effects model) for (A) per 1 ln(ng/ml) increment , (B) per 1 ng/ml increment and (C) for high versus low categories of exposure.

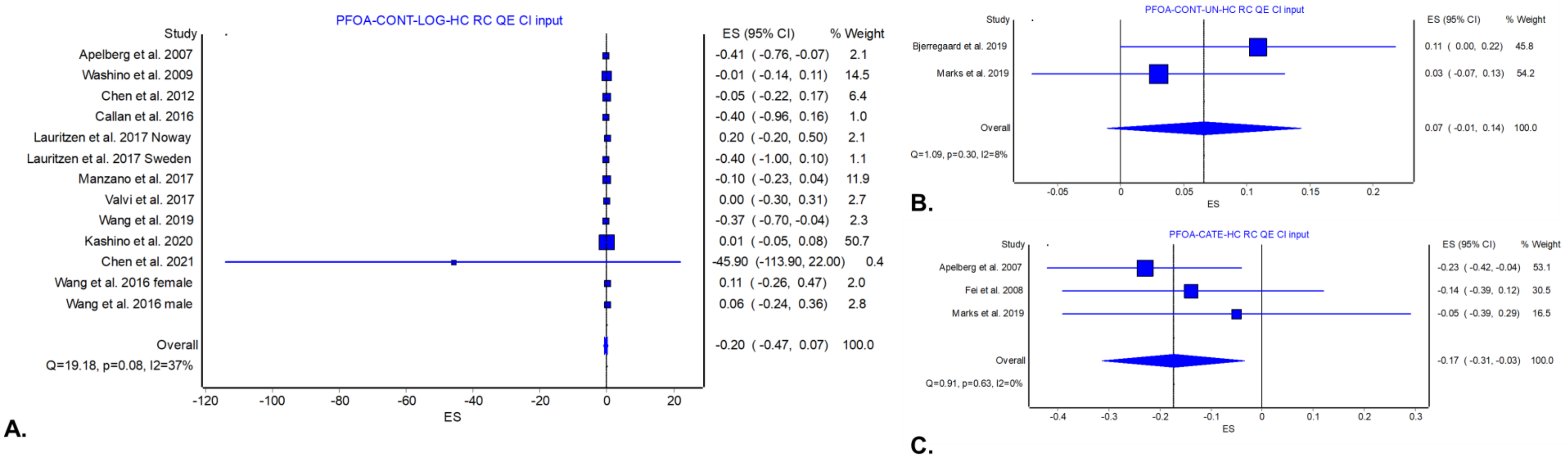

Fig S4. Forest plot on the effect of PFOA on GA (gestational age)/weeks (quality effects model) for (A) per 1 ln(ng/ml) increment , (B) for high versus low categories of exposure.

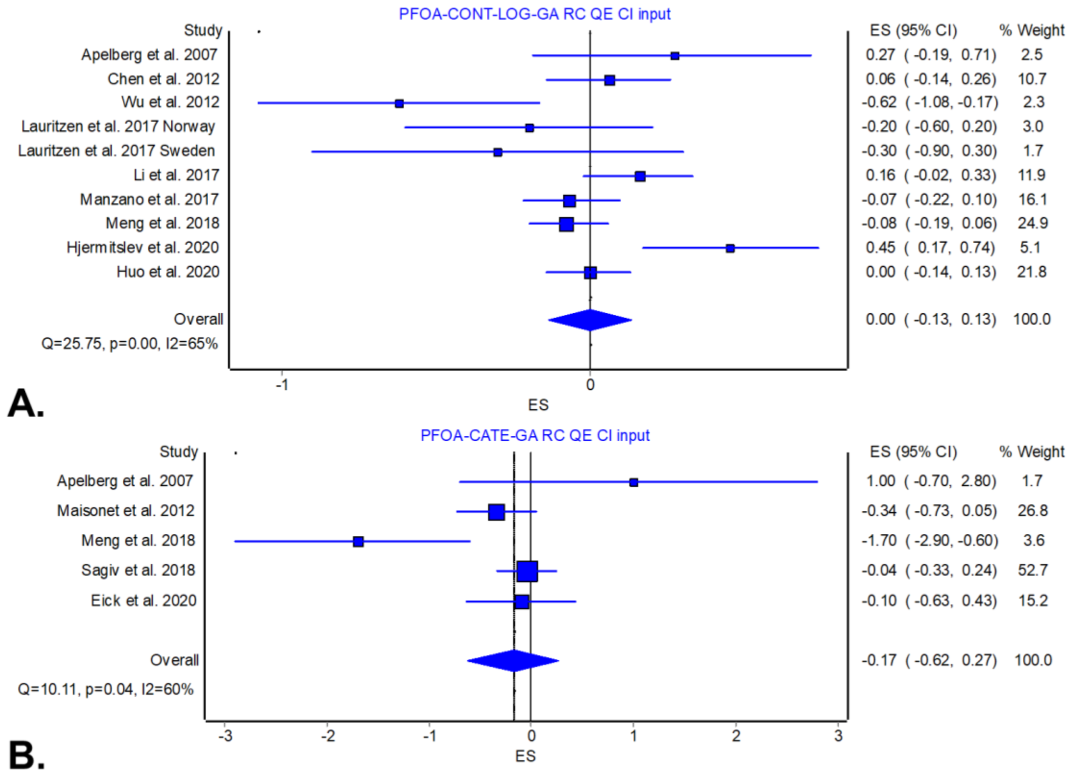

Fig S5. Forest plot on the effect of PFOA on PI (Ponderal index)/g/cm2 *100 (quality effects model) for (A) per 1 ln(ng/ml) increment , (B) per 1 ng/ml increment and (C) for high versus low categories of exposure.

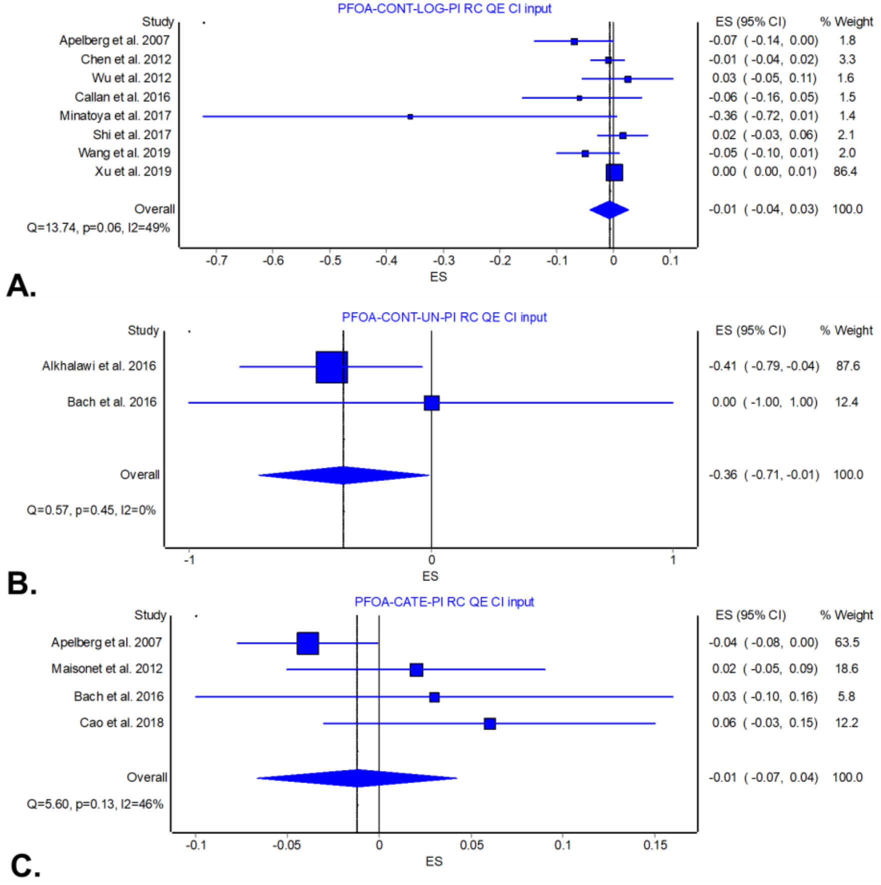

Fig S6. Forest plot on the effect of PFOA on PTB (preterm birth) (quality effects model) for (A) per 1 ln(ng/ml) increment , (B) for high versus low categories of exposure.

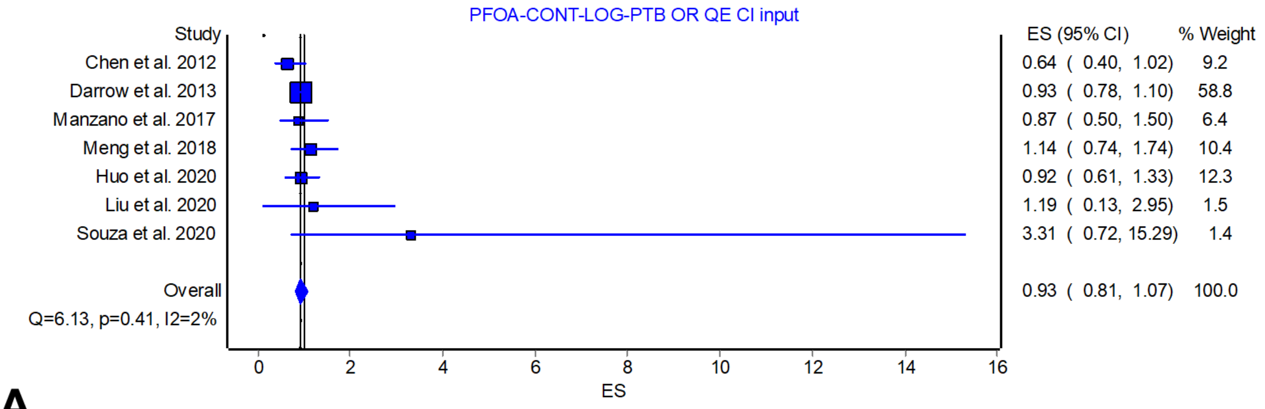

A.

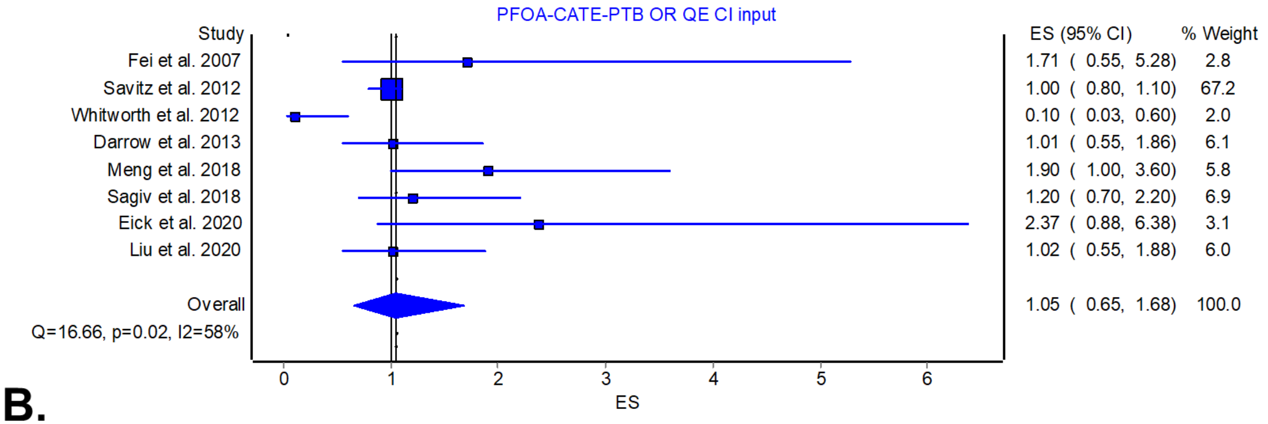

B.

Fig S7. Forest plot on the effect of PFOA on LBW (low birth weight) (quality effects model) for (A) per 1 ln(ng/ml) increment , (B) for high versus low categories of exposure.

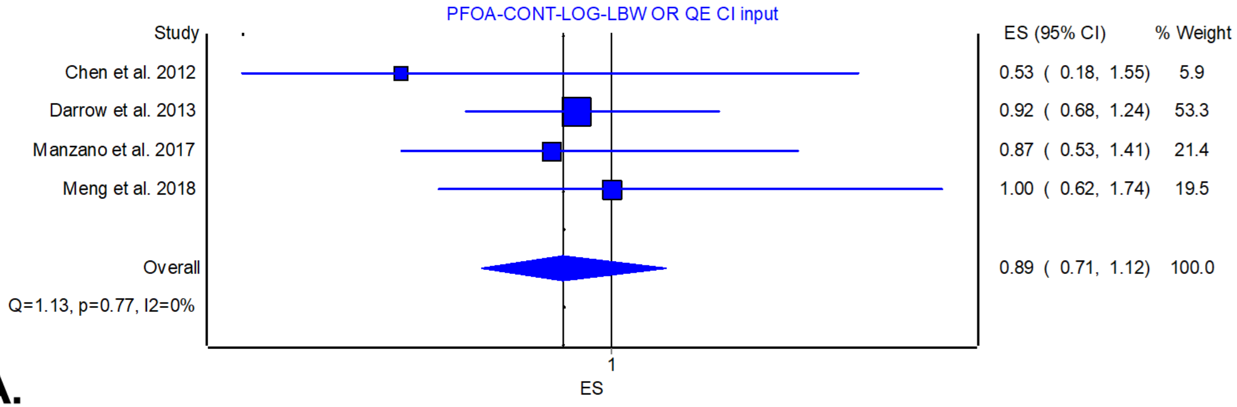

A.

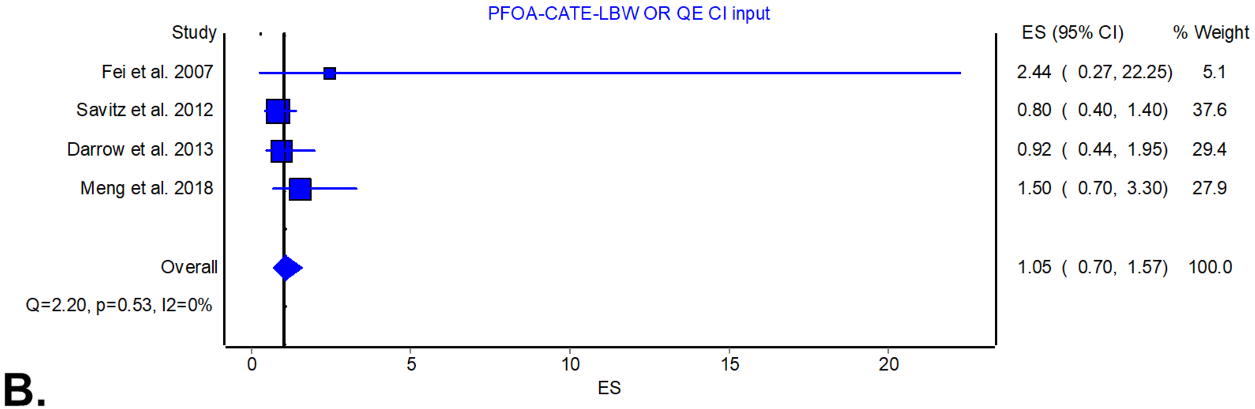

B.

Fig S8. Forest plot on the effect of PFOA on SGA (small for gestational age) (quality effects model) for (A) per 1 ln(ng/ml) increment , (B) for high versus low categories of exposure.

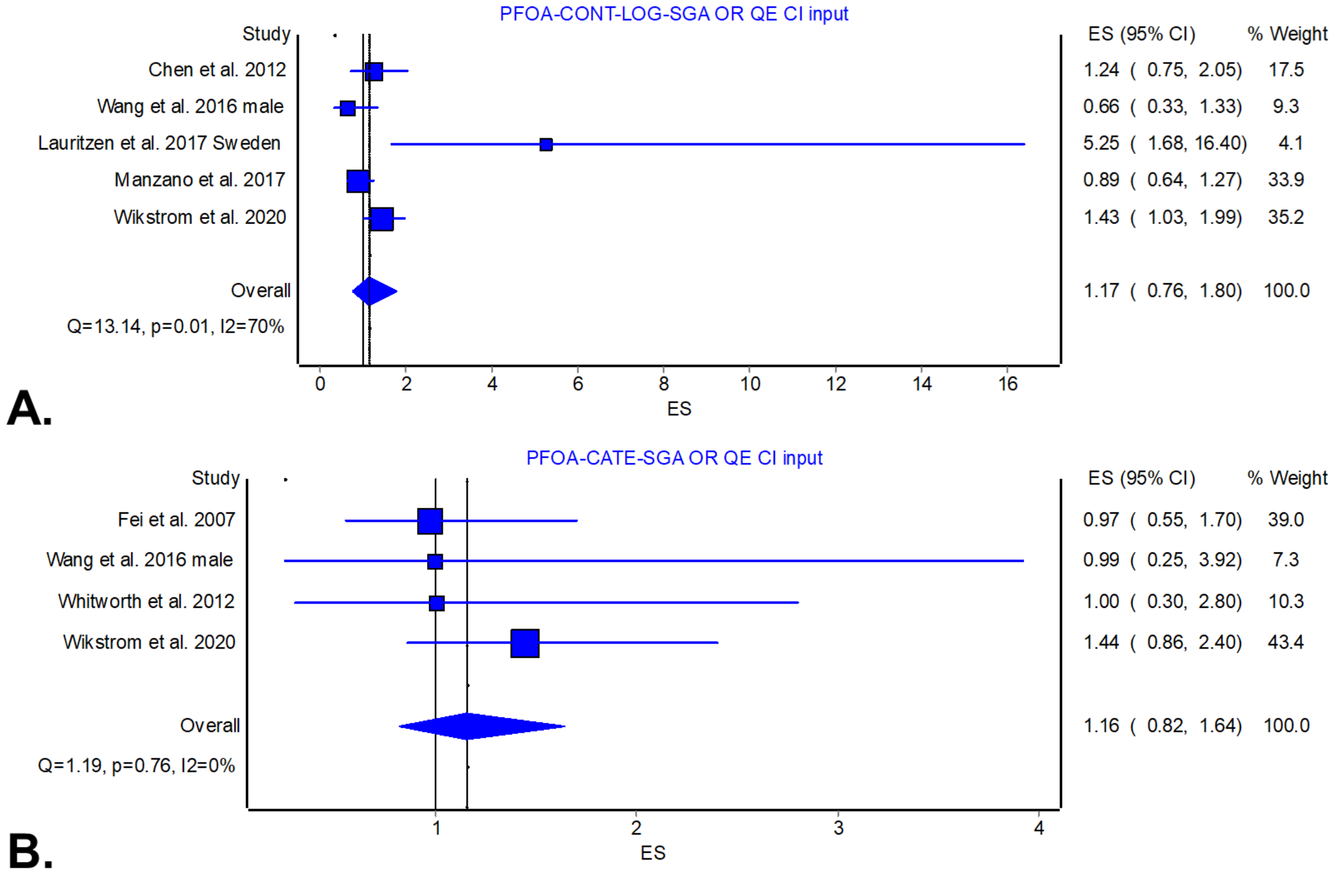

Fig S9. Forest plot on the effect of PFOS on BW (birth weight)/g (quality effects model) for (A) per 1 ln(ng/ml) increment , (B) per 1 ng/ml increment and (C) for high versus low categories of exposure.

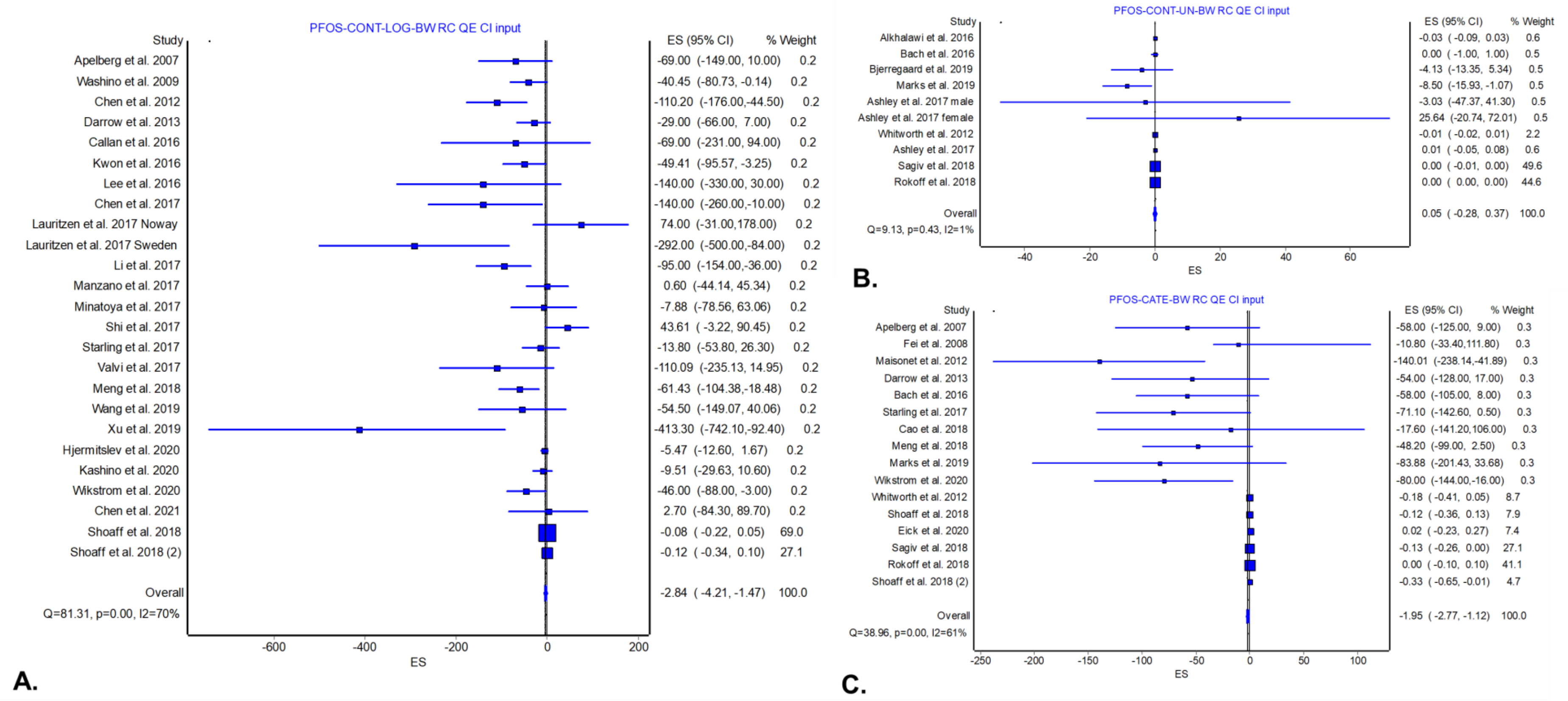

Fig S10. Forest plot on the effect of PFOS on BL (birth length)/cm (quality effects model) for (A) per 1 ln(ng/ml) increment , (B) per 1 ng/ml increment and (C) for high versus low categories of exposure.

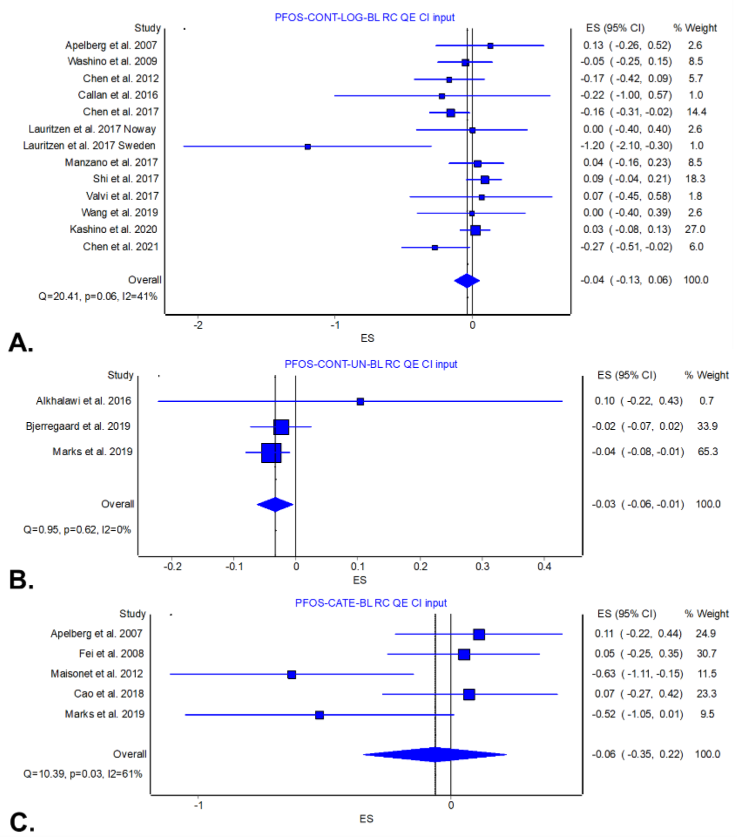

Fig S11. Forest plot on the effect of PFOS on HC (head circumference)/cm (quality effects model) for (A) per 1 ln(ng/ml) increment , (B) per 1 ng/ml increment and (C) for high versus low categories of exposure.

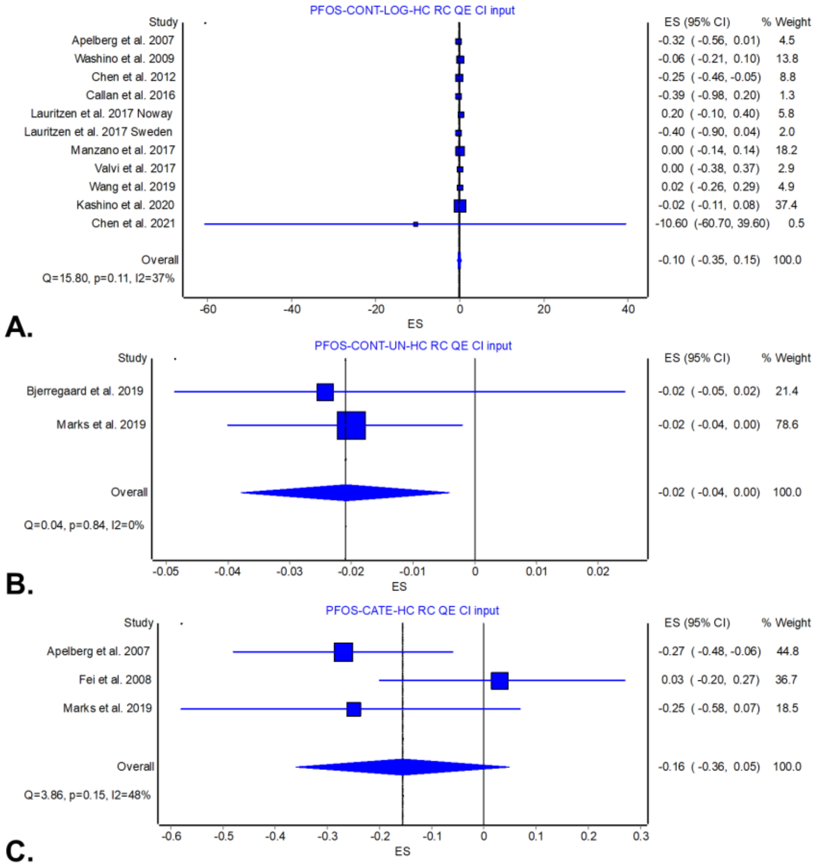

Fig S12. Forest plot on the effect of PFOS on GA (gestational age)/weeks (quality effects model) for (A) per 1 ln(ng/ml) increment , (B) for high versus low categories of exposure.

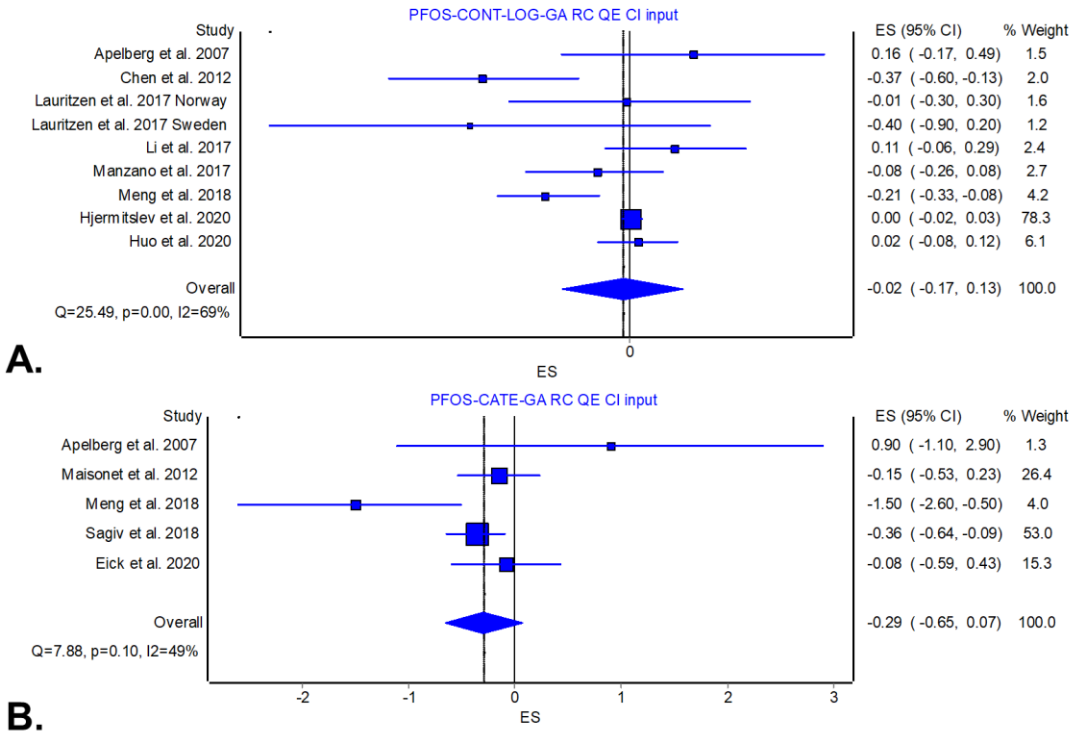

Fig S13. Forest plot on the effect of PFOS on PI (Ponderal index)/g/cm2 *100 (quality effects model) for (A) per 1 ln(ng/ml) increment , (B) per 1 ng/ml increment and (C) for high versus low categories of exposure.

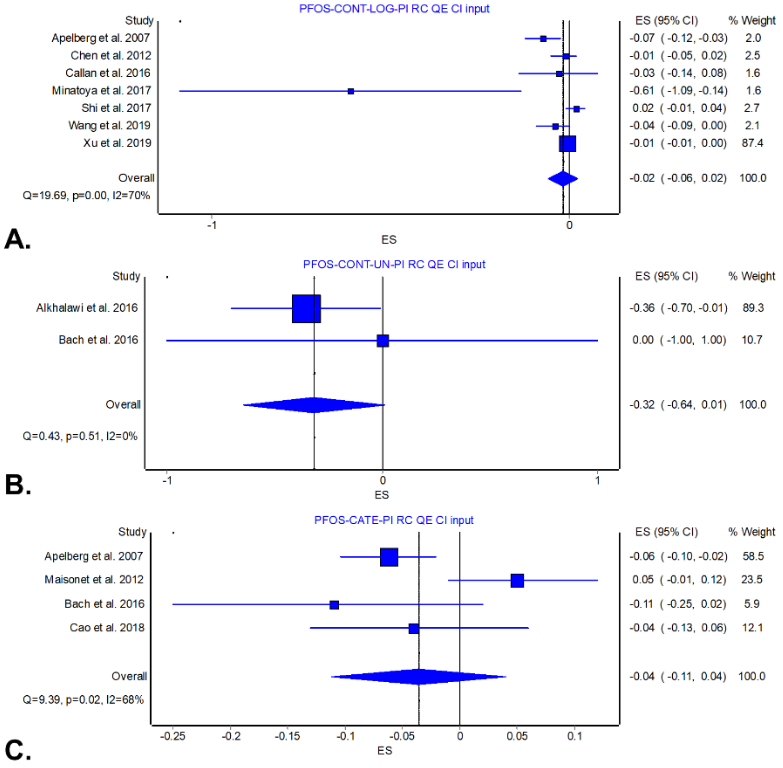

Fig S14. Forest plot on the effect of PFOS on PTB (preterm birth) (quality effects model) for (A) per 1 ln(ng/ml) increment , (B) per 1 ng/ml increment and (C) for high versus low categories of exposure.

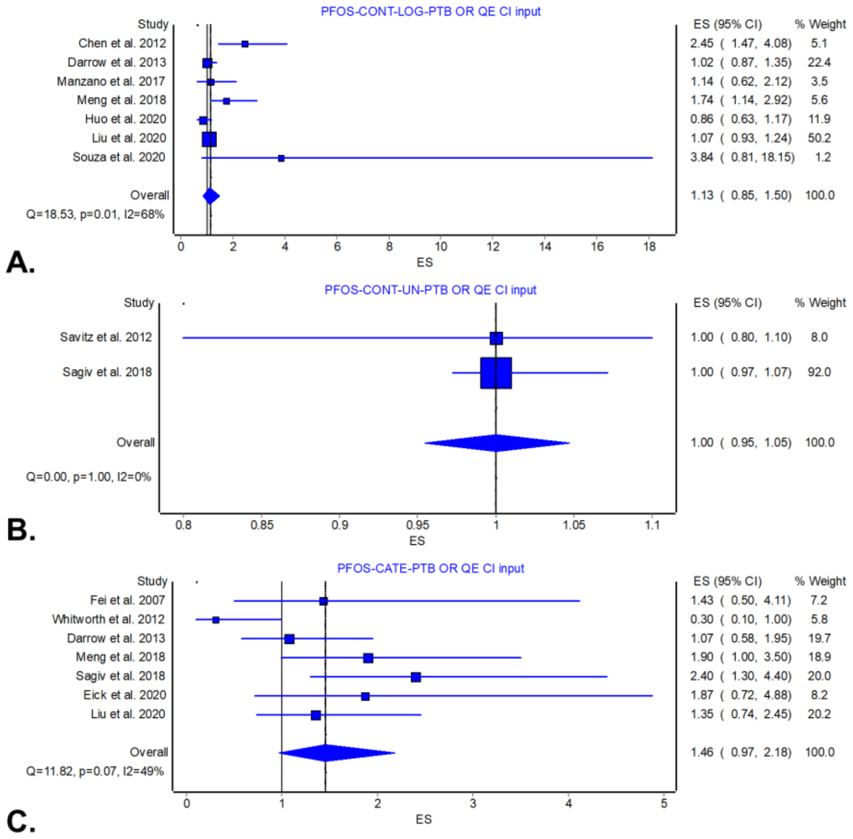

Fig S15. Forest plot on the effect of PFOS on LBW (low birth weight) (quality effects model) for (A) per 1 ln(ng/ml) increment , (B) for high versus low categories of exposure.

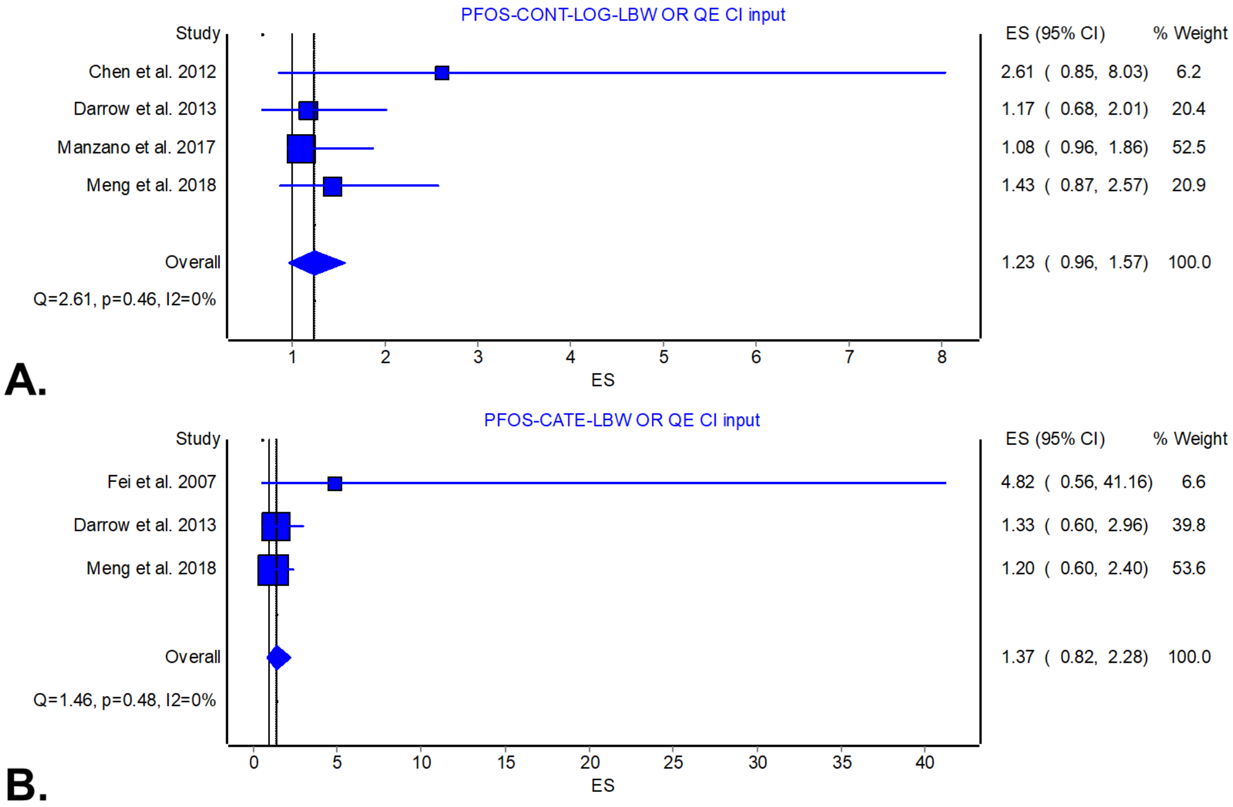

Fig S16. Forest plot on the effect of PFOS on SGA (small for gestational age) (quality effects model) for (A) per 1 ln(ng/ml) increment , (B) for high versus low categories of exposure.

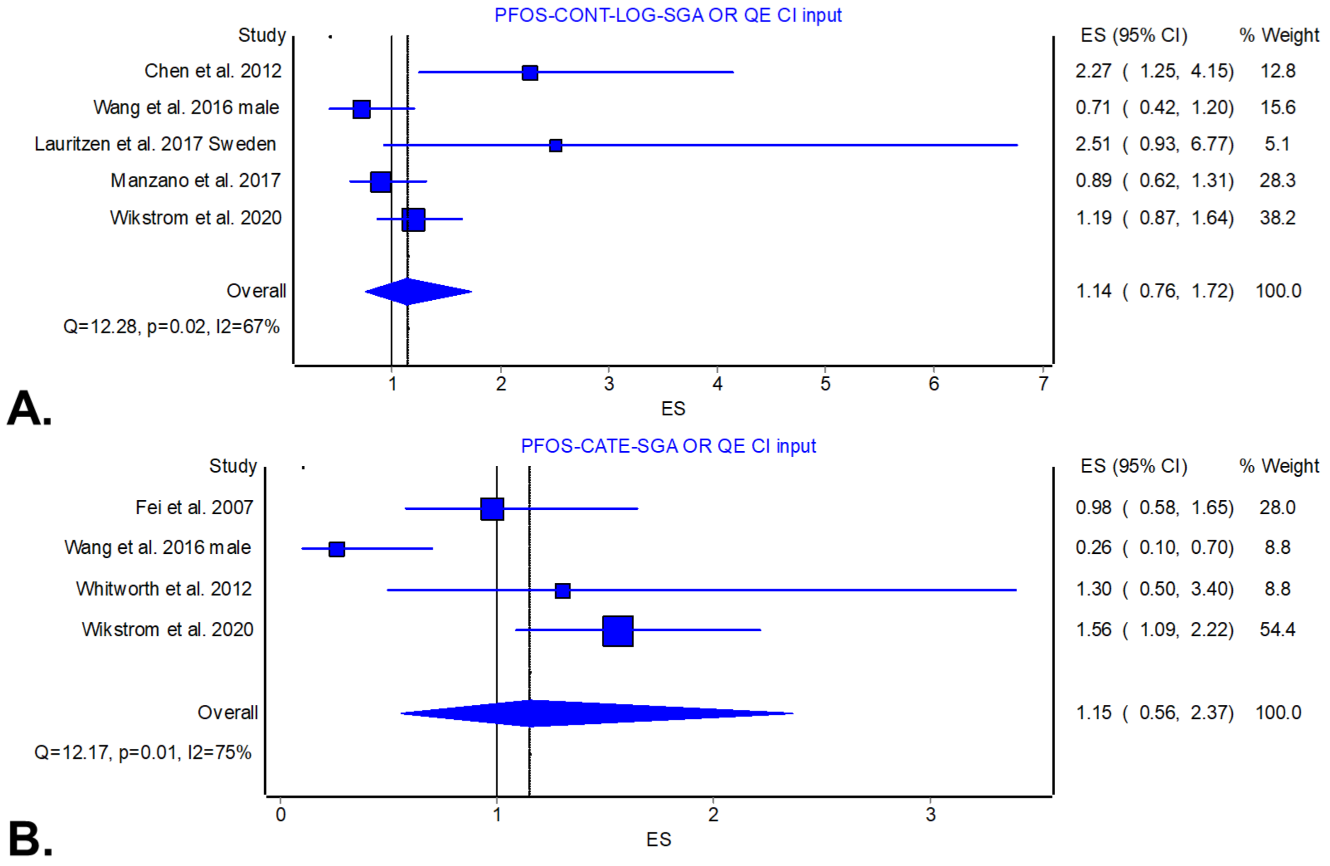

Fig S17. Forest plot on the effect of PFDA on BW (birth weight)/g (quality effects model) for (A) per 1 ln(ng/ml) increment , (B) per 1 ng/ml increment and (C) for high versus low categories of exposure.

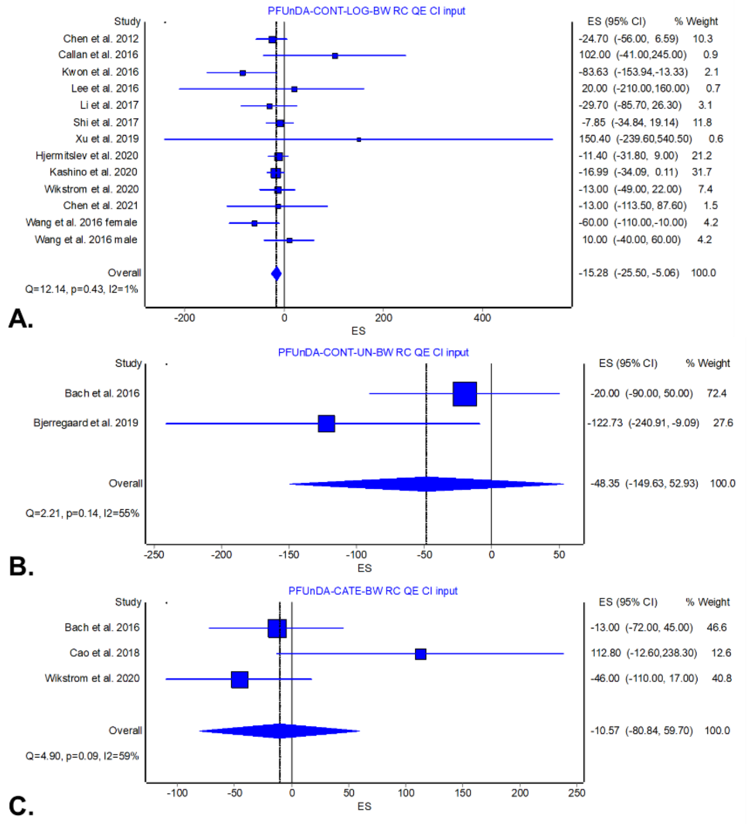

Fig S18. Forest plot on the effect of PFHxS on BW (birth weight)/g (quality effects model) for (A) per 1 ln(ng/ml) increment , (B) per 1 ng/ml increment and (C) for high versus low categories of exposure.

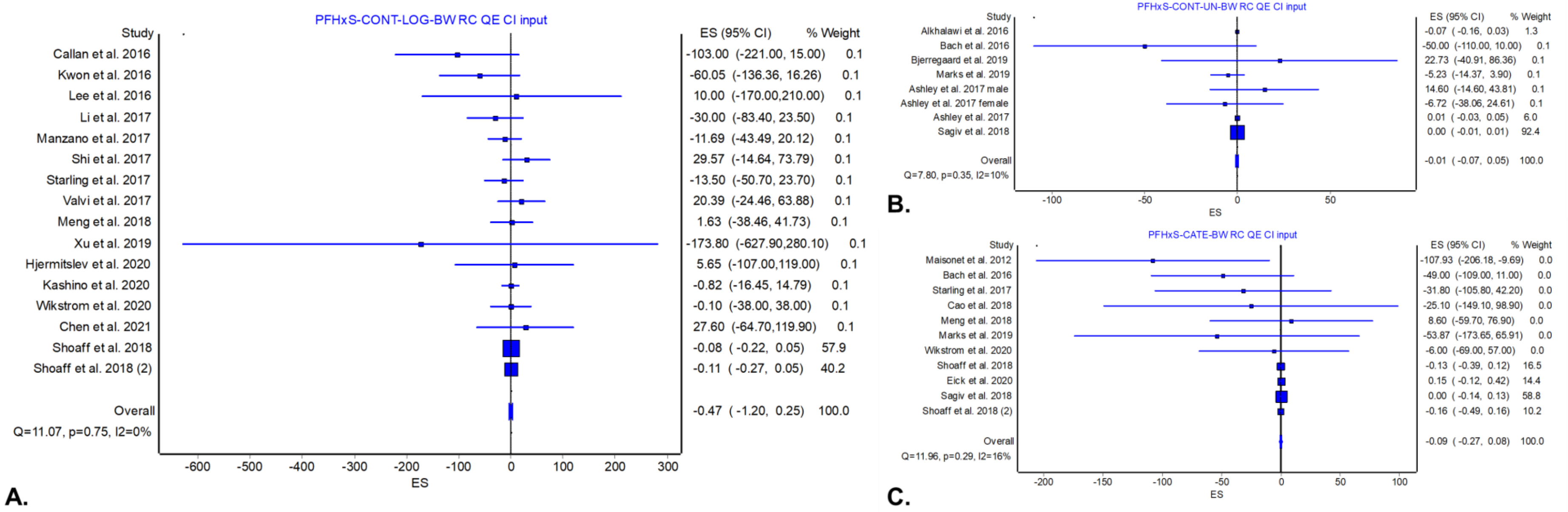

Fig S19. Forest plot on the effect of PFNA on BW (birth weight)/g (quality effects model) for (A) per 1 ln(ng/ml) increment , (B) per 1 ng/ml increment and (C) for high versus low categories of exposure.

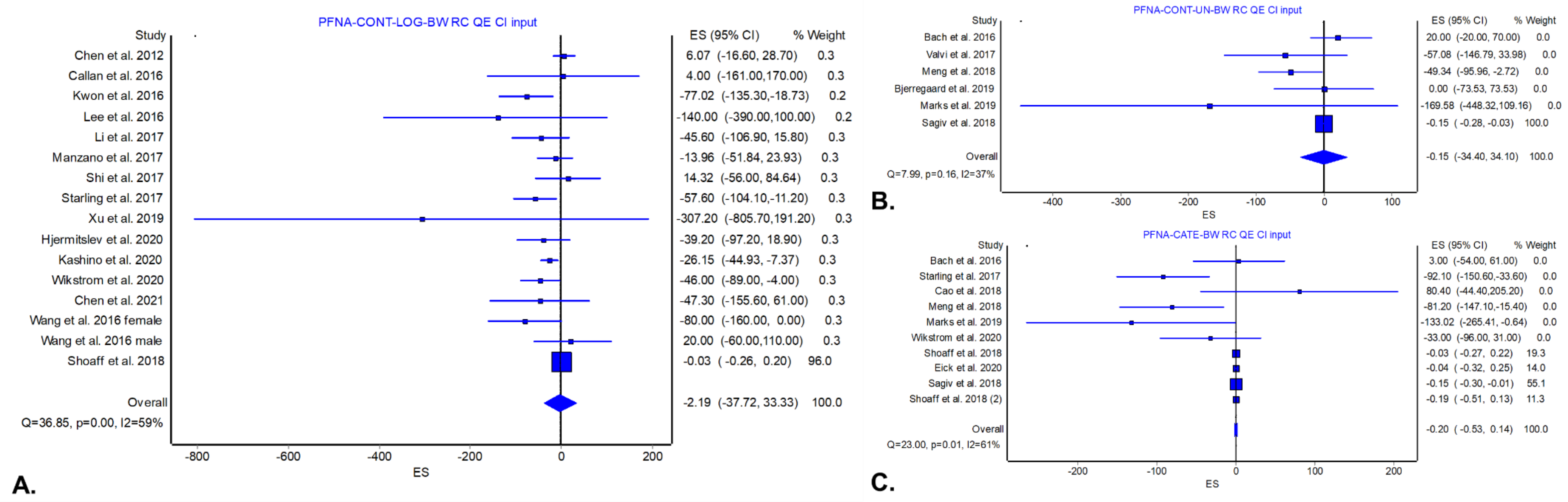

Fig S20. Forest plot on the effect of PFDA on BW (birth weight)/g (quality effects model) for (A) per 1 ln(ng/ml) increment , (B) per 1 ng/ml increment and (C) for high versus low categories of exposure.

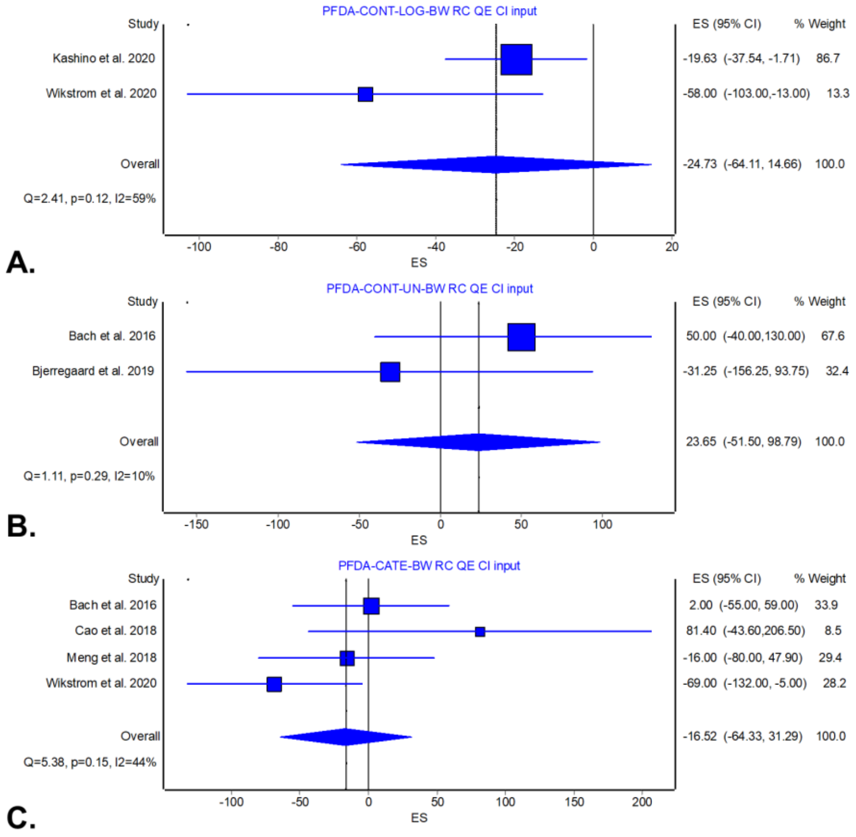

Fig S21. Forest plot on the effect of (A)PFDoDA (B)PFTrDA (C)PFHpA (D)PFDeA (E)PFOSA on BW (birth weight)/g (quality effects model) for per 1 ln(ng/ml) increment of exposure.

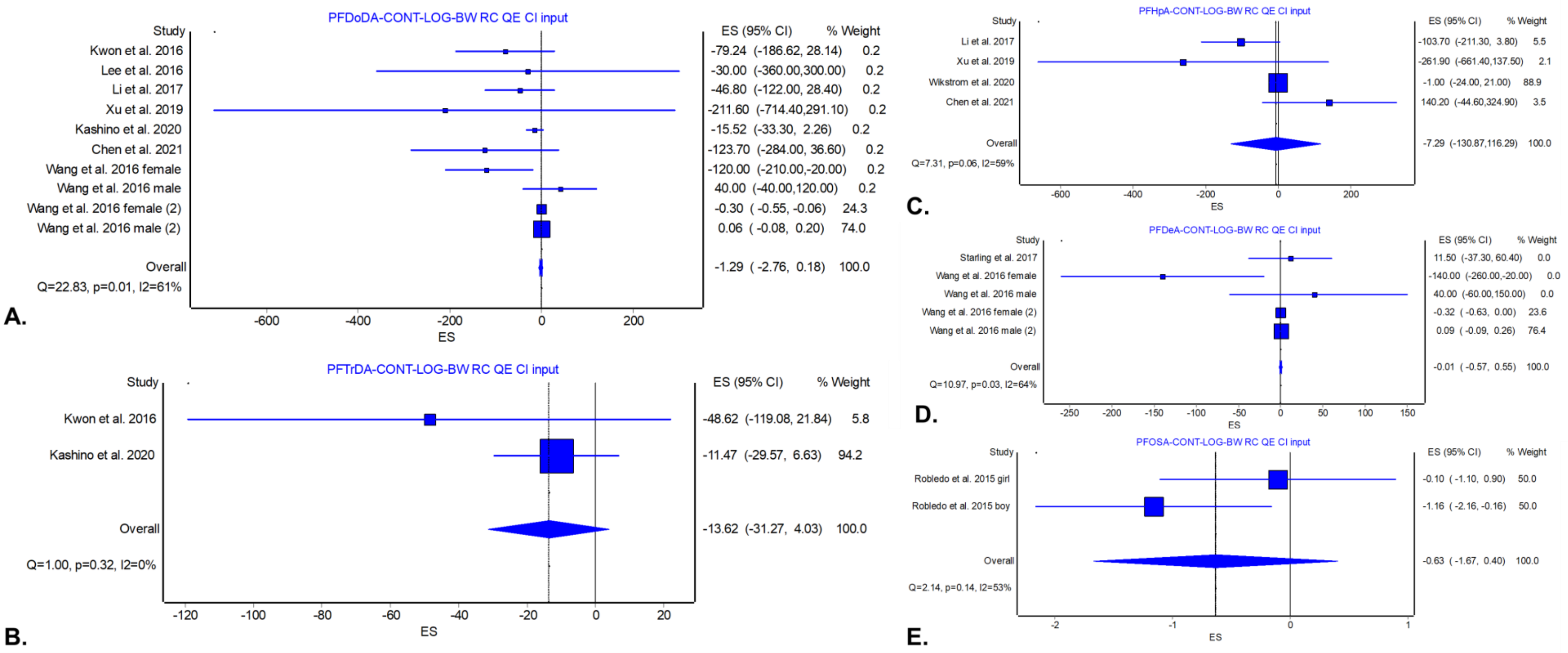

Fig S22. Forest plot on the effect of (a)PFNA (b)PFHxS on BL (birth length)/cm (quality effects model) for (A) per 1 ln(ng/ml) increment , (B) per 1 ng/ml increment and (C) for high versus low categories of exposure.

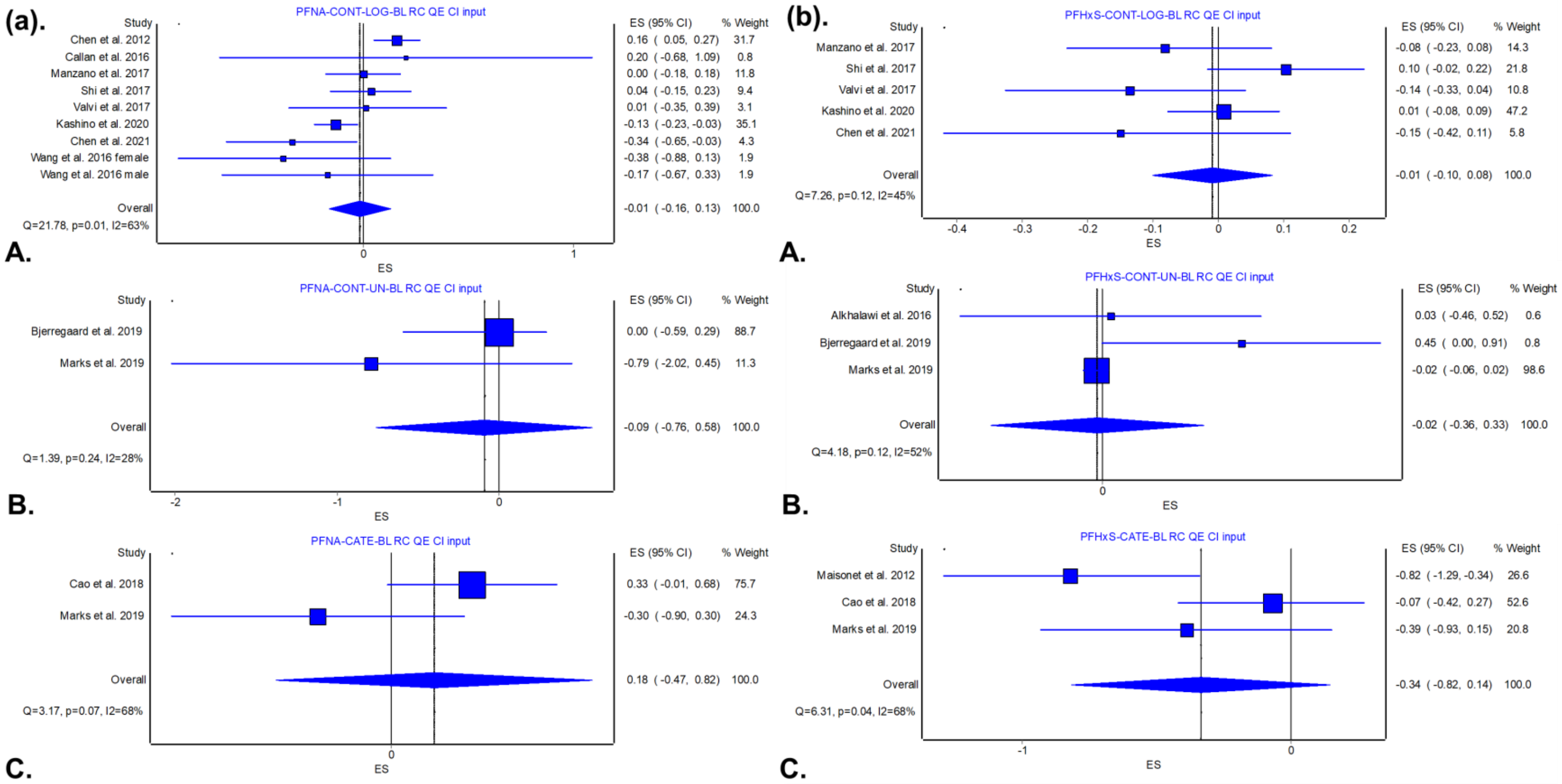

Fig S23. Forest plot on the effect of (A)PFDeDA (B)PFDA (C)PFDoDA (D)PFUnDA on BL (birth length)/cm (quality effects model) for per 1 ln(ng/ml) increment of exposure.

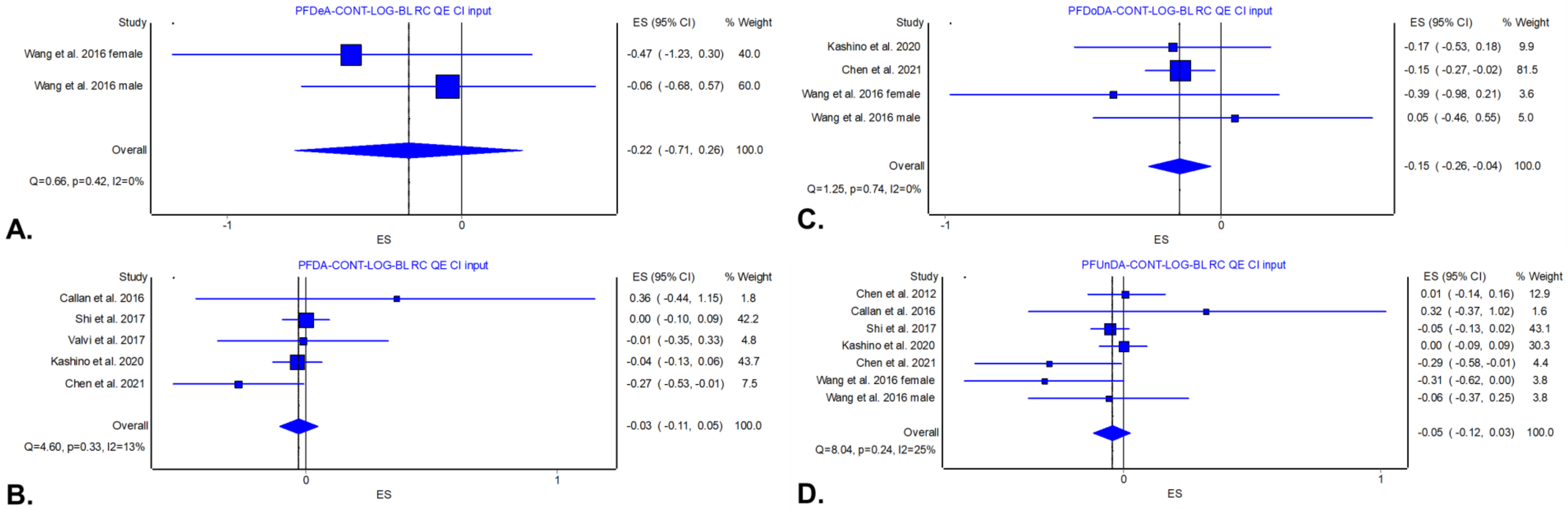

Fig S24. Forest plot on the effect of (a)PFNA (b)PFHxS on HC (head circumference)/cm (quality effects model) for (A) per 1 ln(ng/ml) increment and (B) per 1 ng/ml increment of exposure.

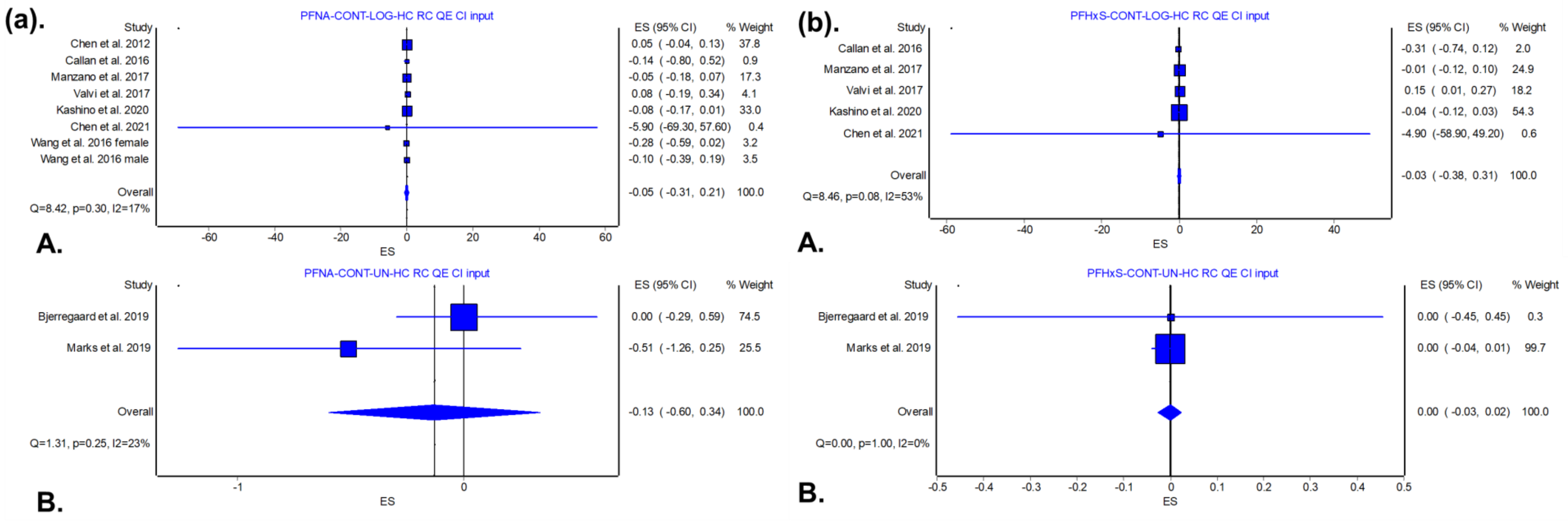

Fig S25. Forest plot on the effect of (A) PFDA (B) PFDeDA (C)PFDoDA (D)PFUnDA on HC (head circumference)/cm (quality effects model) for per 1 ln(ng/ml) increment of exposure.

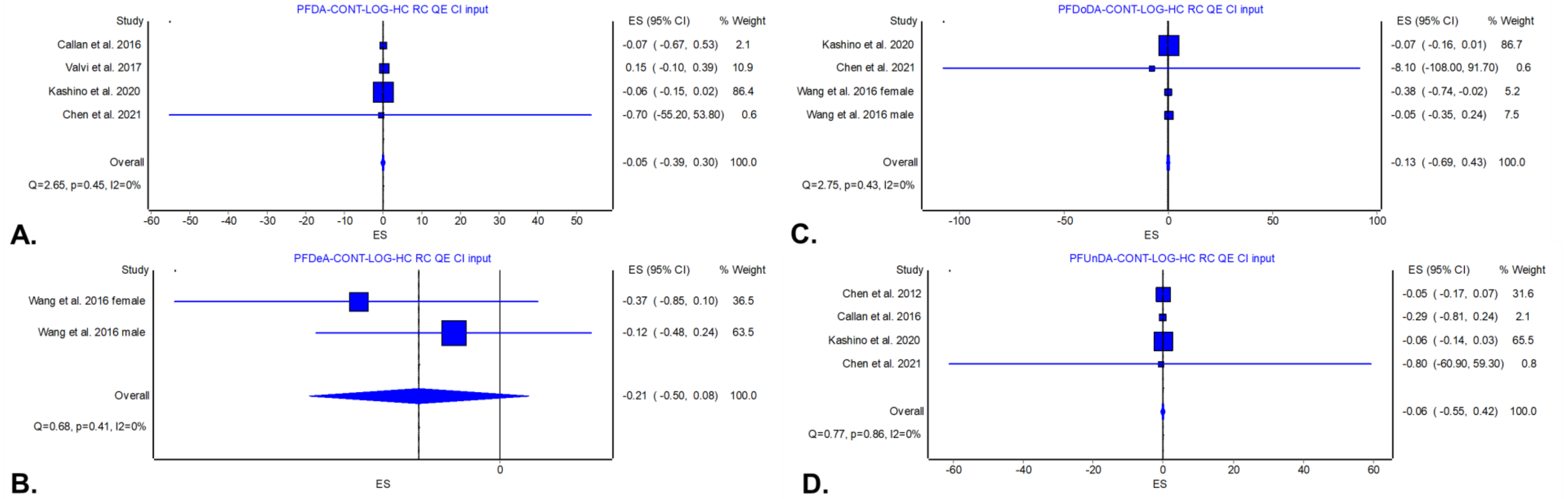

Fig S26. Forest plot on the effect of (a)PFNA (b)PFHxS on GA (gestational age)/weeks (quality effects model) for (A) per 1 ln(ng/ml) increment and (B) per 1 ng/ml increment of exposure.

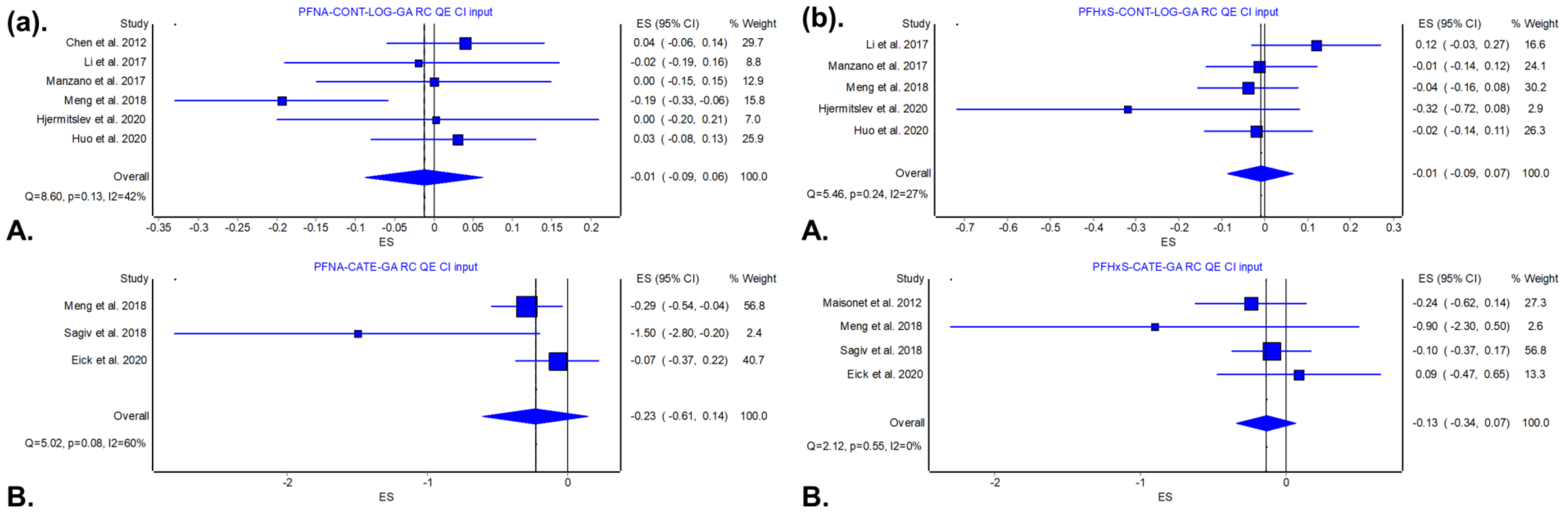

Fig S27. Forest plot on the effect of (A) PFDA (B) PFHpS (C)PFDoDA (D)PFUnDA (E)PFHpA on GA (gestational age)/weeks (quality effects model) for per 1 ln(ng/ml) increment of exposure.

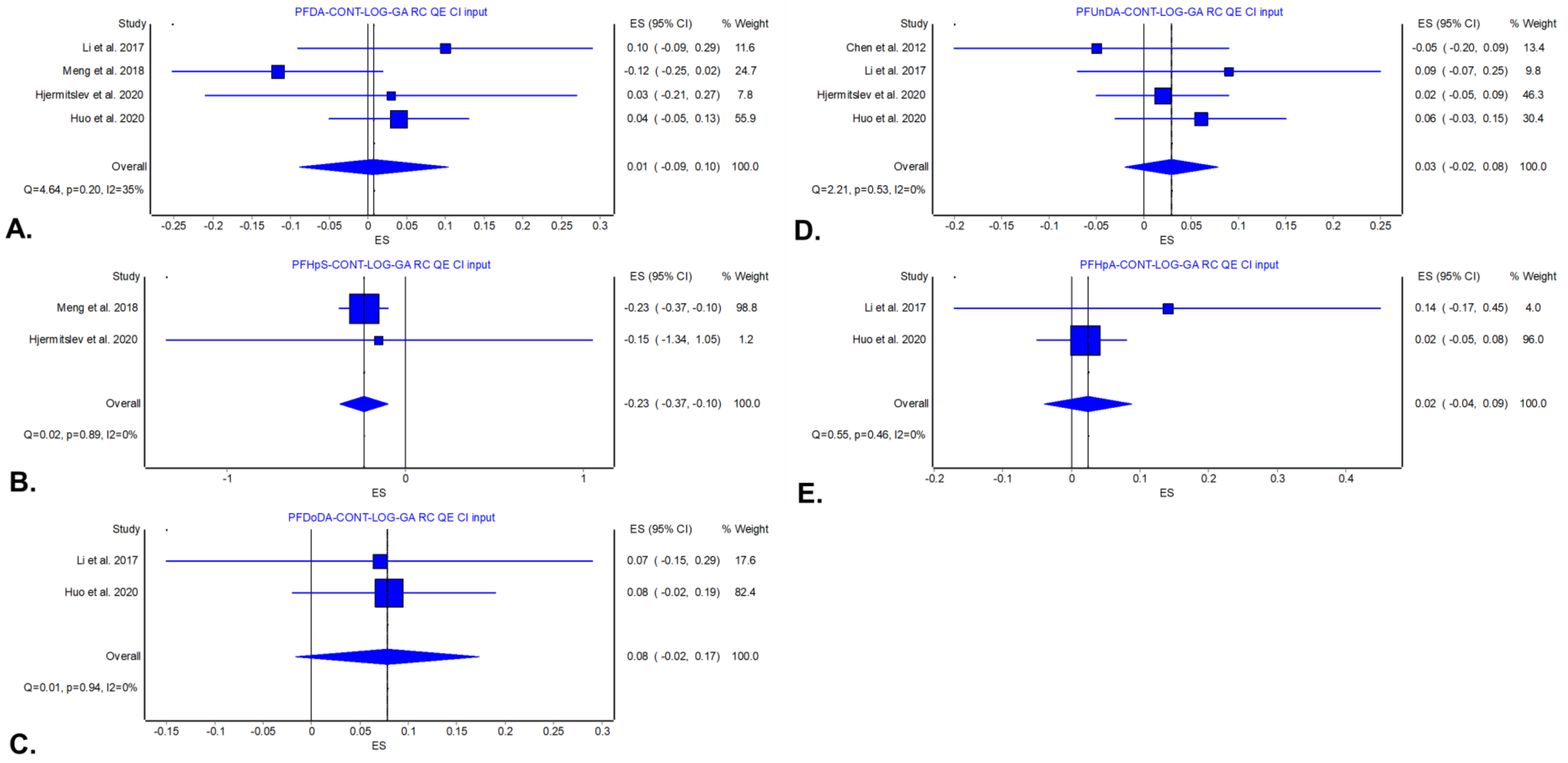

Fig S28. Forest plot on the effect of PFHxS on PI (Ponderal index)/g/cm2 *100 (quality effects model) for (A) per 1 ln(ng/ml) increment , (B) per 1 ng/ml increment and (C) for high versus low categories of exposure.

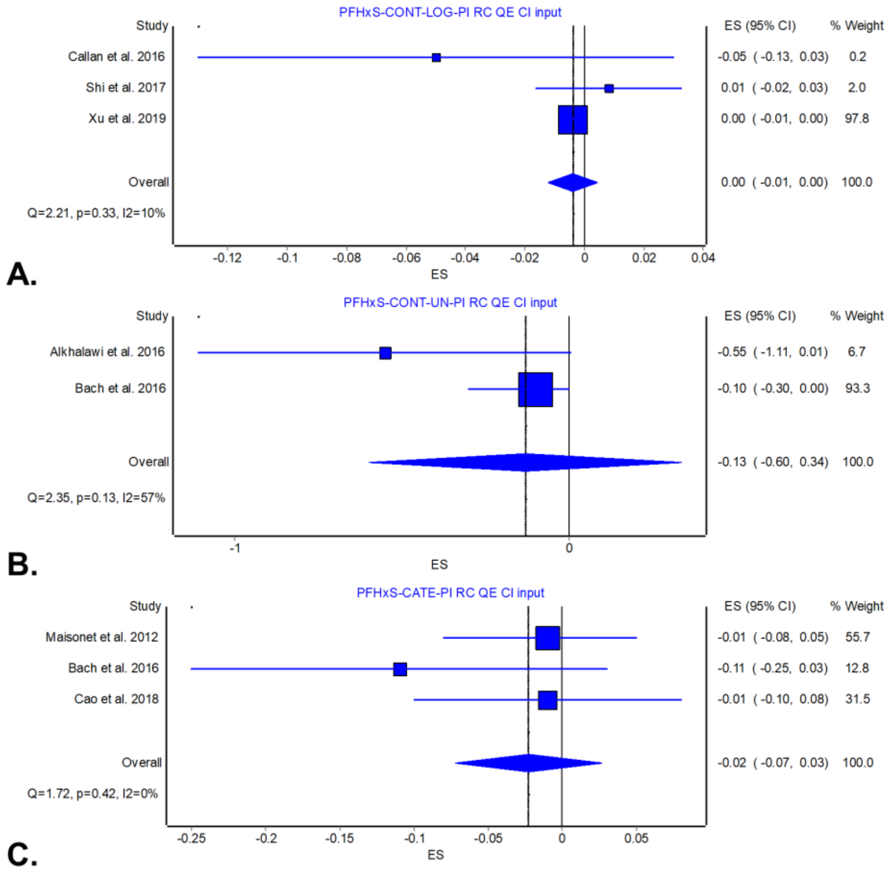

Fig S29. Forest plot on the effect of (a)PFNA (b)PFUnDA (c)PFDA on PI (Ponderal index)/g/cm2 *100 (quality effects model) for per 1 ln(ng/ml) increment and (B) for high versus low categories of exposure.

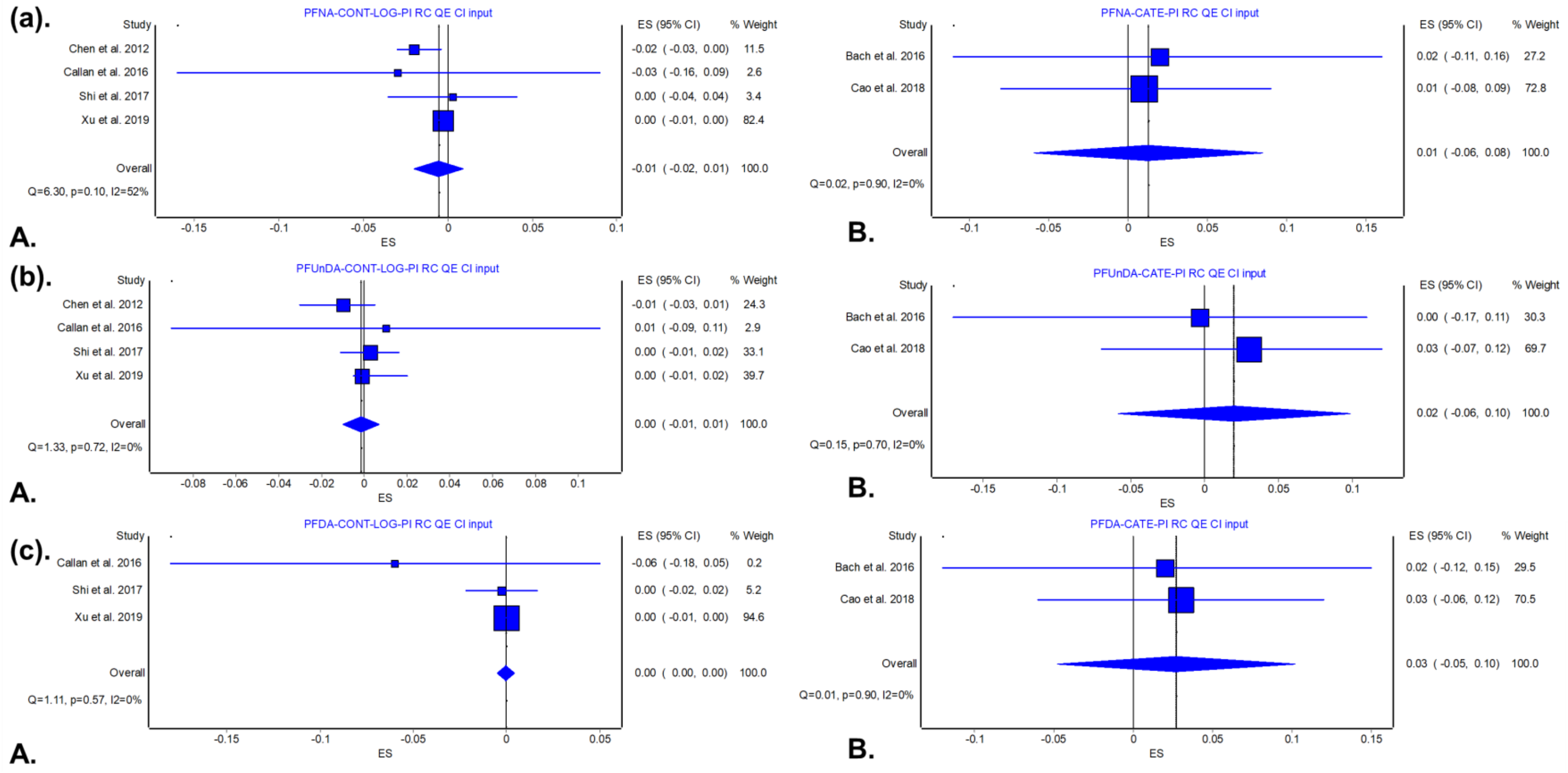

Fig S30. Forest plot on the effect of (a)PFNA (b) PFHxS on PTB (preterm birth) (quality effects model) for per 1 ln(ng/ml) increment and (B) for high versus low categories of exposure.

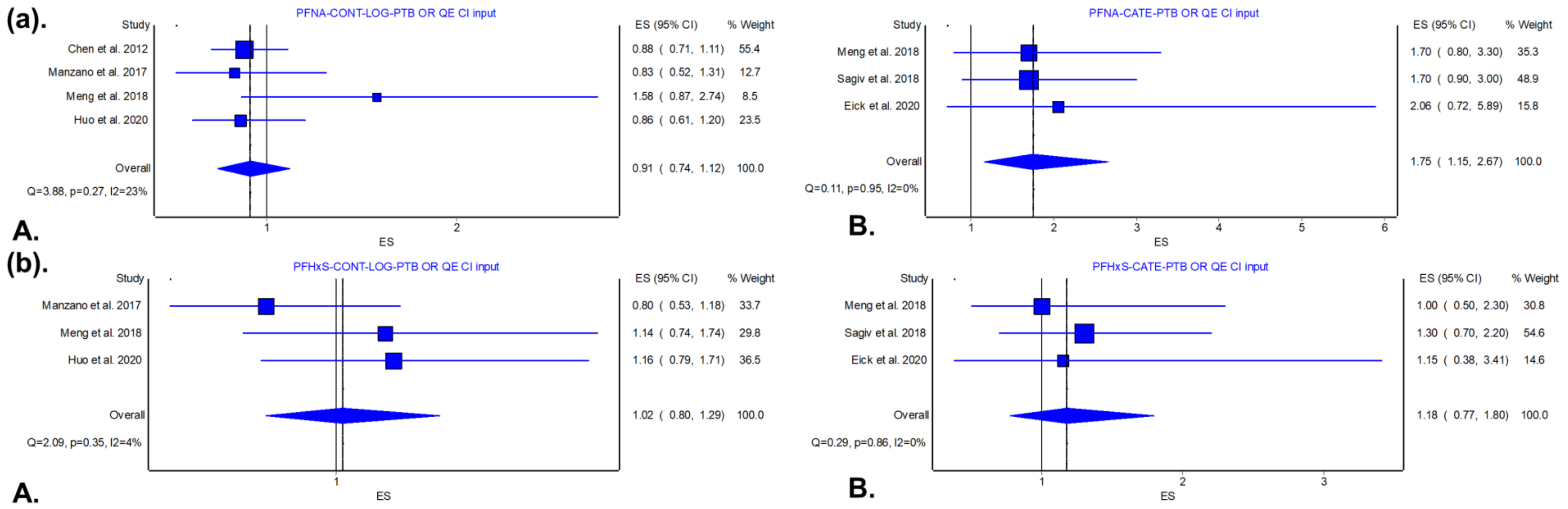

Fig S31. Forest plot on the effect of (a)PFDA (b) PFUnDA on PTB (preterm birth) (quality effects model) for per 1 ln(ng/ml) increment of exposure.

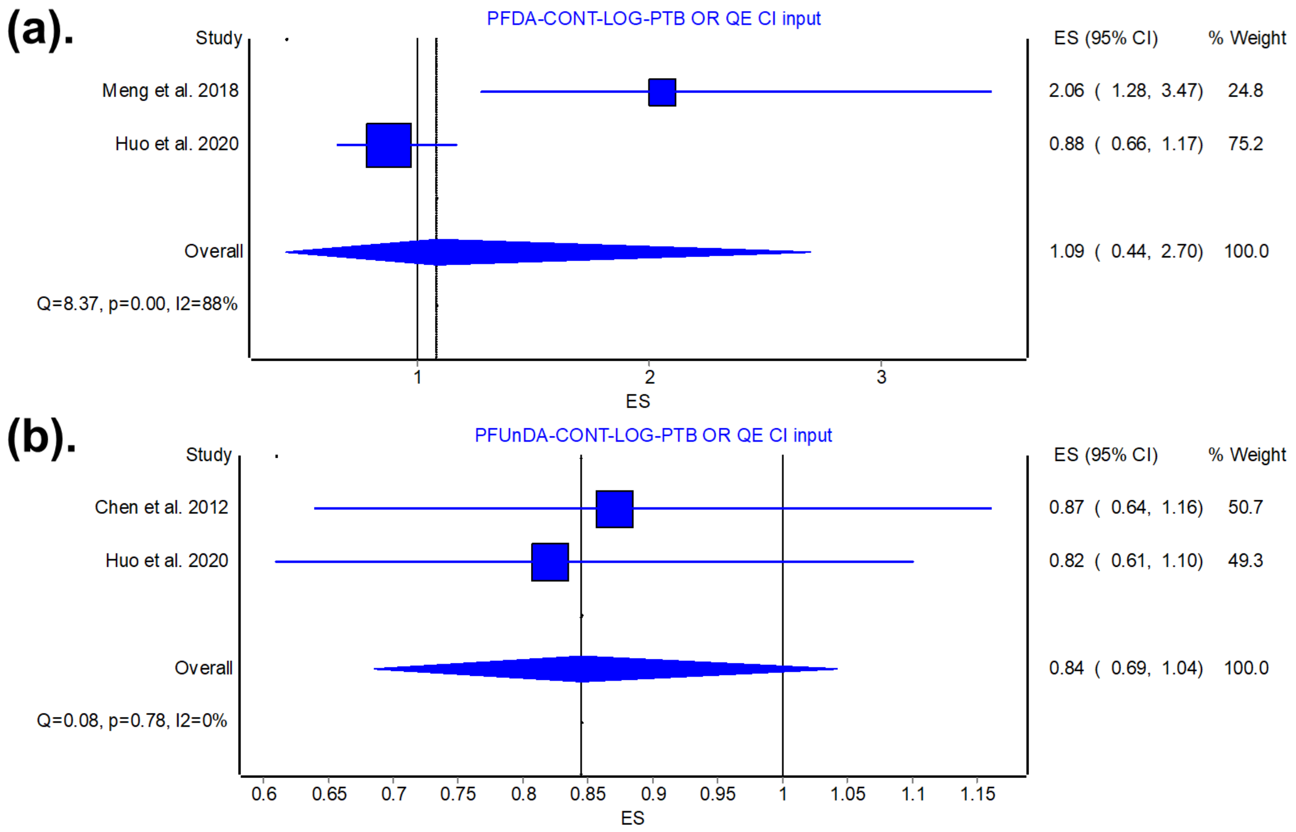

Fig S32. Forest plot on the effect of (a)PFNA (b) PFHxS on LBW (low birth weight) (quality effects model) for per 1 ln(ng/ml) increment of exposure.

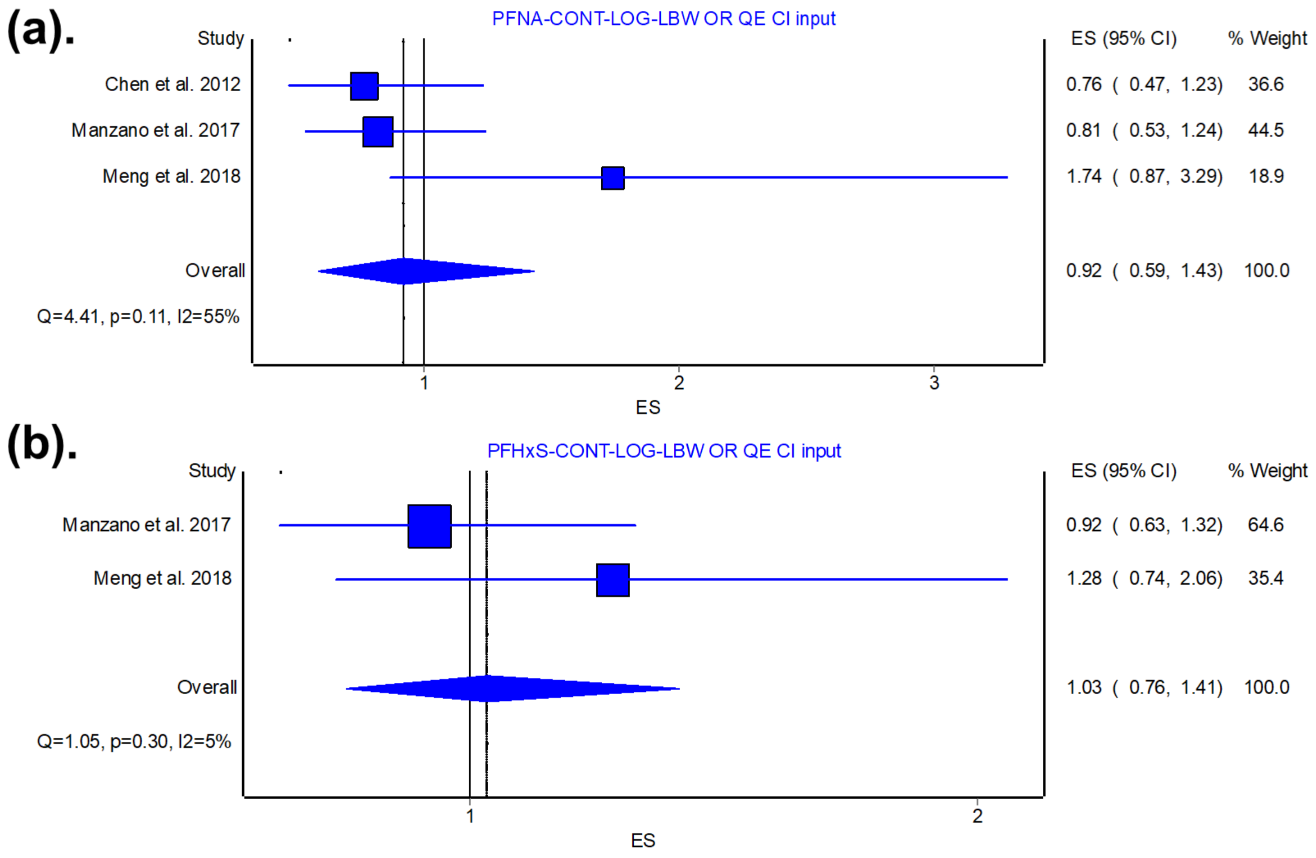

Fig S33. Forest plot on the effect of (a)PFDoDA (b) PFHxS (c)PFDeA (d)PFNA (e)PFUnDA on SGA (small for gestational age) (quality effects model) for per 1 ln(ng/ml) increment of exposure.

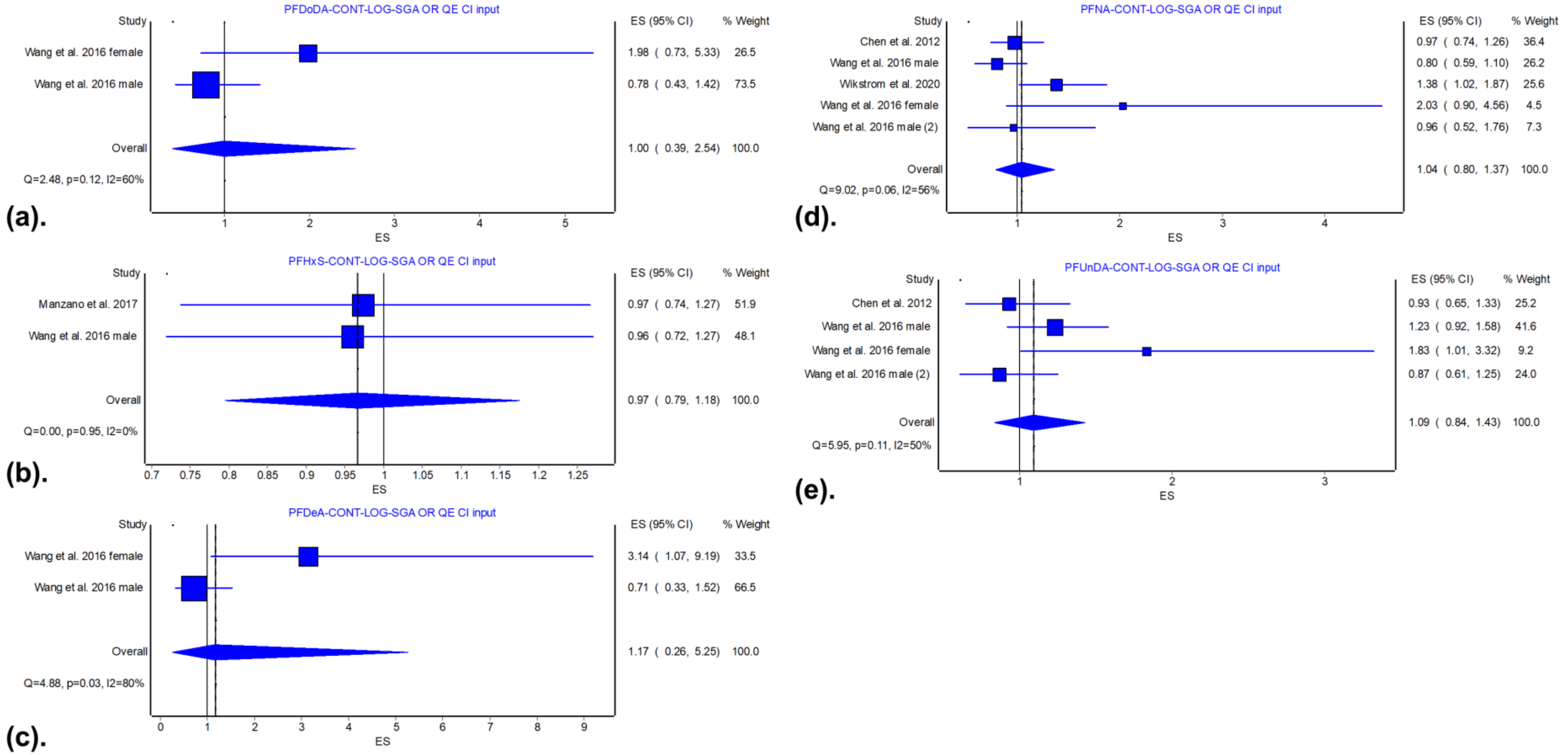

Fig S34. Doi plot and LFK index analysis of publication bias in reported associations between PFOA and BW (birth weight)/g for (A) per 1 ln(ng/ml) increment, (B) per 1 ng/ml increment and (C) for high versus low categories of exposure.

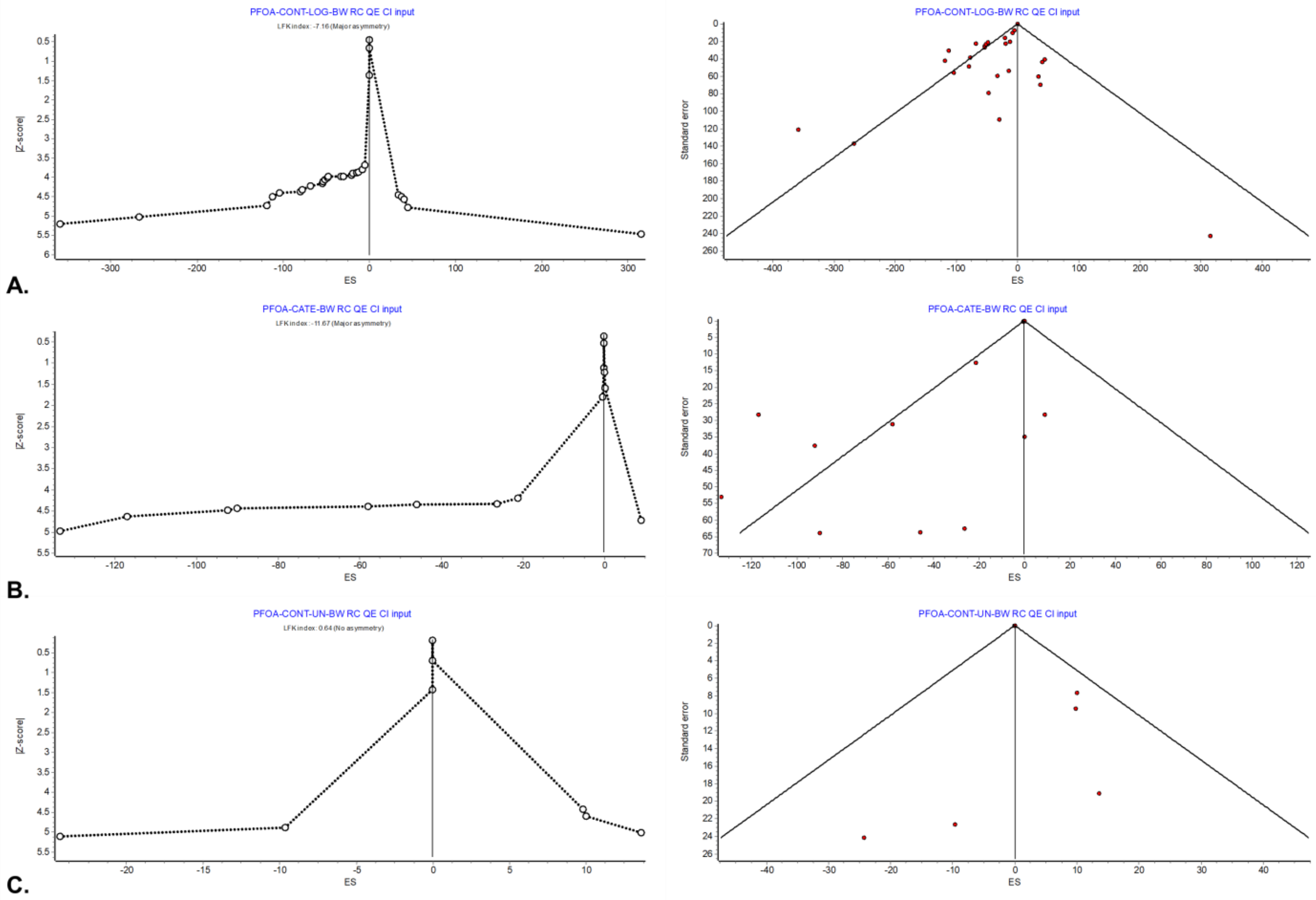

Fig S35. Doi plot and LFK index analysis of publication bias in reported associations between PFOA and BL (birth length)/cm for (A) per 1 ln(ng/ml) increment, (B) per 1 ng/ml increment and (C) for high versus low categories of exposure.

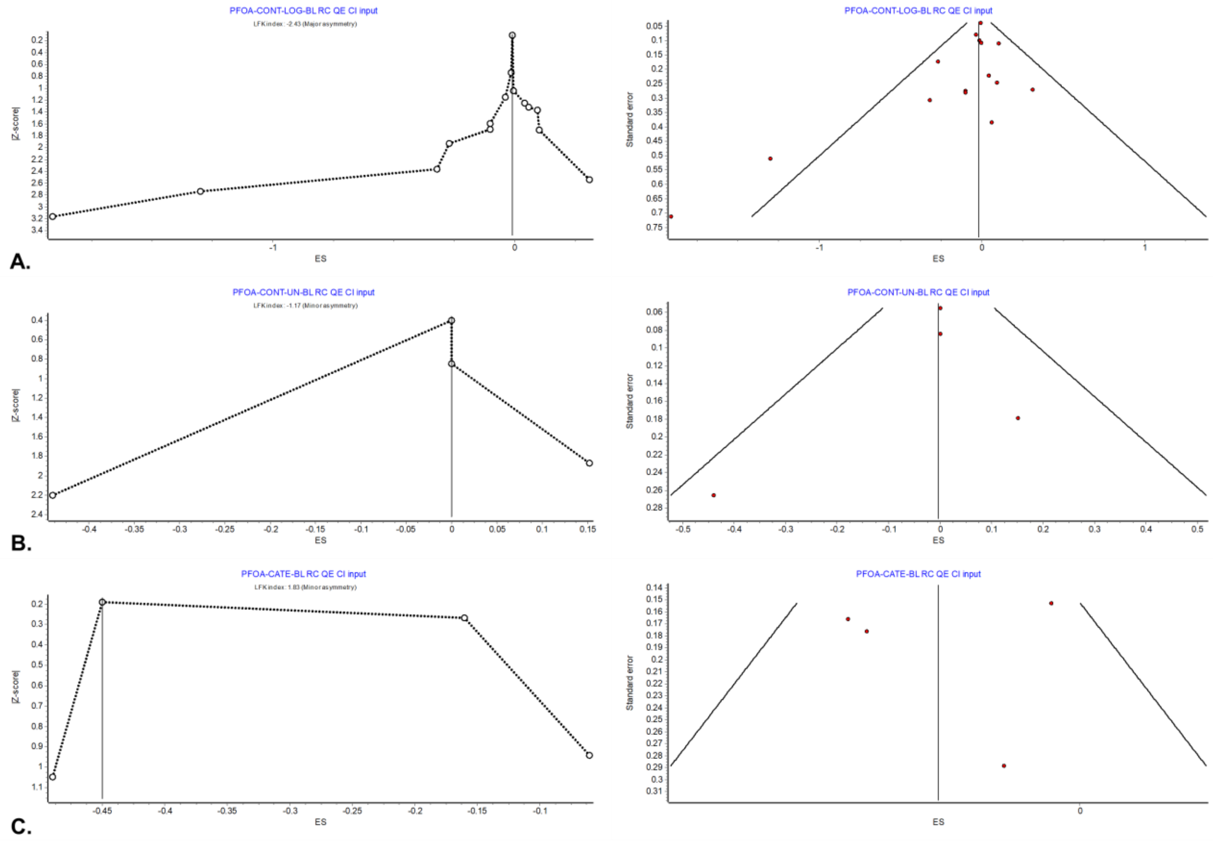

Fig S36. Doi plot and LFK index analysis of publication bias in reported associations between PFOA and HC (head circumference)/cm for (A) per 1 ln(ng/ml) increment, (B) for high versus low categories of exposure.

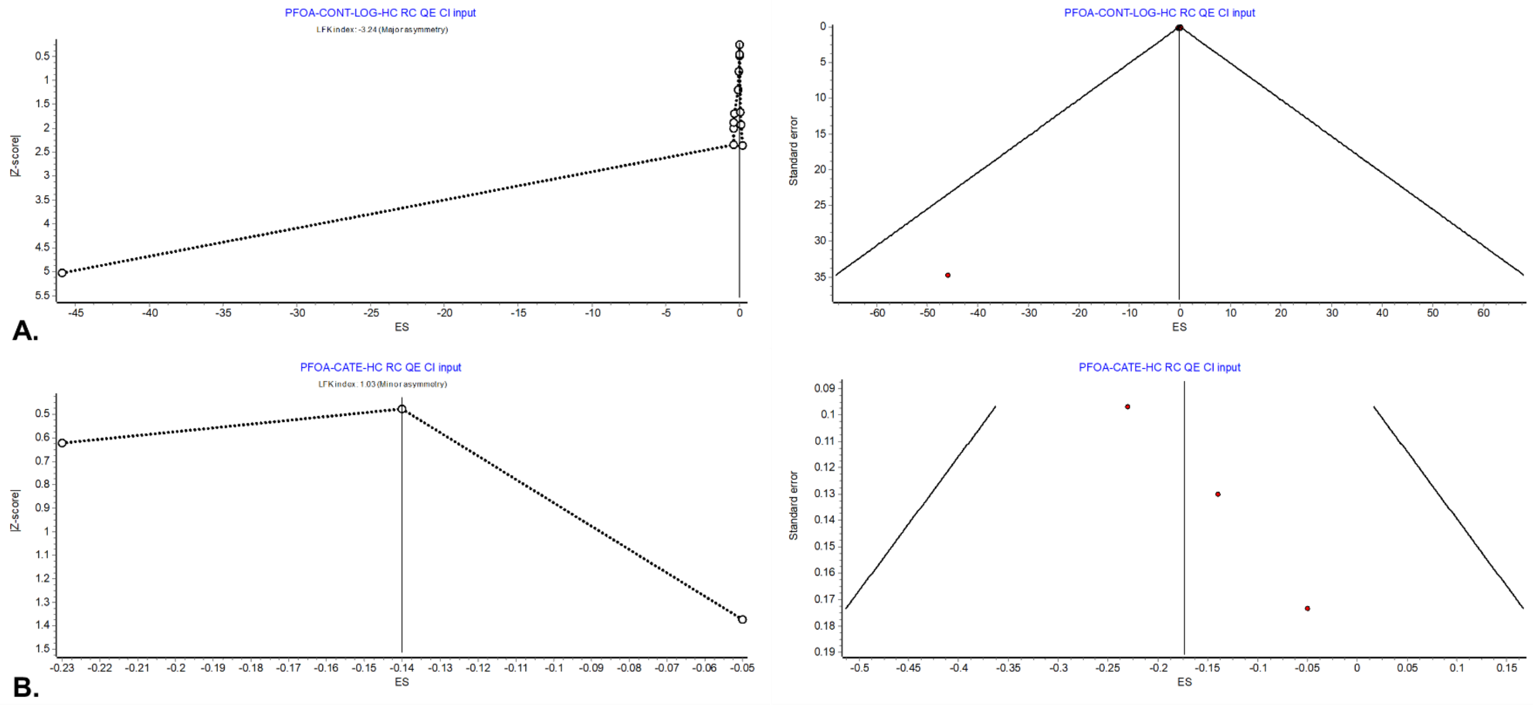

Fig S37. Doi plot and LFK index analysis of publication bias in reported associations between PFOA and GA (gestational age)/weeks for (A) per 1 ln(ng/ml) increment, (B) for high versus low categories of exposure.

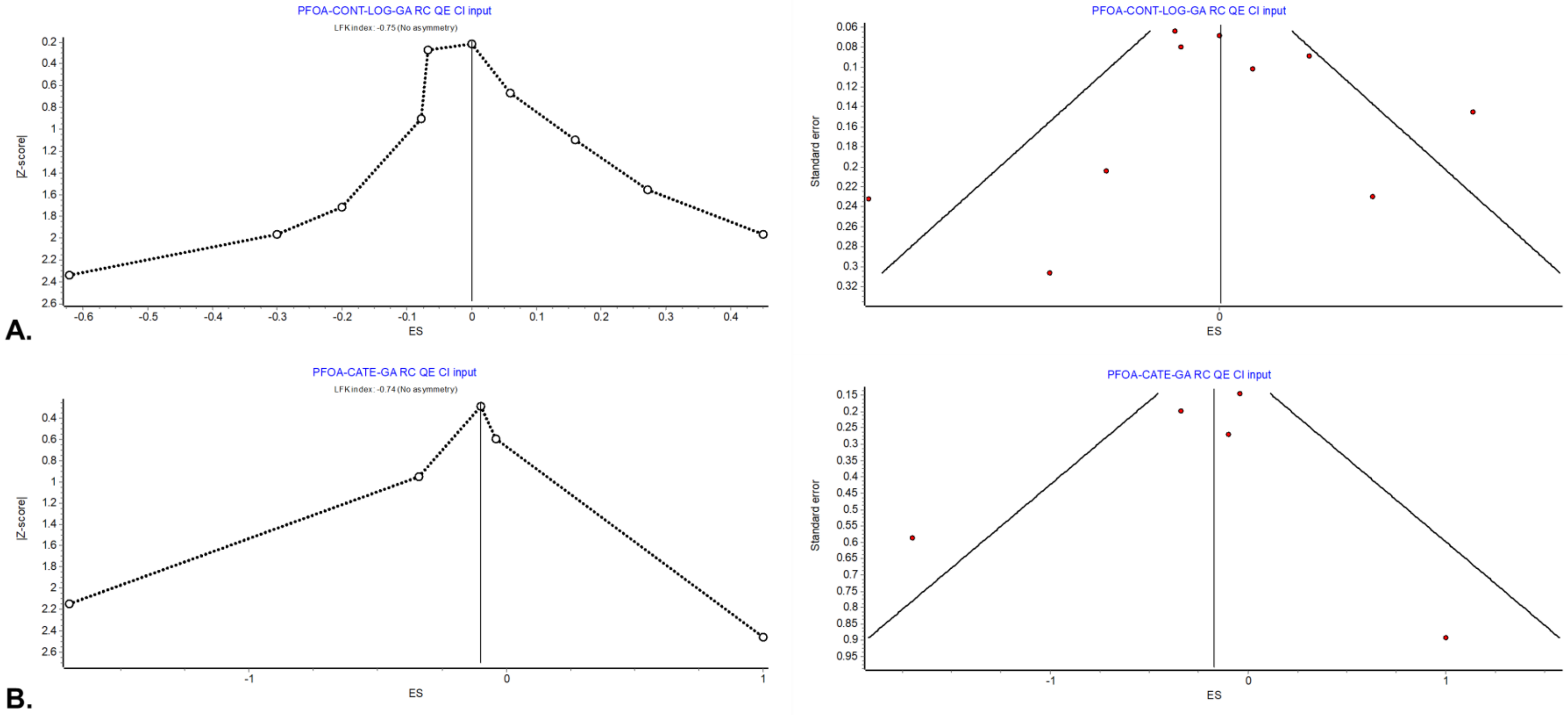

Fig S38. Doi plot and LFK index analysis of publication bias in reported associations between PFOA and PI (Ponderal index)/g/cm2 *100 for (A) per 1 ln(ng/ml) increment, (B) for high versus low categories of exposure.

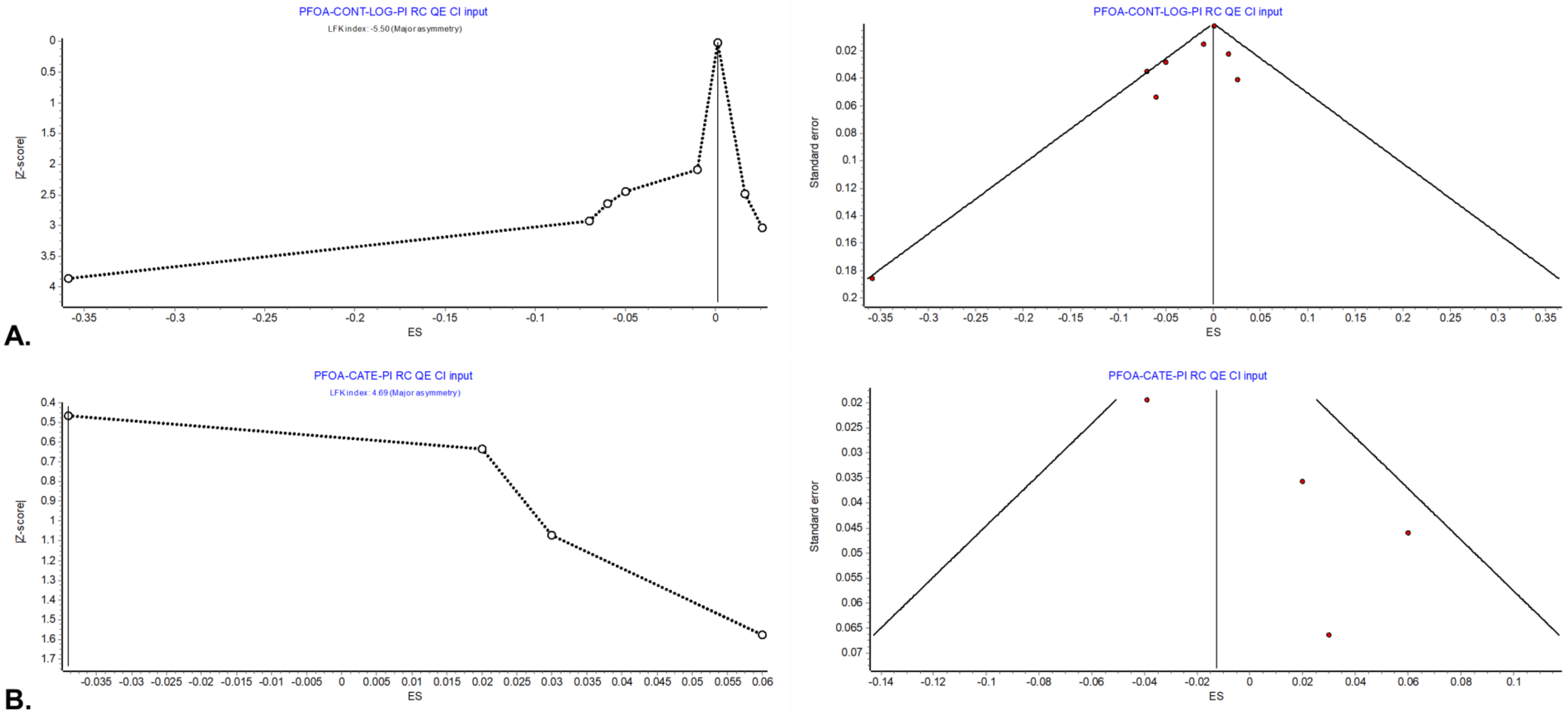

Fig S39. Doi plot and LFK index analysis of publication bias in reported associations between PFOA and PTB (preterm birth) for (A) per 1 ln(ng/ml) increment, (B) for high versus low categories of exposure.

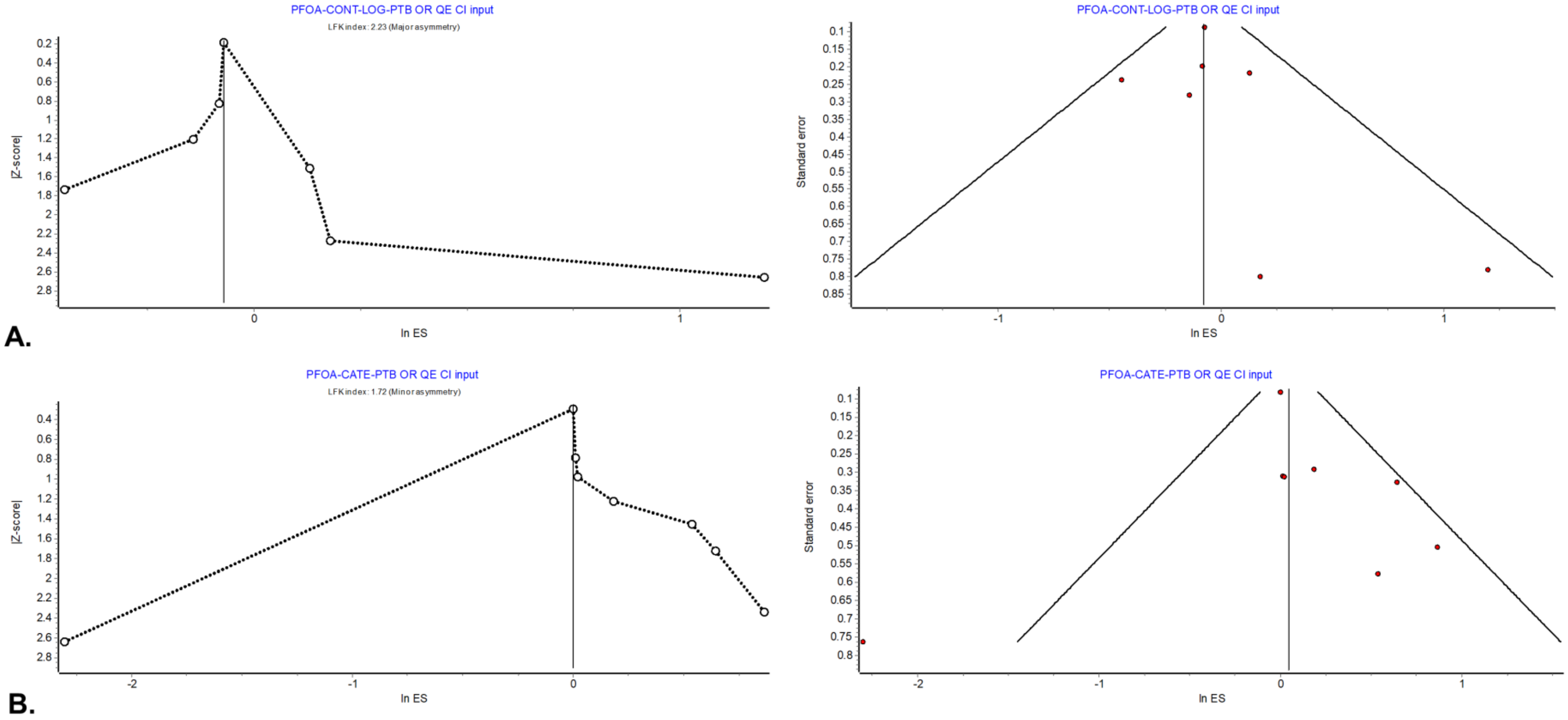

Fig S40. Doi plot and LFK index analysis of publication bias in reported associations between PFOA and LBW (low birth weight) for (A) per 1 ln(ng/ml) increment, (B) for high versus low categories of exposure.

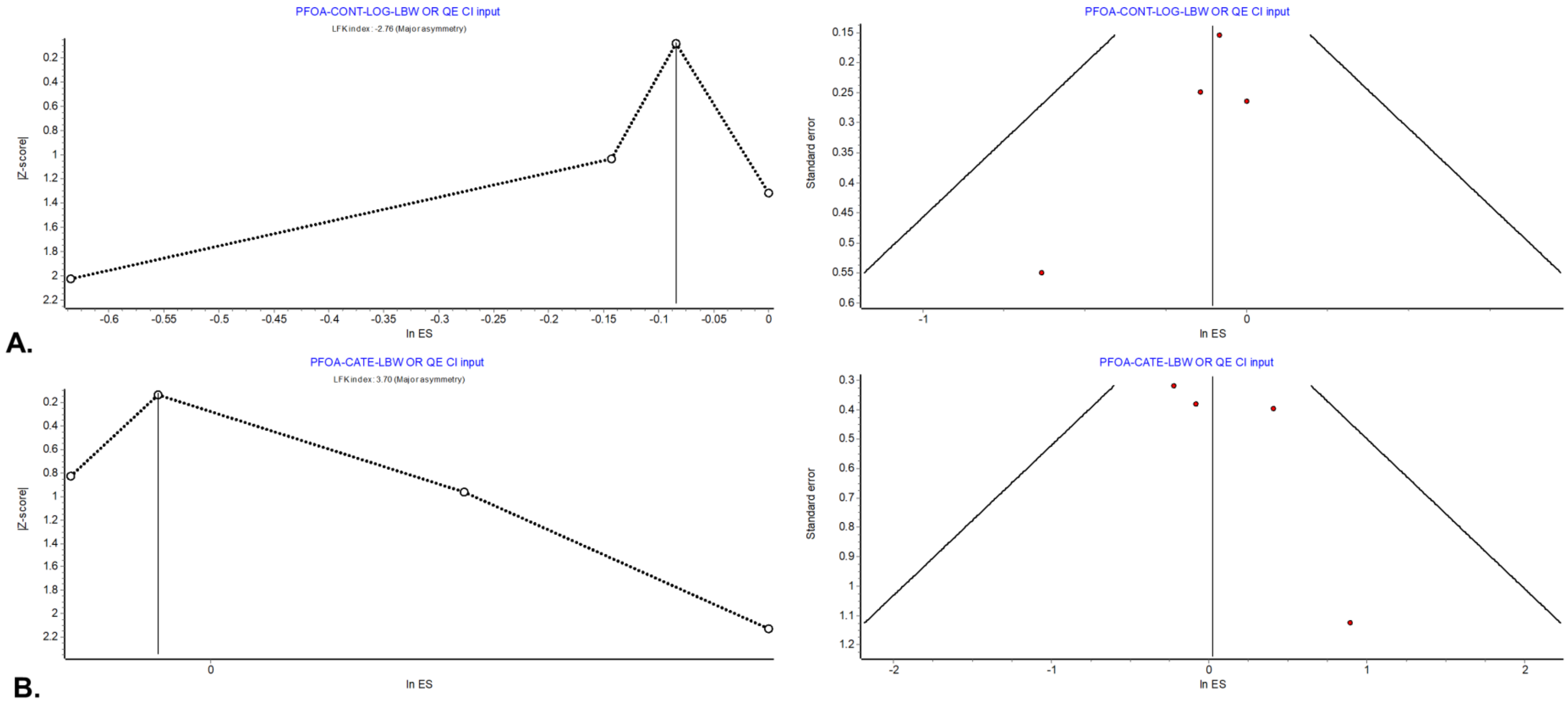

Fig S41. Doi plot and LFK index analysis of publication bias in reported associations between PFOA and SGA (small for gestational age) for (A) per 1 ln(ng/ml) increment, (B) for high versus low categories of exposure.

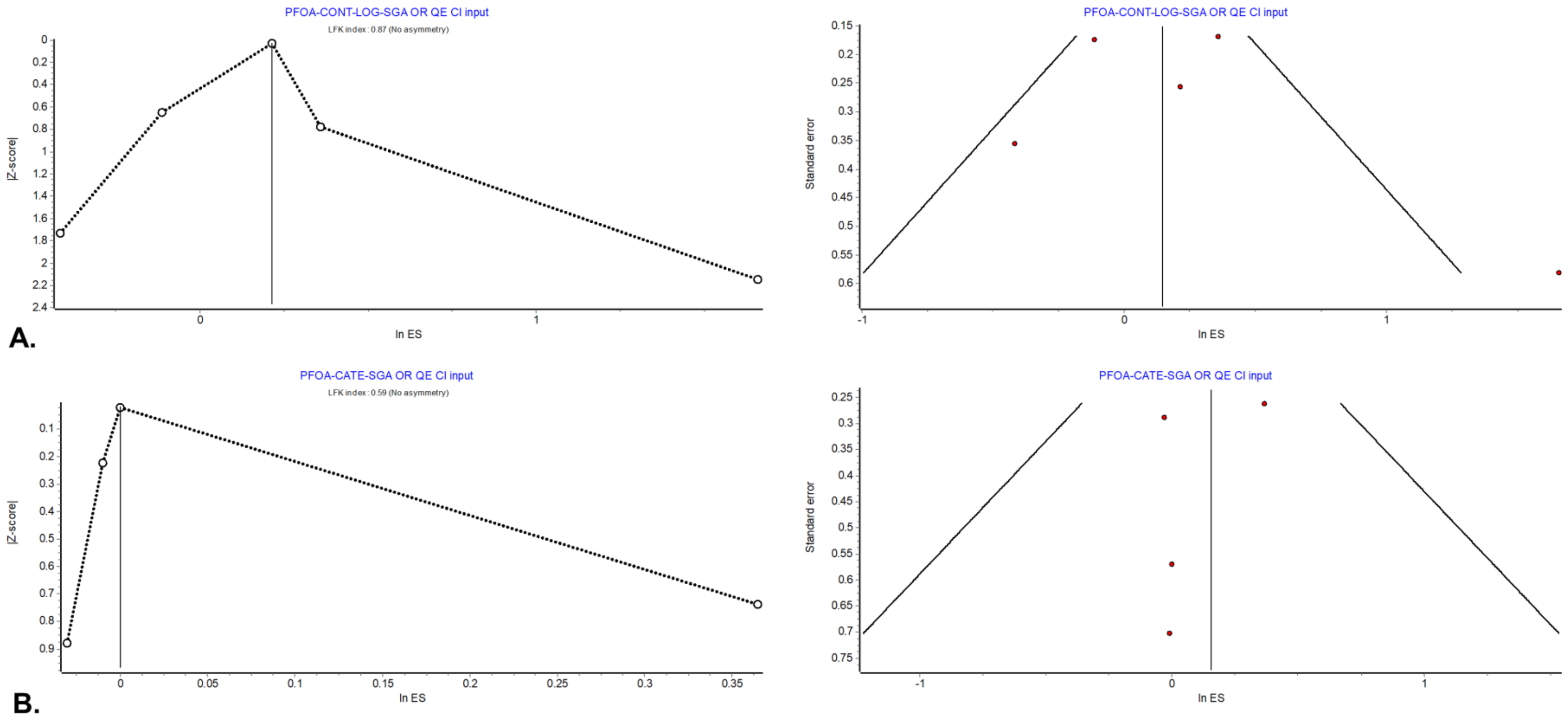

Fig S42. Doi plot and LFK index analysis of publication bias in reported associations between PFOS and BW (birth weight)/g for (A) per 1 ln(ng/ml) increment, (B) for high versus low categories of exposure.

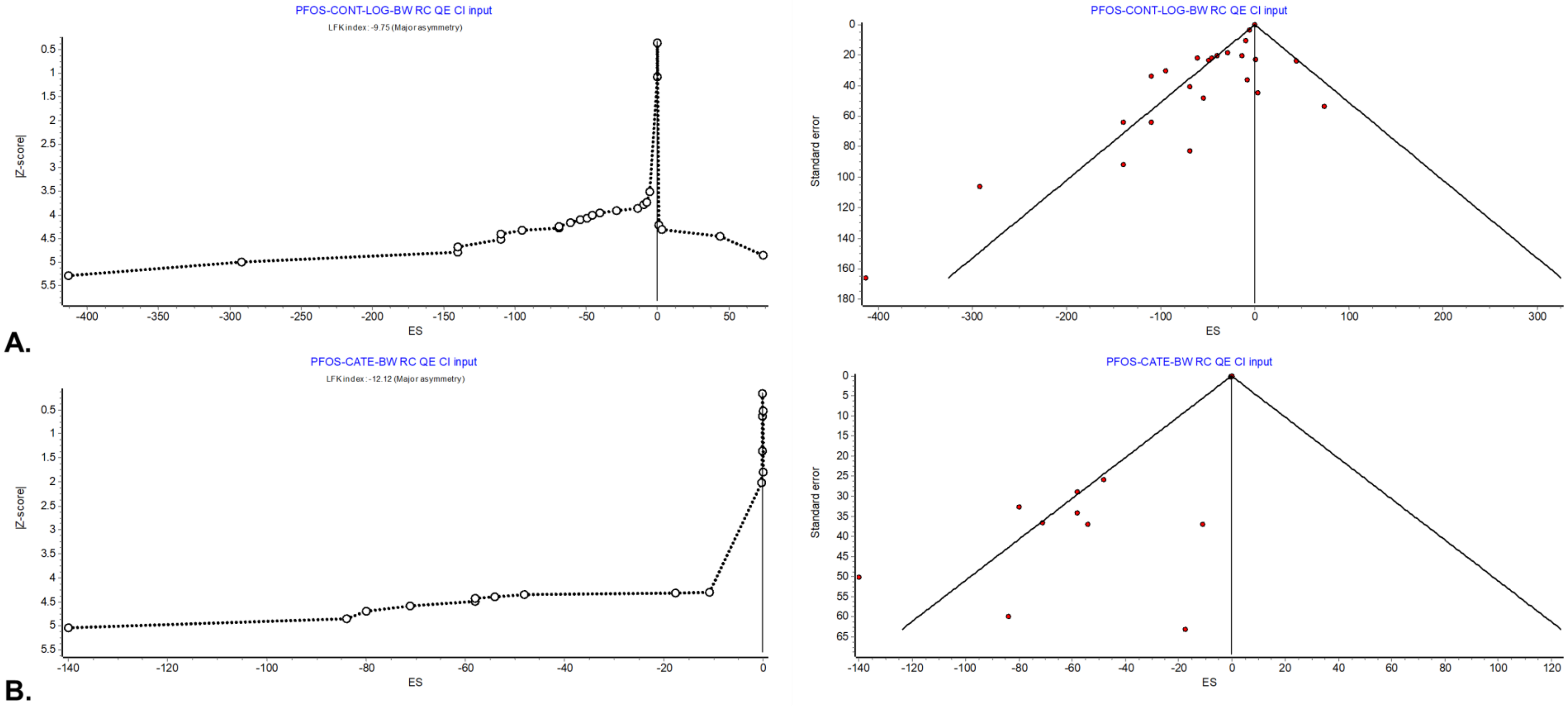

Fig S43. Doi plot and LFK index analysis of publication bias in reported associations between PFOS and BL (birth length)/cm for (A) per 1 ln(ng/ml) increment, (B) per 1 ng/ml increment and (C) for high versus low categories of exposure.

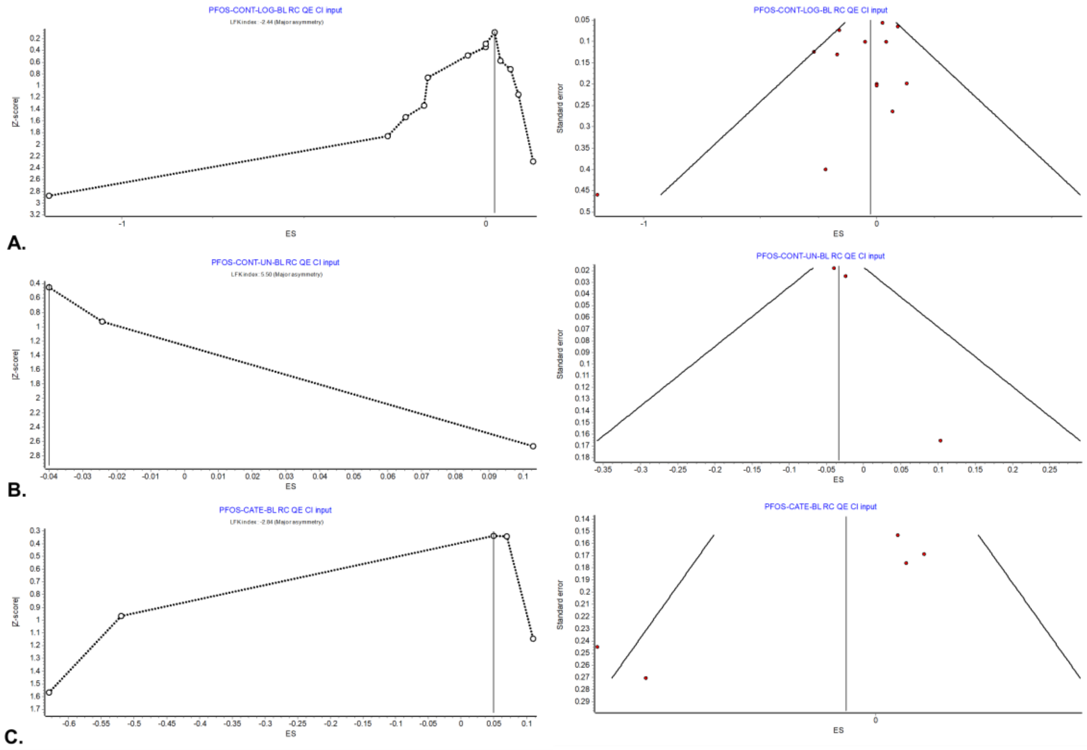

Fig S44. Doi plot and LFK index analysis of publication bias in reported associations between PFOS and HC (head circumference)/cm for (A) per 1 ln(ng/ml) increment, (B) for high versus low categories of exposure.

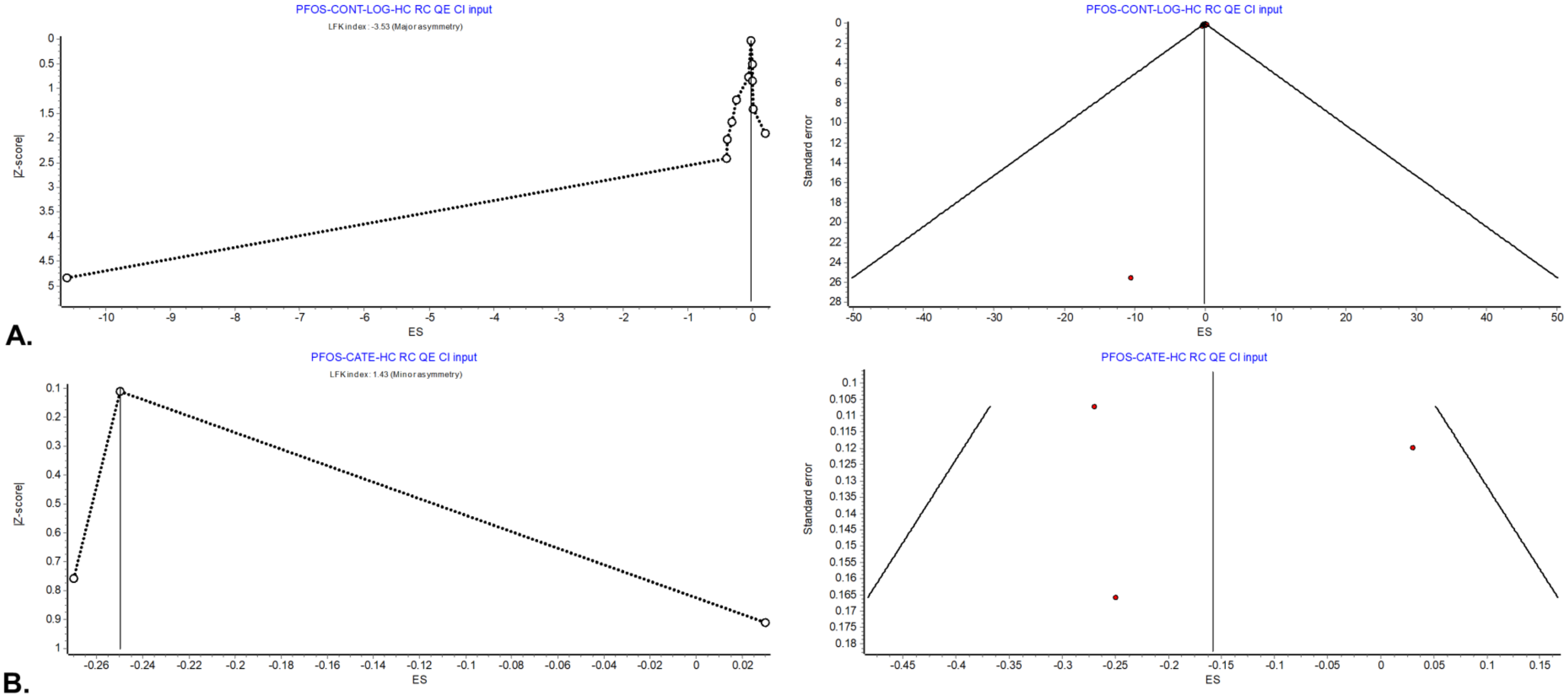

Fig S45. Doi plot and LFK index analysis of publication bias in reported associations between PFOS and GA (gestational age)/weeks for (A) per 1 ln(ng/ml) increment, (B) for high versus low categories of exposure.

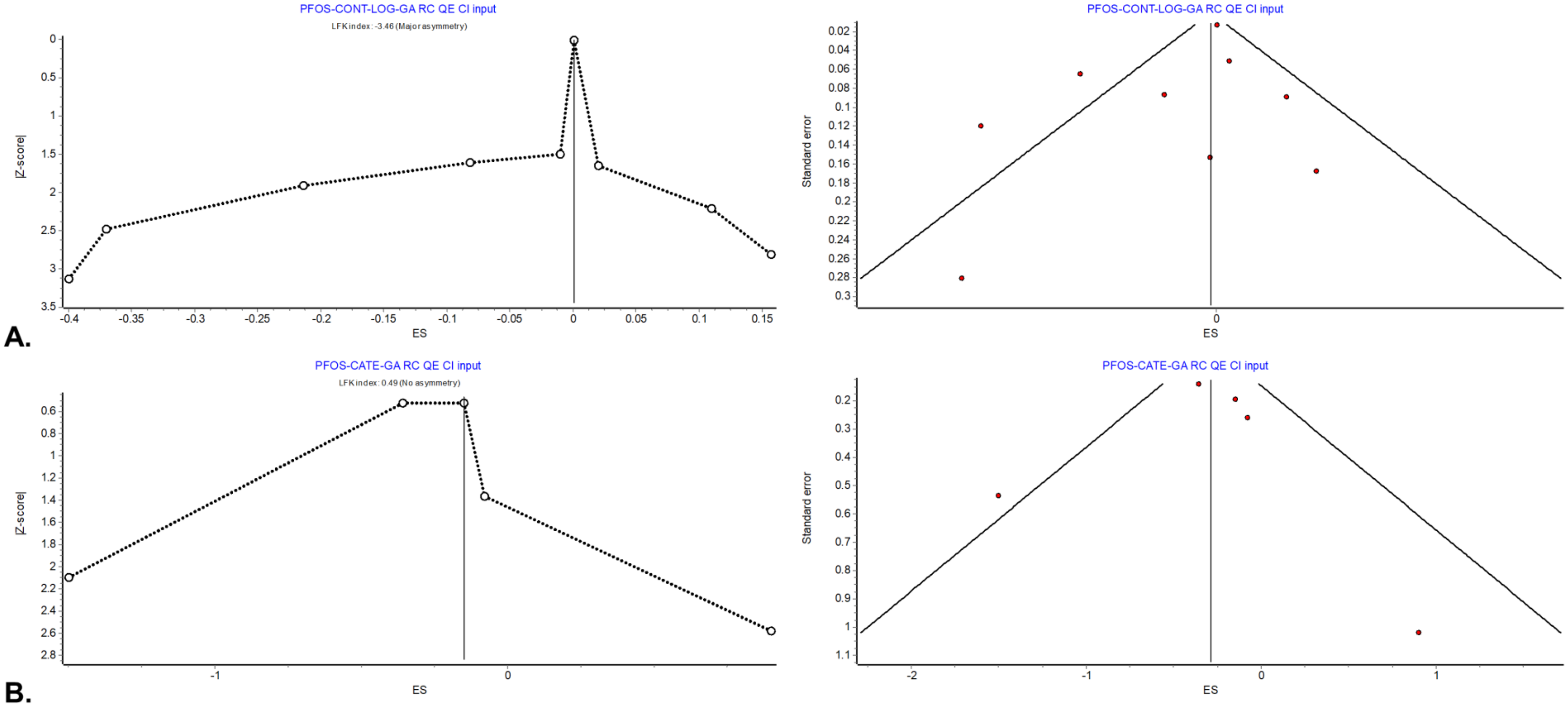

Fig S46. Doi plot and LFK index analysis of publication bias in reported associations between PFOS and PI (Ponderal index)/g/cm2 *100 for (A) per 1 ln(ng/ml) increment, (B) for high versus low categories of exposure.

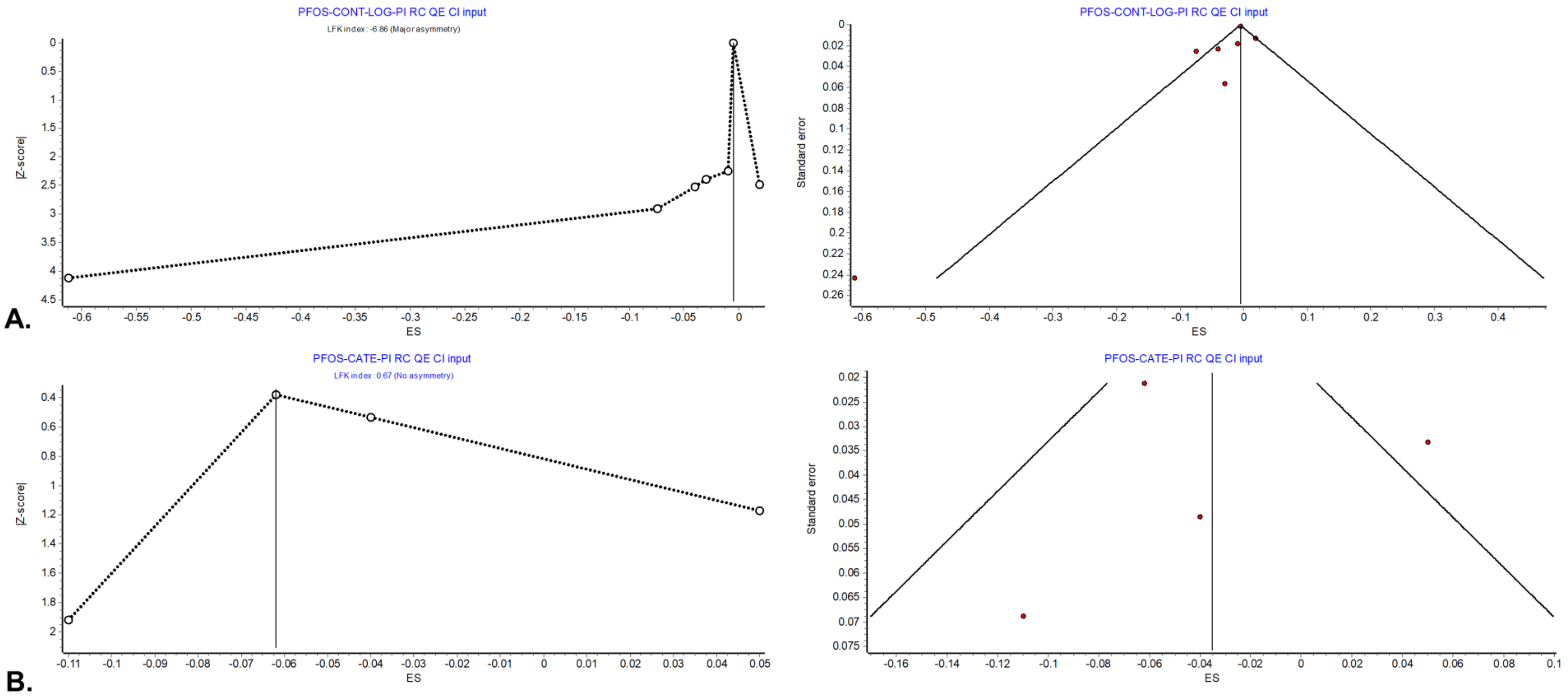

Fig S47. Doi plot and LFK index analysis of publication bias in reported associations between PFOS and PTB (preterm birth) for (A) per 1 ln(ng/ml) increment, (B) for high versus low categories of exposure.

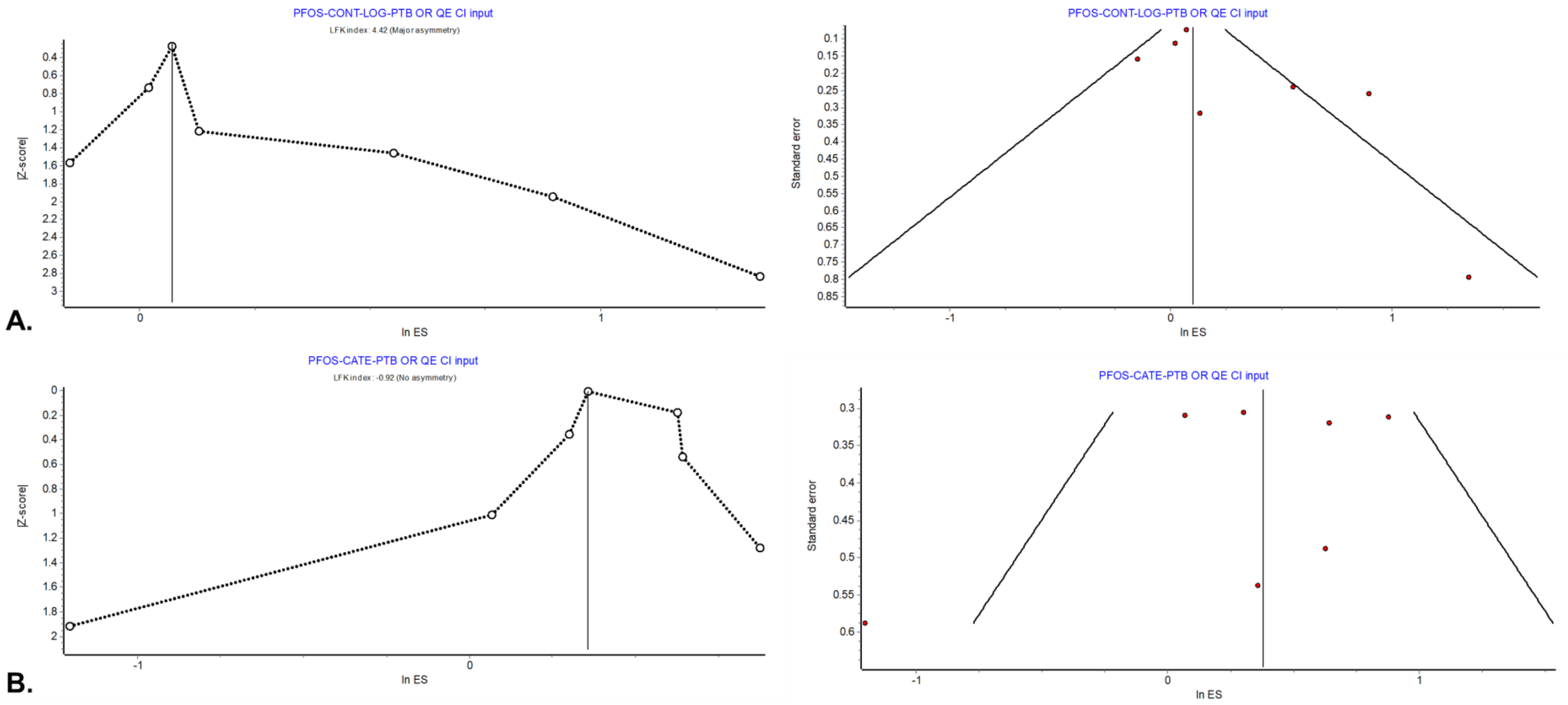

Fig S48. Doi plot and LFK index analysis of publication bias in reported associations between PFOS and LBW (low birth weight) for (A) per 1 ln(ng/ml) increment, (B) for high versus low categories of exposure.

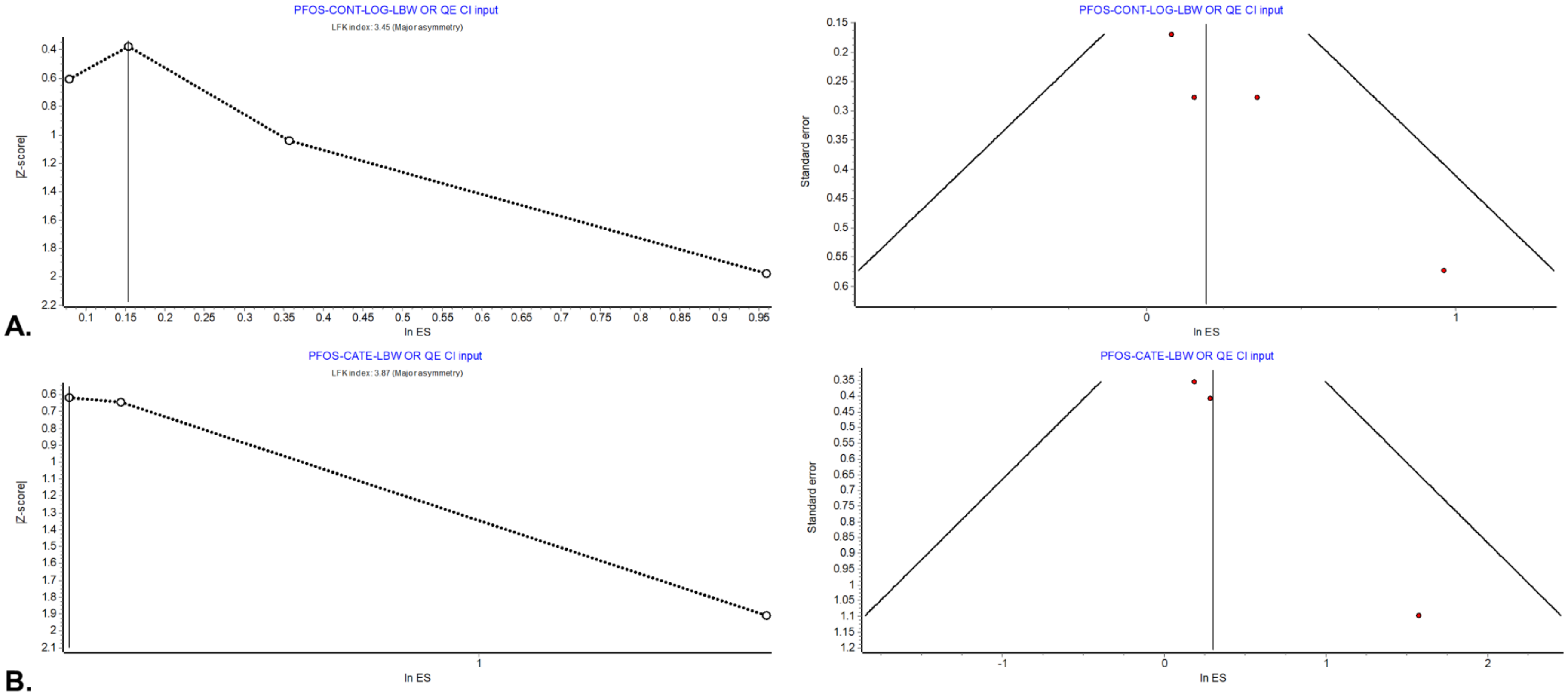

Fig S49. Doi plot and LFK index analysis of publication bias in reported associations between PFOS and SGA (small for gestational age) for (A) per 1 ln(ng/ml) increment, (B) for high versus low categories of exposure.

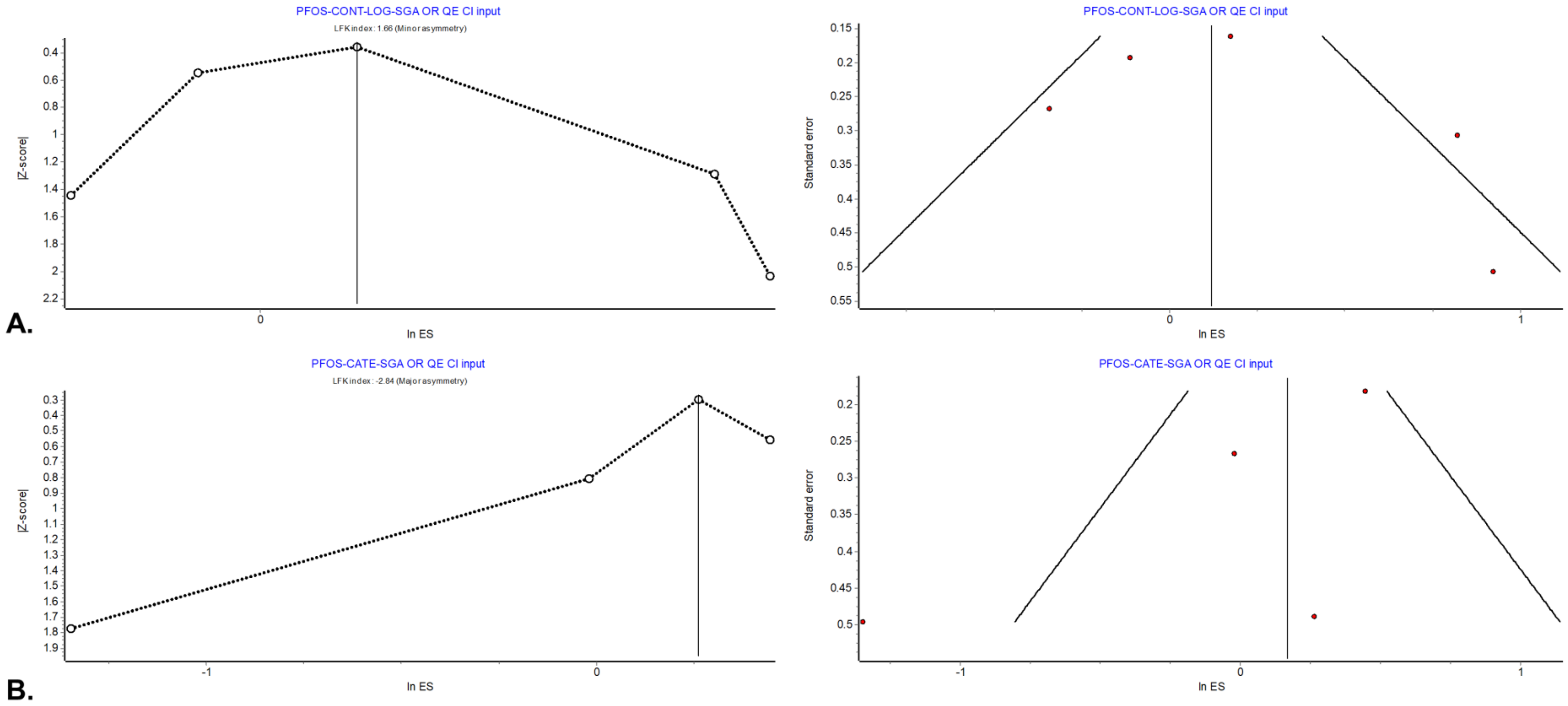

Fig S50. Doi plot and LFK index analysis of publication bias in reported associations between PFDA on BW (birth weight)/g for (A) per 1 ln(ng/ml) increment, (B) for high versus low categories of exposure.

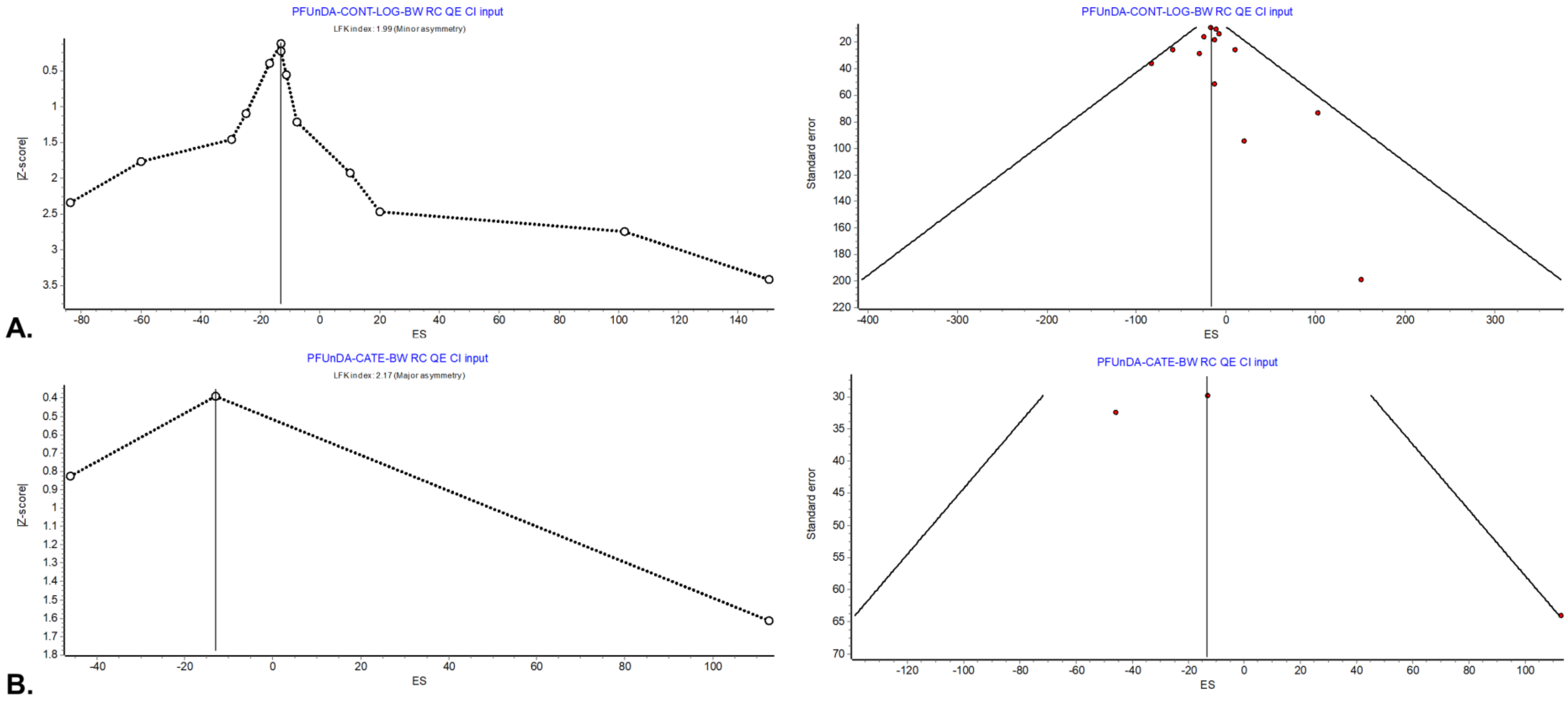

Fig S51. Doi plot and LFK index analysis of publication bias in reported associations between PFHxS on BW (birth weight)/g for (A) per 1 ln(ng/ml) increment, (B) for high versus low categories of exposure.

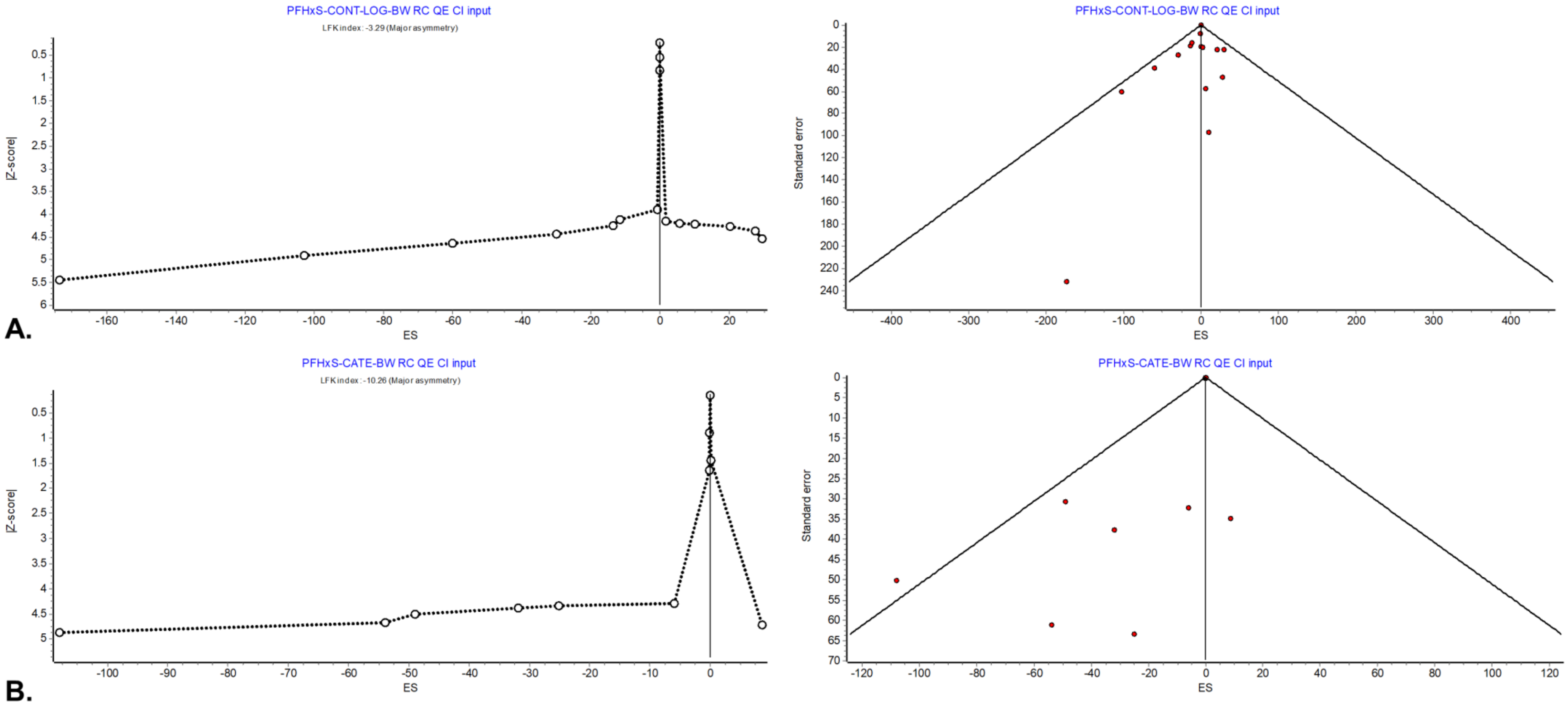

Fig S52. Doi plot and LFK index analysis of publication bias in reported associations between PFNA on BW (birth weight)/g for (A) per 1 ln(ng/ml) increment, (B) per 1 ng/ml increment and (C) for high versus low categories of exposure.

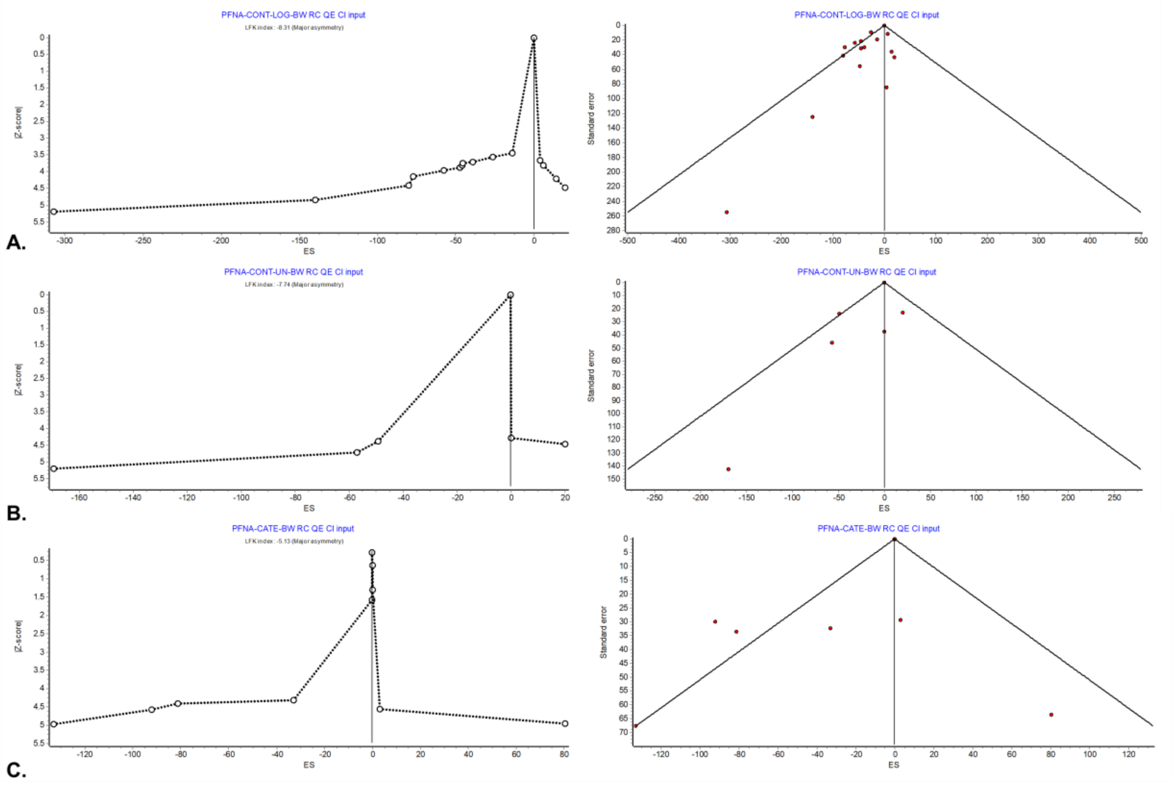

Fig S53. Doi plot and LFK index analysis of publication bias in reported associations between PFDA on BW (birth weight)/g for high versus low categories of exposure.

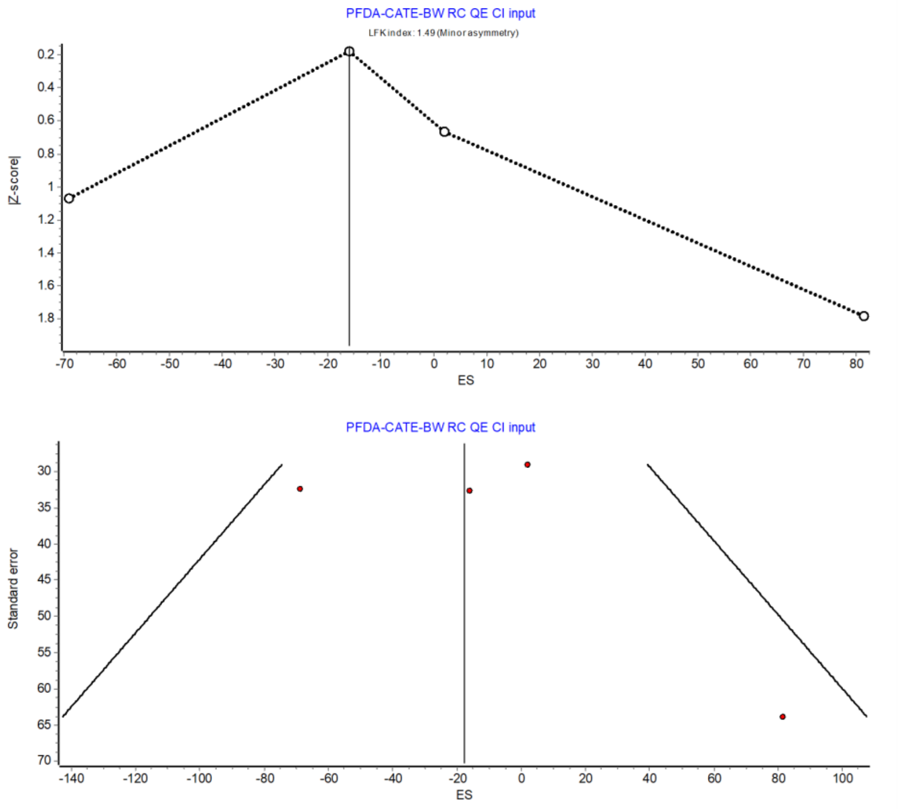

Fig S54. Doi plot and LFK index analysis of publication bias in reported associations between (A)PFDoDA (B)PFHpA (C)PFDeA on BW (birth weight)/g (quality effects model) for per 1 ln(ng/ml) increment of exposure.

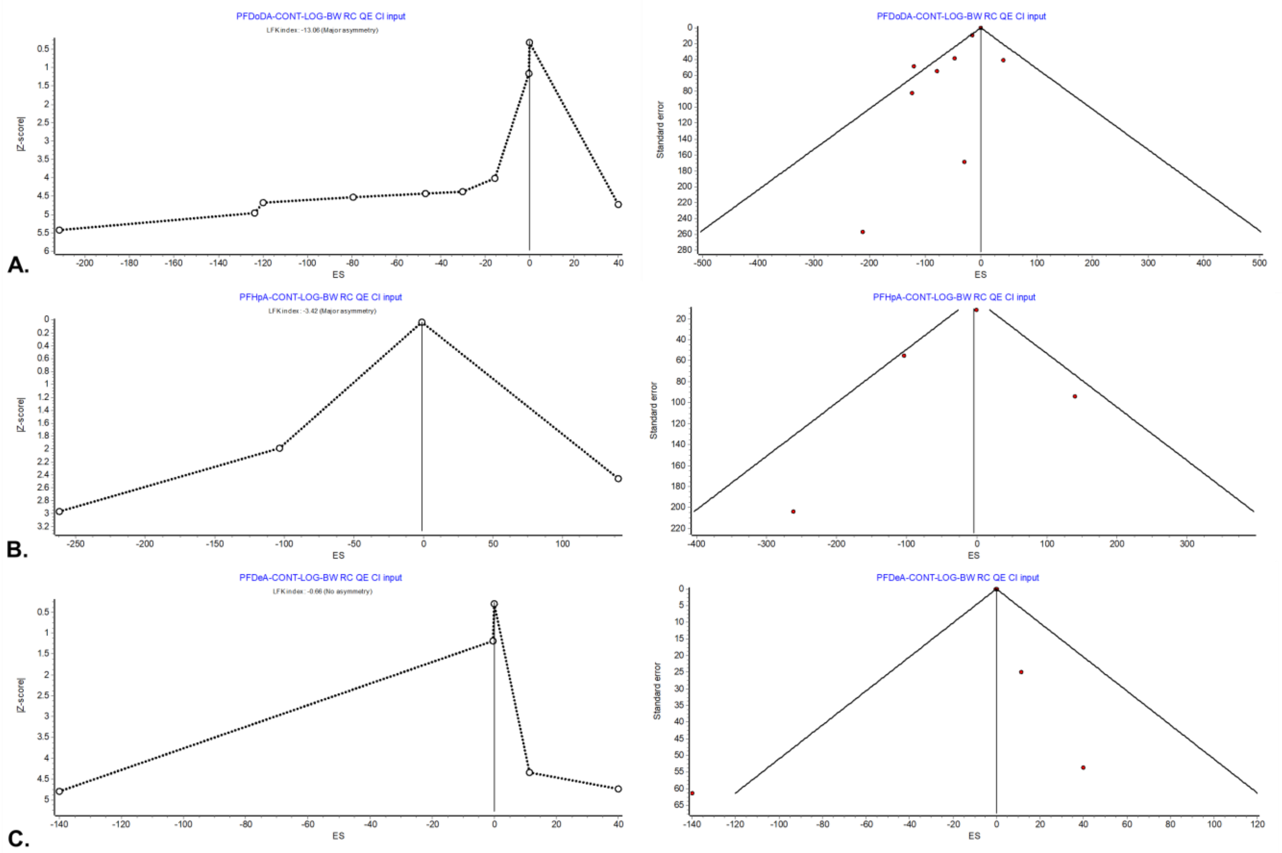

Fig S55. Doi plot and LFK index analysis of publication bias in reported associations between (a)PFNA (b)PFHxS on BL (birth length)/cm for (A) per 1 ln(ng/ml) increment , (B) per 1 ng/ml increment and (C) for high versus low categories of exposure.

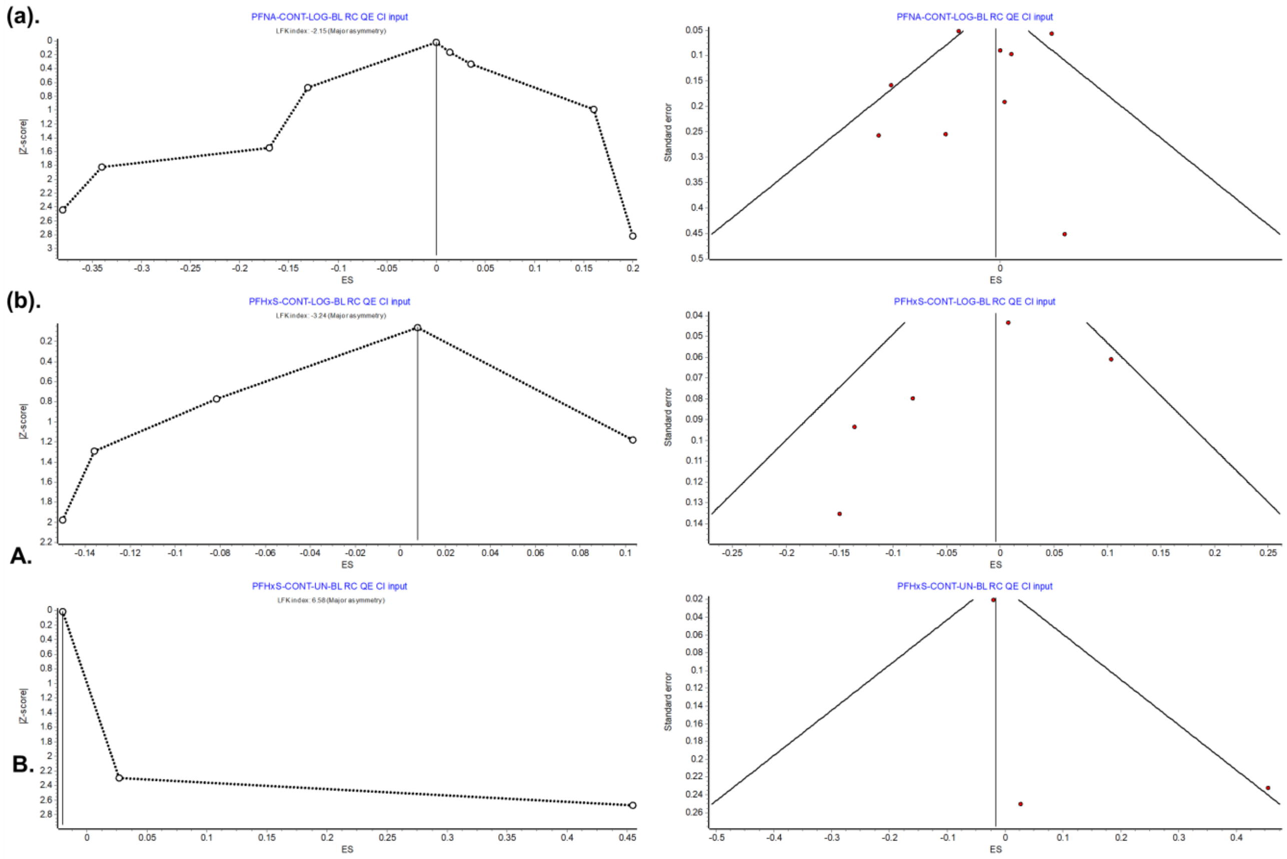

Fig S56. Doi plot and LFK index analysis of publication bias in reported associations between(A)PFDA (B)PFDoDA (C)PFUnDA on BL (birth length)/cm (quality effects model) for per 1 ln(ng/ml) increment of exposure.

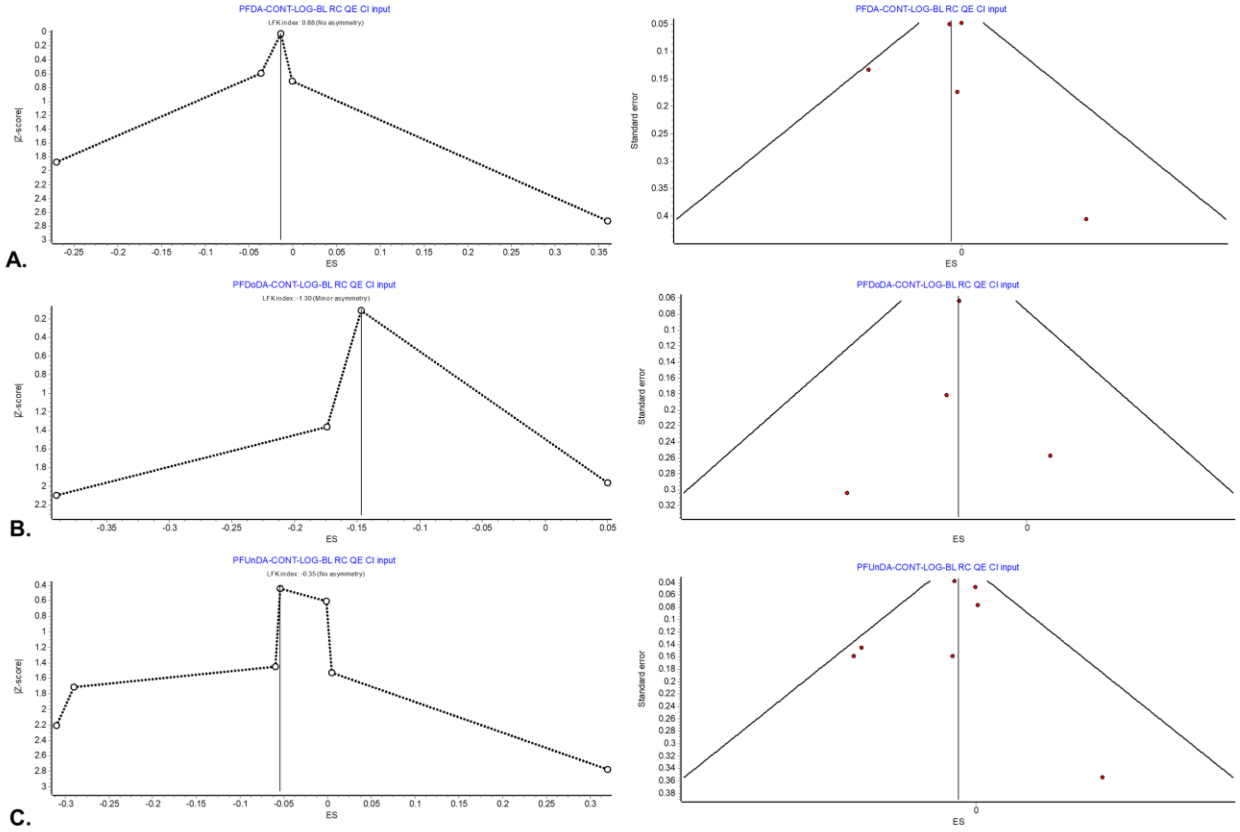

Fig S57. Doi plot and LFK index analysis of publication bias in reported associations between (a)PFNA (b)PFHxS on HC (head circumference)/cm for per 1 ln(ng/ml) increment of exposure.

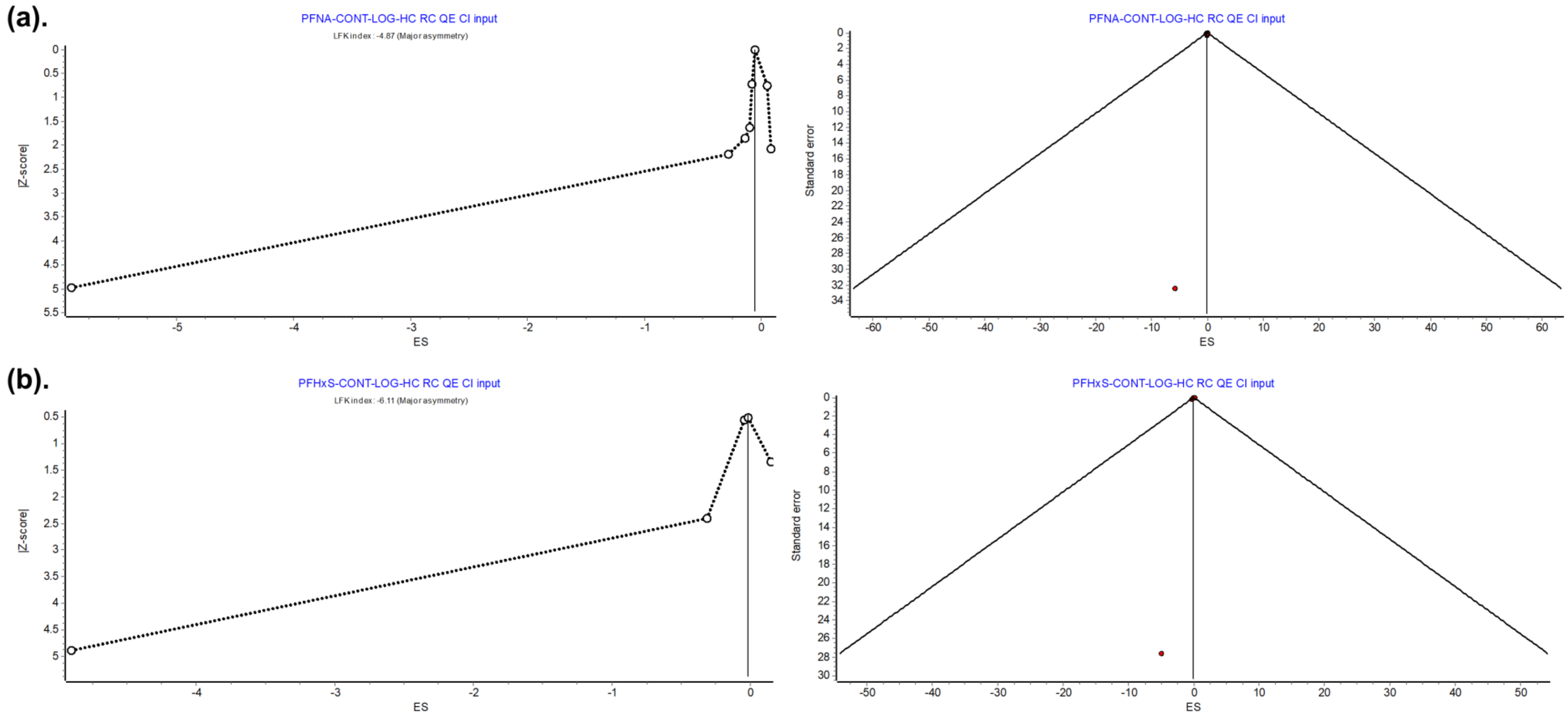

Fig S58. Doi plot and LFK index analysis of publication bias in reported associations between(A)PFDA (B)PFDoDA (C)PFUnDA on HC (head circumference)/cm for per 1 ln(ng/ml) increment of exposure.

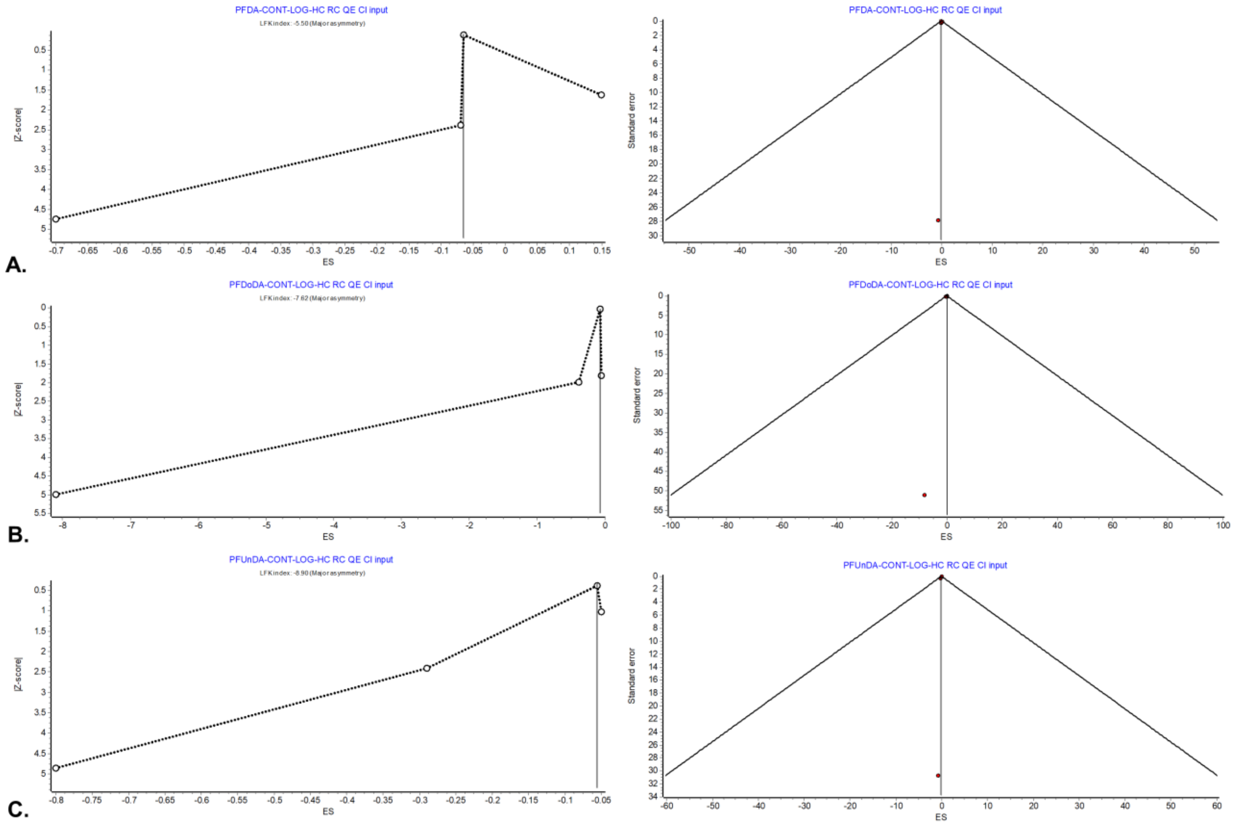

Fig S59. Doi plot and LFK index analysis of publication bias in reported associations between (a)PFNA (b)PFHxS on GA (gestational age)/weeks for (A) per 1 ln(ng/ml) increment and (B) per 1 ng/ml increment of exposure.

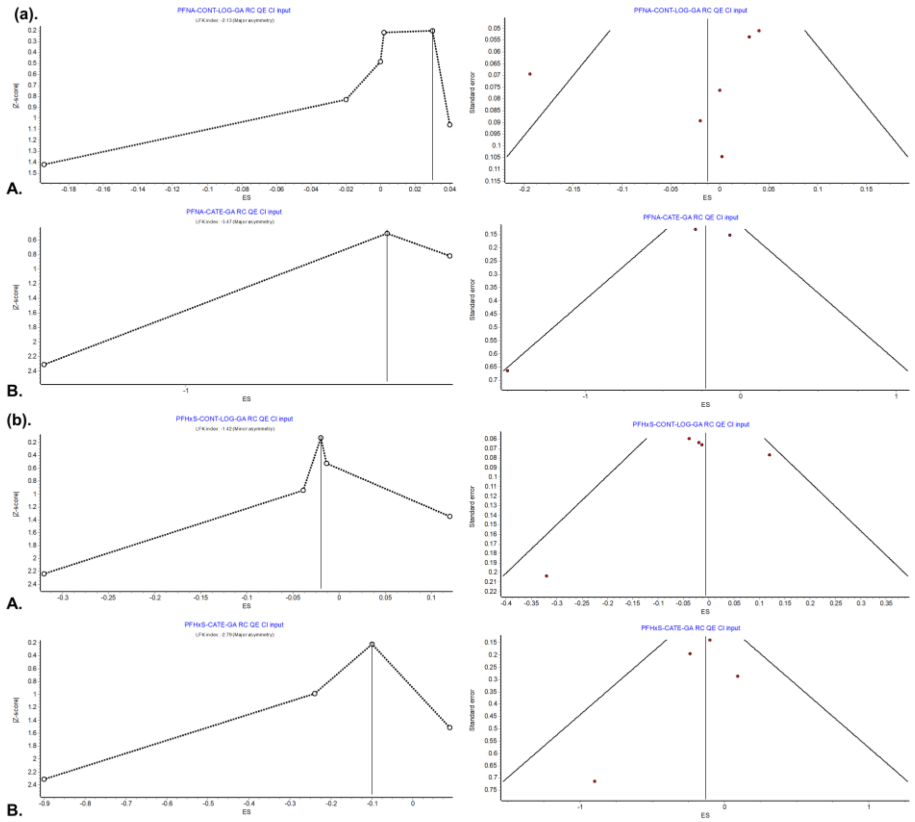

Fig S60. Doi plot and LFK index analysis of publication bias in reported associations between(A)PFDA (B)PFUnDA on GA (gestational age)/weeks for per 1 ln(ng/ml) increment of exposure.

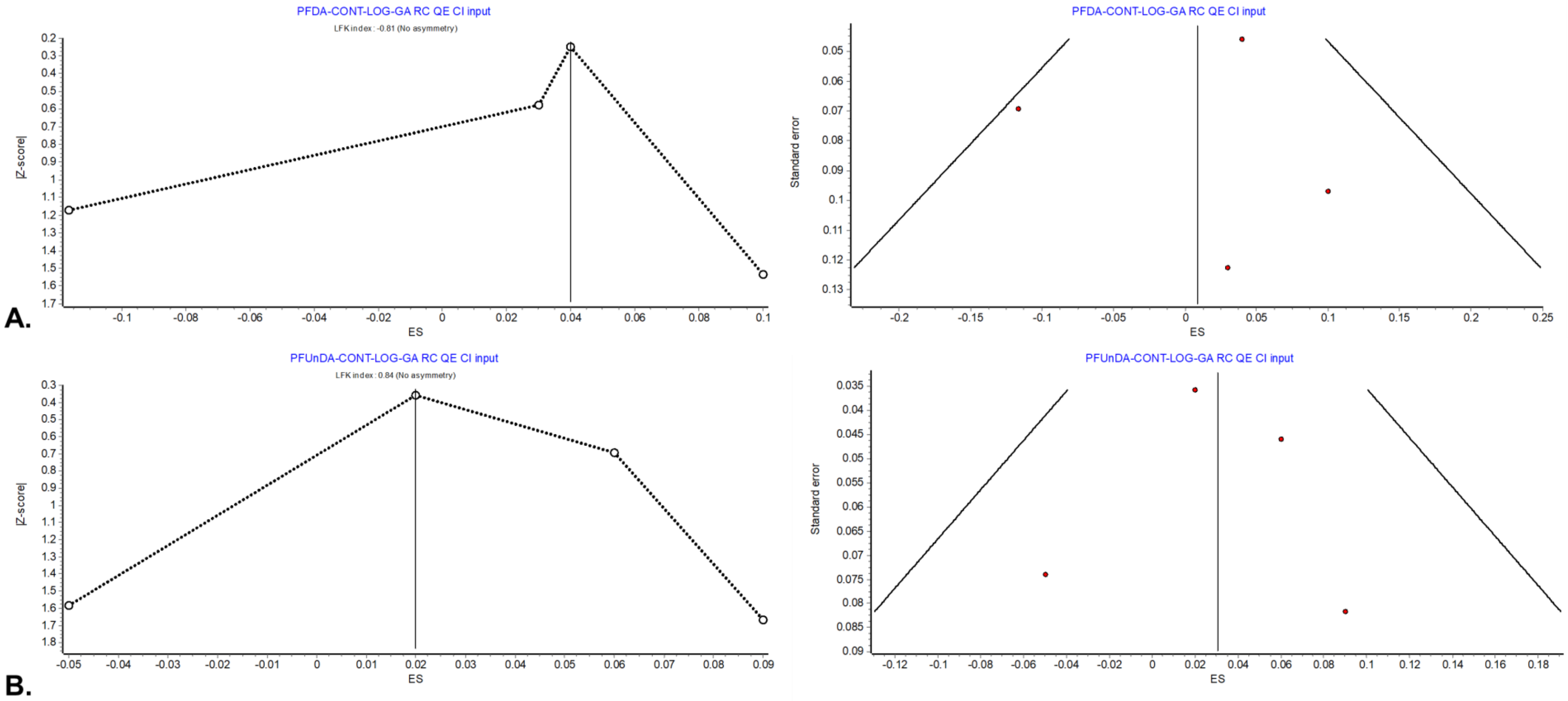

Fig S61. Doi plot and LFK index analysis of publication bias in reported associations between PFHxS on PI (Ponderal index)/g/cm2 *100 for (A) per 1 ln(ng/ml) increment and (B) per 1 ng/ml increment of exposure.

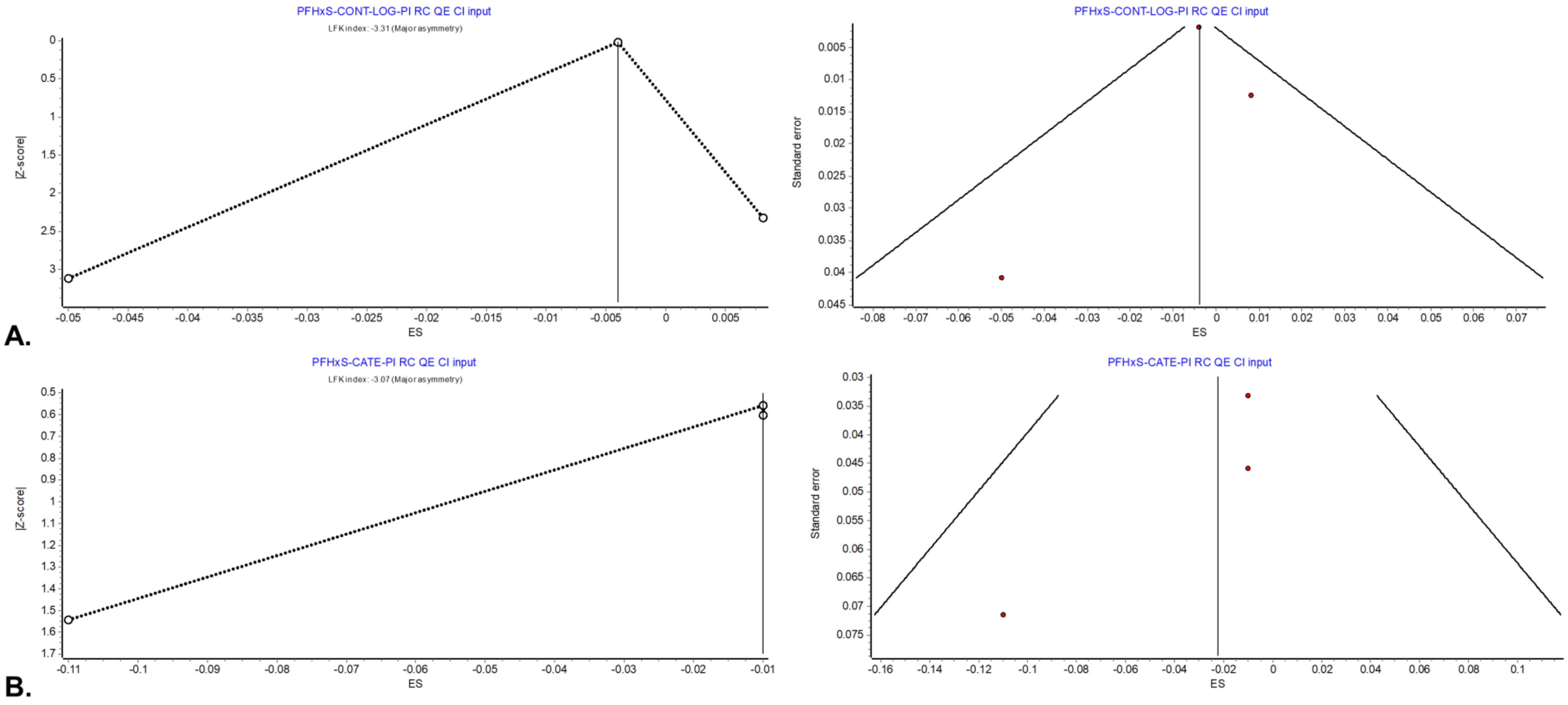

Fig S62. Doi plot and LFK index analysis of publication bias in reported associations between (a)PFNA (b)PFUnDA (c)PFDA on PI (Ponderal index)/g/cm2 *100 for per 1 ln(ng/ml) increment of exposure.

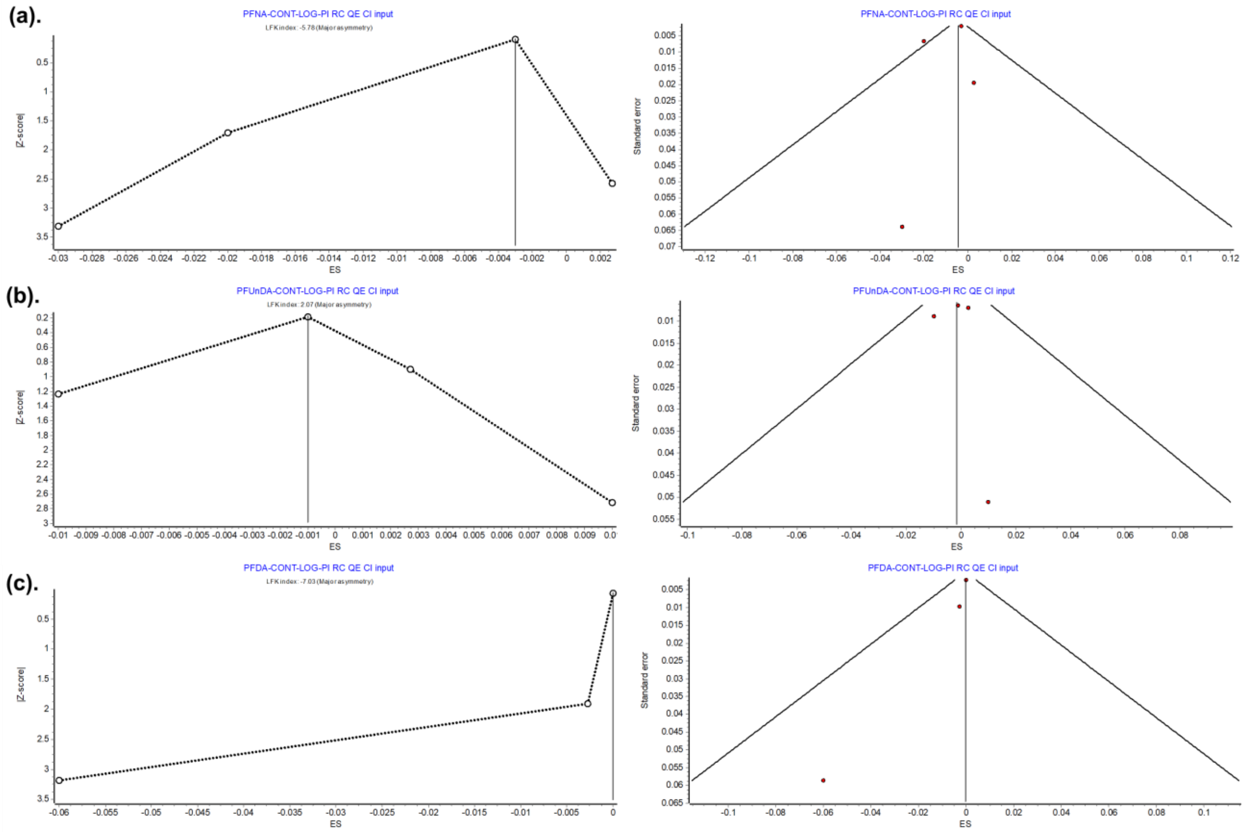

Fig S63. Doi plot and LFK index analysis of publication bias in reported associations between (a)PFNA (b) PFHxS on PTB (preterm birth) for per 1 ln(ng/ml) increment and (B) for high versus low categories of exposure.

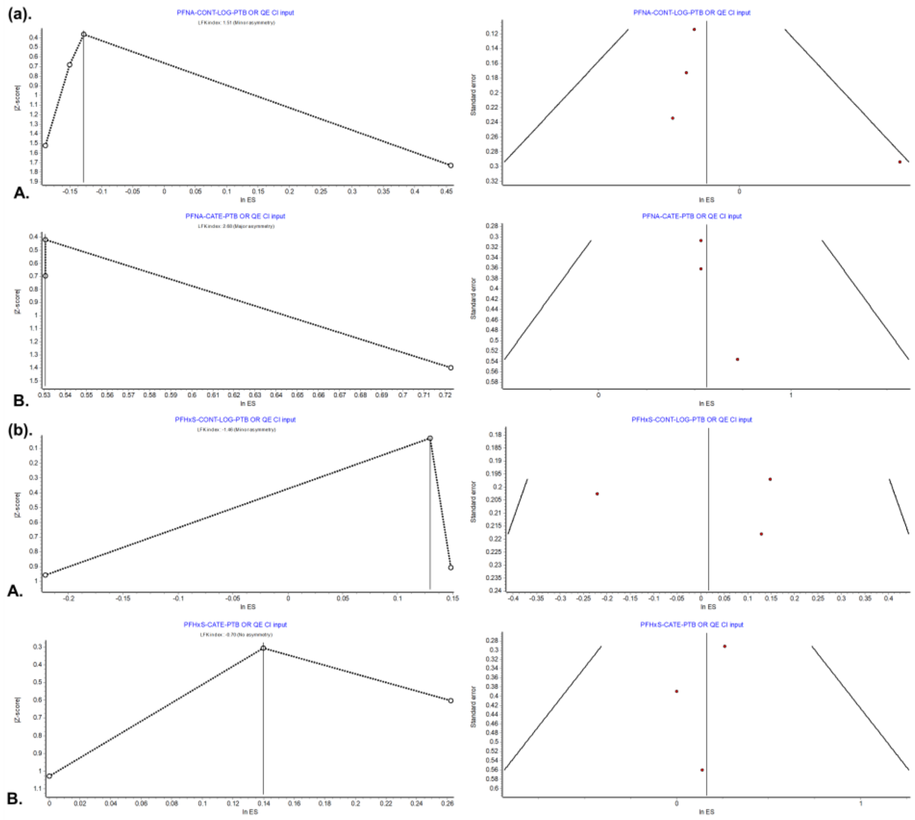

Fig S64. Doi plot and LFK index analysis of publication bias in reported associations between PFNA on LBW (low birth weight) for per 1 ln(ng/ml) increment of exposure.

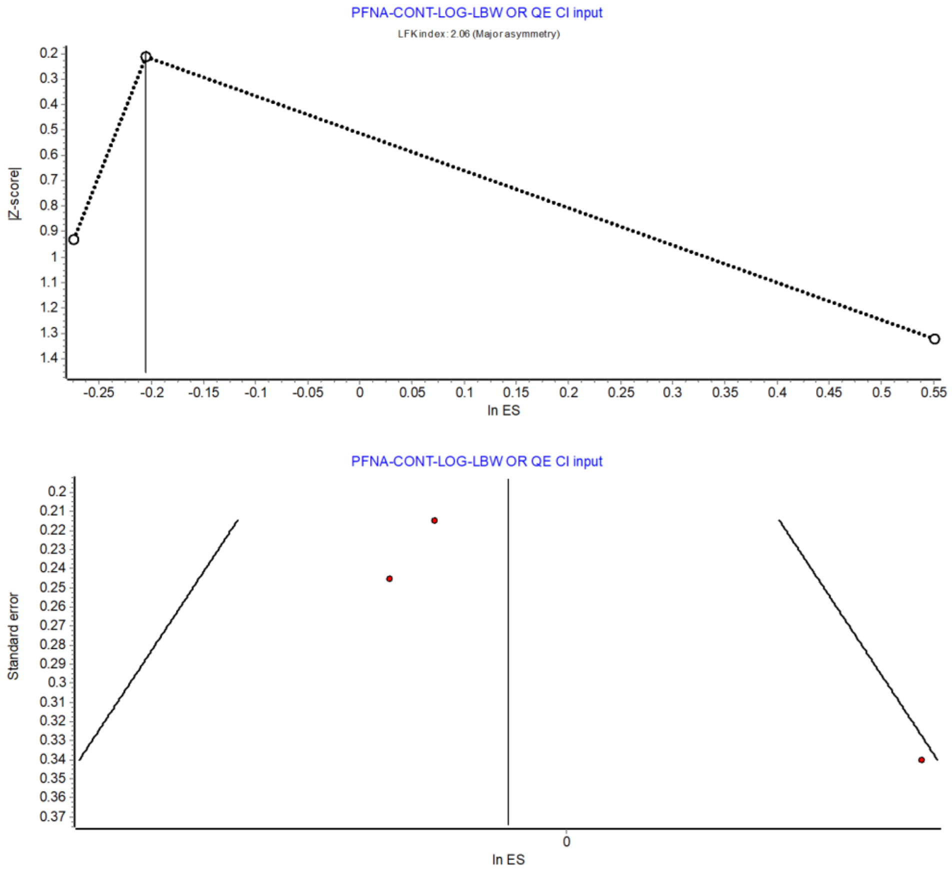

Fig S65. Doi plot and LFK index analysis of publication bias in reported associations between (a)PFNA (b)PFUnDA on SGA (small for gestational age) for per 1 ln(ng/ml) increment of exposure.

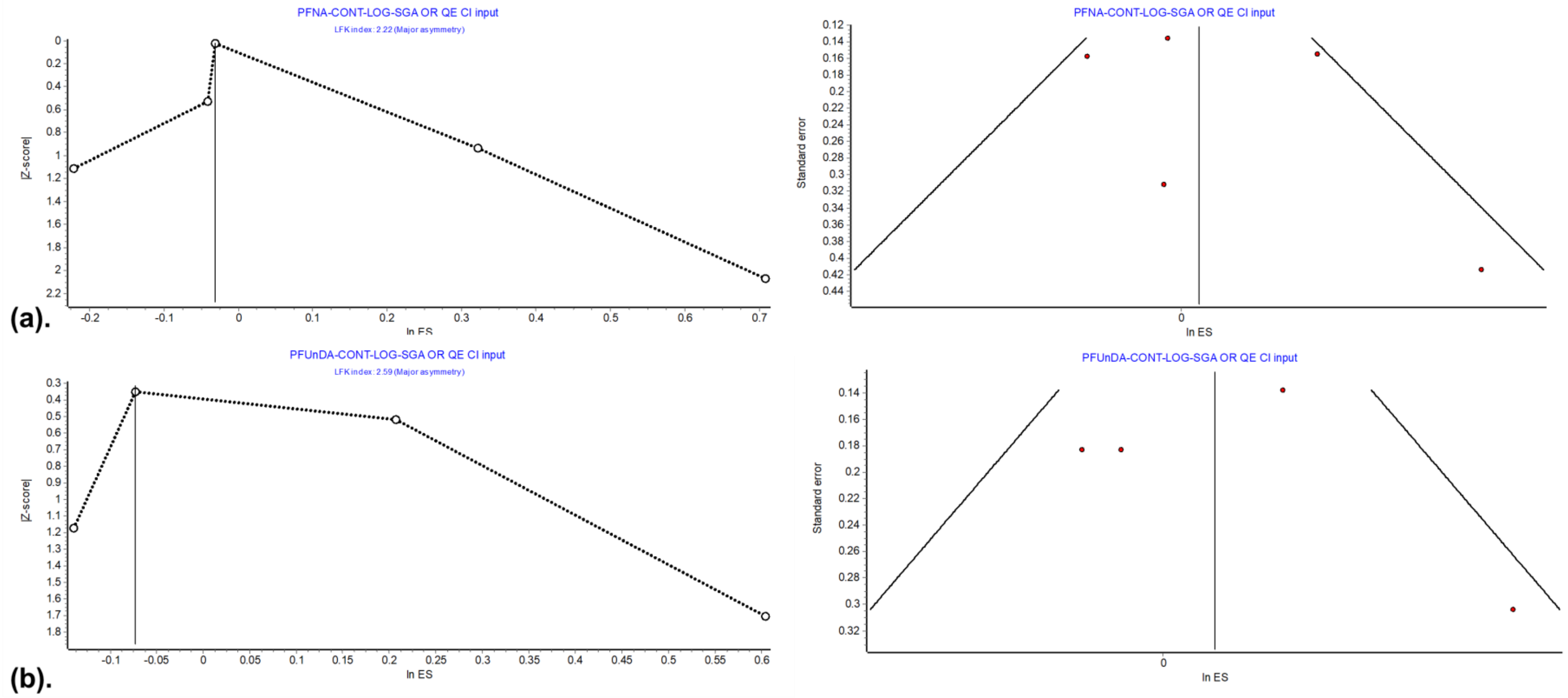

Supplement: Supplementary file 11 [file Data_Sheet_1.pdf]
